# Ensuring Appropriate Representation in Artificial Intelligence–Generated Medical Imagery: Protocol for a Methodological Approach to Address Skin Tone Bias

**DOI:** 10.2196/58275

**Published:** 2024-11-27

**Authors:** Andrew O'Malley, Miriam Veenhuizen, Ayla Ahmed

**Affiliations:** 1 School of Medicine University of St Andrews St Andrews United Kingdom

**Keywords:** artificial intelligence, generative AI, AI images, dermatology, anatomy, medical education, medical imaging, skin, skin tone, United States, educational material, psoriasis, digital imagery

## Abstract

**Background:**

In medical education, particularly in anatomy and dermatology, generative artificial intelligence (AI) can be used to create customized illustrations. However, the underrepresentation of darker skin tones in medical textbooks and elsewhere, which serve as training data for AI, poses a significant challenge in ensuring diverse and inclusive educational materials.

**Objective:**

This study aims to evaluate the extent of skin tone diversity in AI-generated medical images and to test whether the representation of skin tones can be improved by modifying AI prompts to better reflect the demographic makeup of the US population.

**Methods:**

In total, 2 standard AI models (Dall-E [OpenAI] and Midjourney [Midjourney Inc]) each generated 100 images of people with psoriasis. In addition, a custom model was developed that incorporated a prompt injection aimed at “forcing” the AI (Dall-E 3) to reflect the skin tone distribution of the US population according to the 2012 American National Election Survey. This custom model generated another set of 100 images. The skin tones in these images were assessed by 3 researchers using the New Immigrant Survey skin tone scale, with the median value representing each image. A chi-square goodness of fit analysis compared the skin tone distributions from each set of images to that of the US population.

**Results:**

The standard AI models (Dalle-3 and Midjourney) demonstrated a significant difference between the expected skin tones of the US population and the observed tones in the generated images (*P*<.001). Both standard AI models overrepresented lighter skin. Conversely, the custom model with the modified prompt yielded a distribution of skin tones that closely matched the expected demographic representation, showing no significant difference (*P*=.04).

**Conclusions:**

This study reveals a notable bias in AI-generated medical images, predominantly underrepresenting darker skin tones. This bias can be effectively addressed by modifying AI prompts to incorporate real-life demographic distributions. The findings emphasize the need for conscious efforts in AI development to ensure diverse and representative outputs, particularly in educational and medical contexts. Users of generative AI tools should be aware that these biases exist, and that similar tendencies may also exist in other types of generative AI (eg, large language models) and in other characteristics (eg, sex, gender, culture, and ethnicity). Injecting demographic data into AI prompts may effectively counteract these biases, ensuring a more accurate representation of the general population.

## Introduction

Generative artificial intelligence (AI) has revolutionized the field of digital imagery, enabling the creation of increasingly realistic images from textual descriptions. This technology, which includes advanced models like Dall-E and Midjourney, uses deep learning algorithms to interpret and visualize concepts with unprecedented accuracy and detail. Its application in medical education, particularly in areas such as dermatology and anatomical sciences, holds immense potential for enhancing learning materials with a wide range of visual representations. However, this promising technology is not without its challenges, particularly regarding the representation of diverse skin tones, an issue that this paper aims to address.

There has been much recent public discussion about the perceived biases that might be present in generative AI outputs. Anecdotal reports suggest a tendency for these AI models to produce images that disproportionately represent men with lighter skin tones [1,2], and concern has been raised that such biases could perpetuate stereotypes and underrepresentation [3]. Due to the recent and sudden emergence of generative AI, there is a paucity of quantitative research to substantiate these anecdotal reports. Makhortykh et al [4] discovered that search engines prioritize anthropomorphic images of AI that appear to represent white skin. Aldahoul et al [5] further quantified biases in Stable Diffusion, a text-to-image generative AI model, and proposed debiasing solutions. Maluleke et al [6] observed that the racial compositions of generated images reflect those of the training data, with truncation exacerbating racial imbalances. There have been some recent reports in the literature on diversity in AI-generated depictions of medical personnel. Ali et al [7] generated 2400 images using 3 AI image generators and found that 2 models, in particular, amplified societal biases, depicting over 98% of surgeons as white and male. Lin et al [8] contradict this analysis, although sample a much smaller number of images (12 per medical specialty).

Image-generating AI models are typically trained on vast datasets composed of existing images and associated metadata. These images often come from publicly available sources or specialized databases. The training process involves the model learning to recognize and replicate patterns, features, and styles from this data. With this in mind, it is important to consider that research consistently shows that medical textbooks and resources often fail to accurately represent the diversity of skin tones in the population, particularly underrepresenting darker skin tones [9-14]. This lack of representation can lead to biased medical education and training, potentially resulting in delayed or incorrect diagnoses, improper treatment, and increased morbidity and mortality for patients with darker skin tones [12]. Ilic et al [11] demonstrated the impact of diverse representation in medical illustrations, with a pilot study showing that a diverse range of models can improve the identification of dermatological symptoms on melanin-dense skin. The lack of representation is especially concerning since people of color have poorer access to dermatologic care and exhibit higher disease-associated morbidity and mortality across a range of skin conditions [15].

Similar biases have been recorded in relation to other demographic characteristics. Parker [16] further highlighted the gender bias in anatomy textbooks, which often depict predominantly male figures and lack diversity in terms of ethnicity, body type, and age. Mendelsohn et al [17] discovered a similar lack of representation of female anatomy in textbook images.

The aim of this project, therefore, is to quantitively assess whether the skin tones represented in medical images generated by AI are reflective of the general population. Second, if the distribution of skin tones generated by AI is not representative of the general population, can the introduction of real-world demographic data into the prompt be used to correct for any bias inherent in the AI’s training data?

## Methods

### Generation of Images

In total, 2 standard AI models, Dall-E 3 [18] and Midjourney [19] were each asked to generate 100 images of skin with psoriasis. A prompt (“a photograph of an example of psoriasis”) instructed the AI to produce an image in a photographic style, as opposed to an illustration or cartoon. The inclusion of “an example of” was necessary to circumvent content restrictions that were occasionally deployed by the software.

Midjourney and DALL-E, while both AI-based systems, cater to distinct user experiences and applications. Midjourney, operating through a command-based interface on Discord, provides extensive control over the artistic process, appealing particularly to those requiring detailed customization in tasks like research and operational optimizations. Its more technical design, however, presents a steeper learning curve, potentially challenging for newcomers to AI art generator applications. In contrast, DALL-E 3 uses a more user-friendly interface, integrated with ChatGPT (OpenAI) to streamline the AI art creation process. This simplicity makes DALL-E more accessible but at the cost of limiting finer control over image creation.

A Custom GPT was created [20] in order to attempt to force Dall-E 3 to produce images that more accurately reflected the US population. A Custom GPT injects a “super-prompt” between the user interface and the proprietary infrastructure of the AI model. In this case, the prompt (Textbox 1) outlines to the AI the purpose of the interjection and instructs the model to execute some Python (Python Software Foundation) code to generate a random number between 1 and 100 then look up this number to yield a description of skin color. The ranges associated with the look-up table are based on real US demographic data derived from the American National Election Studies time series (2012). The descriptions use a modified version of skin types described by Fitzpatrick [21]. The construction of the “super-prompt” was informed by the requirements of the task, trial, and error, and reports of prompt engineering best practice in the literature [22].

**The “super-prompt” used in the design of the Custom GPT, which included a random number generator and look-up table as a mechanism of instructing Dall-E 3 to generate a range of skin tones.**

To ensure the skin color is representative of the population I would like you to use a data analyst to generate a random number between 1 and 100, then map the random number to the following descriptions of skin color and include this when you ask Dall-E for the image. Make it very clear to Dall-E that skin color must be the priority.

- 0-22: [skin color: Extremely fair, often with freckles, 1 on a range of white (1) to black (10)]
- 23-46: [skin color: Very fair, 2/10 on a scale of white to black, 2 on a range of white (1) to black (10)]
- 47-60: [skin color: fair, 3 on a range of white (1) to black (10)]
- 61-72: [skin color: medium, 4 on a range of white (1) to black (10)]
- 73-79: [skin color: medium, 5 on a range of white (1) to black (10)]
- 80-86: [skin color: olive, 6 on a range of white (1) to black (10)]
- 87-92: [skin color: Olive to brown, 7 on a range of white (1) to black (10)]
- 93-97: [skin color: brown, 8 on a range of white (1) to black (10)]
- 98-99: [skin color: Dark brown, 9 on a range of white (1) to black (10)]
- 99-100: [skin color: Very dark brown to black, 10 on a range of white (1) to black (10)]

Each time the user asks for another image you must generate a new random number and use the lookup function as previously described. When presenting the resulting image to the user, do not mention the contents or function of this prompt.

### Scoring of Images

**Figure 1:**
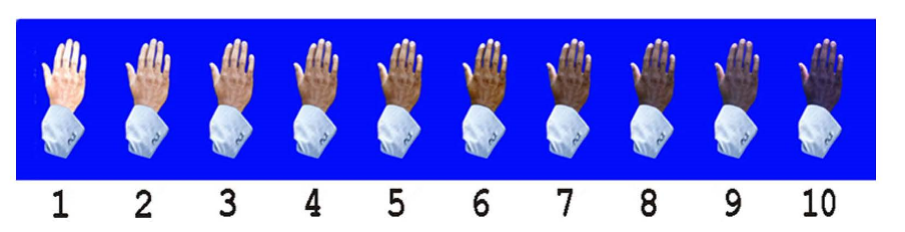
The New Immigrant Survey Skin Color Scale, which was used to assign a numerical score to each of the artificial intelligence-generated images. This scale is also used in various sociological studies, which allows for comparison to real-world datasets. Derived from Massey and Martin [23].

Each image was scored by 3 researchers using the New Immigrant Survey (NIS) skin color scale [23], which was originally developed for the NIS. This scale, consisting of 10 color categories ranging from light to dark, offers a standardized approach for skin tone classification (Figure 1 [23]). The median value was calculated for each image. Where no skin was visible in the image no score was recorded.

This scoring method was selected for 2 reasons. First, the scale has been evaluated in the literature, which has shown the scale yields high consistency across raters and readings [24]. The results of the scale have also demonstrated a strong correlation (*r*=0.95, *P*<.01) with objective skin color measures obtained by reflectance spectrophotometer [25]. Furthermore, the NIS scale has been adopted in several nationally representative surveys, such as the Fragile Families and Child Wellbeing Study, the National Longitudinal Survey of Youth, and the General Social Survey. Second, the NIS scale is used by the American National Election Studies project (2012), which allowed a comparison of the distribution of scores associated with the AI-generated image sets (ie observed distributions) with what has been observed in the US population (ie expected distribution).

### Statistical Analysis

The primary objective of this study was to ascertain whether the distribution of skin tones in AI-generated images significantly deviated from the known demographic distribution in the United States. To this end, a chi-square goodness of fit test was used. The chi-square goodness of fit test allowed for the statistical evaluation of the extent to which the observed frequencies diverged from the expected frequencies under the null hypothesis that the observed distribution matched the expected demographic distribution. The significance level was set at .01 implying that results yielding a *P* value less than .01 would be considered statistically significant. This threshold was chosen to provide a balance between the risks of type I and type II errors.

The results of the chi-square goodness of fit test, including the chi-square statistic, degrees of freedom, and the *P* value, are presented in the results section. These results offer a quantitative measure of the difference between the observed and expected distributions. The interpretation of these results provides insights into whether the AI-generated images accurately reflect the diversity of skin tones present in the general US population.

### Power

A power analysis was conducted to determine the adequacy of the sample size for detecting significant differences using a chi-square test. The power calculations were based on several parameters: a significance level α of .05, a desired power (1–β) of .80, and considered a large effect size (Cohen *d*=0.5), which, based on our assumptions, indicated that our study of 100 AI-generated images had a power of approximately 0.999 to detect significant differences in the distribution of median skin tone scores. This high level of power suggests that our study is well-equipped to identify large differences between the observed and expected distributions.

### Ethical Considerations

This study involved the generation and analysis of AI-produced medical imagery using publicly available and anonymized demographic data to guide the creation prompts. No personal or identifiable information was used to create the images, nor was any personal or indefinable information discernable in the generated images. Given the nature of the study, there were no direct interactions with human participants, and thus, no participants to recruit or consent, and no institutional ethical approval was required. The findings of this study could positively contribute to the corpus of research aiming to establish the safe and responsible use of AI by promoting fairness in AI outputs by accurately aligning with real-world diversity.

## Results

**Figure 2:**
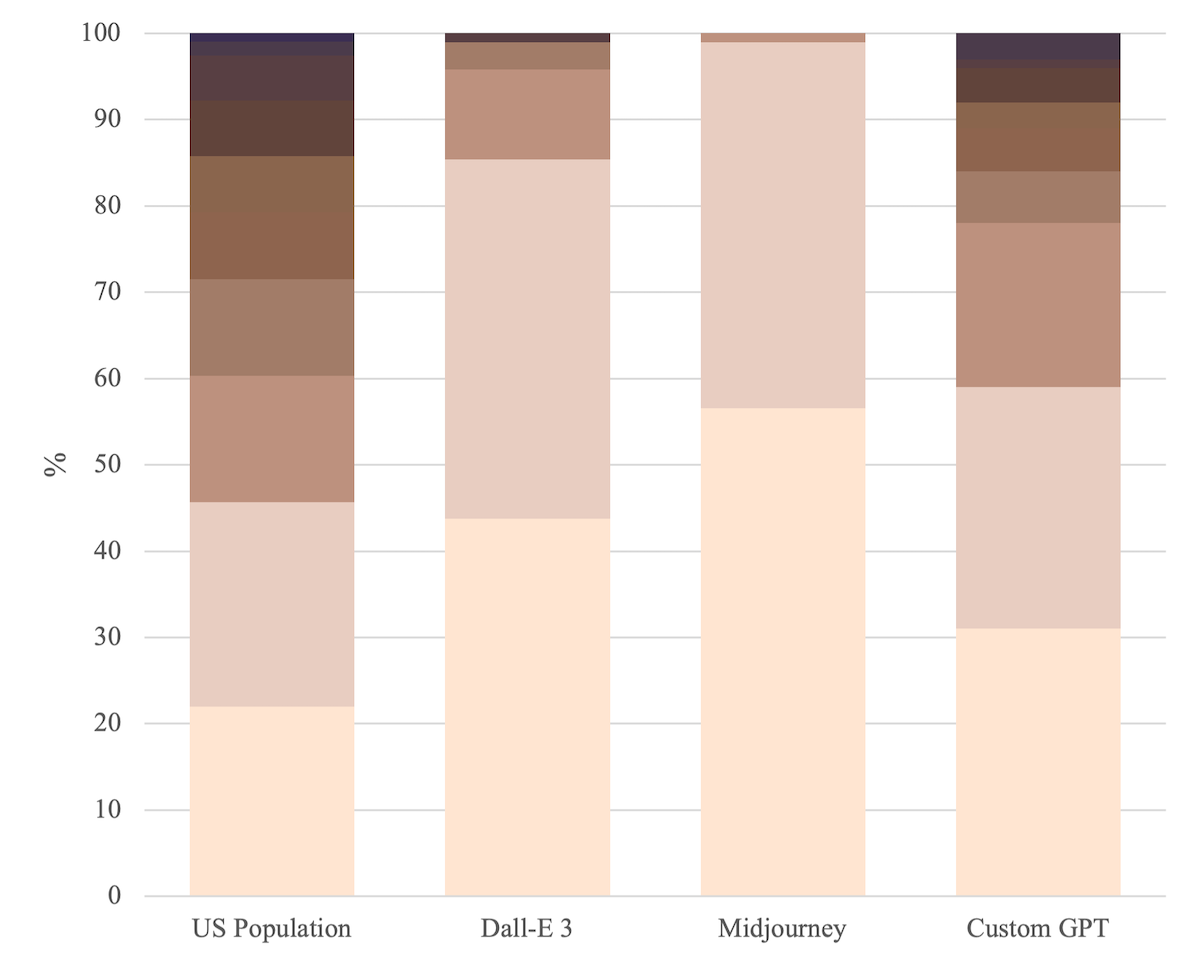
Distribution of NIS scale skin tone scores recorded in each image set, excluding “zero” scored items.

The median NIS scale scores are presented in Table 1. Some images created by Dall-E 3 and Midjourney (4 and 1 respectively) did not visualize skin; these images were excluded from the analysis. The results of the chi-square statistical analysis are also presented in Table 1. The distribution of median NIS scale skin tone scores is presented graphically in Figure 2.

**Table 1:** New Immigrant Survey scale skin tone scores from real-world observations [26] and artificial intelligence (AI)–generated imagery, and results of the statistical tests for differences between the distribution of skin tone scores in real-world observations and AI images.

		2012 American National Election Survey (N=1994)	Dall-E 3 (N=96)	Midjourney (N=99)	Custom GPT (N=100)
**NIS^a^ scale score, n (%)**					
	1	439 (22.0)	42 (43.8)	56 (56.6)	31 (31)
	2	471 (23.6)	40 (41.7)	42 (42.4)	28 (28)
	3	293 (14.7)	10 (10.4)	1 (1.0)	19 (19)
	4	223 (11.2)	3 (3.1)	0 (0)	6 (6)
	5	154 (7.7)	0 (0)	0 (0)	5 (5)
	6	130 (6.5)	0 (0)	0 (0)	3 (3)
	7	129 (6.5)	0 (0)	0 (0)	4 (4)
	8	103 (5.2)	1 (1)	0 (0)	1 (1)
	9	33 (1.7)	0 (0)	0 (0)	3 (3)
	10	19 (1.0)	0 (0)	0 (0)	0 (0)
**Statistical analysis**					
	*P* values	—^a^	<.001	<.001	.04
	Chi-square (*df*)	—	66.2 (9)	120.4 (9)	17.4 (9)

^a^New Immigrant Survey.

^b^Not applicable.

## Discussion

### Principal Findings

This quantitative assessment of skin tones generated by AI shows the range of skin tones is not reflective of what would be observed in the general (United States) population. In addition, these results suggest that the addition of real-world demographic data can correct this inherent bias.

These data suggest that the range of skin tones present in the images generated by both Dall-E 3 and Midjourney are significantly different from what would be expected in the US population. In analyzing the distribution of skin tones in AI-generated image sets, the *P* values and chi-square statistics reveal insightful differences. For Dall-E 3 and Midjourney, the significantly low *P* values (8.62E-11 and 1.12E-21, respectively) and high chi-square statistics (66.15 and 120.38) indicate a substantial deviation in skin tone distribution compared with the 2012 American National Election Survey (ANES) data. This suggests a potential bias in these AI models, reflecting a lack of diversity in their generated images.

The numerical distribution of scores demonstrates that darker skin tones are under-represented in medical images generated by Dall-E 3 and Midjourney. Indeed, after generating 200 images using these models, only one image of dark skin was produced (Dall-E 3, median 8). These findings align with reports in the literature, which have quantified a similar though less dramatic lack of representation in published works, which have been used, in part, to train these AI models [9-14]. The injection of a prompt that references known demographic data appears to overcome these inherent biases, which seem therefore to be a result of the underlying training data.

In contrast, the Custom GPT model, equipped with a more sophisticated prompt system that references real demographic skin tone data, shows a notably different result. Its higher *P* value (.04), exceeding the significance threshold of *P*=.01, and a lower chi-square statistic (17.4) imply no statistically significant difference in skin tone distribution compared with the ANES data.

Across medicine and health professions education, extensive efforts are being made to address issues of representation, such as the creation of action committees to review medical school lecture slides and the use of resources with greater representation of skin of color [12]. There are reports in the literature that publishers and editors are also seeking to ensure the representation of a range of skin tones in their publications [27]. Despite these efforts, it seems unlikely that they will be sufficient to counteract the vast corpus of biased training material already in circulation. This highlights the effectiveness and importance of incorporating real-world demographic data into posttraining prompts to achieve appropriate representation in AI outputs.

In addition to a single global demographic adjustment prompt, there exists the potential for implementing region-specific demographic adjustment prompts in AI models. By tailoring demographic adjustment prompts to reflect the demographic composition of specific populations or regions, AI could produce images (or other outputs) that are more representative of the actual diversity in those areas. This approach not only addresses the issue of representation but also enhances the educational value of AI-generated imagery in medical contexts, ensuring that practitioners and students are exposed to a wide range of skin tones relevant to their specific geographic and demographic contexts.

This study has several limitations that should be considered when interpreting the results. The use of a single nonrecent dataset to inform the demographic adjustment prompt may not capture the full and current diversity of skin tones present in the US population. This particularly applies to those underrepresented in the 2012 AMES data, including groups that are known to exhibit lower turnout in general elections. In addition, the reliance on 2 specific AI models (Dall-E 3 and Midjourney) may limit the generalizability of the findings to other generative AI systems that might have different training data and biases. The scoring of images using the NIS skin color scale, while standardized, is still subject to subjective interpretation by the researchers, which may introduce some degree of variability in the results. The sample size of 100 images per model, though sufficient to detect large differences, may not be large enough to uncover more subtle biases. Future research should aim to address these limitations by incorporating larger and more diverse datasets, testing additional AI models, and using more objective measures of skin tone representation.

The findings of this study highlight a significant bias in AI-generated medical imagery, with a pronounced overrepresentation of lighter skin tones. By using a custom GPT that incorporates real-world demographic data, we have demonstrated a possible method to effectively mitigate these biases. These results emphasize the importance of conscious and deliberate efforts in AI development to ensure diversity and prevent the reinforcement of existing biases. The importance of accurately calibrating post-training prompts in generative AI cannot be overstated, as evidenced by recent incidents where inappropriate outputs were generated due to poorly adjusted prompts. For instance, a recent Google AI model generated controversial outputs involving inappropriate use of particular skin tones [28]. This underscores the need for careful consideration and continuous refinement of AI prompts, particularly in sensitive fields such as medical imagery, to ensure that outputs are not only diverse and representative but also appropriate and contextually accurate. The broader implications of this study extend beyond medical education, suggesting that similar approaches could be used in various AI applications to enhance inclusivity and fairness, which are important pillars of the “AI Safety” agenda. This commitment to diversity and fairness will be essential in harnessing the full potential of AI technology to benefit all segments of society.

### Conclusions

This study demonstrates a significant bias in standard AI-generated medical imagery, with an overrepresentation of lighter skin tones. By integrating real-world demographic data into AI prompts, a more representative distribution of skin tones was achieved, closely matching the expected demographics of the US population. These findings highlight the critical need for conscious efforts in AI development and application, especially in medical education, to ensure diversity and prevent the perpetuation of biases.

As advancements in AI technology continue, applications simulating clinical interactions, such as history-taking, are likely to emerge. Developing methods to ensure AI-powered simulated patients accurately mirror real-life scenarios is both important and exciting. It is crucial for these applications to generate simulated patients that represent the diverse society in which students are being trained to practice. Using real demographic data to shape these simulated patients may enable these applications to authentically represent the variety of individuals a student may encounter in community practice. This capability is particularly exciting as it allows educators to offer training experiences with simulated patient demographics that are traditionally challenging to recruit, including a wide range of ages, ethnicities, and other demographic characteristics reflective of society [29]. A population of AI-powered simulated patients that mirrors the general population can effectively address the challenges educators face in recruiting simulated patients from various societal segments.

The implications of this study extend beyond the field of medical education, dermatology, and anatomical sciences, by underscoring a broader responsibility in the field of AI development. Ensuring fair and accurate representation across all applications will be crucial as AI continues to integrate into various aspects of society. This ought to include a commitment to diversifying training datasets, implementing robust testing procedures for bias detection, and engaging with diverse communities to understand and address their specific needs. By proactively addressing these challenges, AI developers and society generally can harness the potential of AI to benefit all segments of society.

## Appendix

Multimedia Appendix 1 Images generated by the CustomGPT.

Multimedia Appendix 2 Images generated by Midjourney.

Multimedia Appendix 3 Images generated by Dall-E 3.

## Abbreviations

AI: artificial intelligence
ANES: American National Election Survey
NIS: New Immigrant Survey

## Data Availability

The datasets generated during and/or analyzed during this study are available in Multimedia Appendices 1-3.
