# Supplementary figures and images for: Ensuring Appropriate Representation in Artificial Intelligence–Generated Medical Imagery: Protocol for a Methodological Approach to Address Skin Tone Bias (part 1 of 3)
Source: JMIR AI. 2024 Nov 27;3:e58275. doi: 10.2196/58275 (PMC11635324; doi:10.2196/58275)

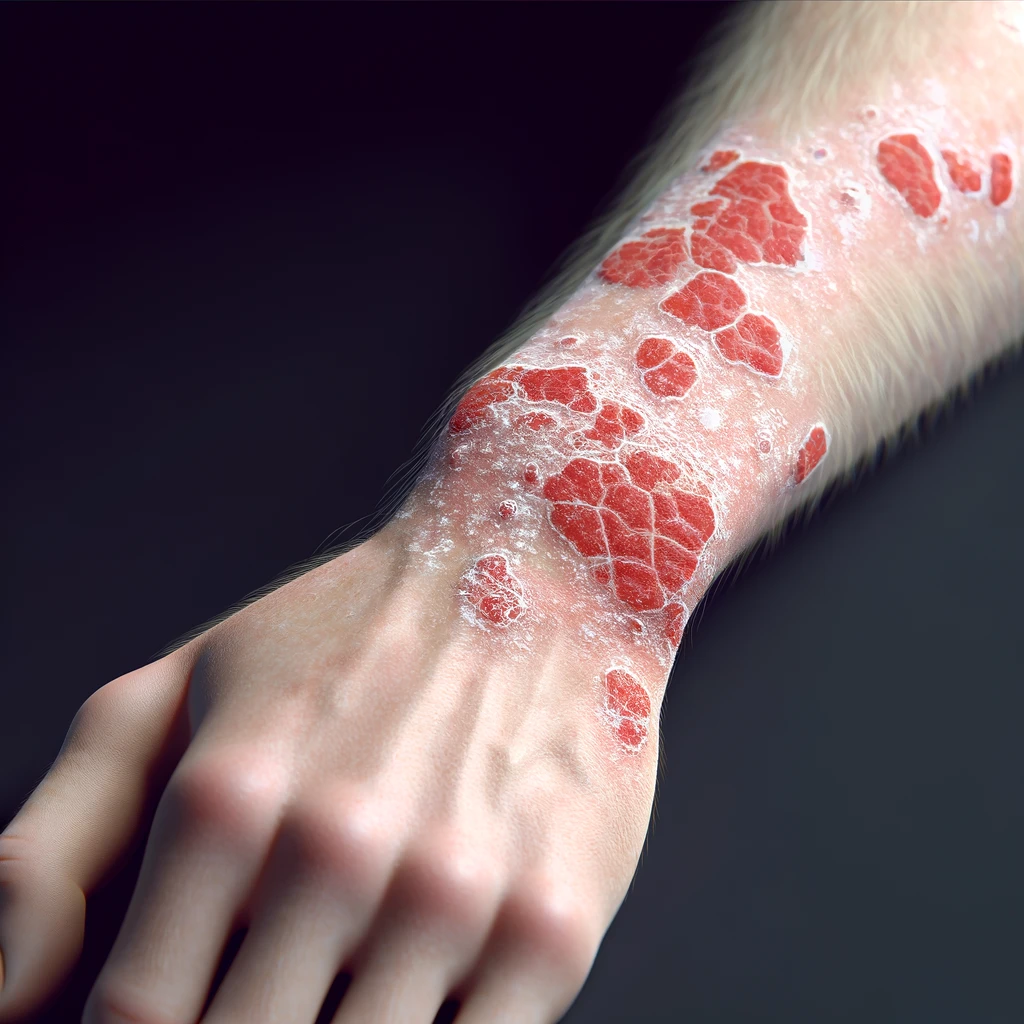

Supplement: Multimedia Appendix 1 [file ai_v3i1e58275_app1.zip › 82 custom GPT.png]

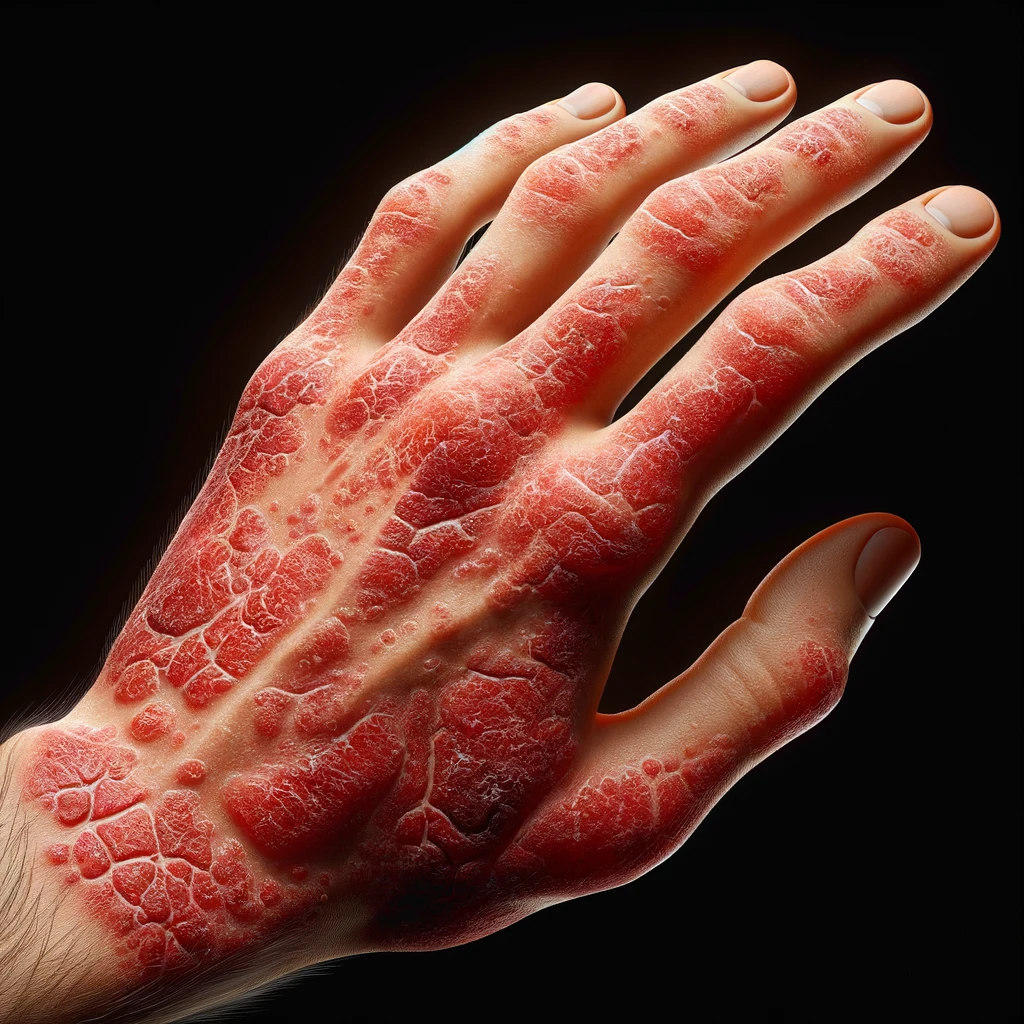

Supplement: Multimedia Appendix 1 [file ai_v3i1e58275_app1.zip › 42 custom GPT.png]

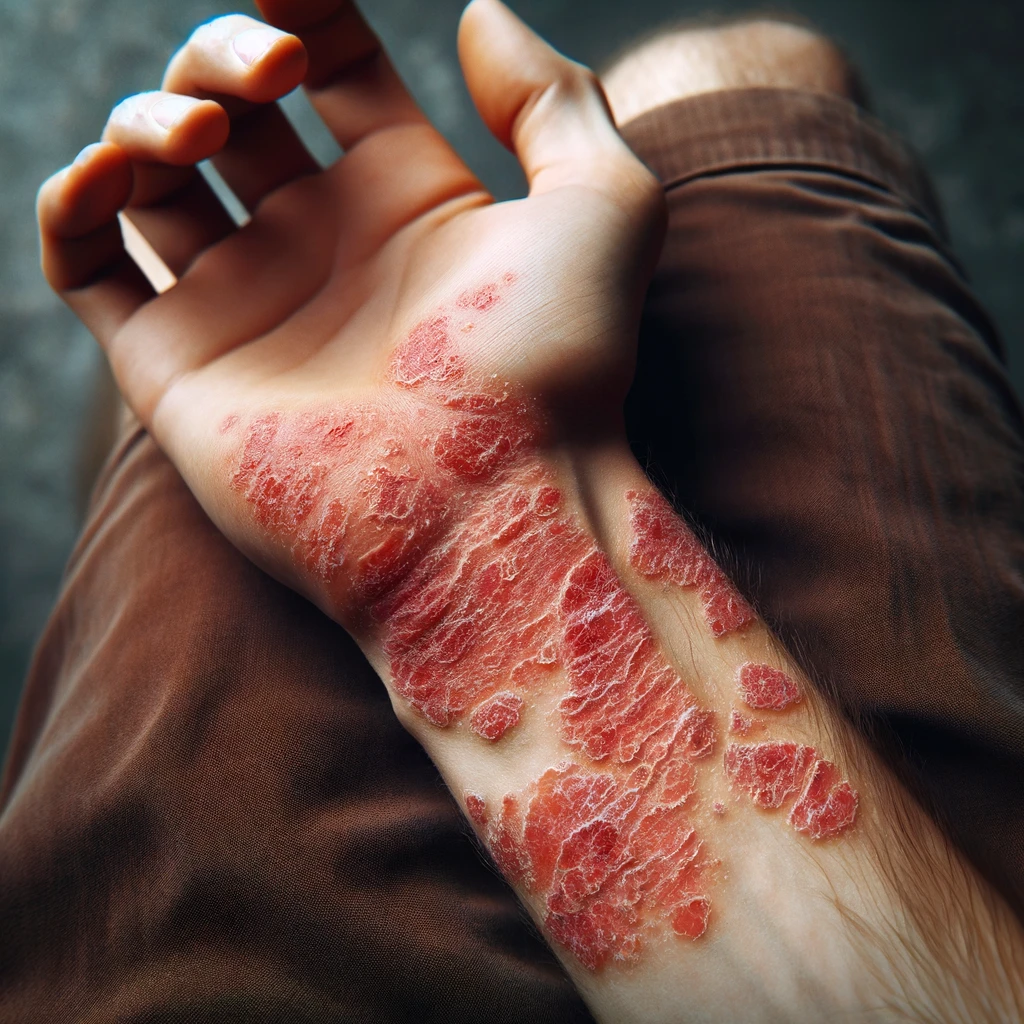

Supplement: Multimedia Appendix 1 [file ai_v3i1e58275_app1.zip › 31 custom GPT.png]

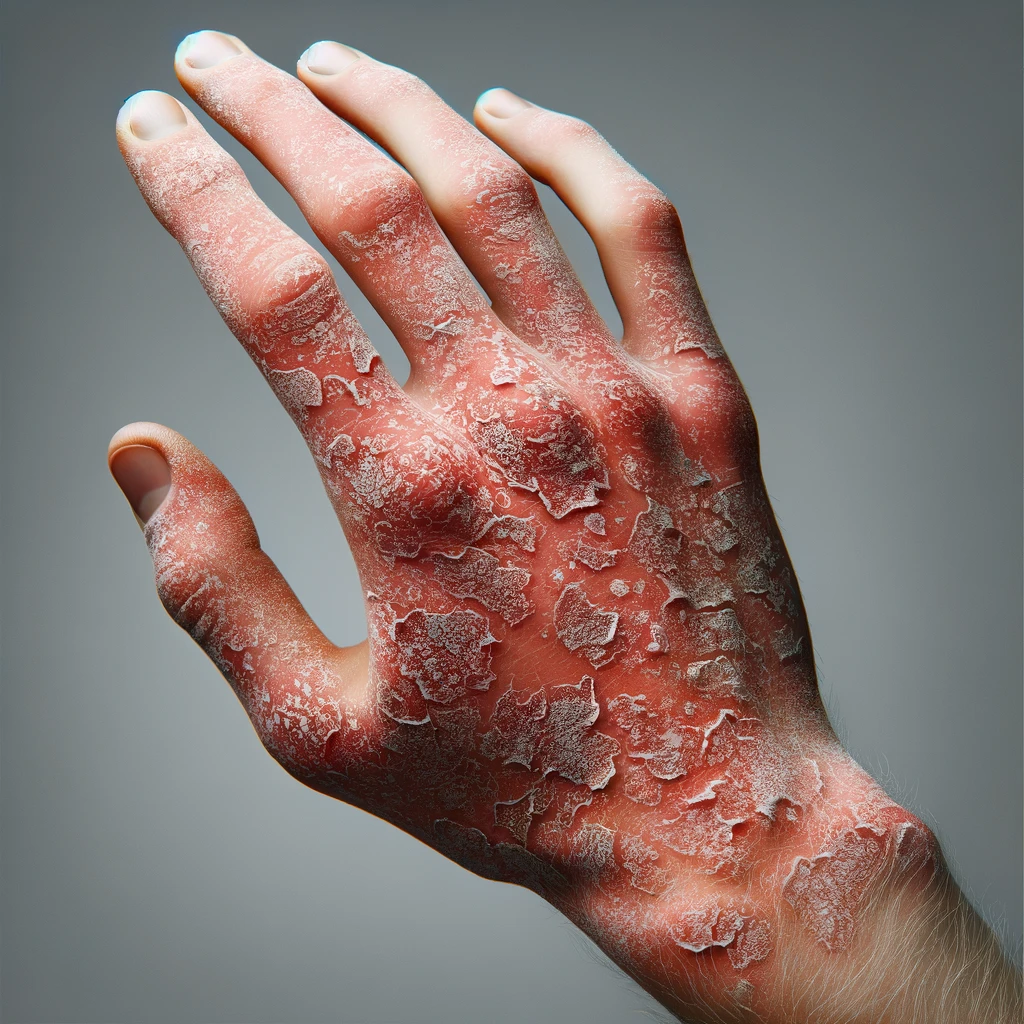

Supplement: Multimedia Appendix 1 [file ai_v3i1e58275_app1.zip › 61 custom GPT.png]

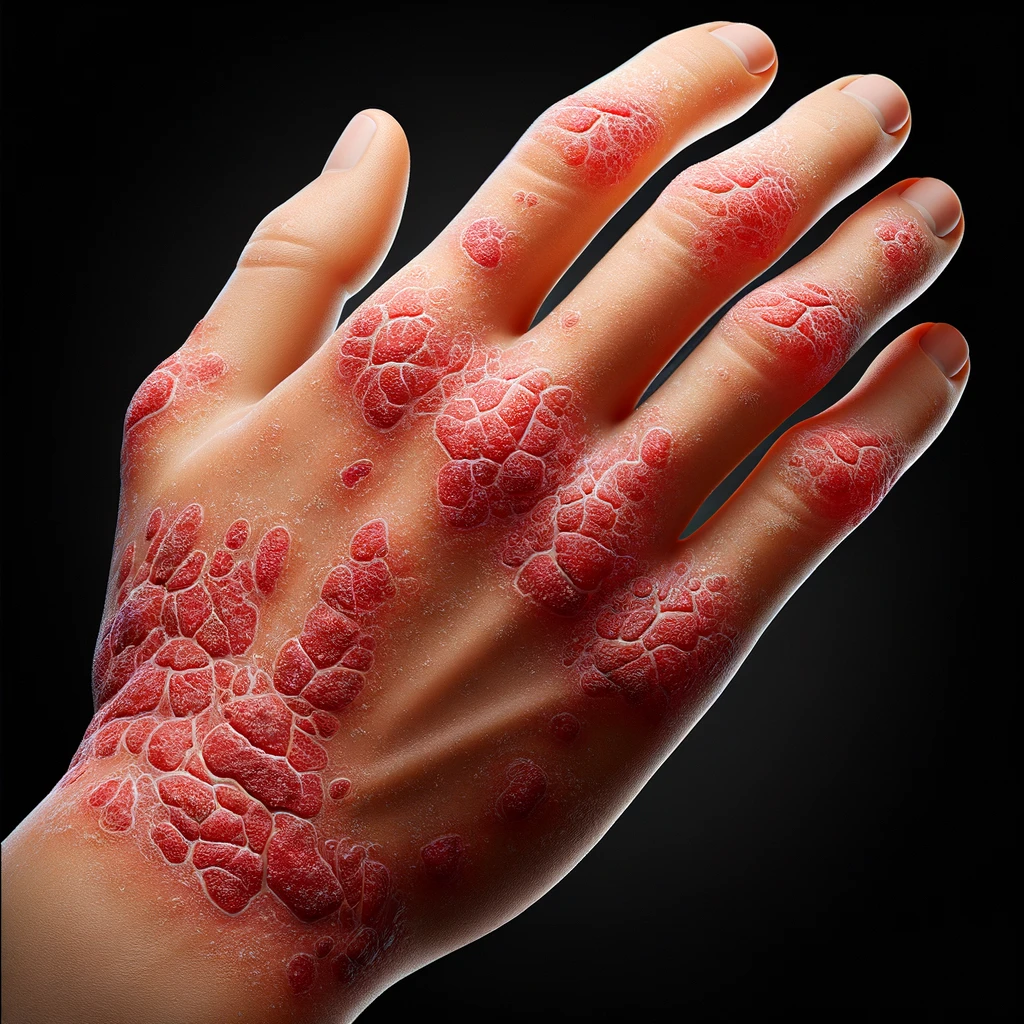

Supplement: Multimedia Appendix 1 [file ai_v3i1e58275_app1.zip › 11 custom GPT.png]

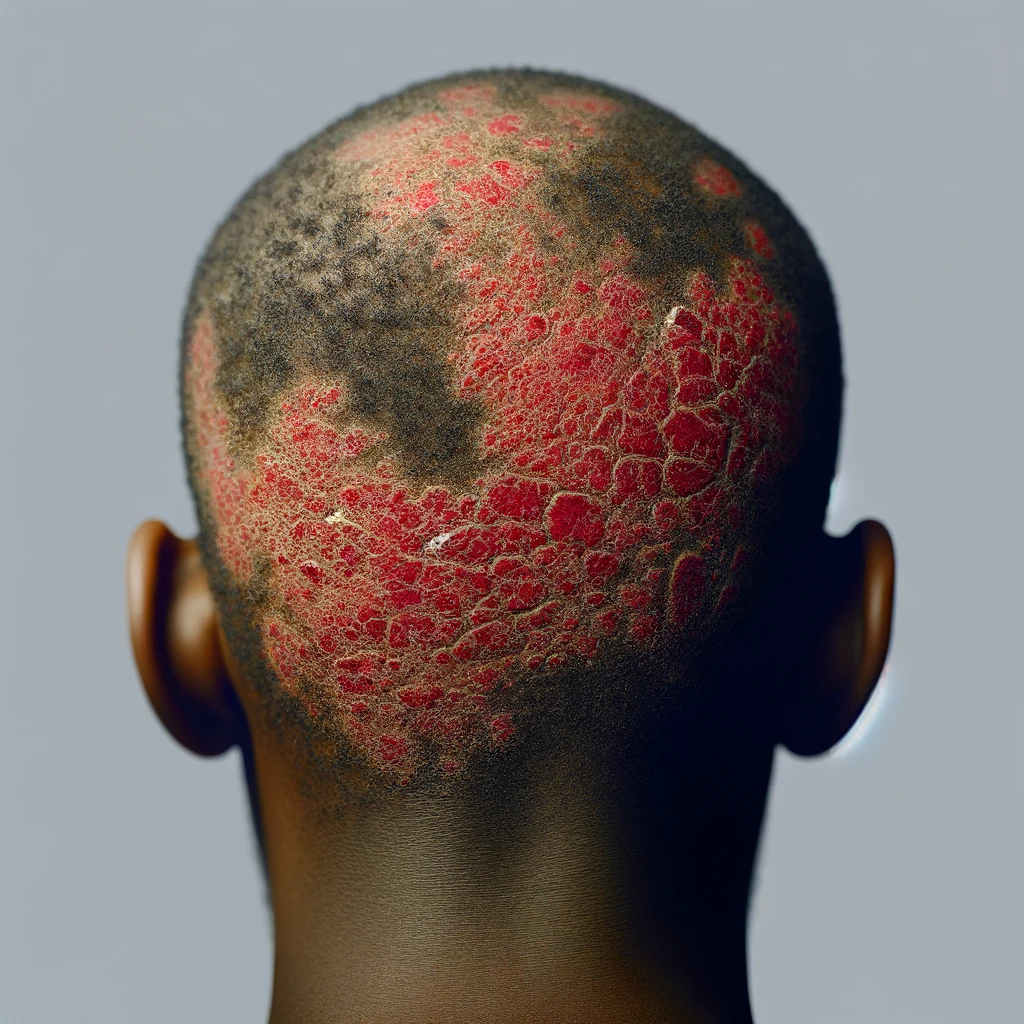

Supplement: Multimedia Appendix 1 [file ai_v3i1e58275_app1.zip › 39 custom GPT.png]

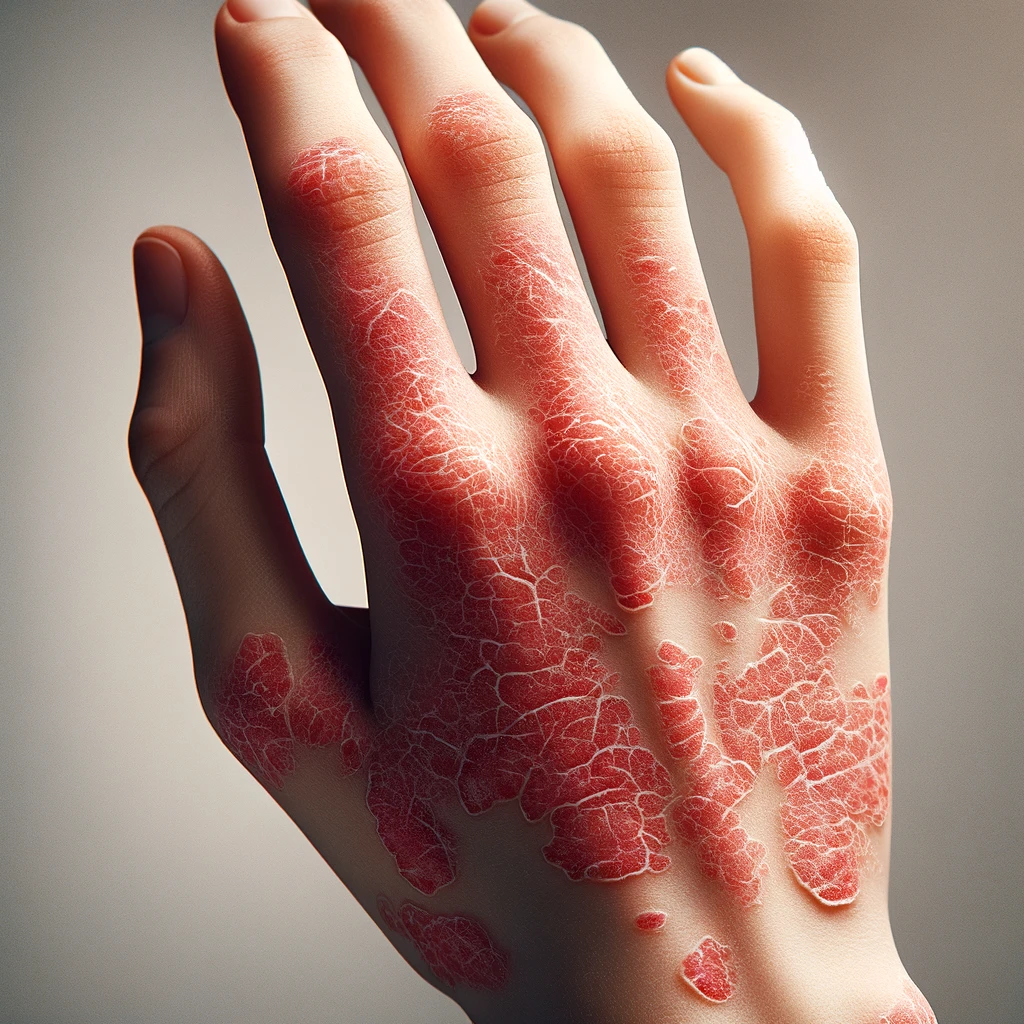

Supplement: Multimedia Appendix 1 [file ai_v3i1e58275_app1.zip › 22 custom GPT.png]

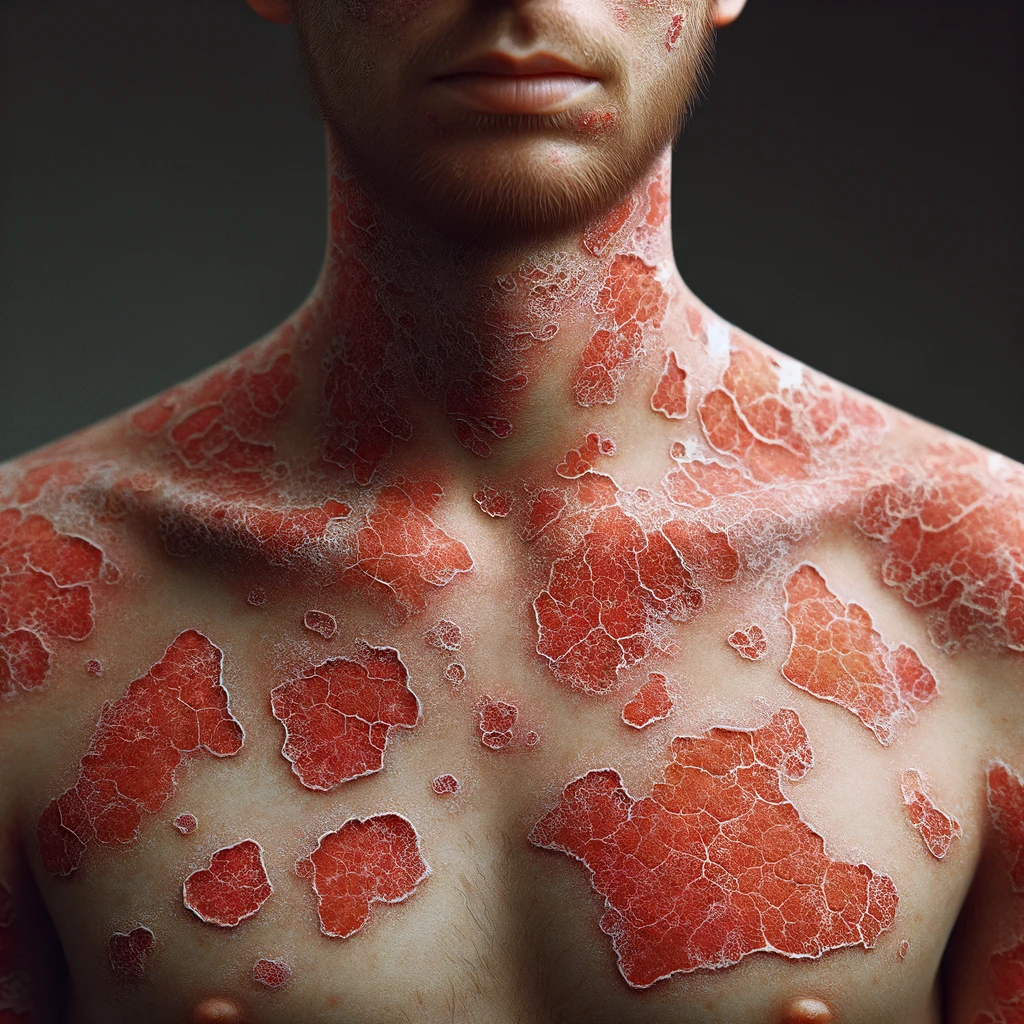

Supplement: Multimedia Appendix 1 [file ai_v3i1e58275_app1.zip › 33 custom GPT.png]

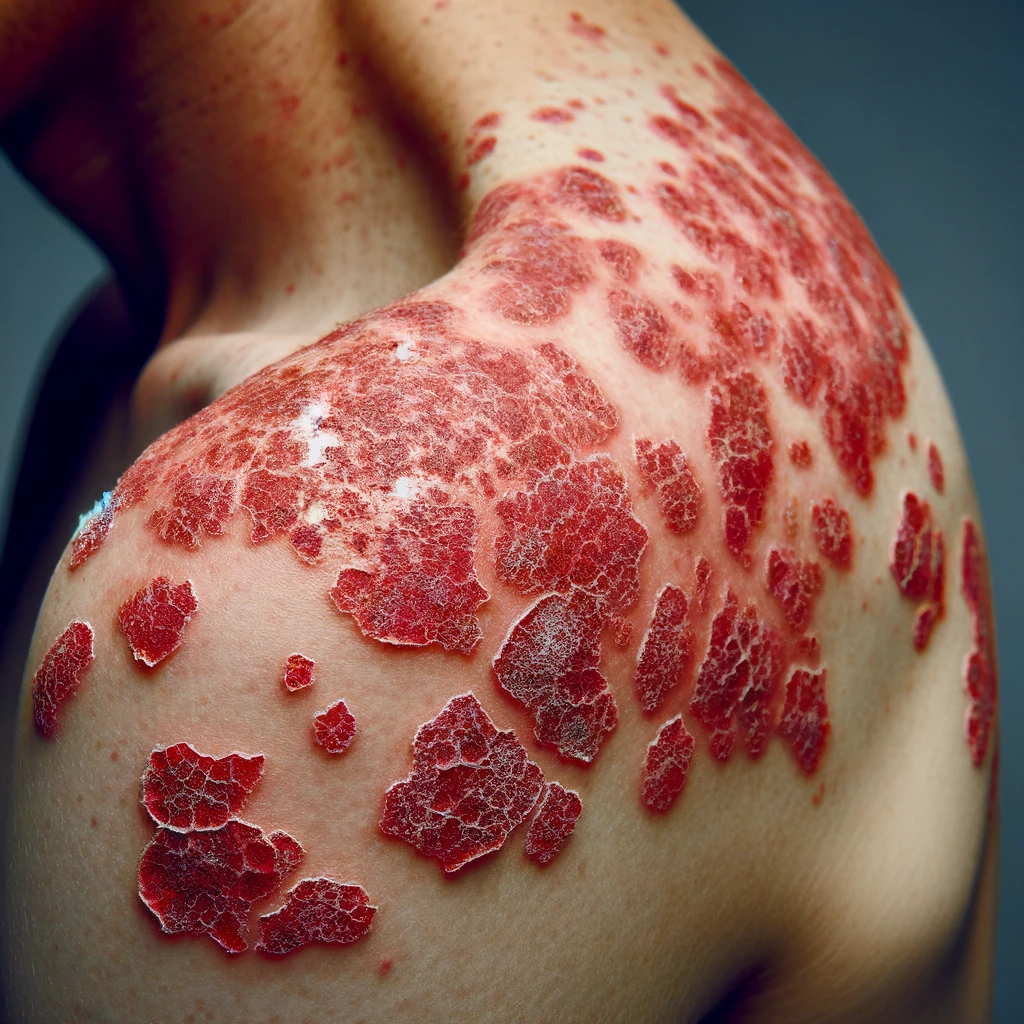

Supplement: Multimedia Appendix 1 [file ai_v3i1e58275_app1.zip › 48 custom GPT.png]

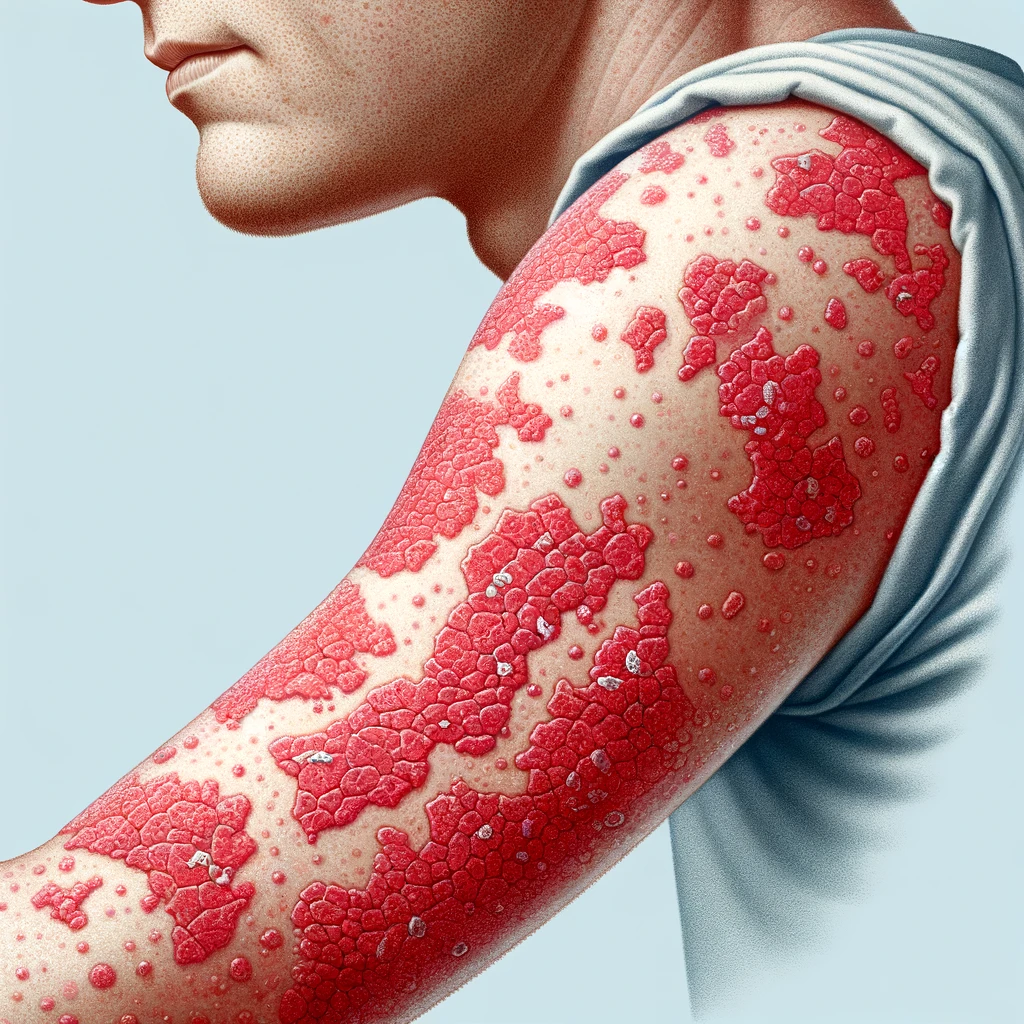

Supplement: Multimedia Appendix 1 [file ai_v3i1e58275_app1.zip › 58 custom GPT.png]

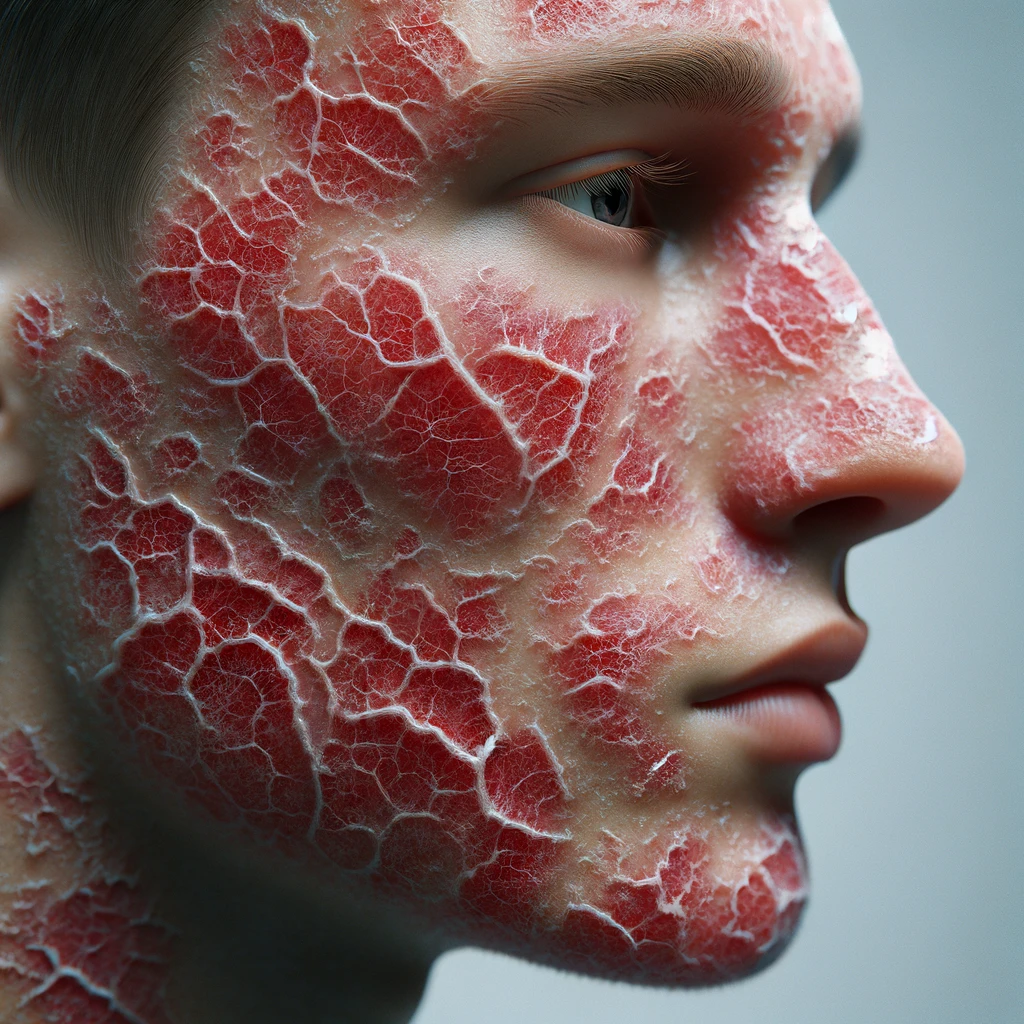

Supplement: Multimedia Appendix 1 [file ai_v3i1e58275_app1.zip › 36 custom GPT.png]

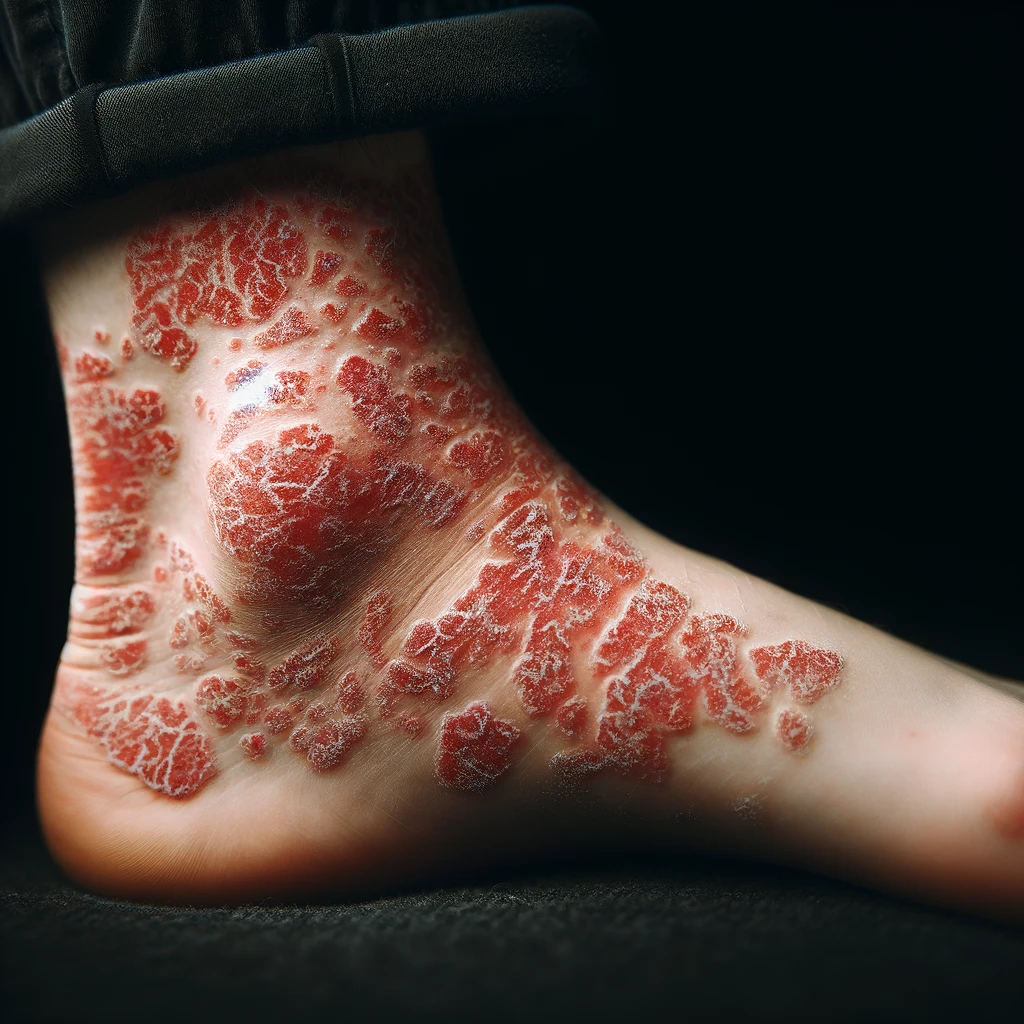

Supplement: Multimedia Appendix 1 [file ai_v3i1e58275_app1.zip › 02 custom GPT.png]

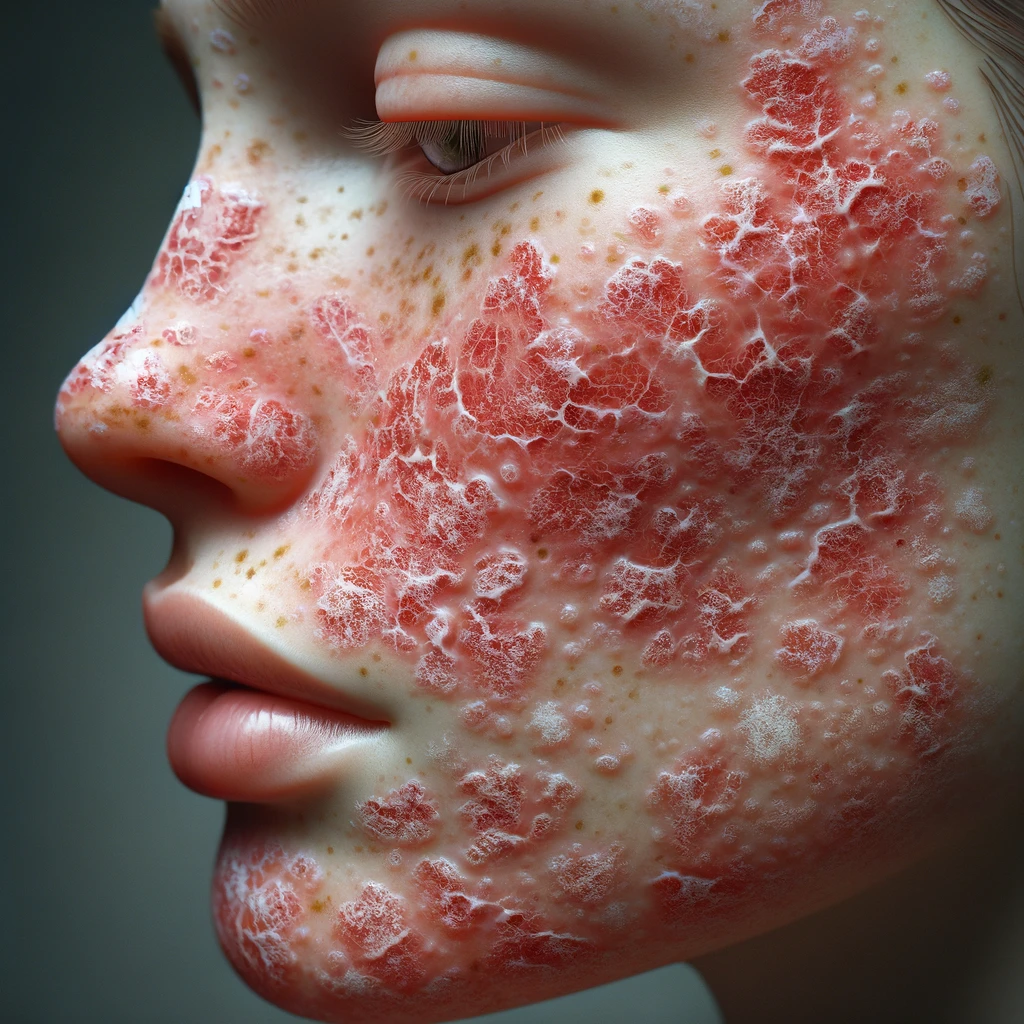

Supplement: Multimedia Appendix 1 [file ai_v3i1e58275_app1.zip › 87 custom GPT.png]

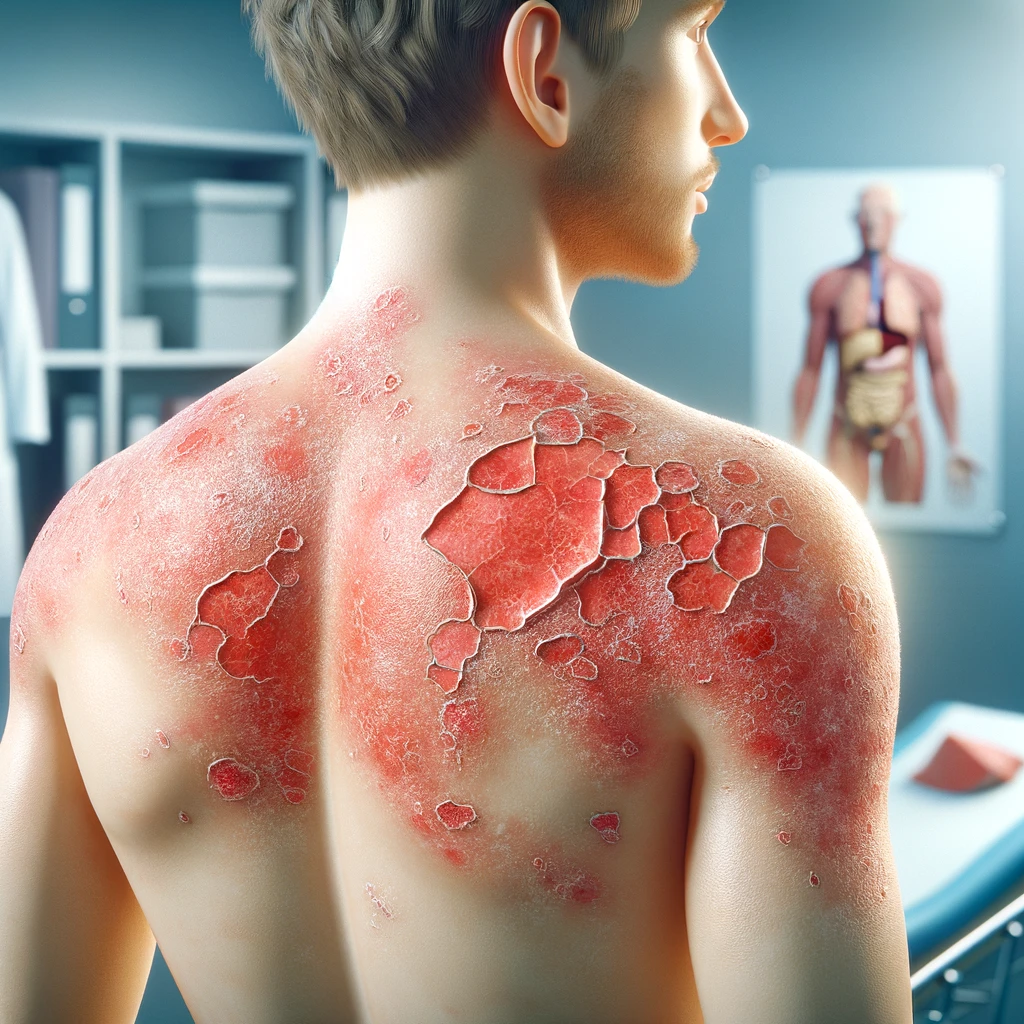

Supplement: Multimedia Appendix 1 [file ai_v3i1e58275_app1.zip › 49 custom GPT.png]

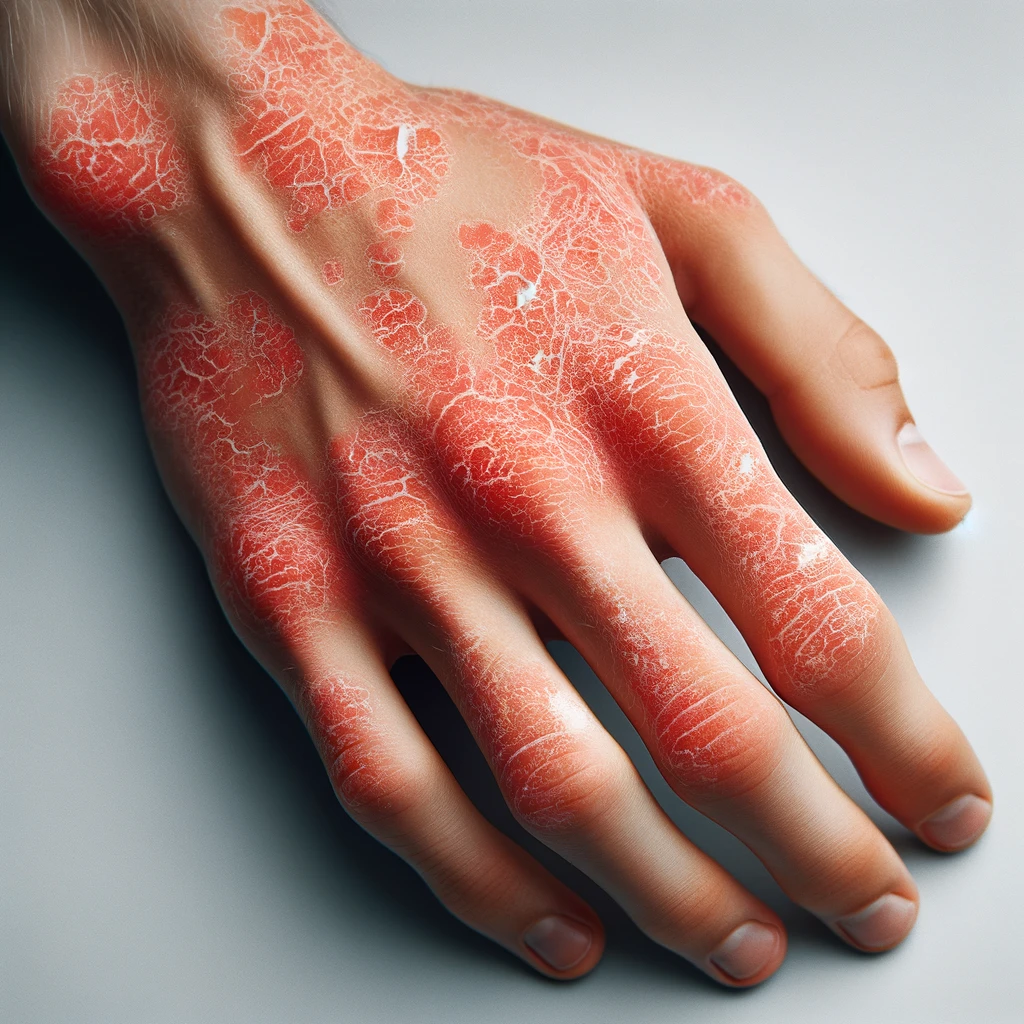

Supplement: Multimedia Appendix 1 [file ai_v3i1e58275_app1.zip › 45 custom GPT.png]

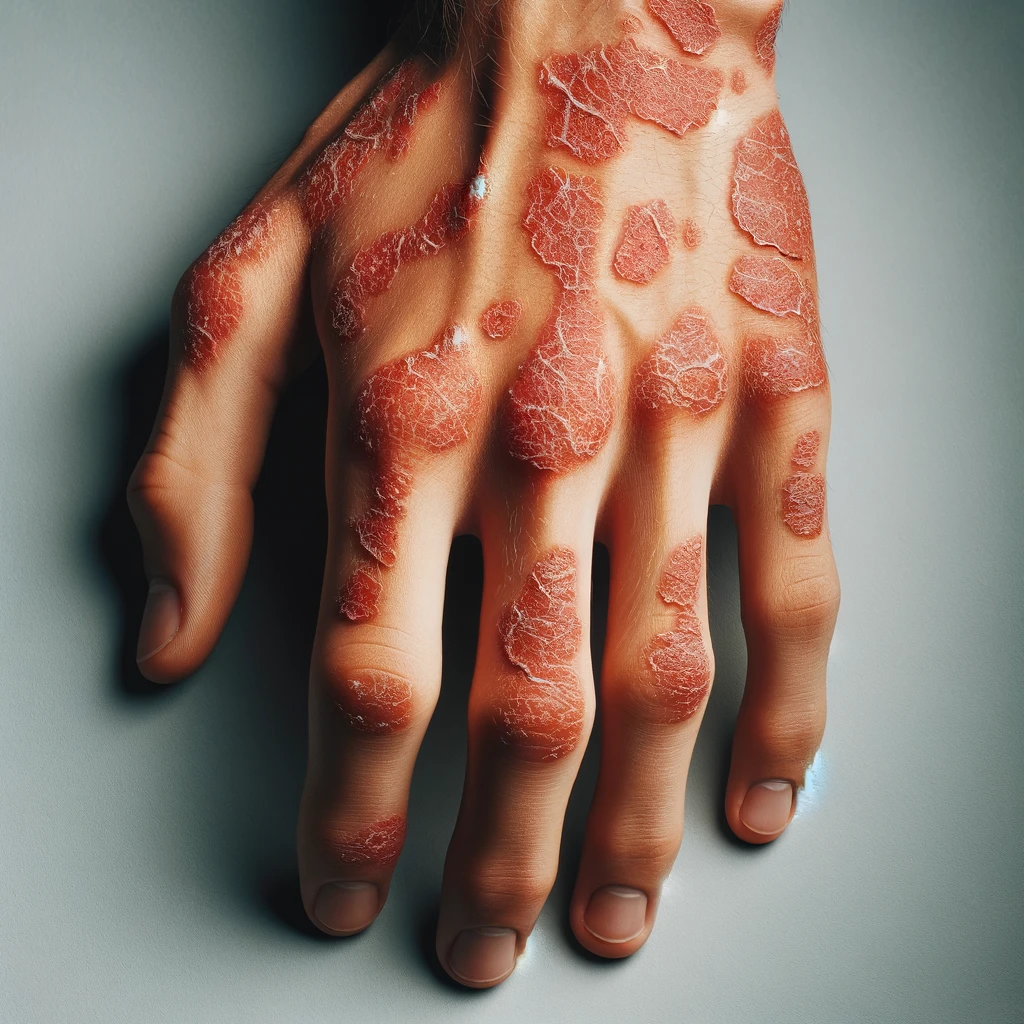

Supplement: Multimedia Appendix 1 [file ai_v3i1e58275_app1.zip › 51 custom GPT.png]

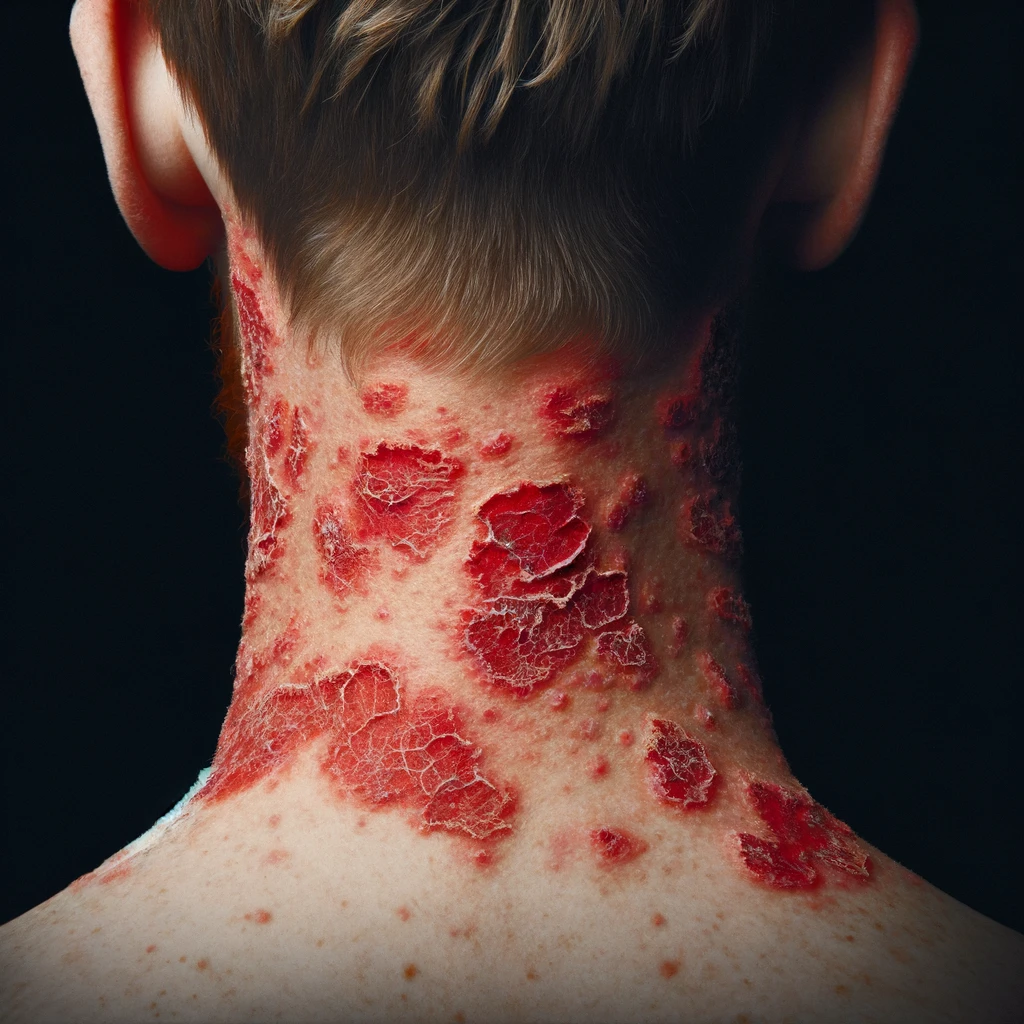

Supplement: Multimedia Appendix 1 [file ai_v3i1e58275_app1.zip › 17 custom GPT.png]

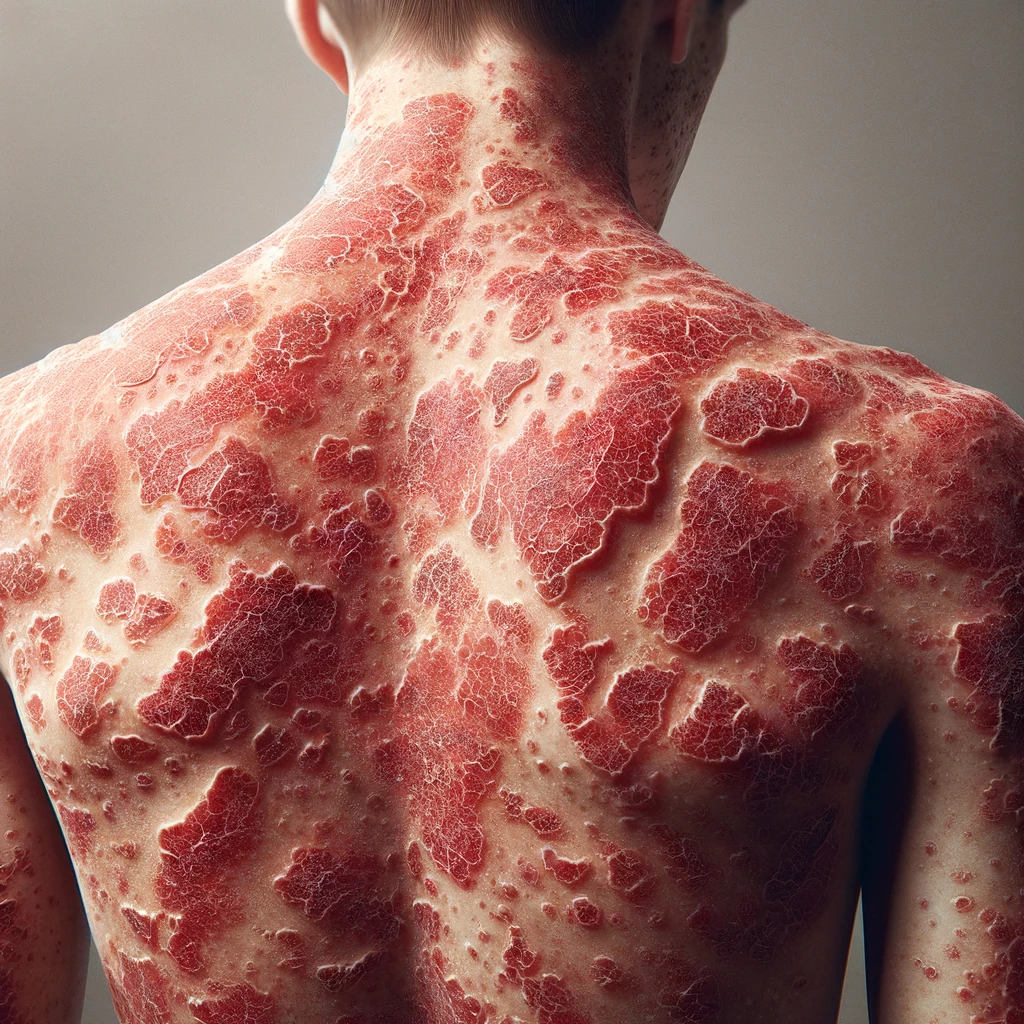

Supplement: Multimedia Appendix 1 [file ai_v3i1e58275_app1.zip › 57 custom GPT.png]

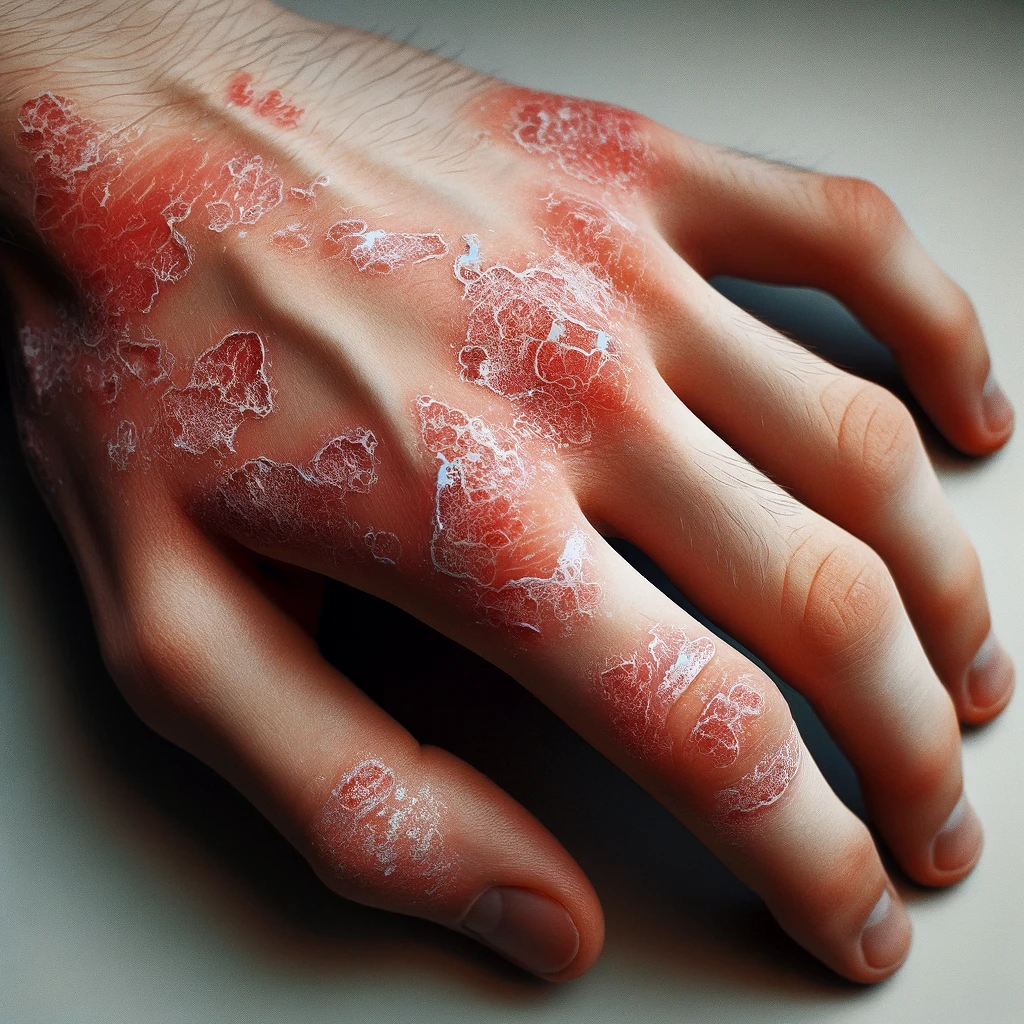

Supplement: Multimedia Appendix 1 [file ai_v3i1e58275_app1.zip › 03 custom GPT.png]

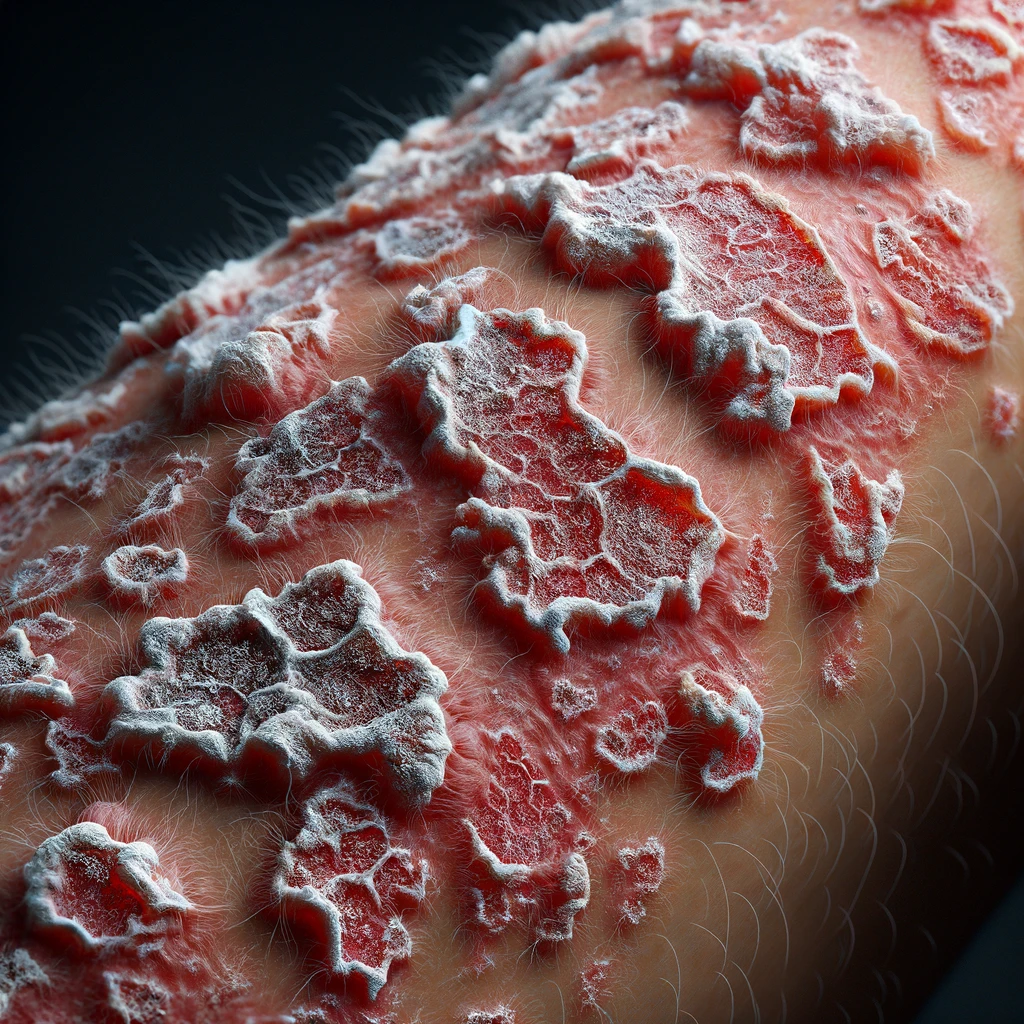

Supplement: Multimedia Appendix 1 [file ai_v3i1e58275_app1.zip › 64 custom GPT.png]

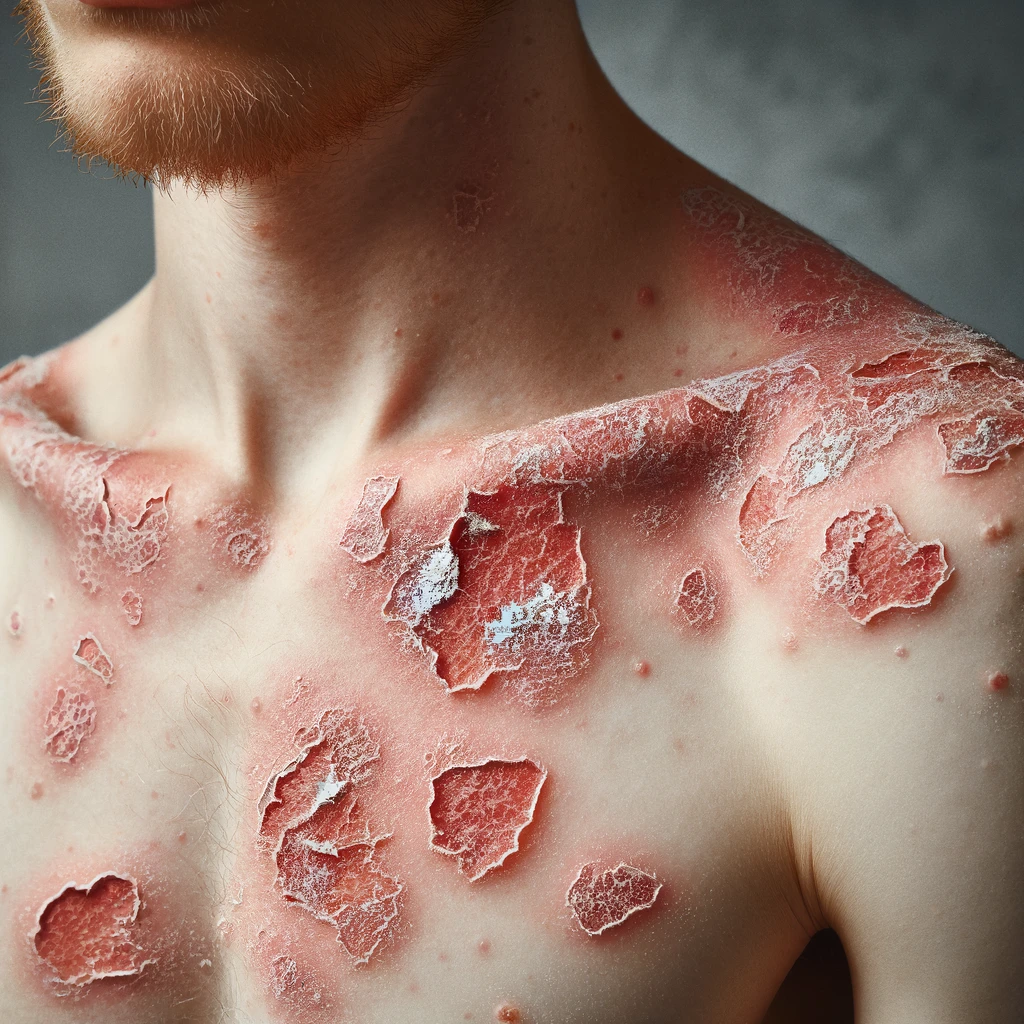

Supplement: Multimedia Appendix 1 [file ai_v3i1e58275_app1.zip › 35 custom GPT.png]

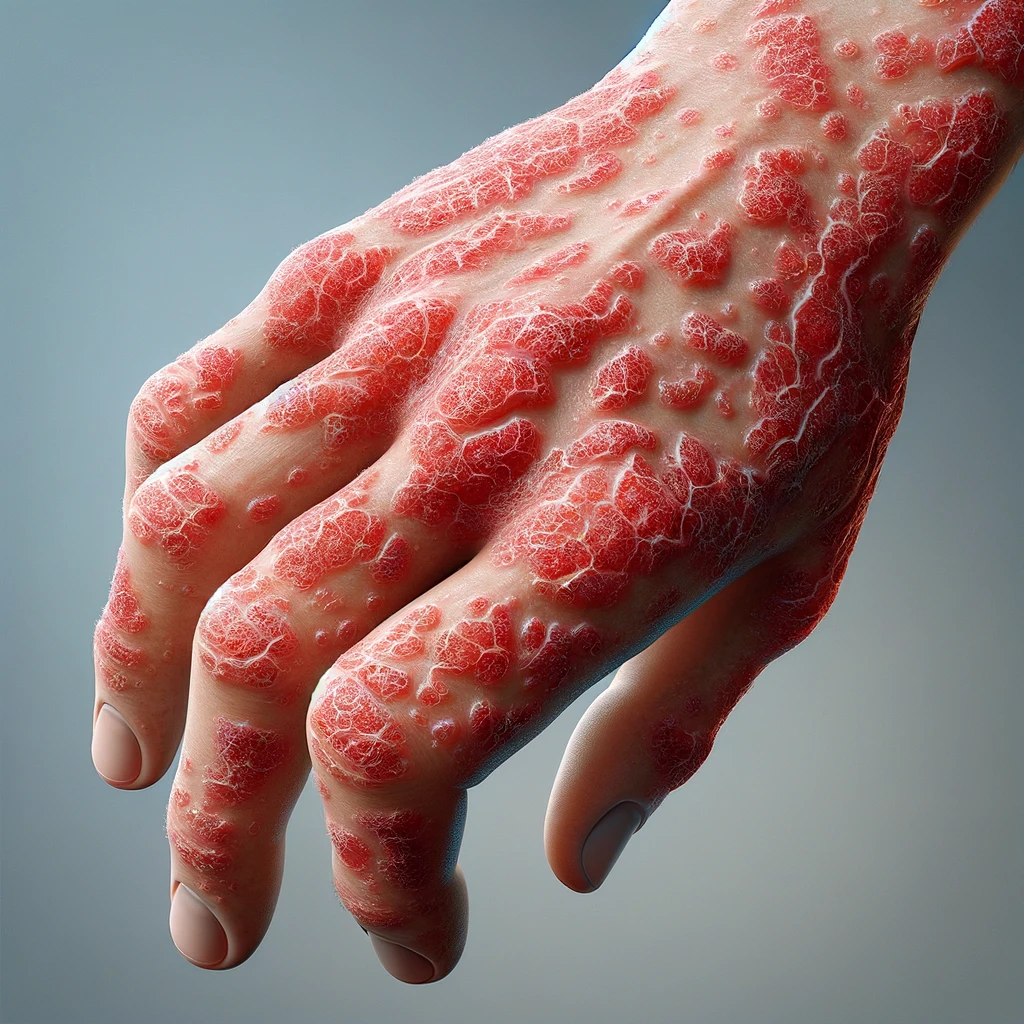

Supplement: Multimedia Appendix 1 [file ai_v3i1e58275_app1.zip › 01 custom GPT.png]

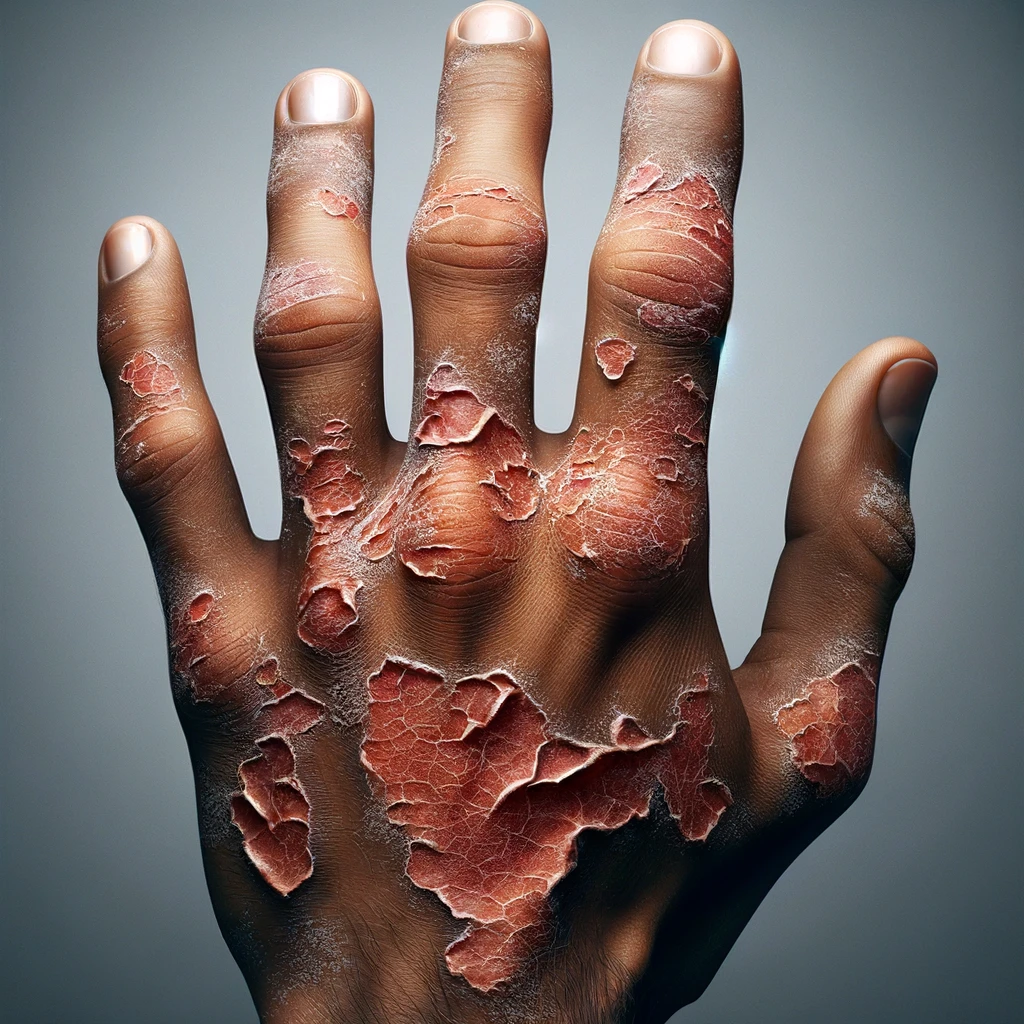

Supplement: Multimedia Appendix 1 [file ai_v3i1e58275_app1.zip › 76 custom GPT.png]

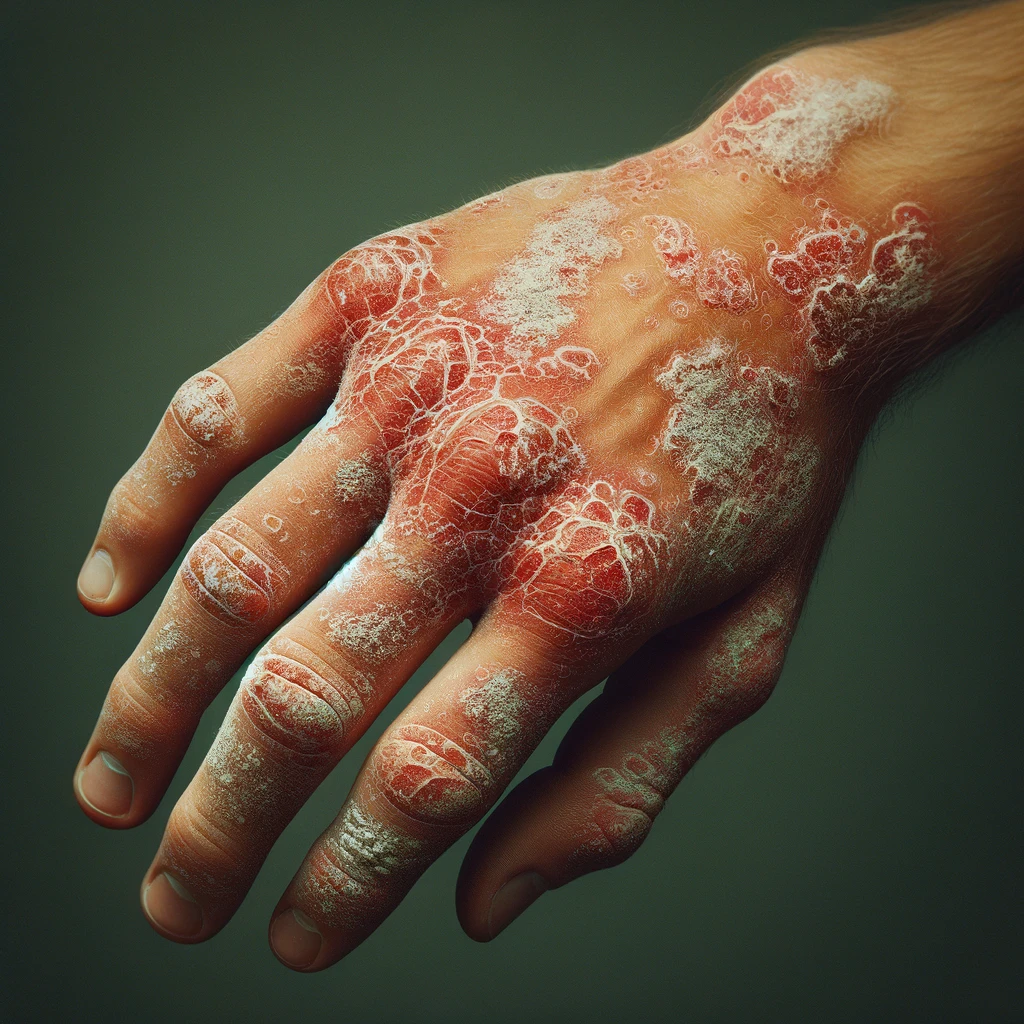

Supplement: Multimedia Appendix 1 [file ai_v3i1e58275_app1.zip › 73 custom GPT.png]

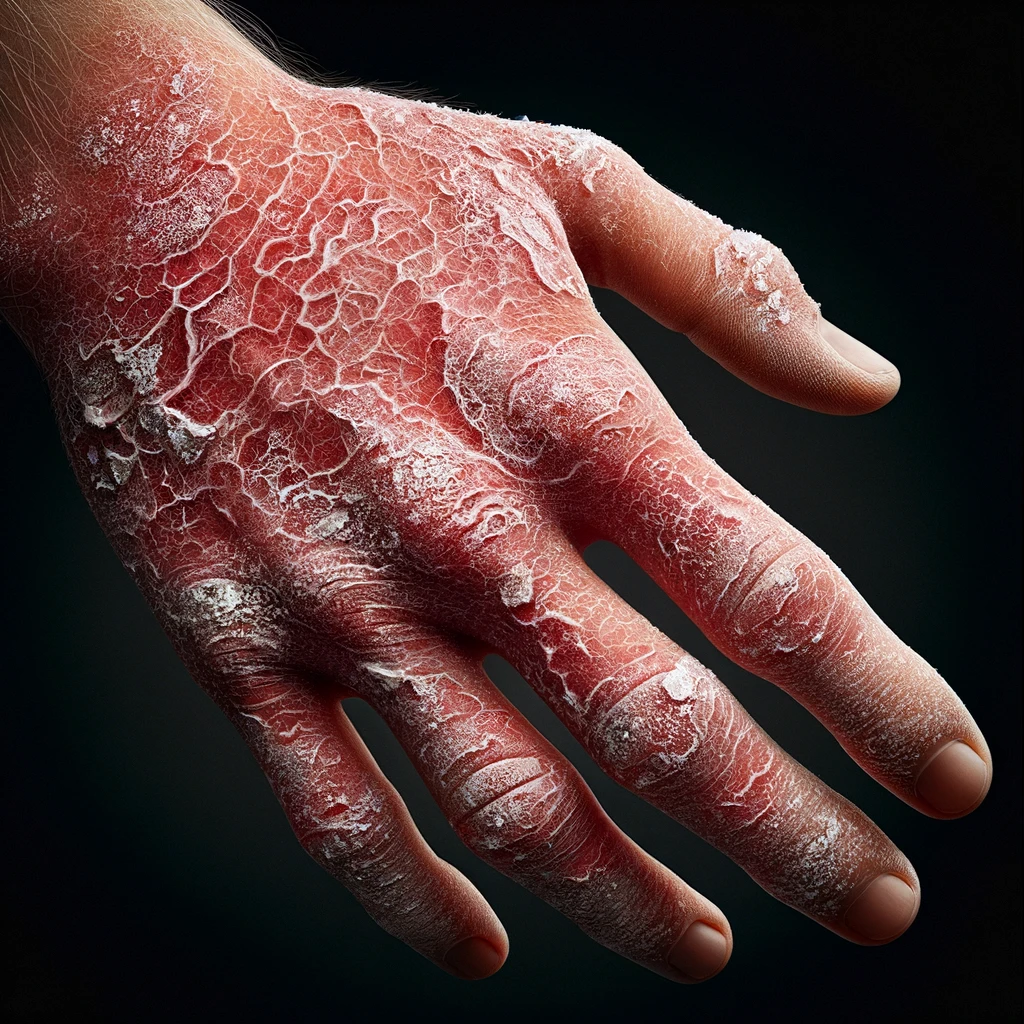

Supplement: Multimedia Appendix 1 [file ai_v3i1e58275_app1.zip › 78 custom GPT.png]

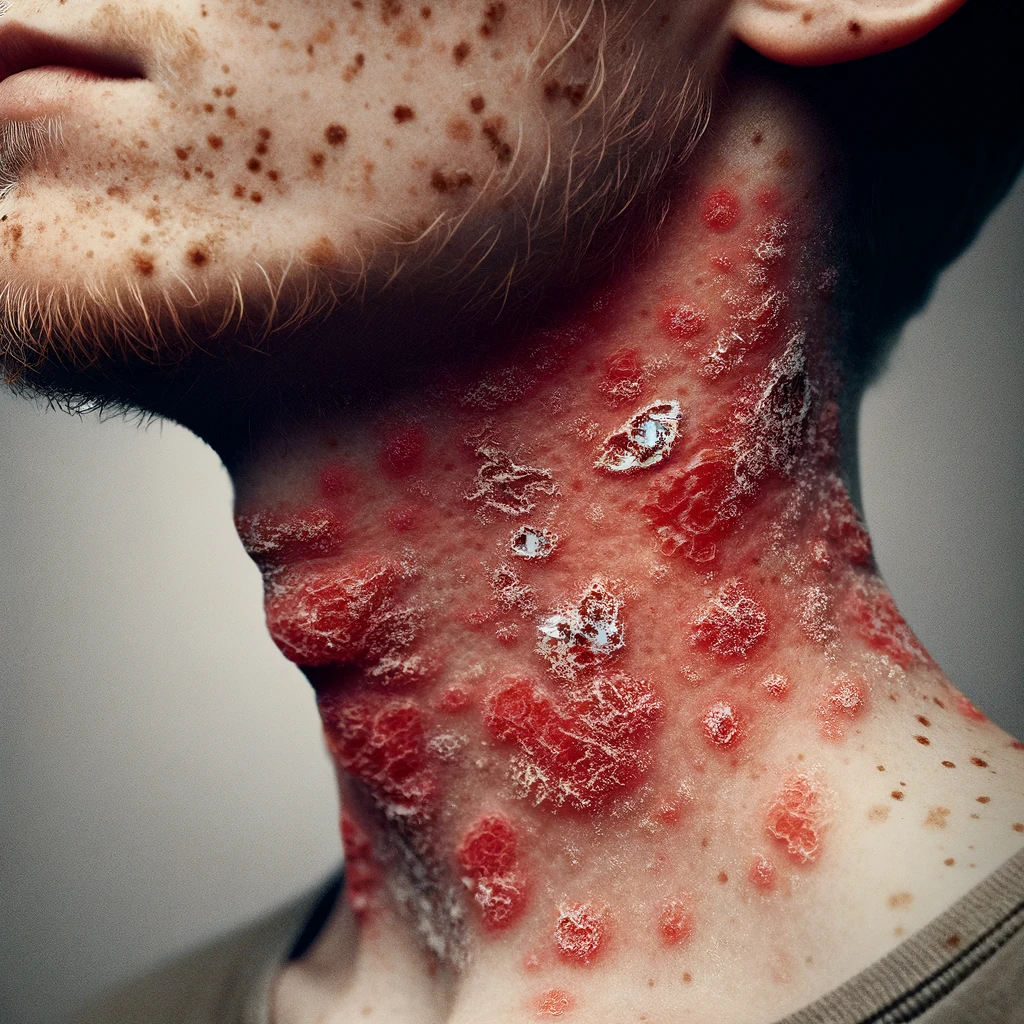

Supplement: Multimedia Appendix 1 [file ai_v3i1e58275_app1.zip › 95 custom GPT.png]

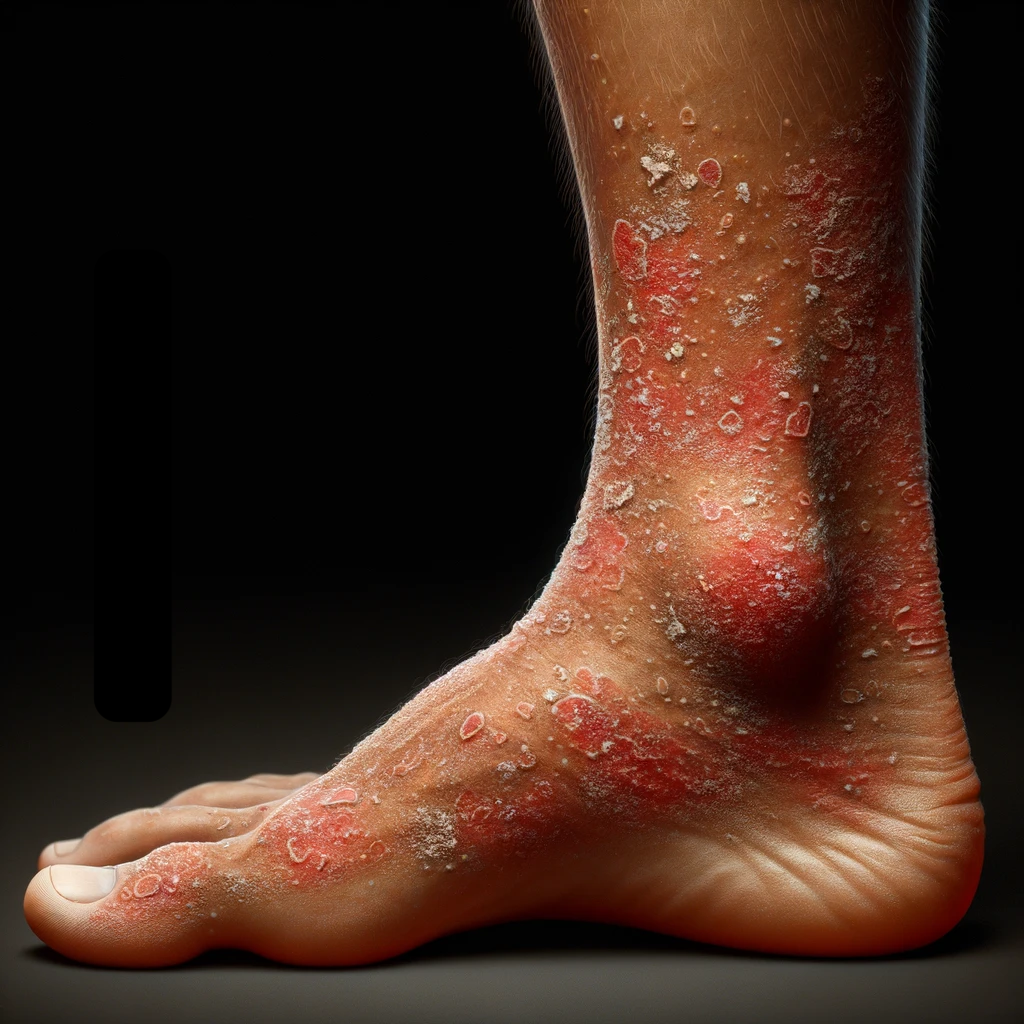

Supplement: Multimedia Appendix 1 [file ai_v3i1e58275_app1.zip › 98 custom GPT.png]

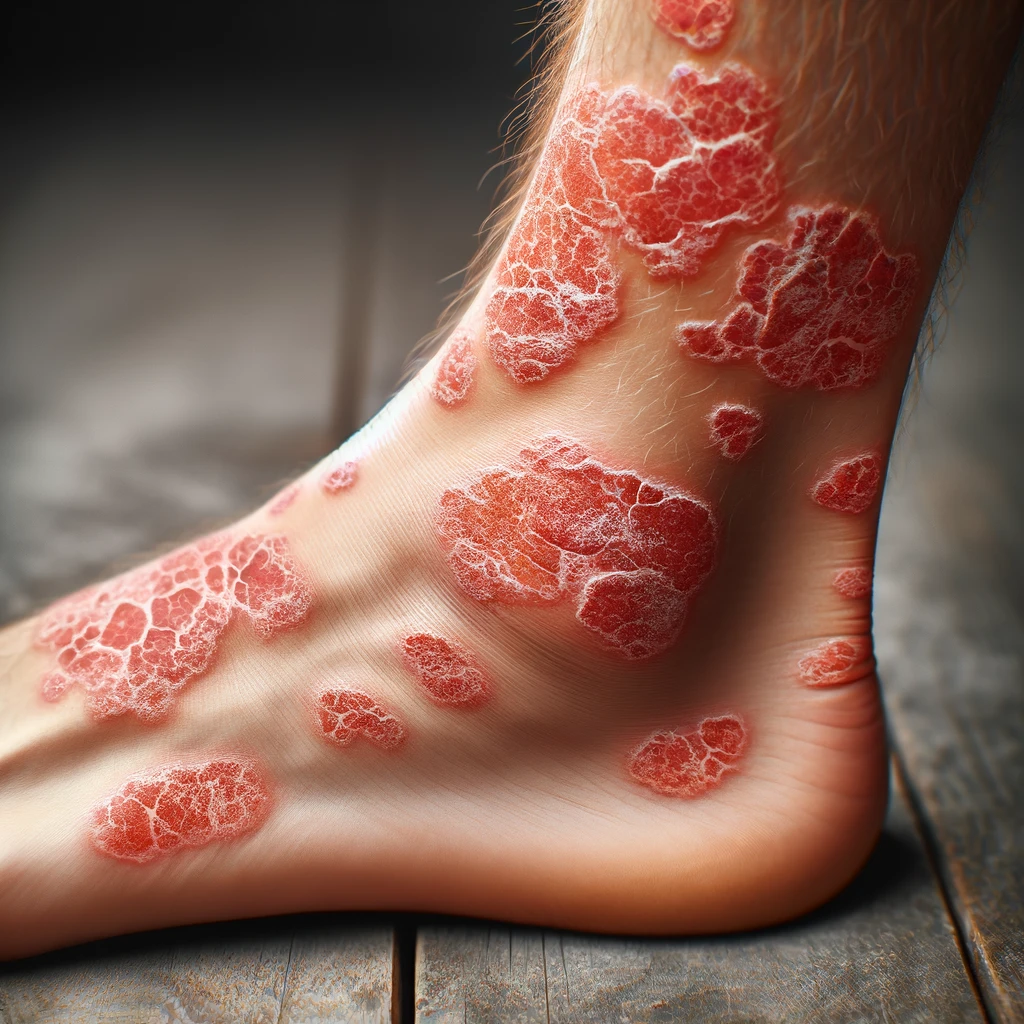

Supplement: Multimedia Appendix 1 [file ai_v3i1e58275_app1.zip › 09 custom GPT.png]

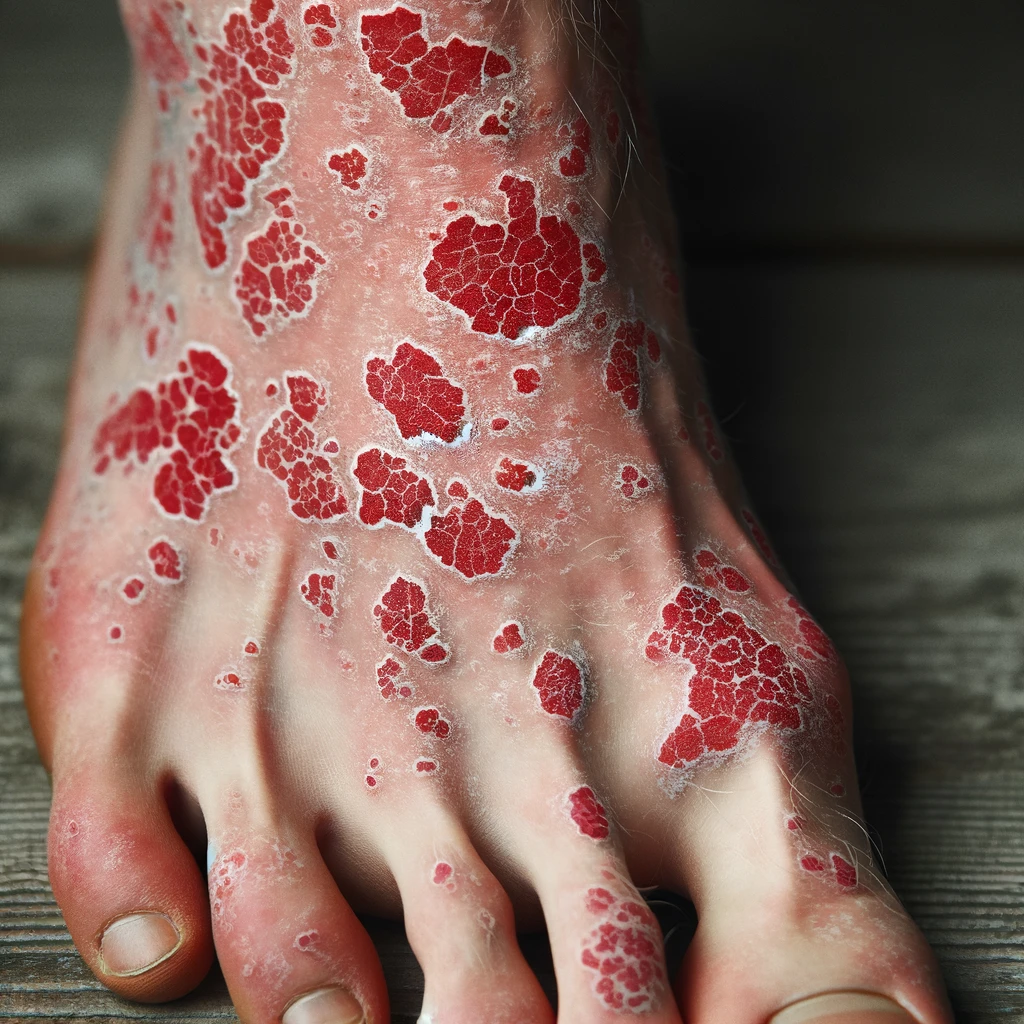

Supplement: Multimedia Appendix 1 [file ai_v3i1e58275_app1.zip › 69 custom GPT.png]

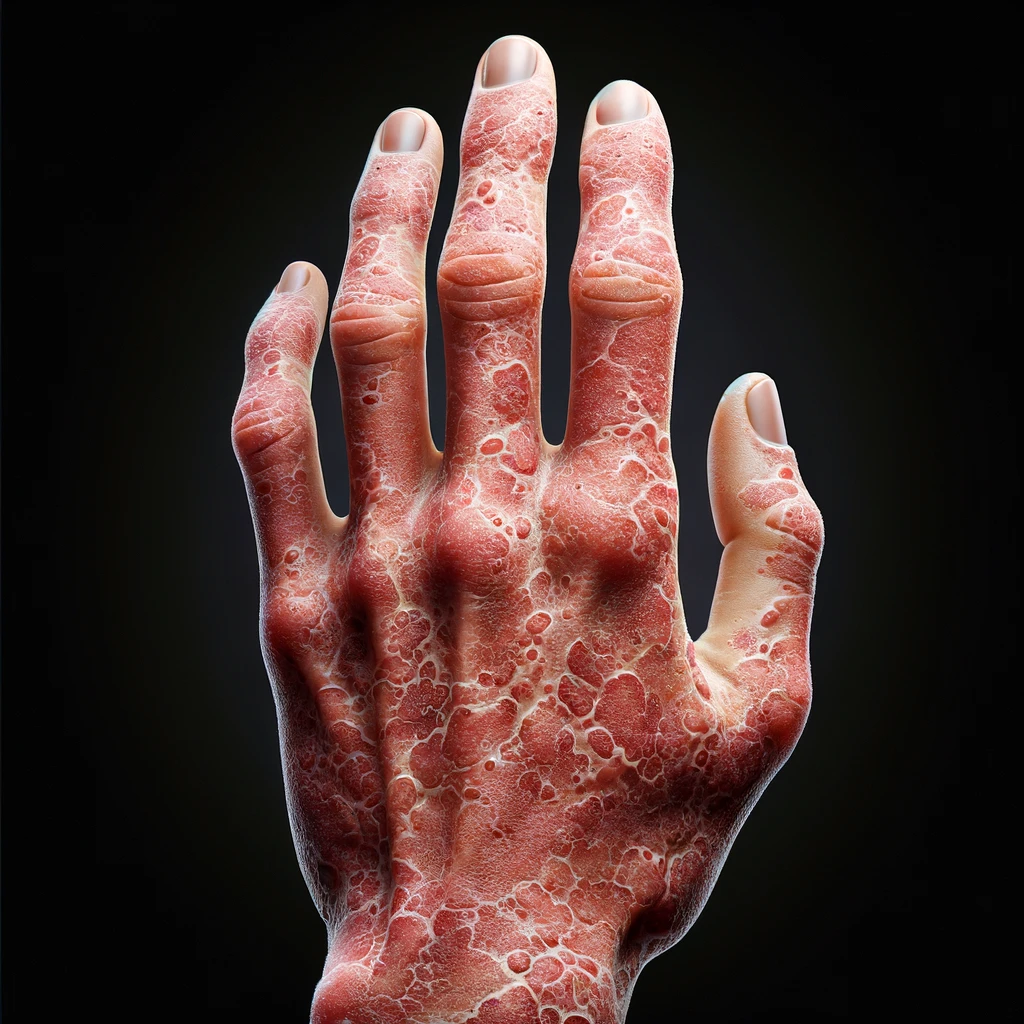

Supplement: Multimedia Appendix 1 [file ai_v3i1e58275_app1.zip › 63 custom GPT.png]

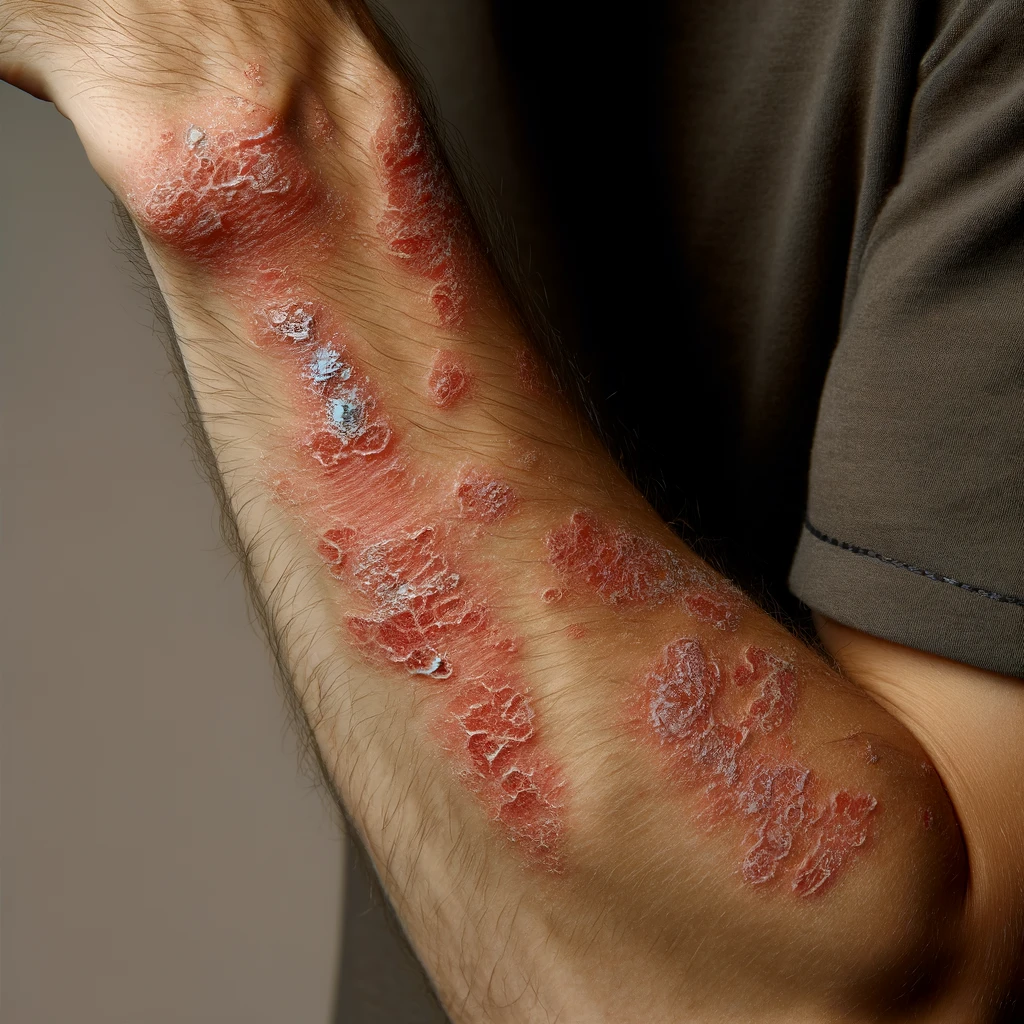

Supplement: Multimedia Appendix 1 [file ai_v3i1e58275_app1.zip › 12 custom GPT.png]

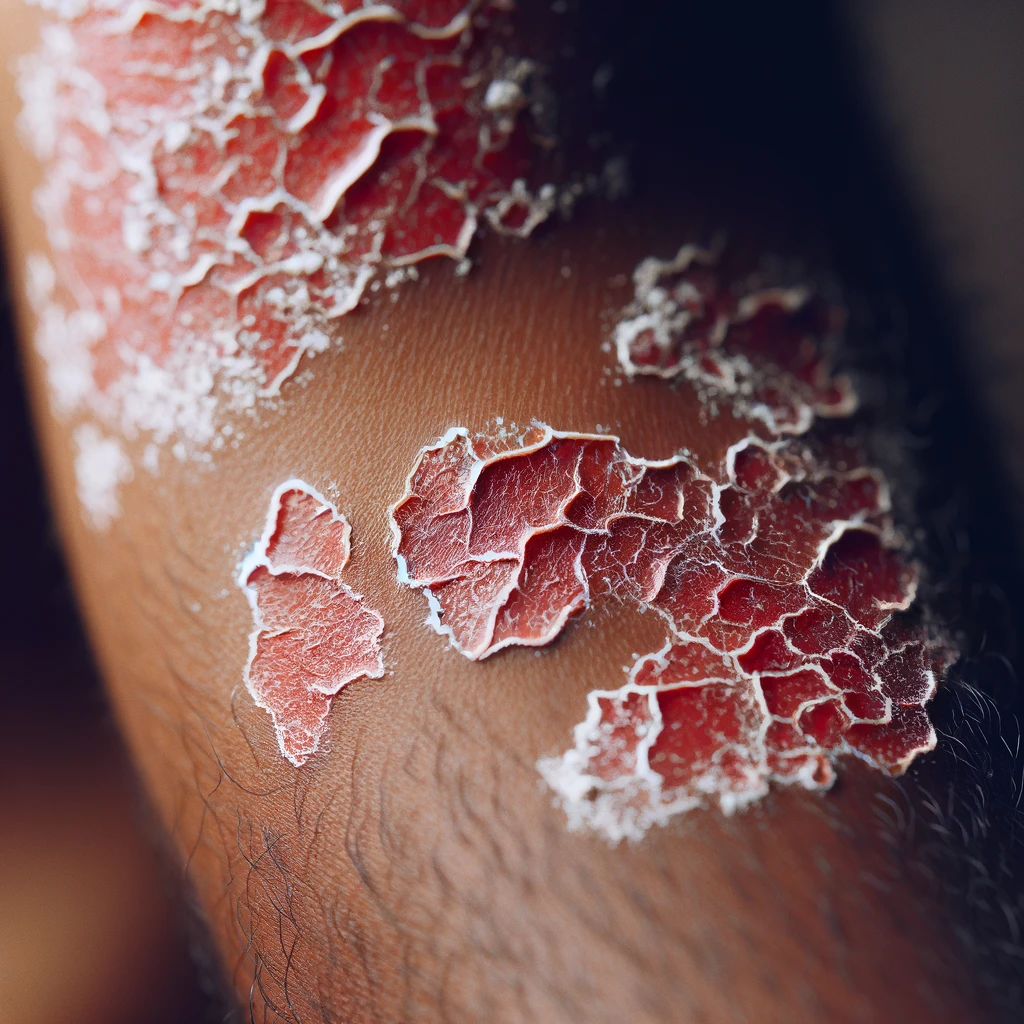

Supplement: Multimedia Appendix 1 [file ai_v3i1e58275_app1.zip › 59 custom GPT.png]

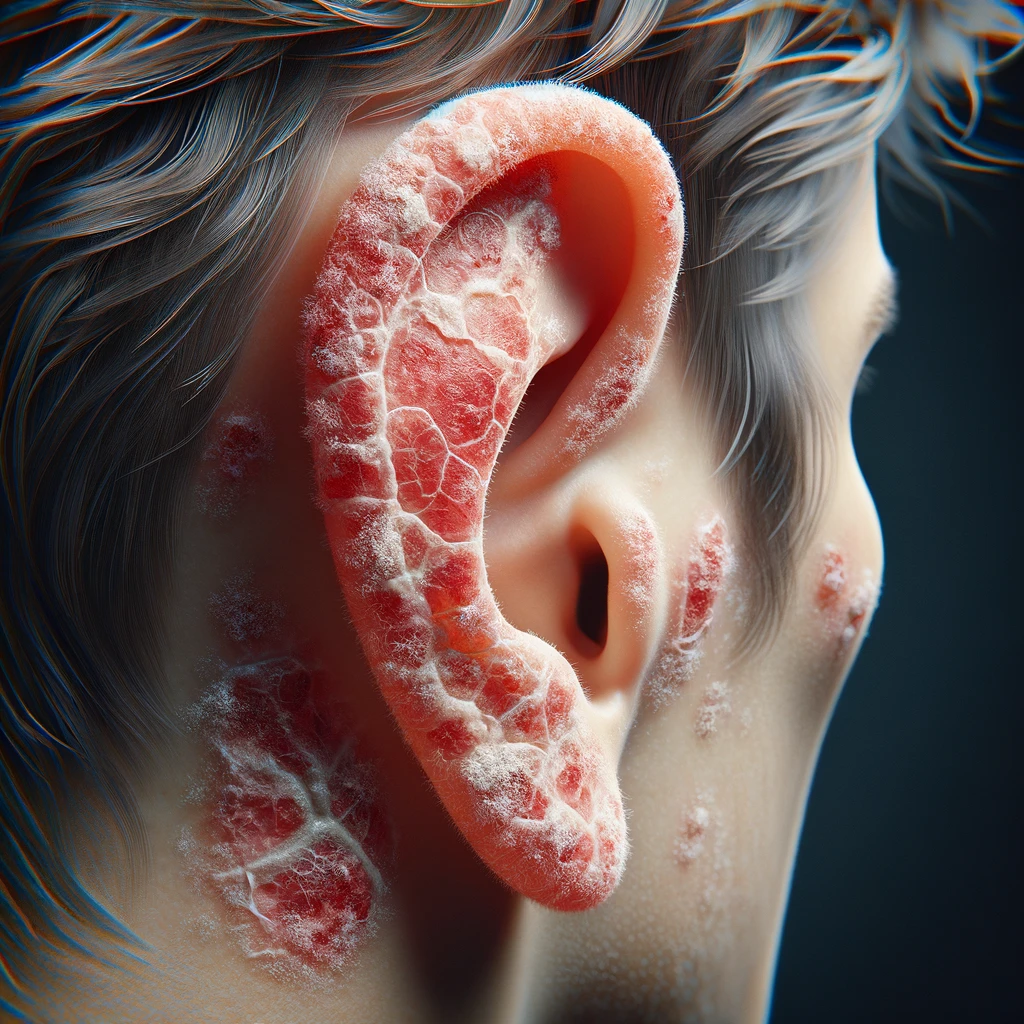

Supplement: Multimedia Appendix 1 [file ai_v3i1e58275_app1.zip › 93 custom GPT.png]

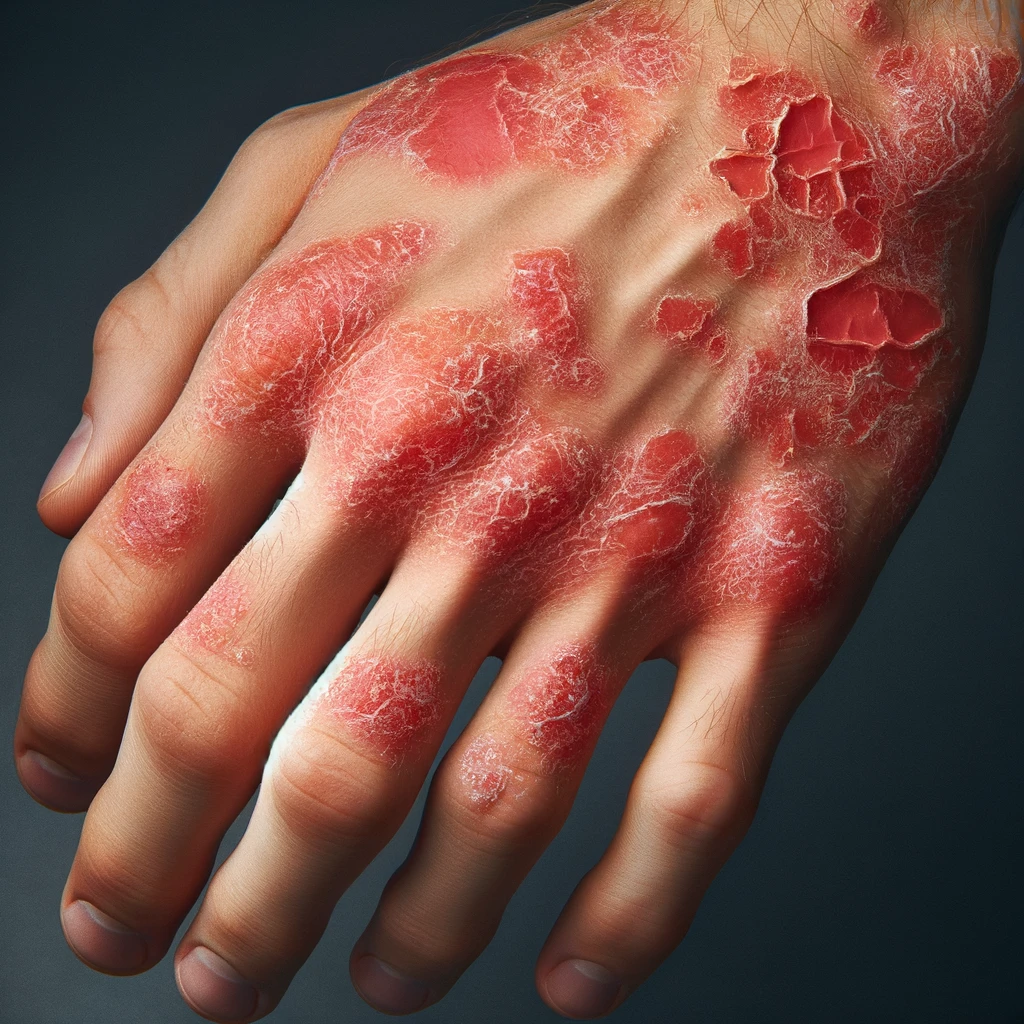

Supplement: Multimedia Appendix 1 [file ai_v3i1e58275_app1.zip › 80 custom GPT.png]

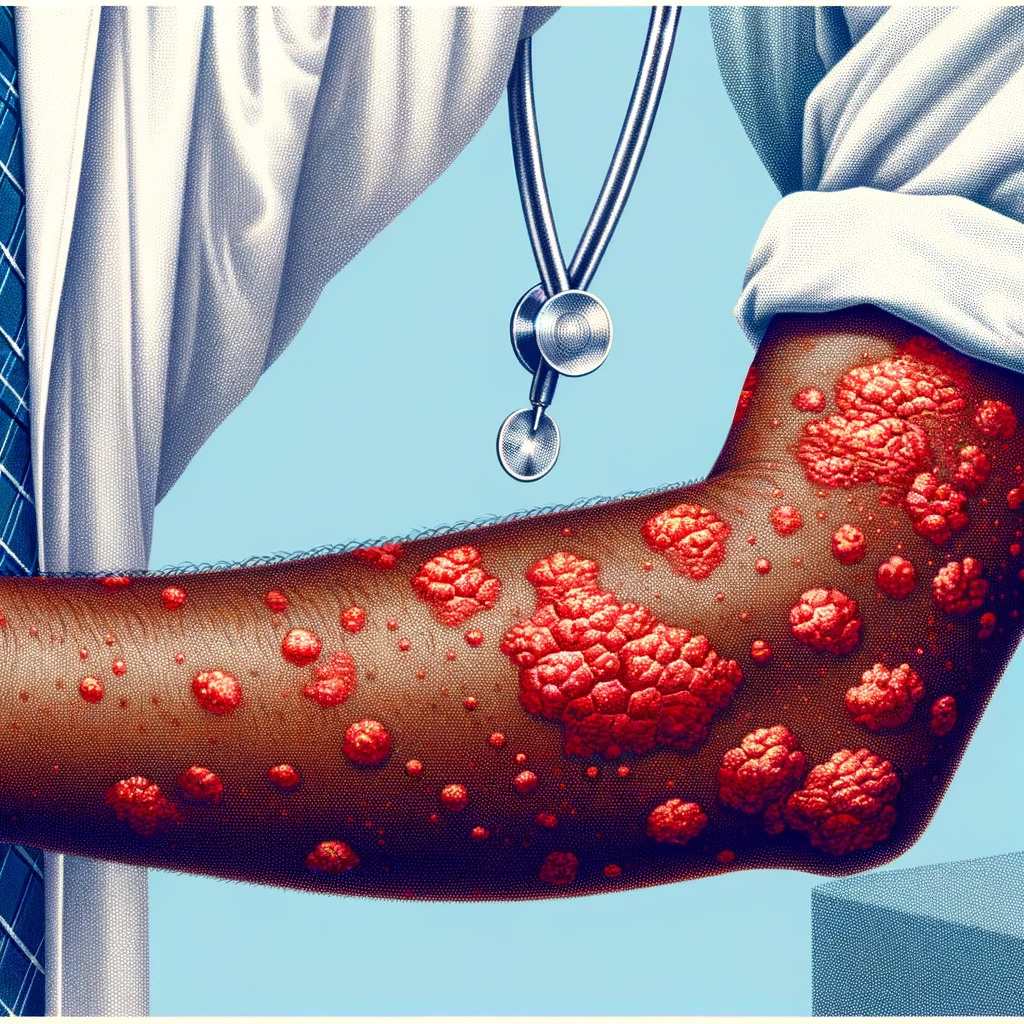

Supplement: Multimedia Appendix 1 [file ai_v3i1e58275_app1.zip › 54 custom GPT.png]

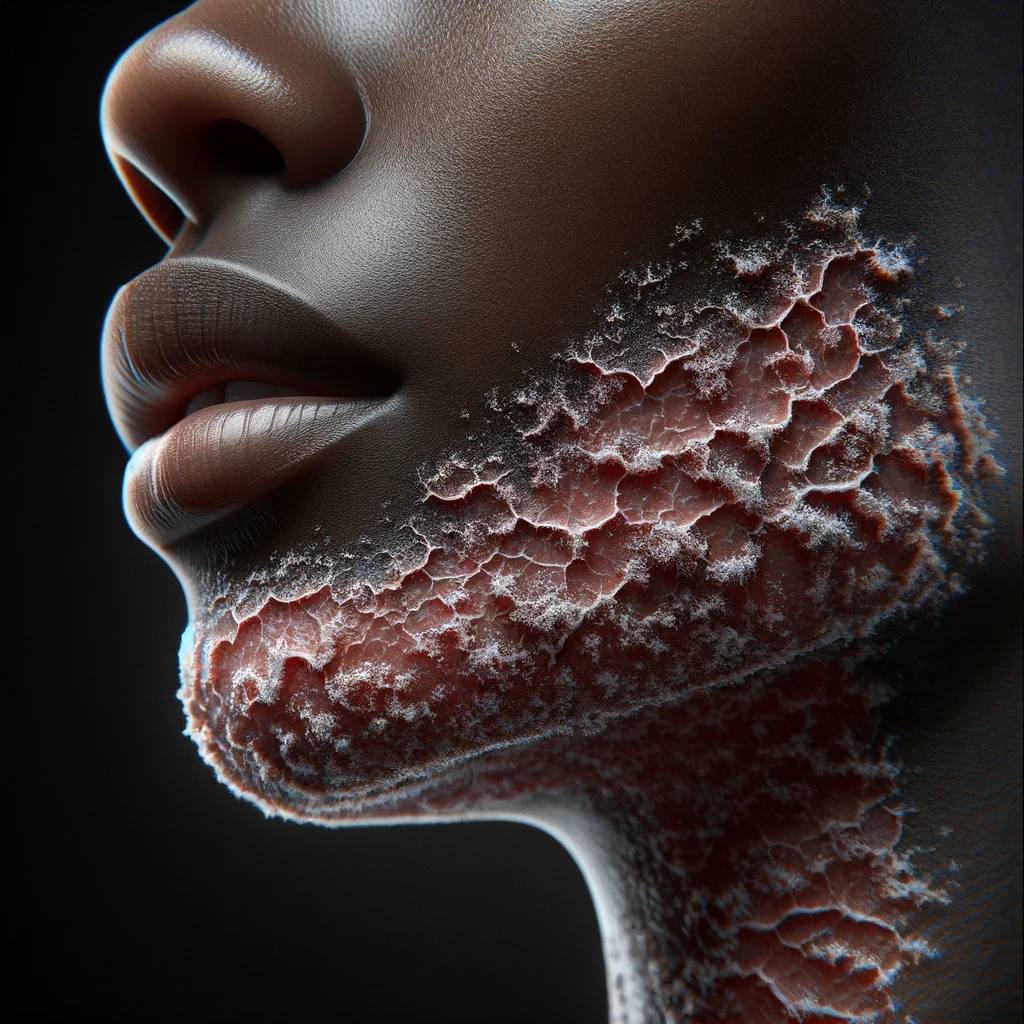

Supplement: Multimedia Appendix 1 [file ai_v3i1e58275_app1.zip › 96 custom GPT.png]

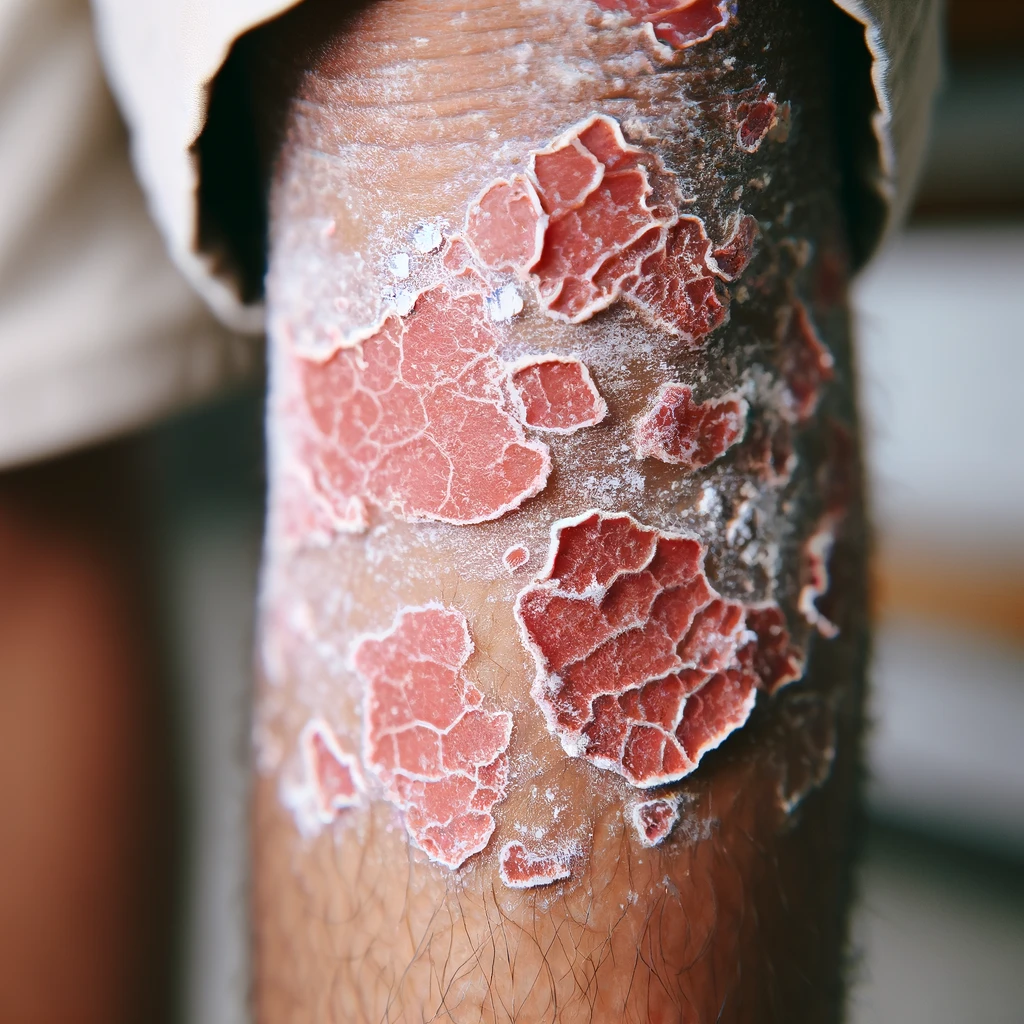

Supplement: Multimedia Appendix 1 [file ai_v3i1e58275_app1.zip › 52 custom GPT.png]

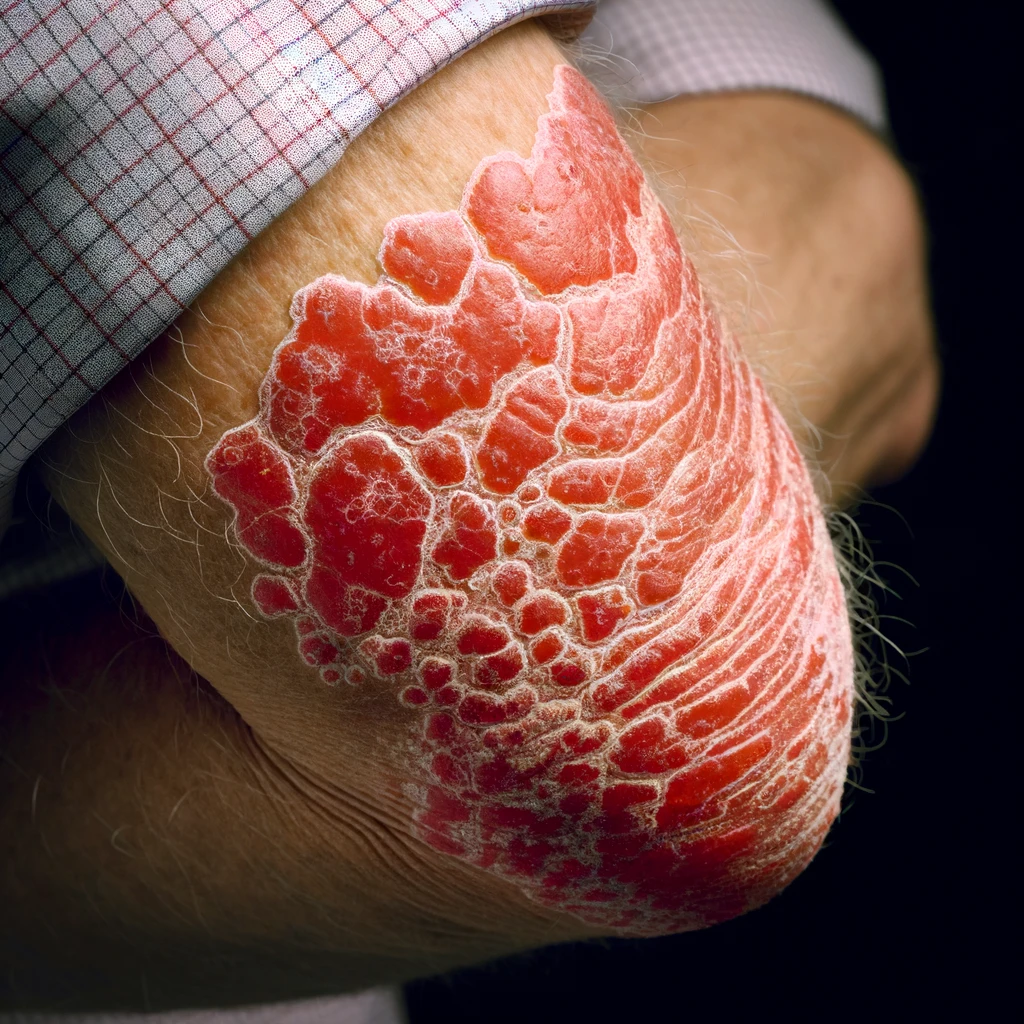

Supplement: Multimedia Appendix 1 [file ai_v3i1e58275_app1.zip › 81 custom GPT.png]

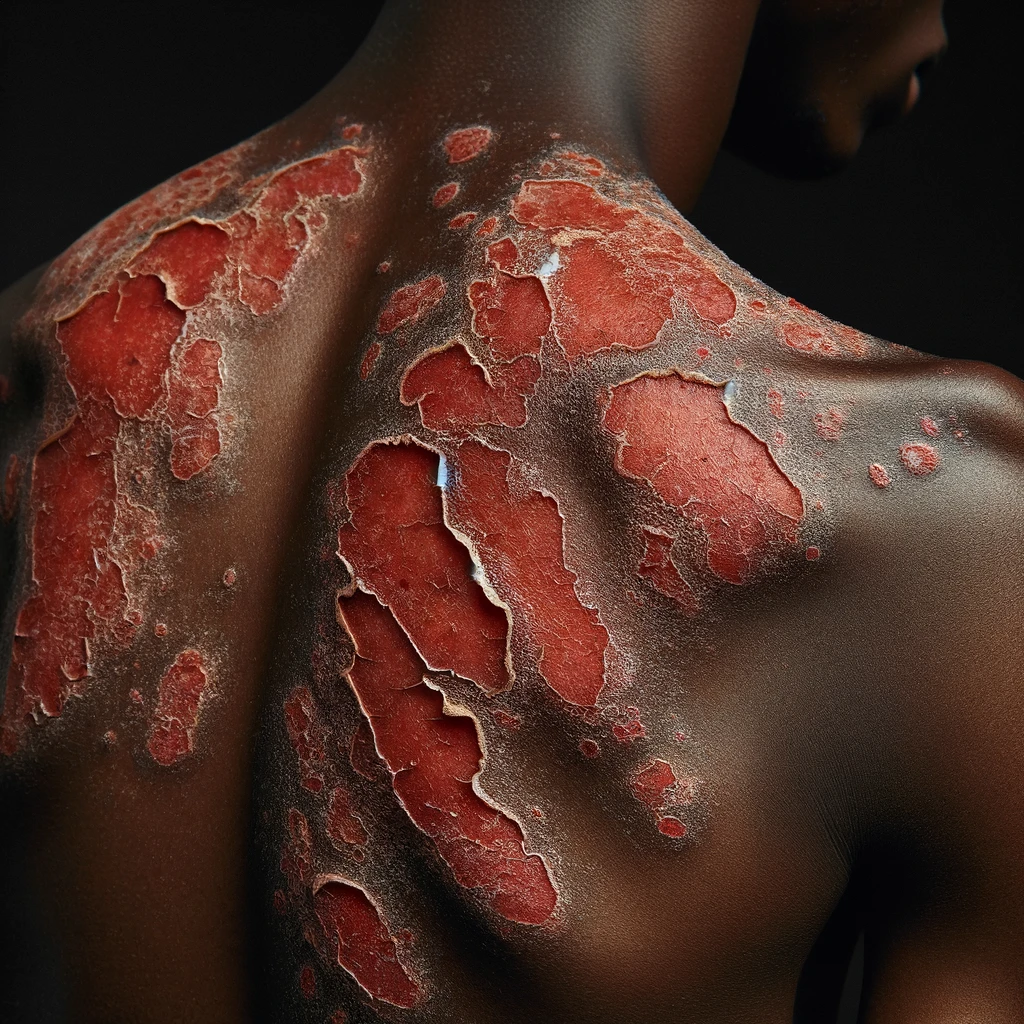

Supplement: Multimedia Appendix 1 [file ai_v3i1e58275_app1.zip › 26 custom GPT.png]

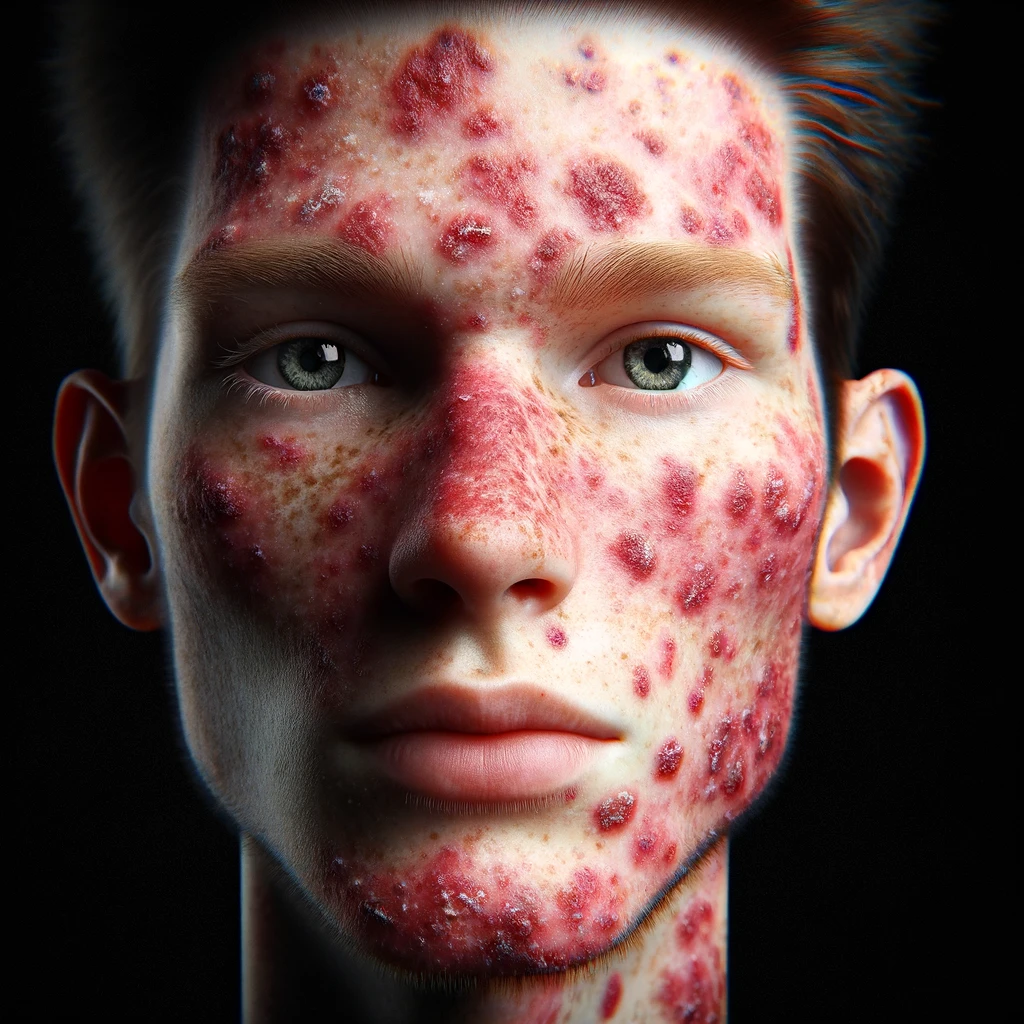

Supplement: Multimedia Appendix 1 [file ai_v3i1e58275_app1.zip › 92 custom GPT.png]

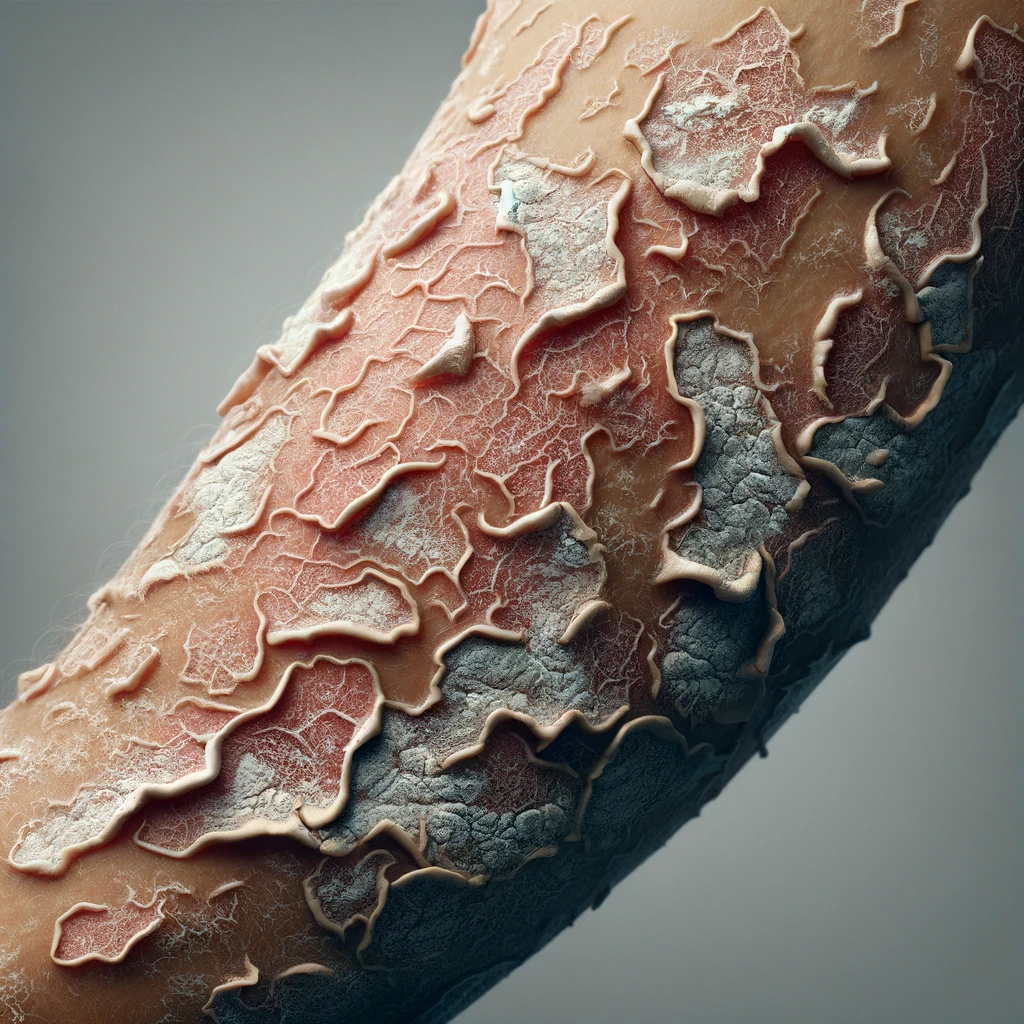

Supplement: Multimedia Appendix 1 [file ai_v3i1e58275_app1.zip › 67 custom GPT.png]

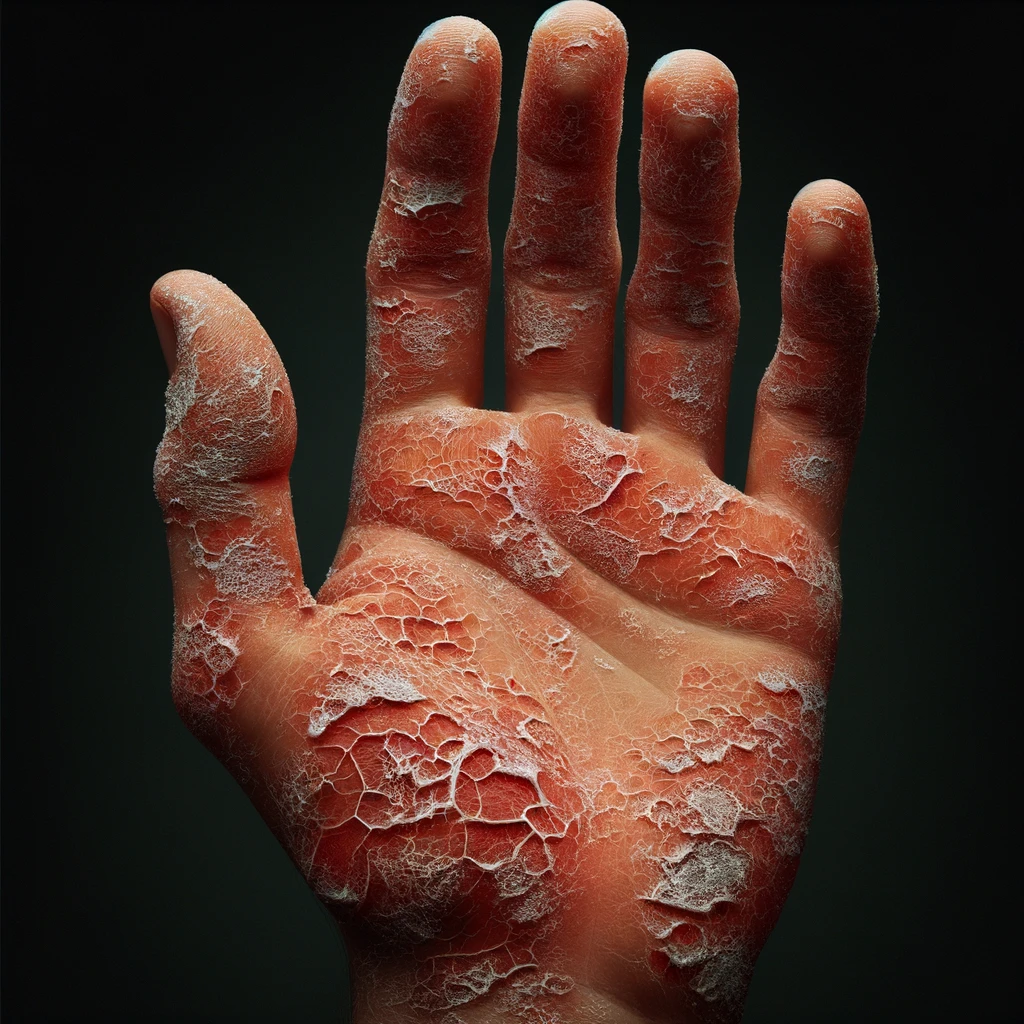

Supplement: Multimedia Appendix 1 [file ai_v3i1e58275_app1.zip › 18 custom GPT.png]

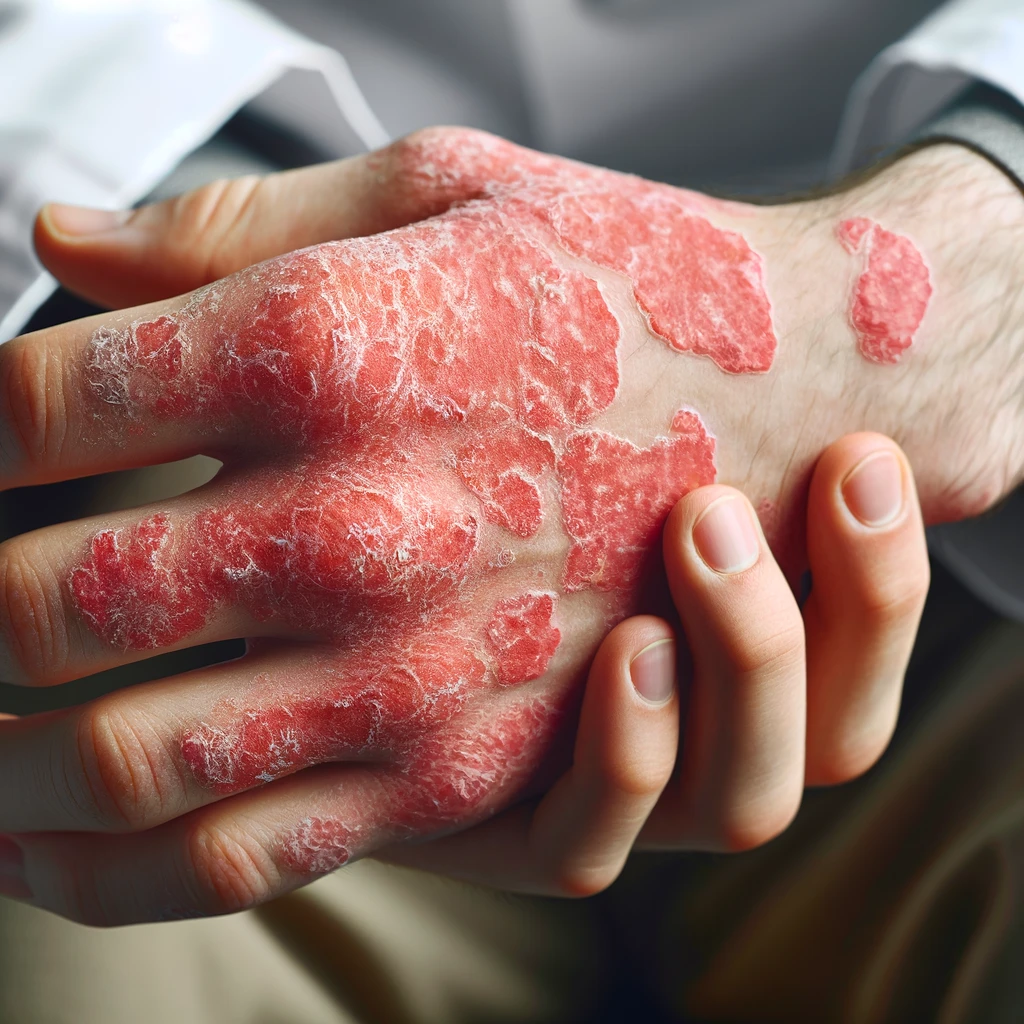

Supplement: Multimedia Appendix 1 [file ai_v3i1e58275_app1.zip › 77 custom GPT.png]

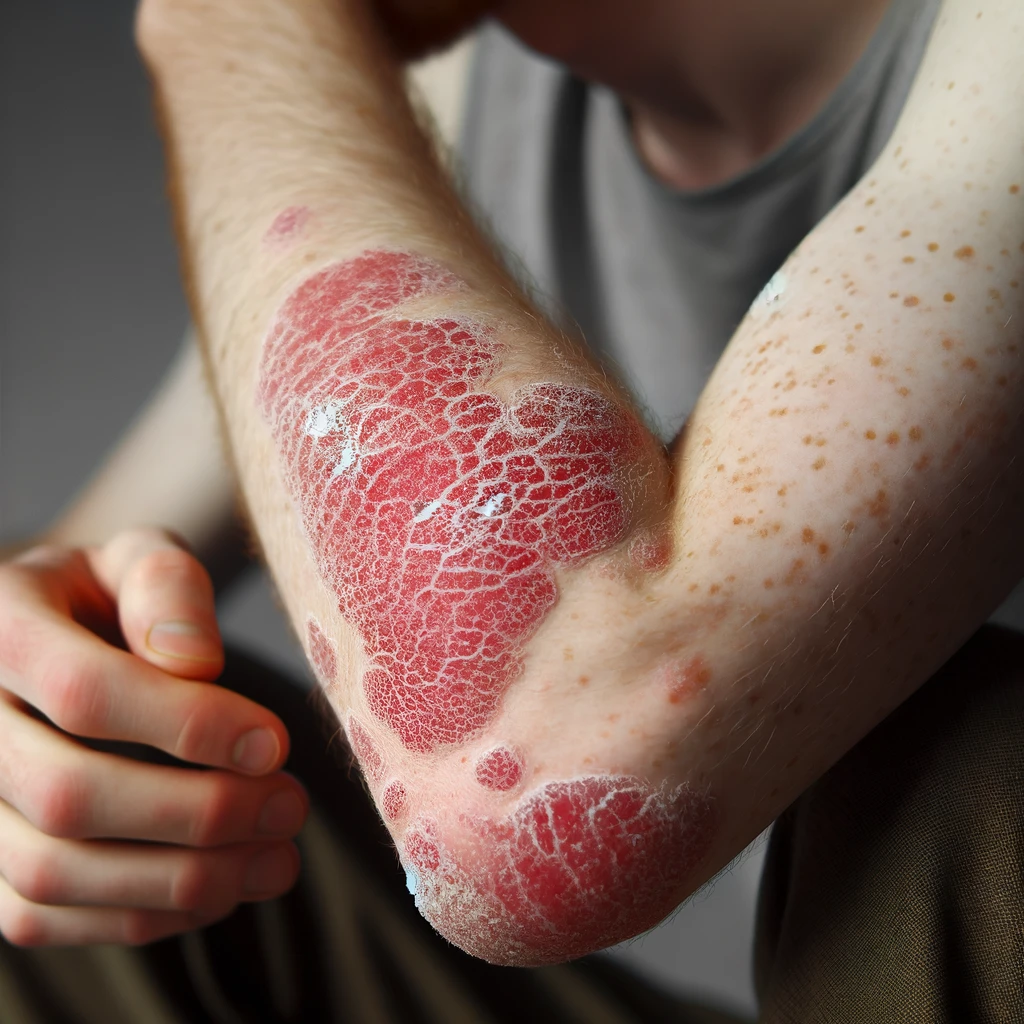

Supplement: Multimedia Appendix 1 [file ai_v3i1e58275_app1.zip › 16 custom GPT.png]

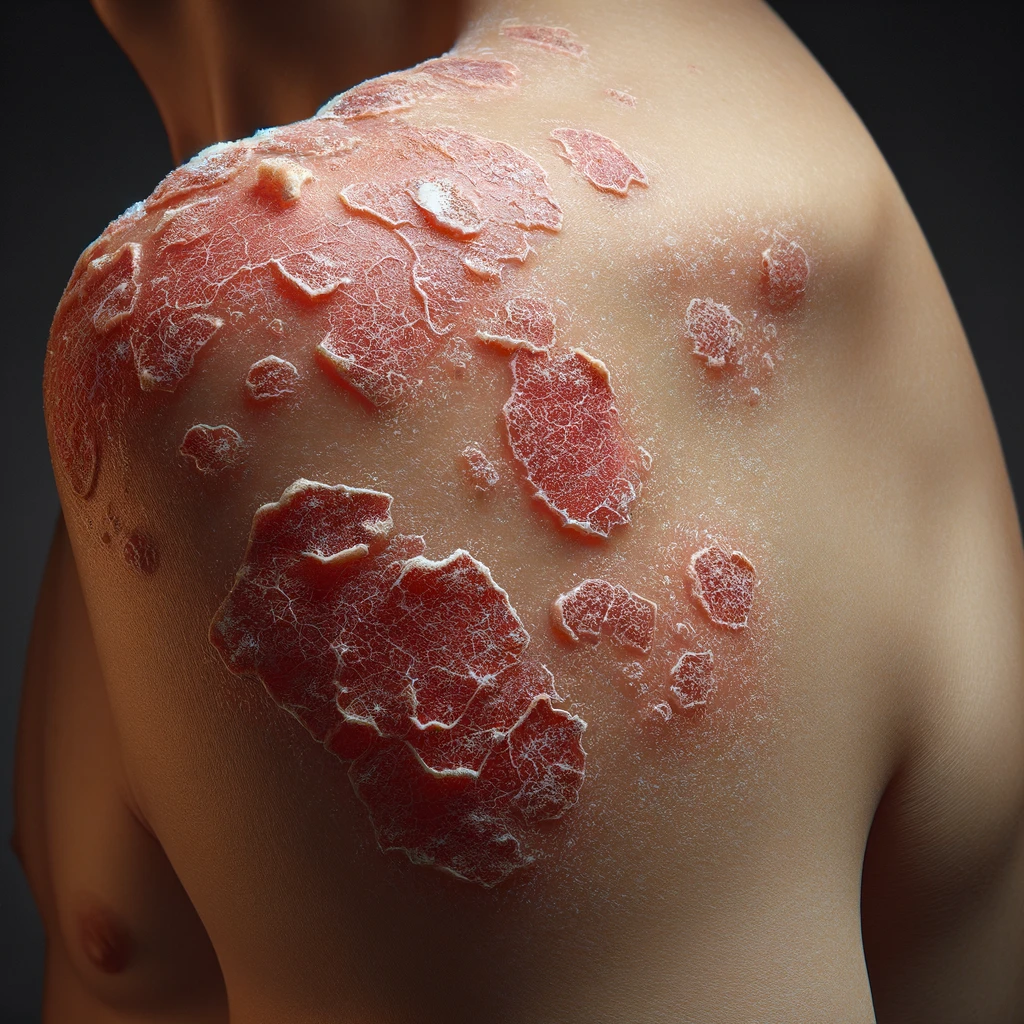

Supplement: Multimedia Appendix 1 [file ai_v3i1e58275_app1.zip › 85 custom GPT.png]

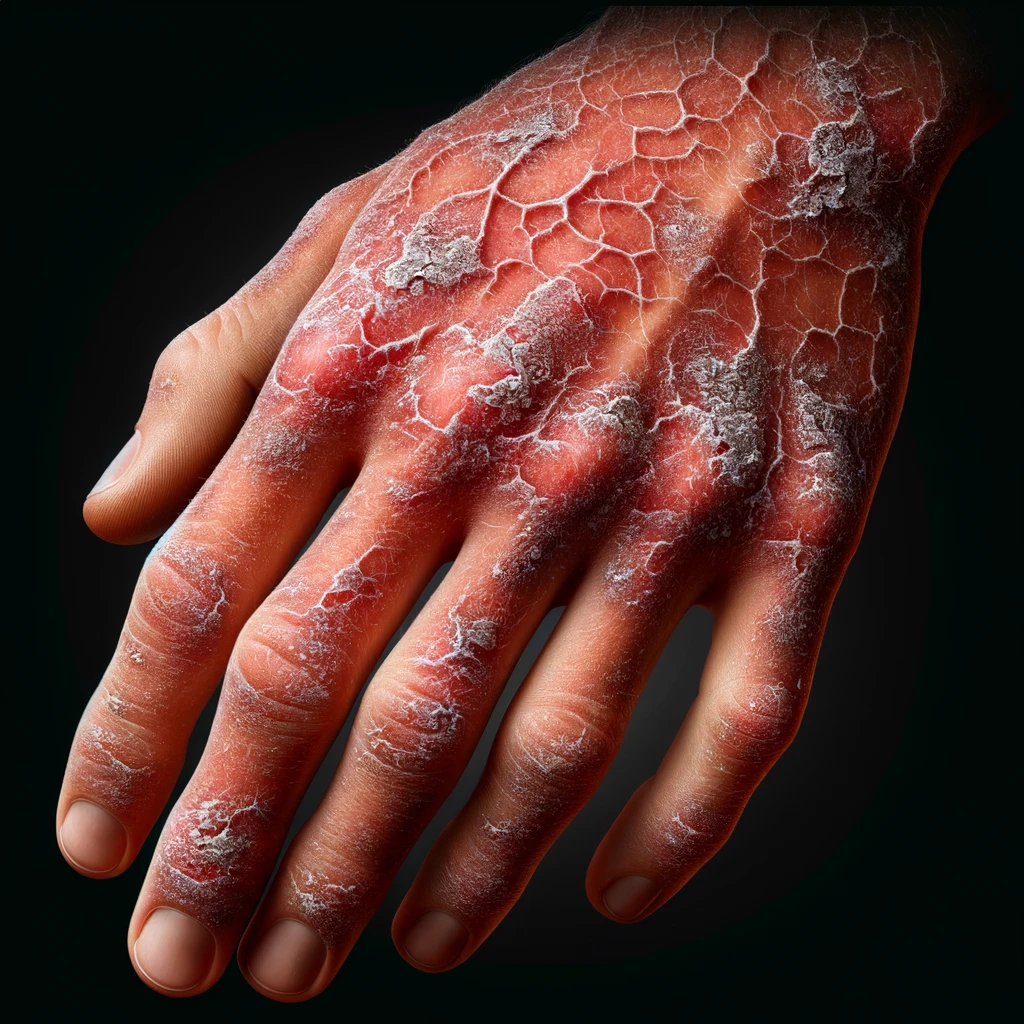

Supplement: Multimedia Appendix 1 [file ai_v3i1e58275_app1.zip › 21 custom GPT.png]

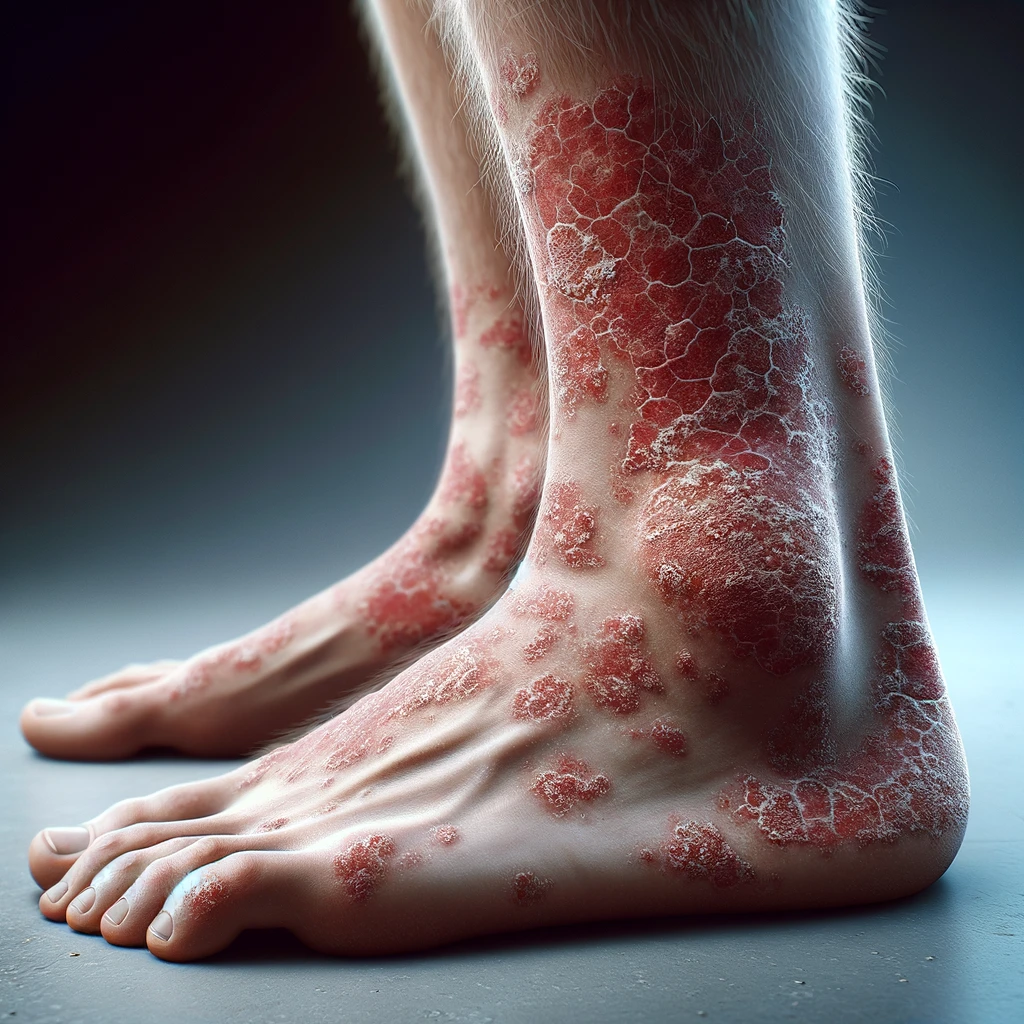

Supplement: Multimedia Appendix 1 [file ai_v3i1e58275_app1.zip › 91 custom GPT.png]

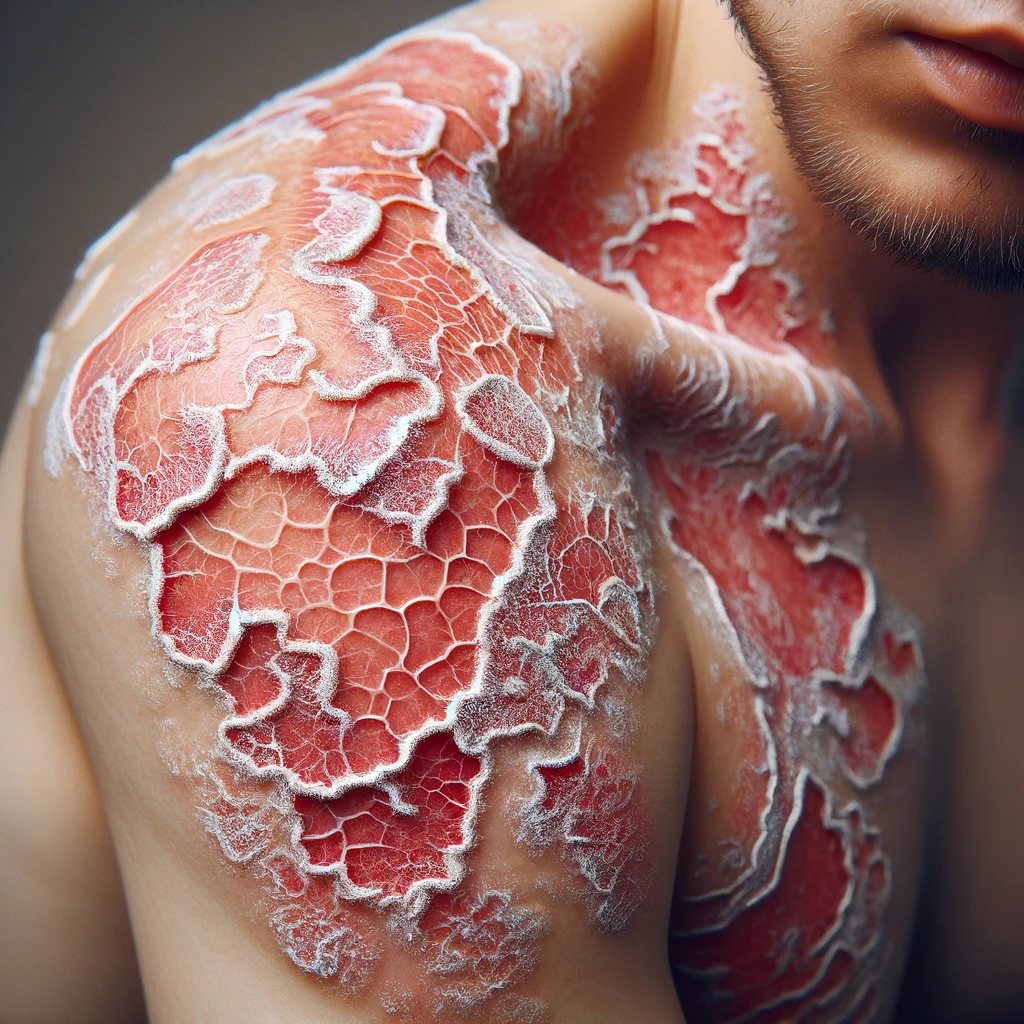

Supplement: Multimedia Appendix 1 [file ai_v3i1e58275_app1.zip › 40 custom GPT.png]

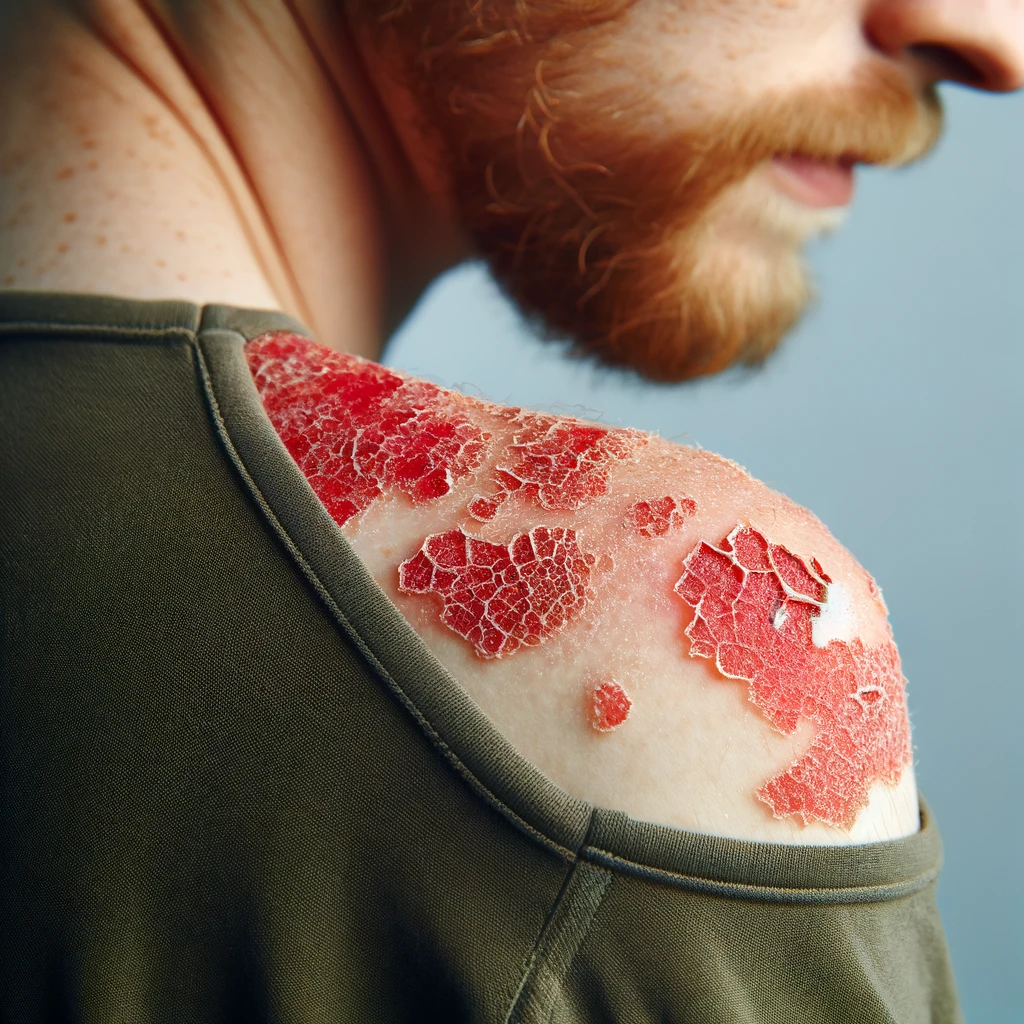

Supplement: Multimedia Appendix 1 [file ai_v3i1e58275_app1.zip › 68 custom GPT.png]

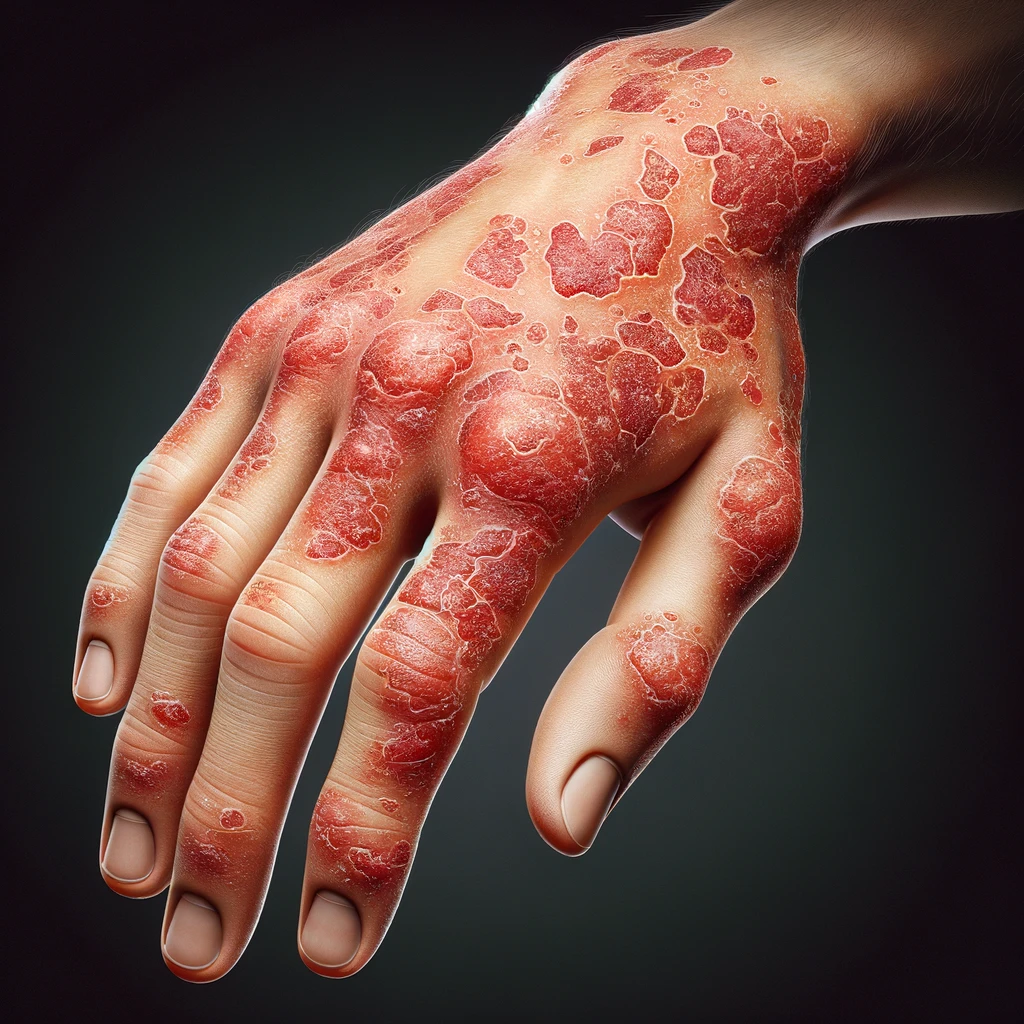

Supplement: Multimedia Appendix 1 [file ai_v3i1e58275_app1.zip › 72 custom GPT.png]

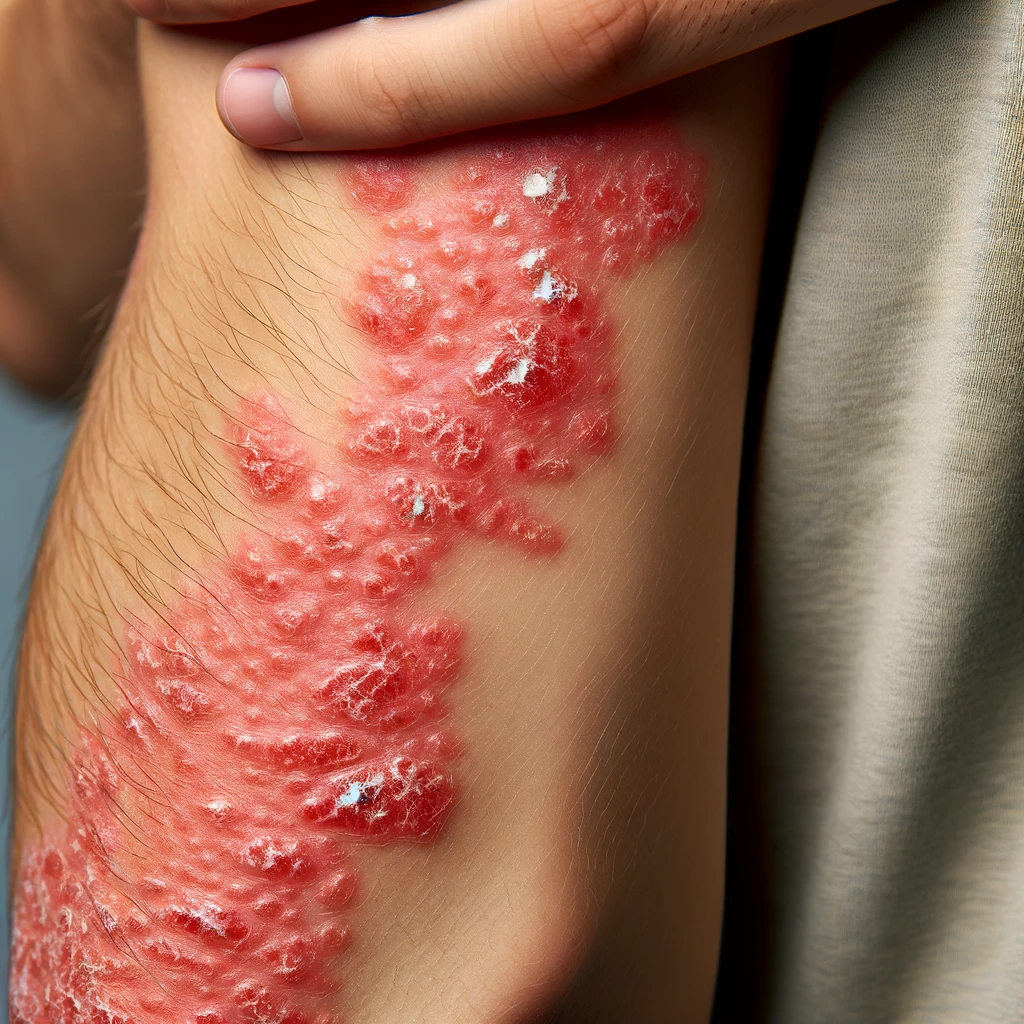

Supplement: Multimedia Appendix 1 [file ai_v3i1e58275_app1.zip › 07 custom GPT.png]

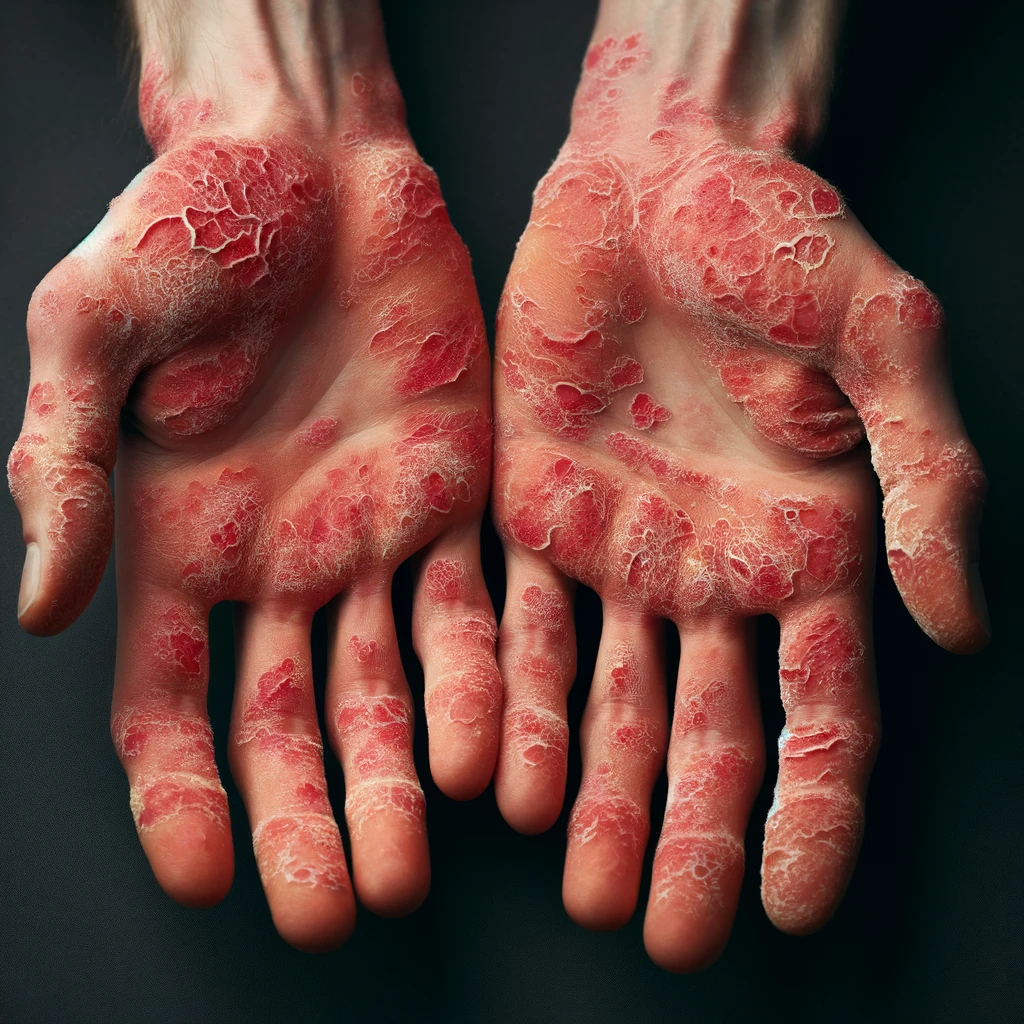

Supplement: Multimedia Appendix 1 [file ai_v3i1e58275_app1.zip › 23 custom GPT.png]

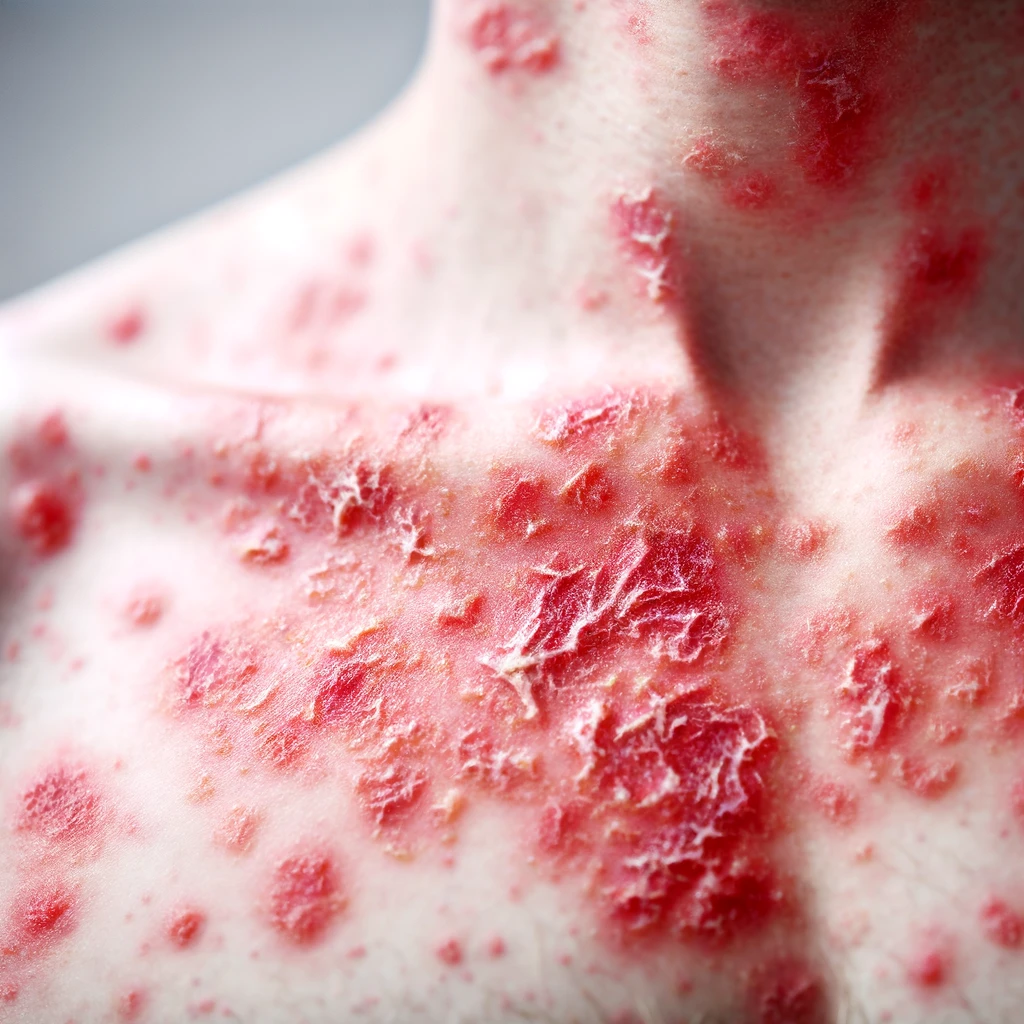

Supplement: Multimedia Appendix 1 [file ai_v3i1e58275_app1.zip › 62 custom GPT.png]

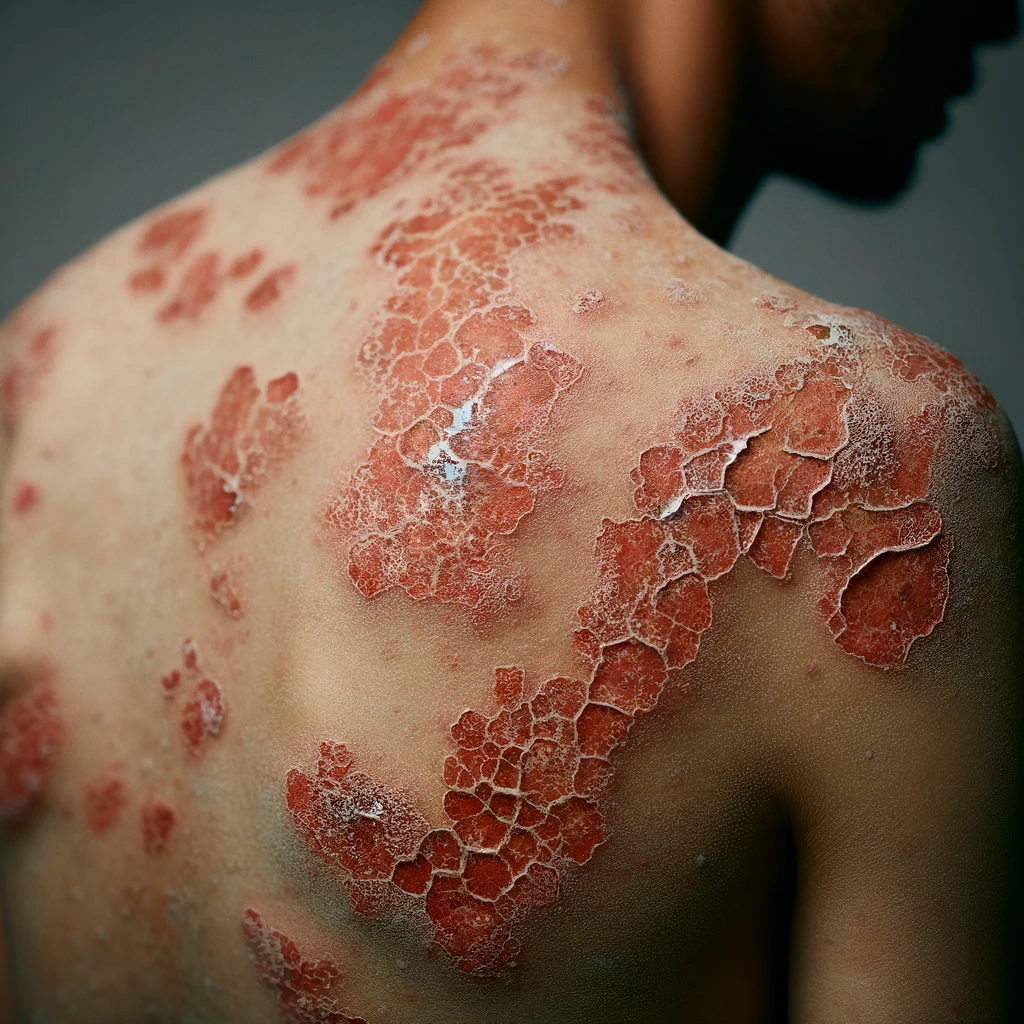

Supplement: Multimedia Appendix 1 [file ai_v3i1e58275_app1.zip › 28 custom GPT.png]

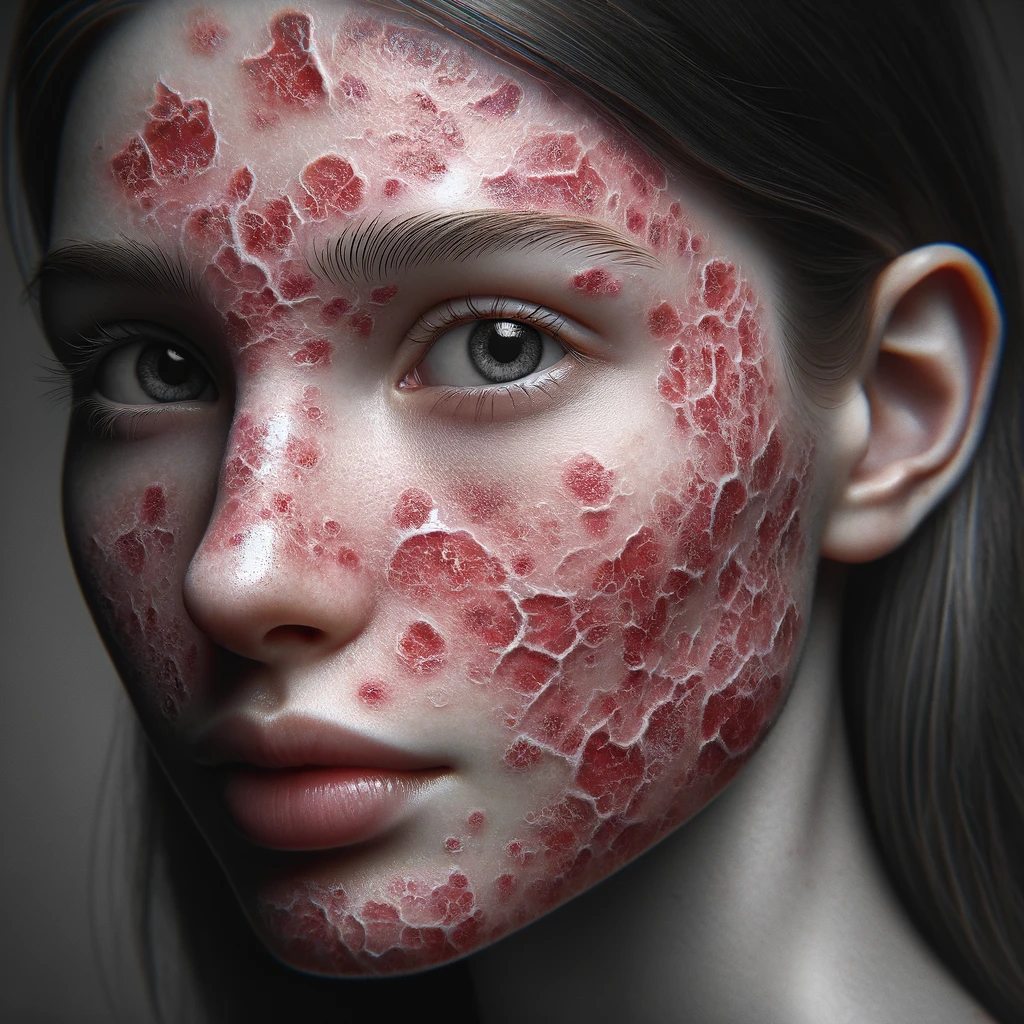

Supplement: Multimedia Appendix 1 [file ai_v3i1e58275_app1.zip › 27 custom GPT.png]

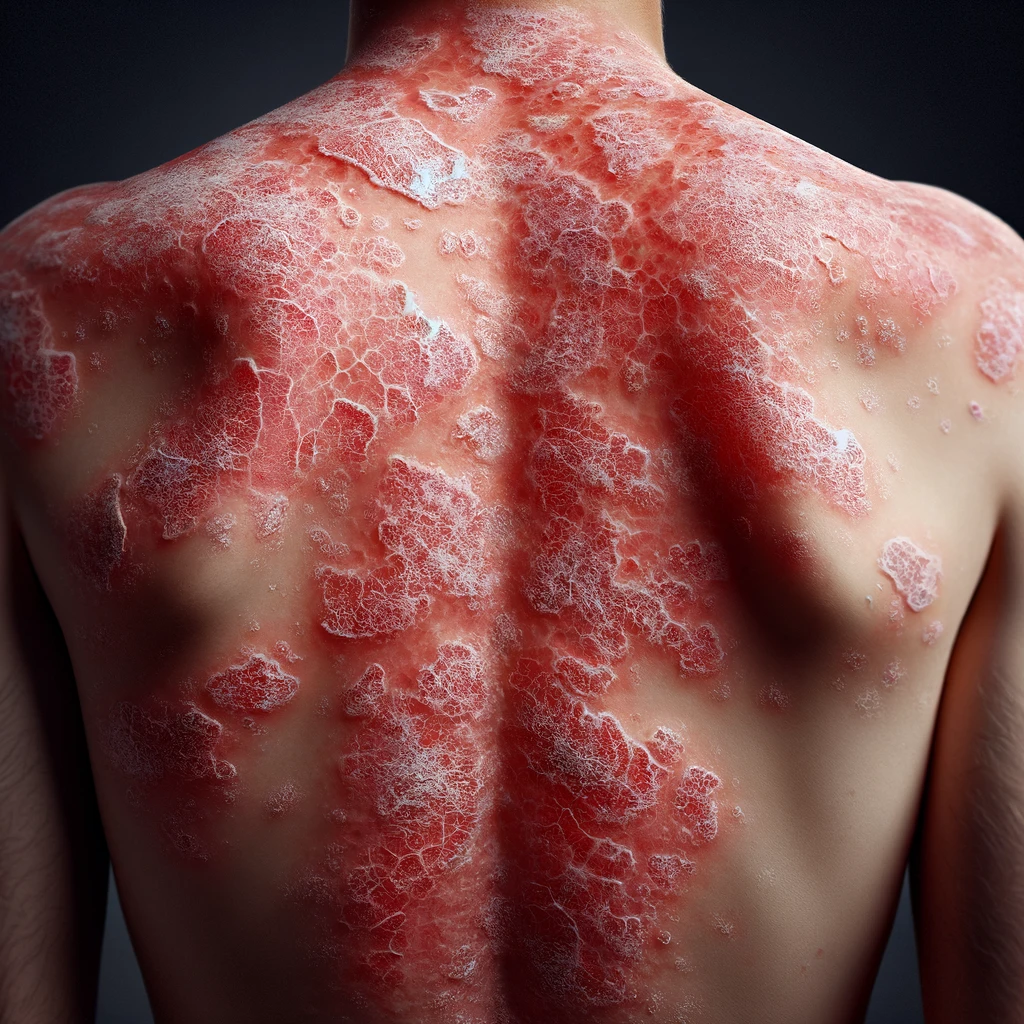

Supplement: Multimedia Appendix 1 [file ai_v3i1e58275_app1.zip › 84 custom GPT.png]

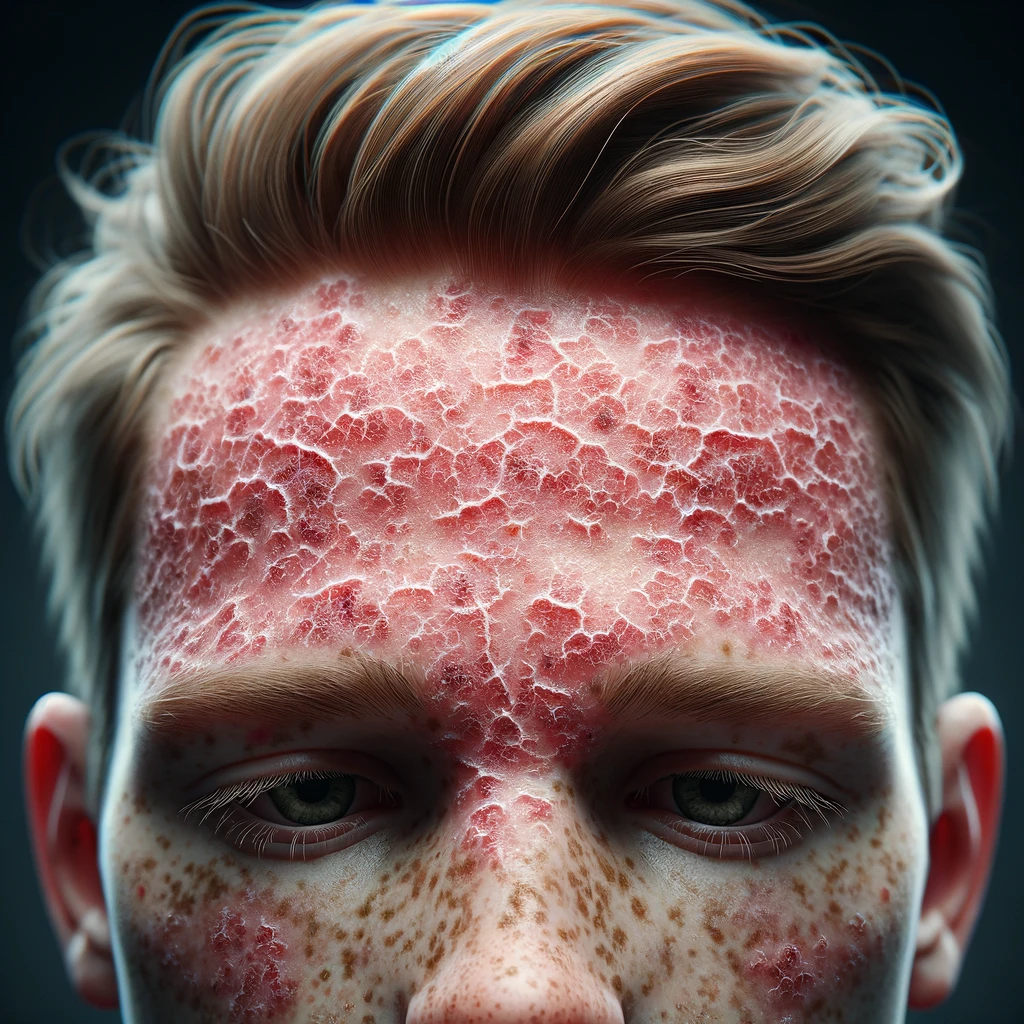

Supplement: Multimedia Appendix 1 [file ai_v3i1e58275_app1.zip › 89 custom GPT.png]

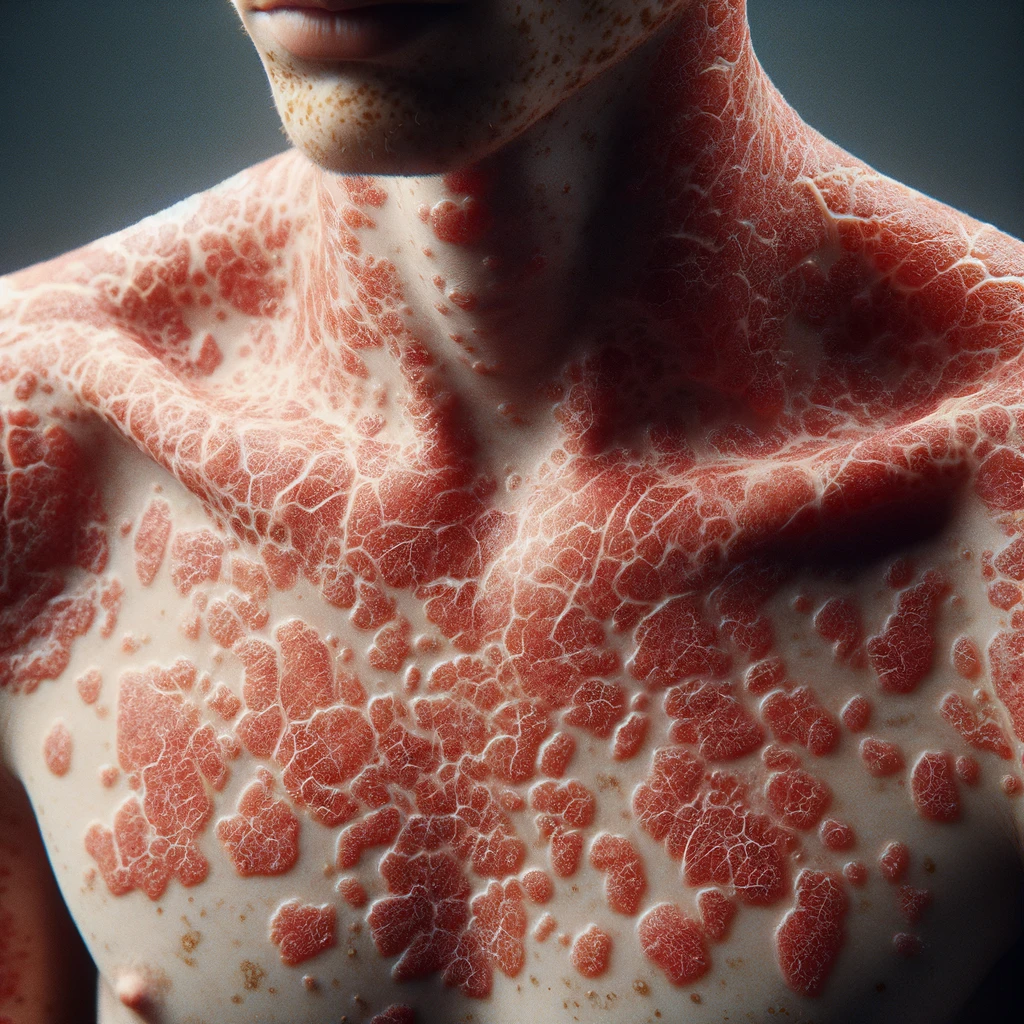

Supplement: Multimedia Appendix 1 [file ai_v3i1e58275_app1.zip › 32 custom GPT.png]

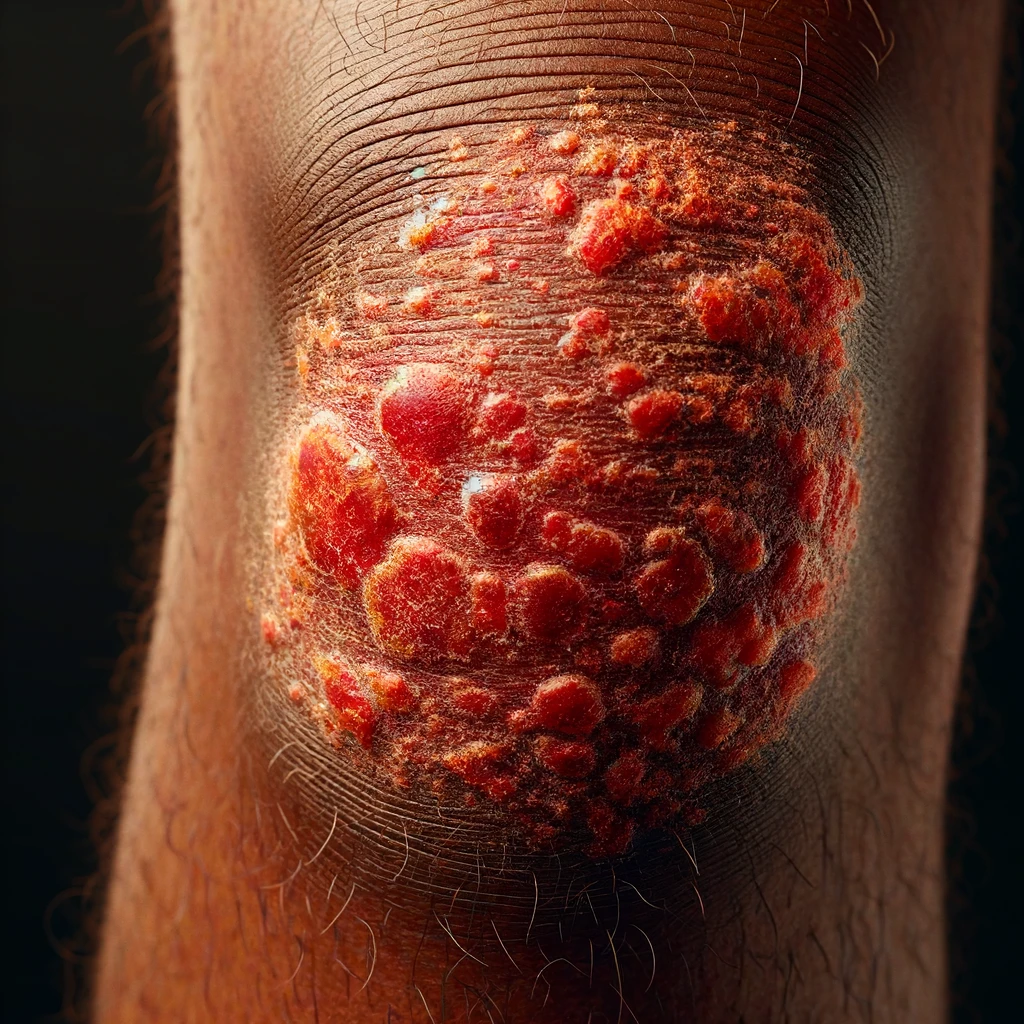

Supplement: Multimedia Appendix 1 [file ai_v3i1e58275_app1.zip › 34 custom GPT.png]

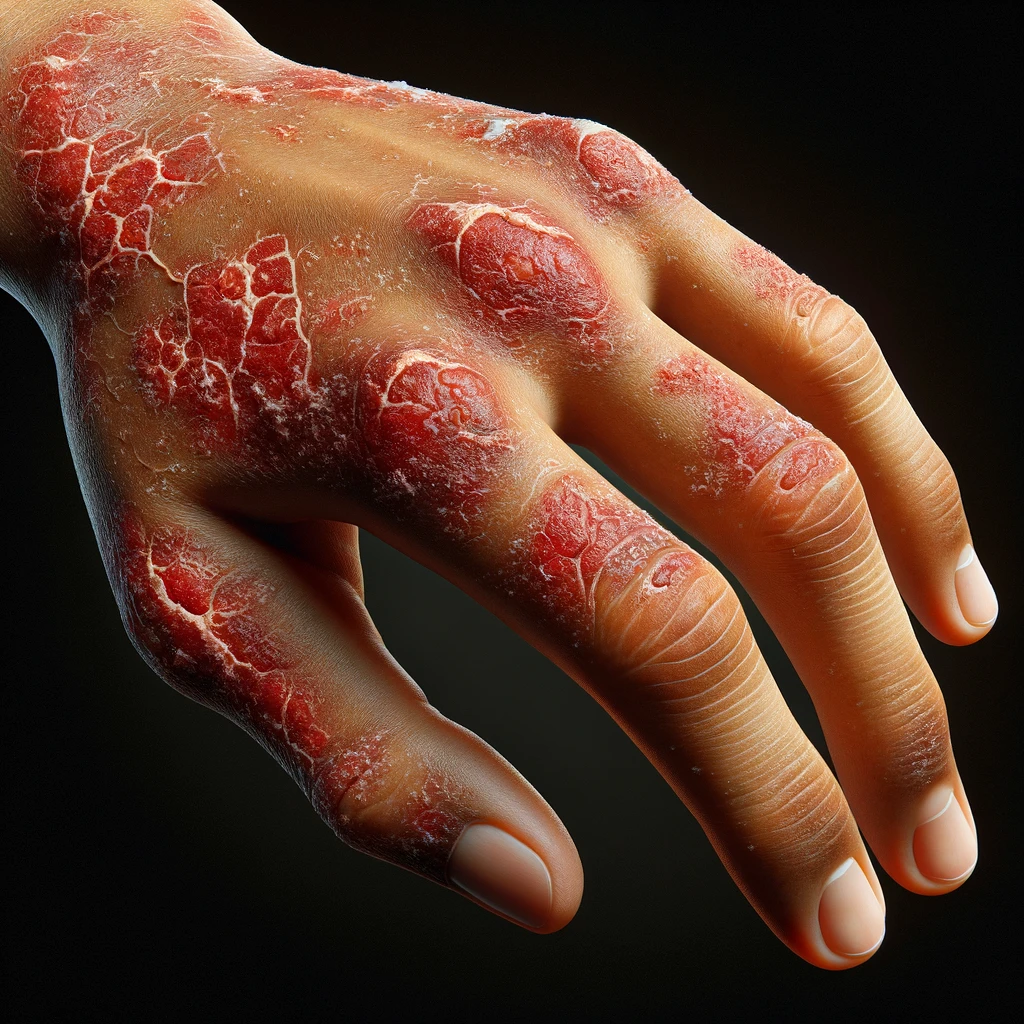

Supplement: Multimedia Appendix 1 [file ai_v3i1e58275_app1.zip › 47 custom GPT.png]

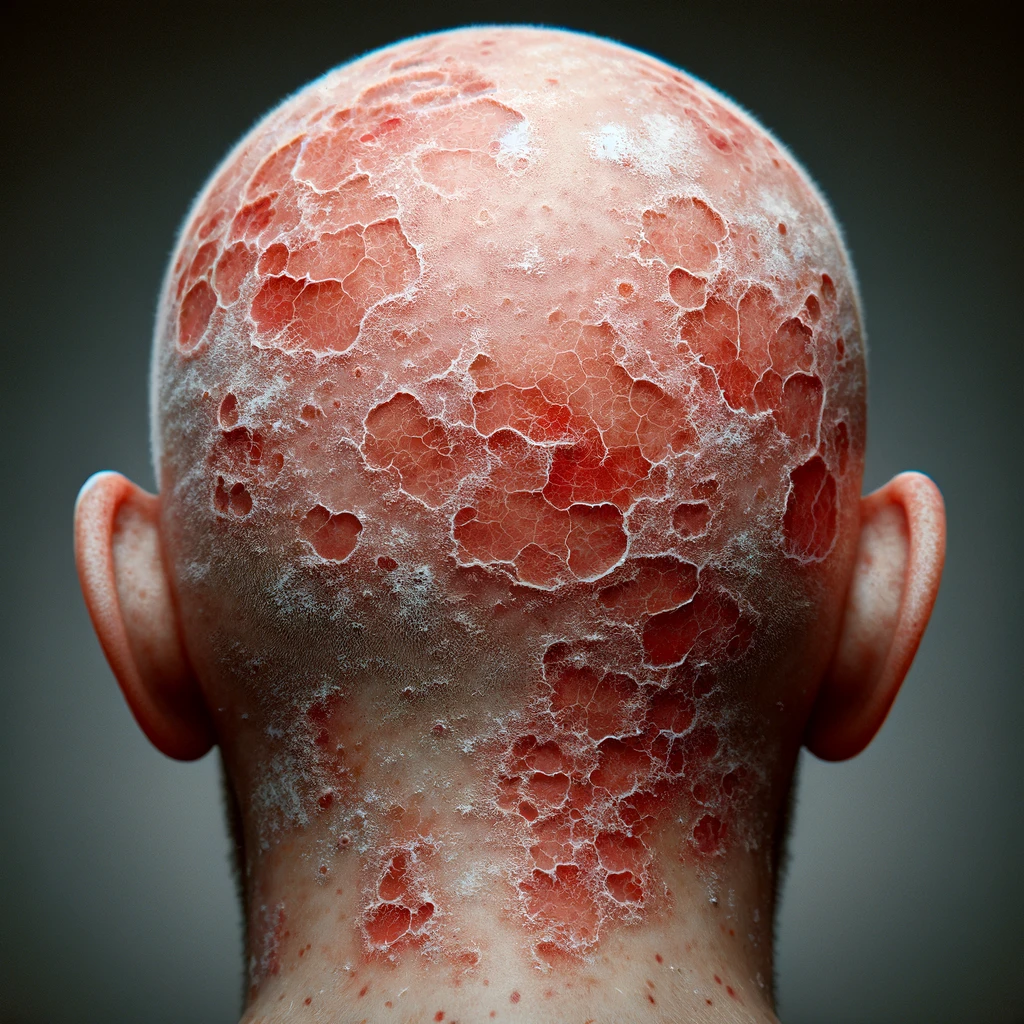

Supplement: Multimedia Appendix 1 [file ai_v3i1e58275_app1.zip › 90 custom GPT.png]

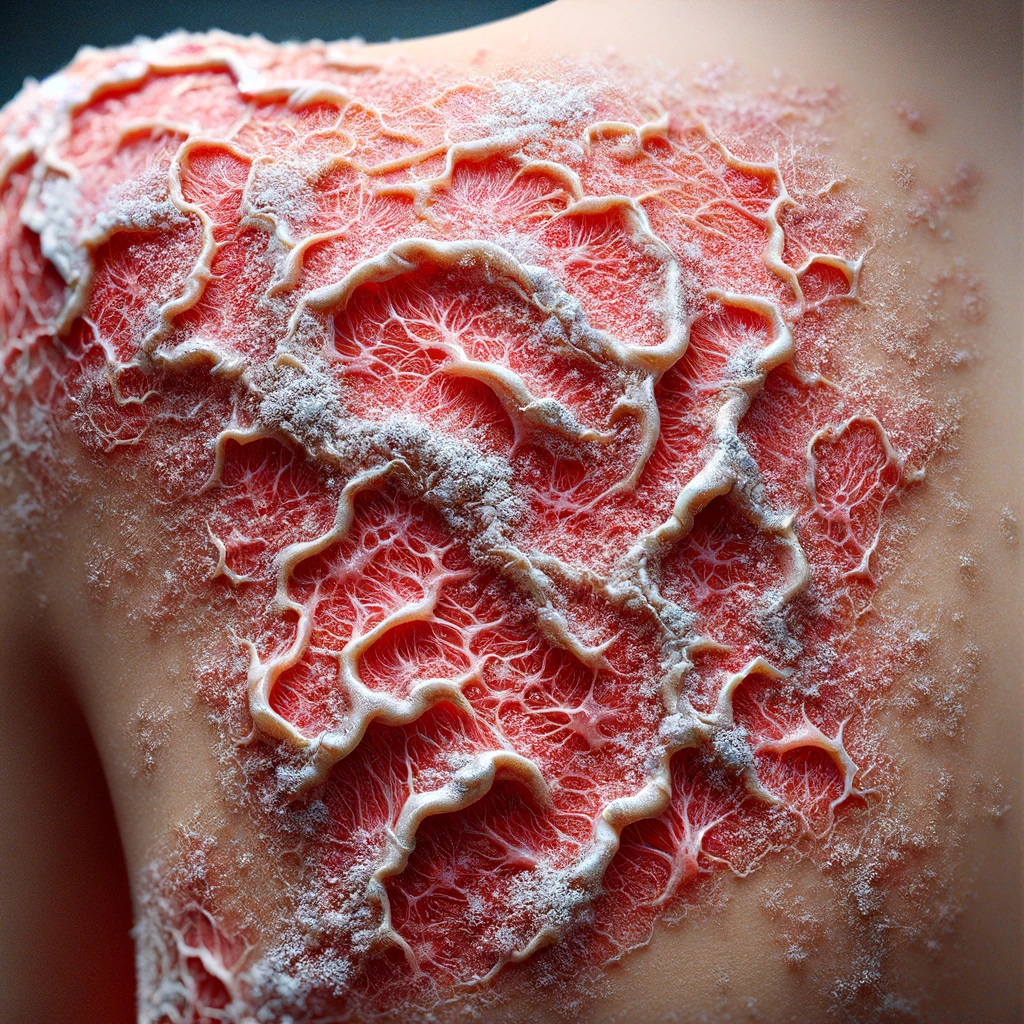

Supplement: Multimedia Appendix 1 [file ai_v3i1e58275_app1.zip › 13 custom GPT.png]

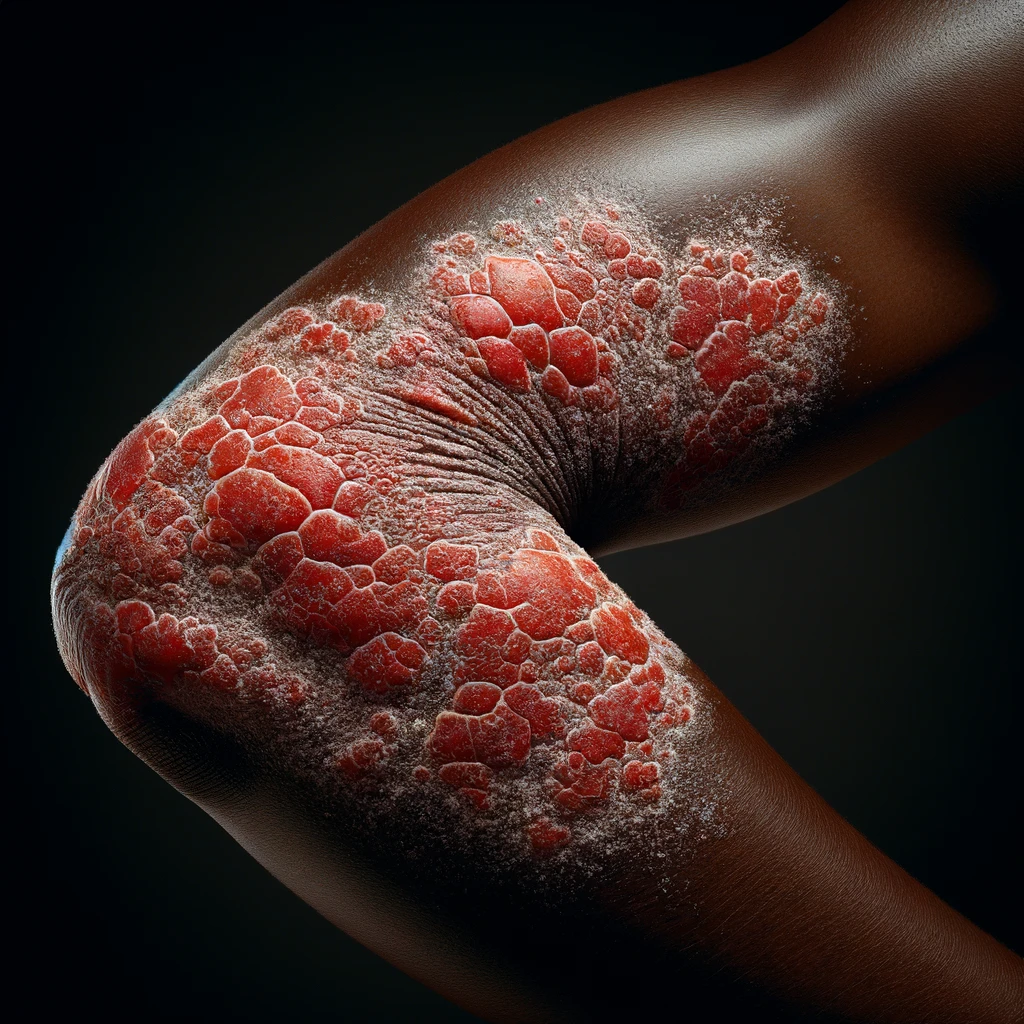

Supplement: Multimedia Appendix 1 [file ai_v3i1e58275_app1.zip › 94 custom GPT.png]

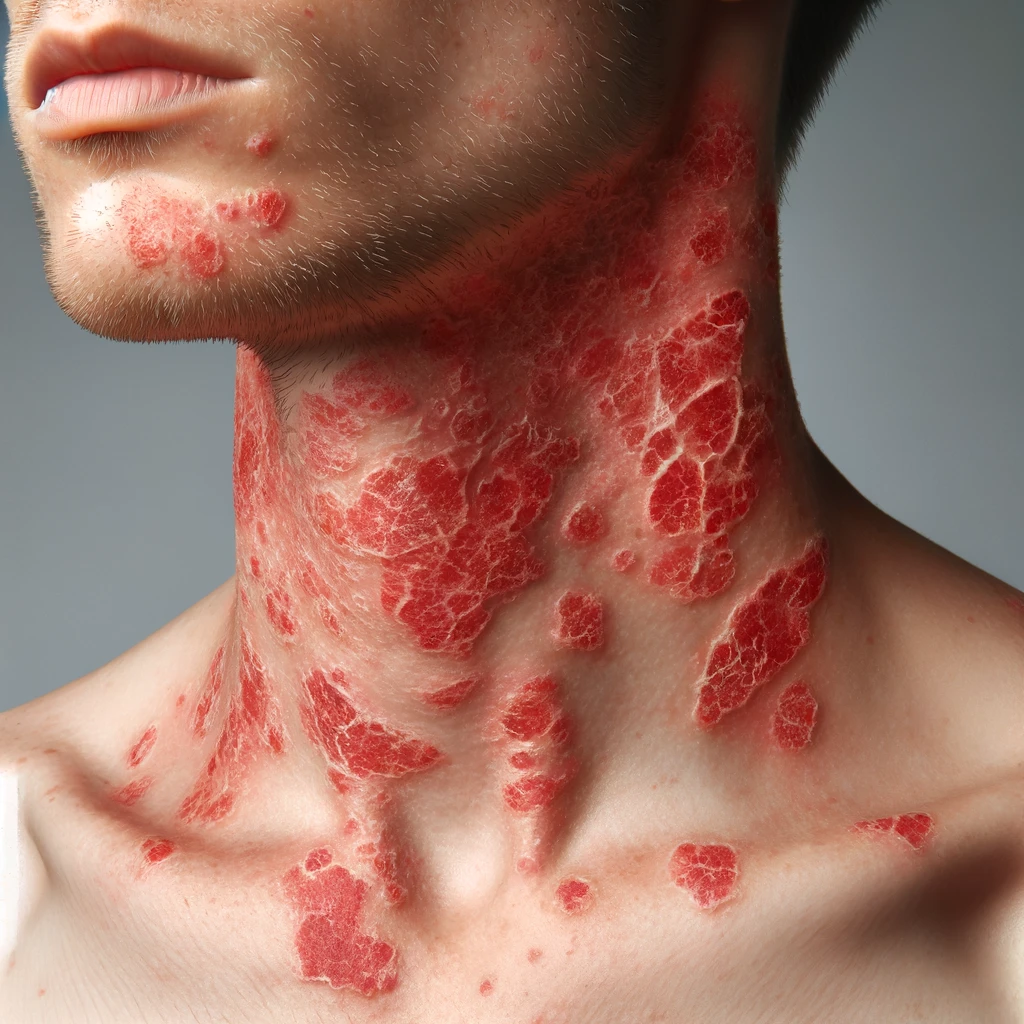

Supplement: Multimedia Appendix 1 [file ai_v3i1e58275_app1.zip › 38 custom GPT.png]

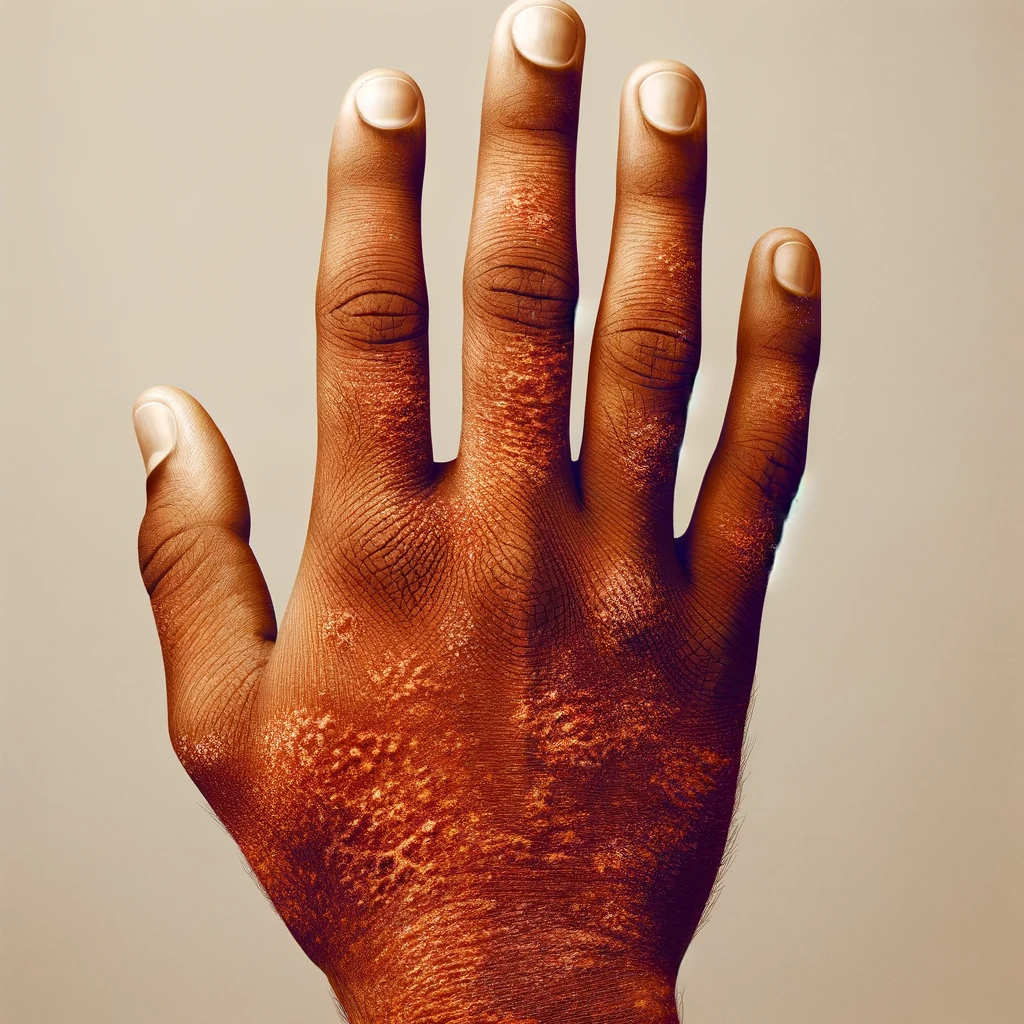

Supplement: Multimedia Appendix 1 [file ai_v3i1e58275_app1.zip › 53 custom GPT.png]

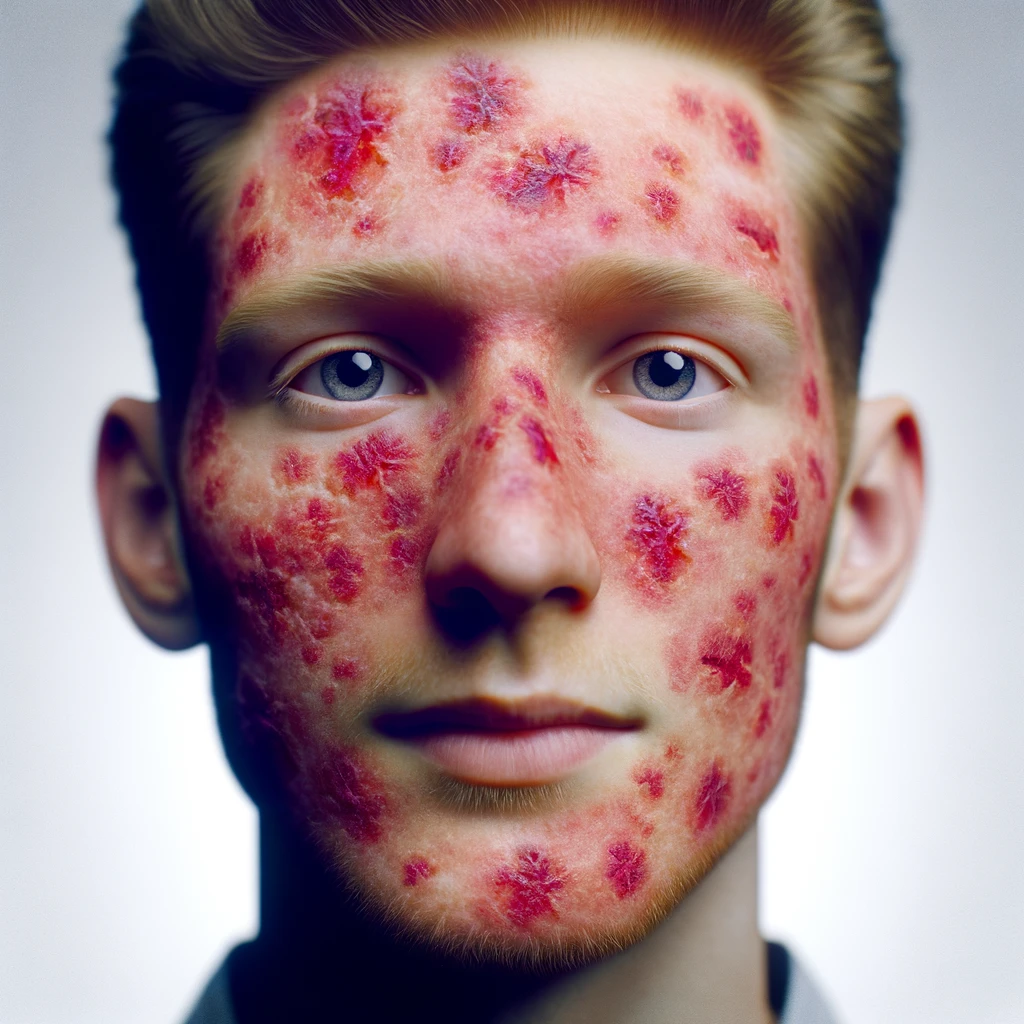

Supplement: Multimedia Appendix 1 [file ai_v3i1e58275_app1.zip › 74 custom GPT.png]

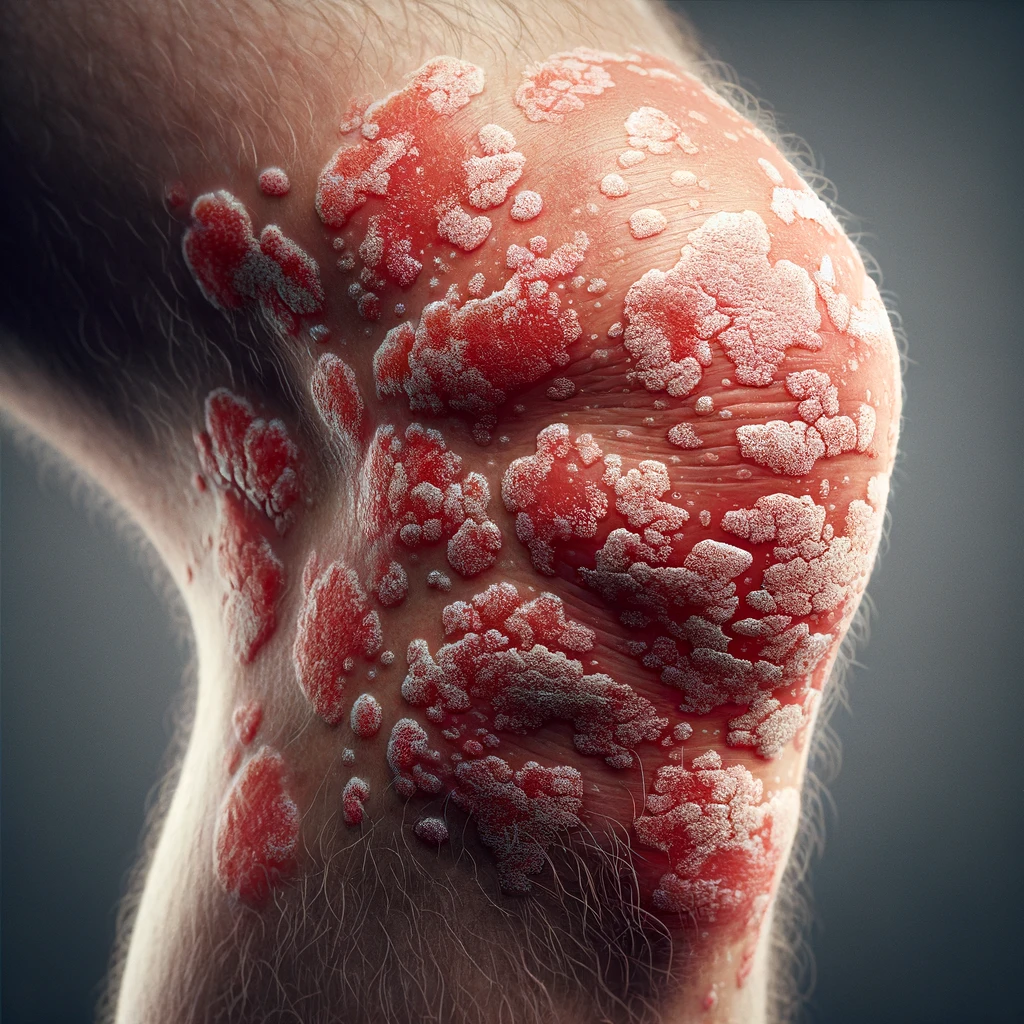

Supplement: Multimedia Appendix 1 [file ai_v3i1e58275_app1.zip › 88 custom GPT.png]

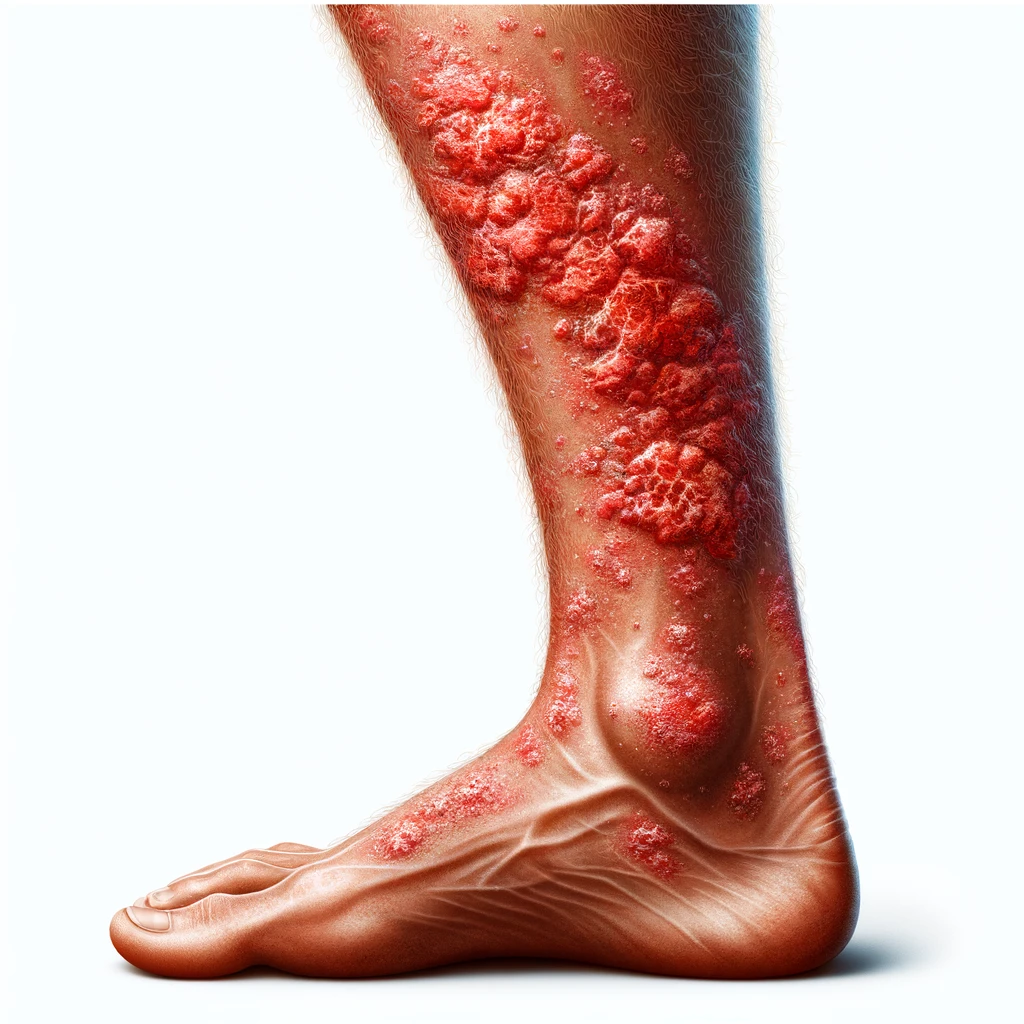

Supplement: Multimedia Appendix 1 [file ai_v3i1e58275_app1.zip › 30 custom GPT.png]

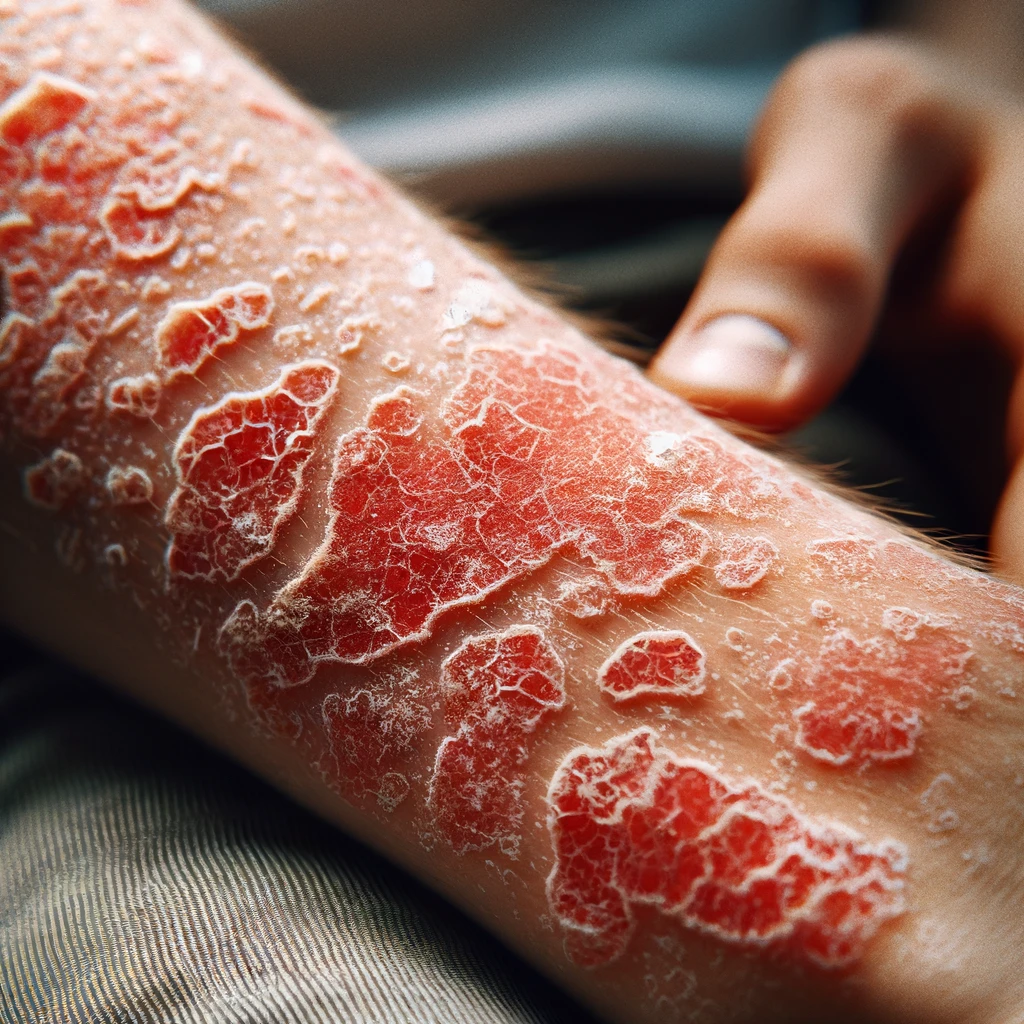

Supplement: Multimedia Appendix 1 [file ai_v3i1e58275_app1.zip › 08 custom GPT.png]

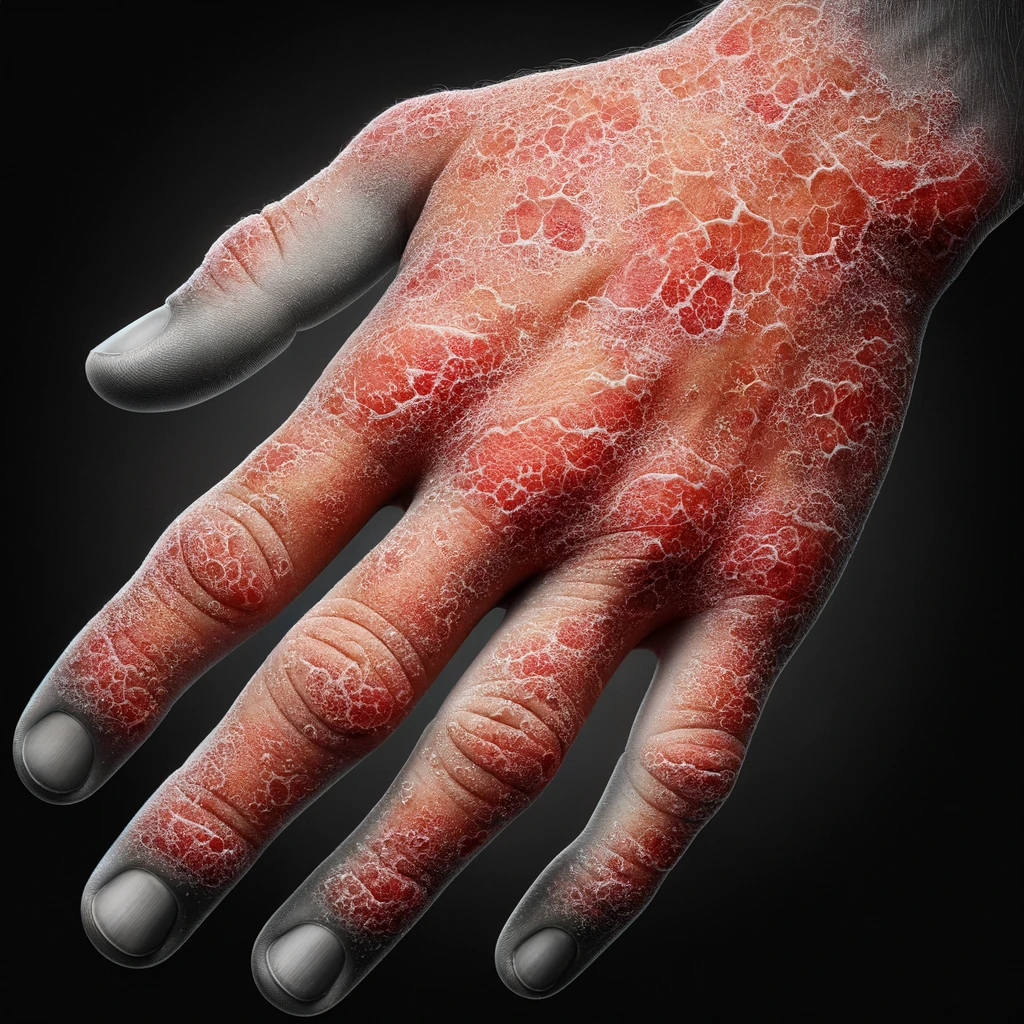

Supplement: Multimedia Appendix 1 [file ai_v3i1e58275_app1.zip › 79 custom GPT.png]

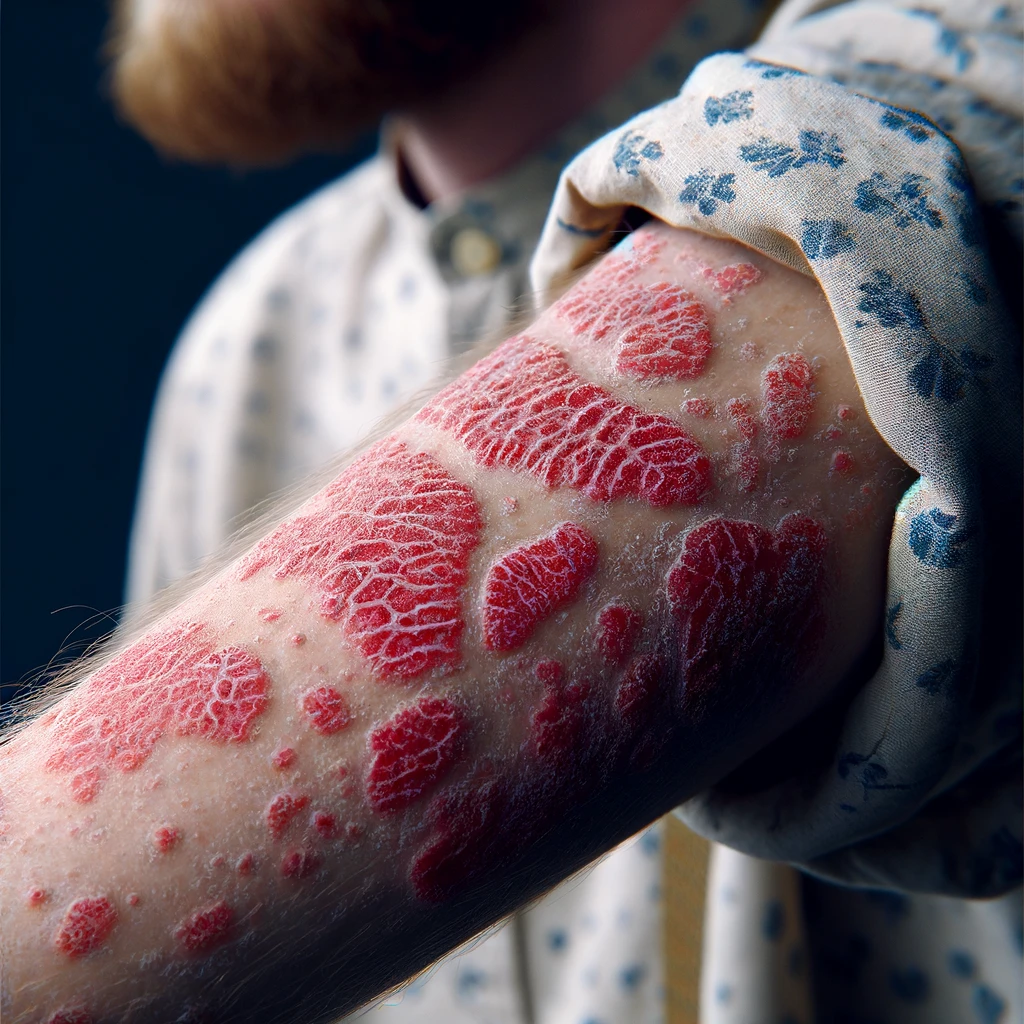

Supplement: Multimedia Appendix 1 [file ai_v3i1e58275_app1.zip › 55 custom GPT.png]

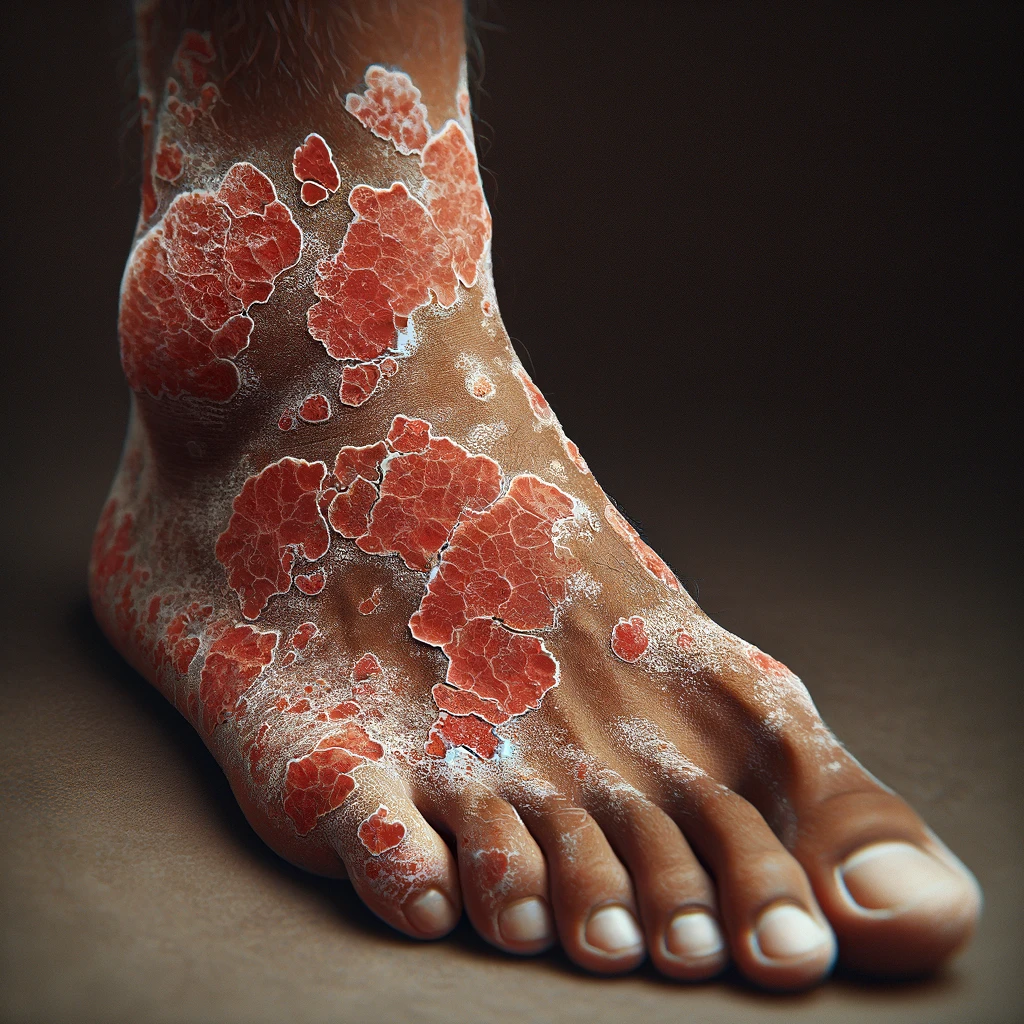

Supplement: Multimedia Appendix 1 [file ai_v3i1e58275_app1.zip › 14 custom GPT.png]

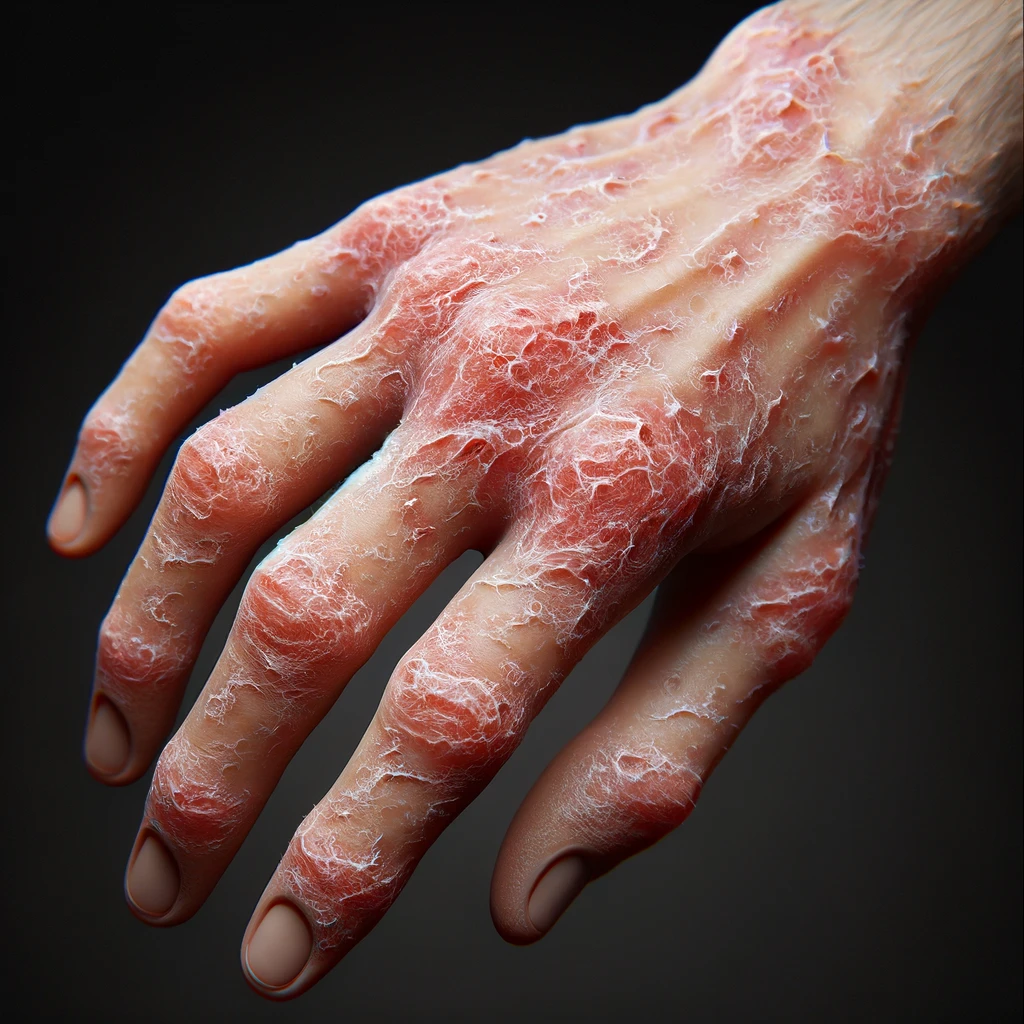

Supplement: Multimedia Appendix 1 [file ai_v3i1e58275_app1.zip › 29 custom GPT.png]

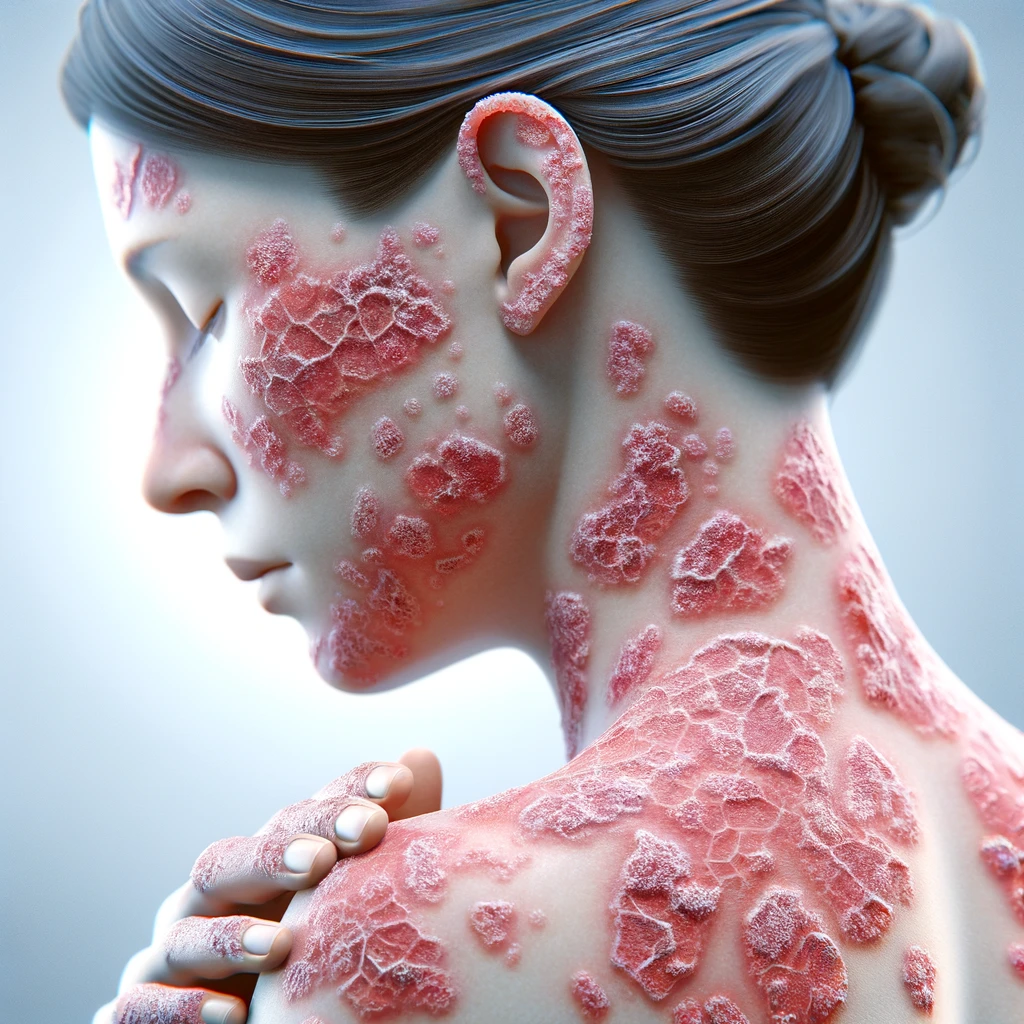

Supplement: Multimedia Appendix 1 [file ai_v3i1e58275_app1.zip › 70 custom GPT.png]

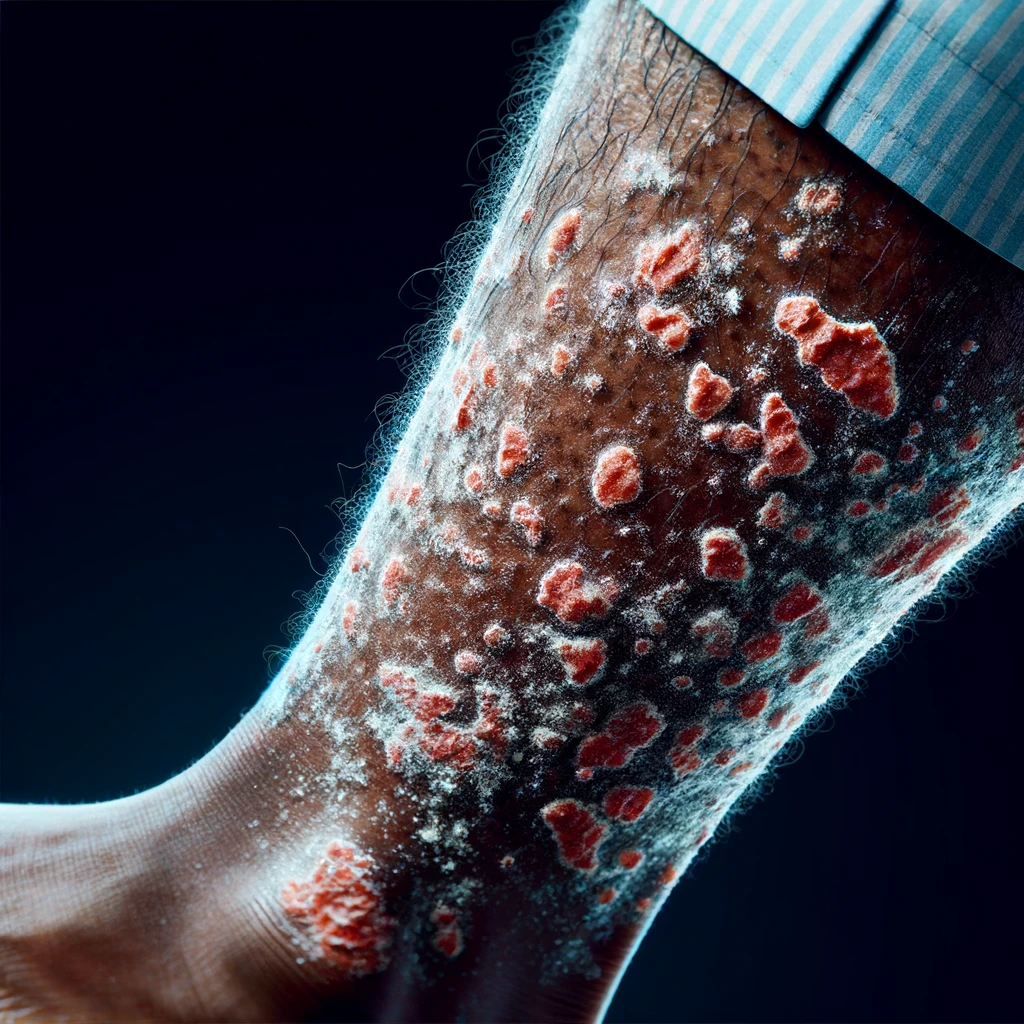

Supplement: Multimedia Appendix 1 [file ai_v3i1e58275_app1.zip › 05 custom GPT.png]

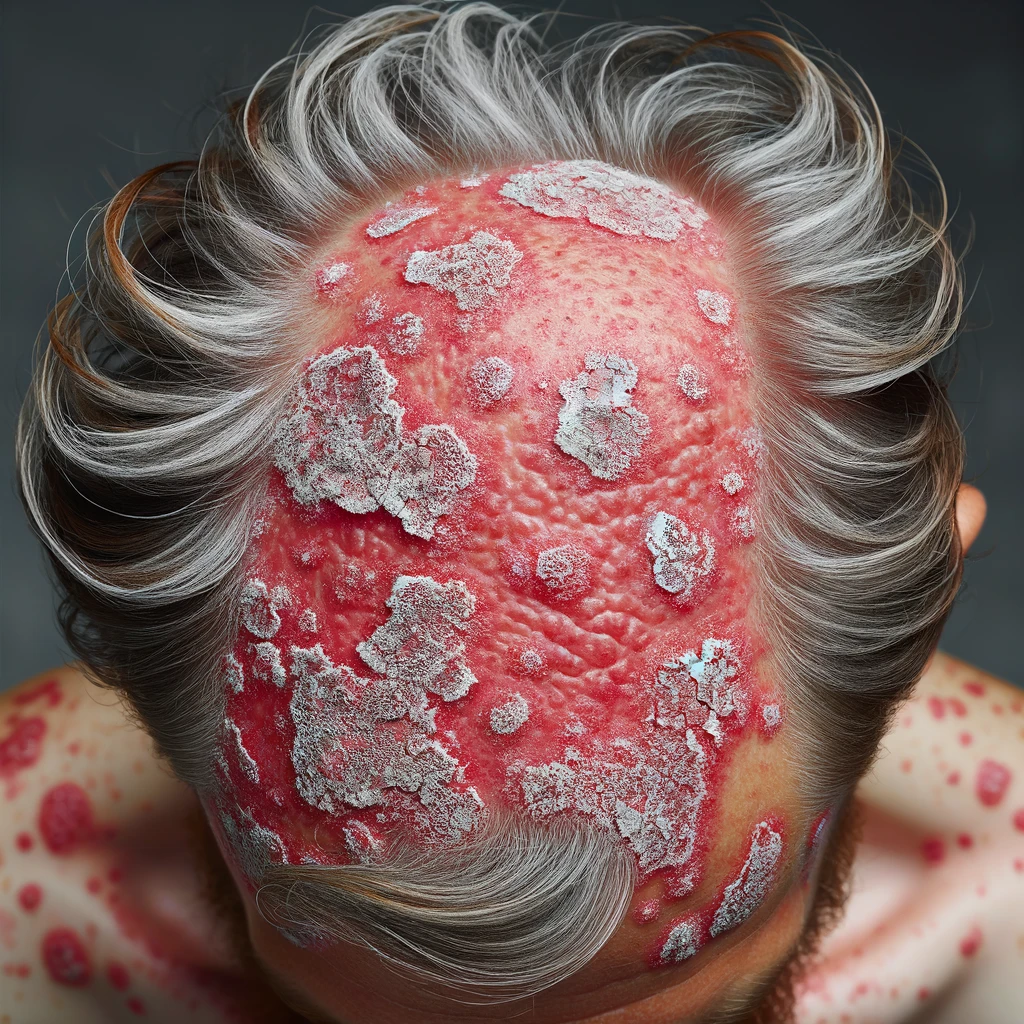

Supplement: Multimedia Appendix 1 [file ai_v3i1e58275_app1.zip › 65 custom GPT.png]

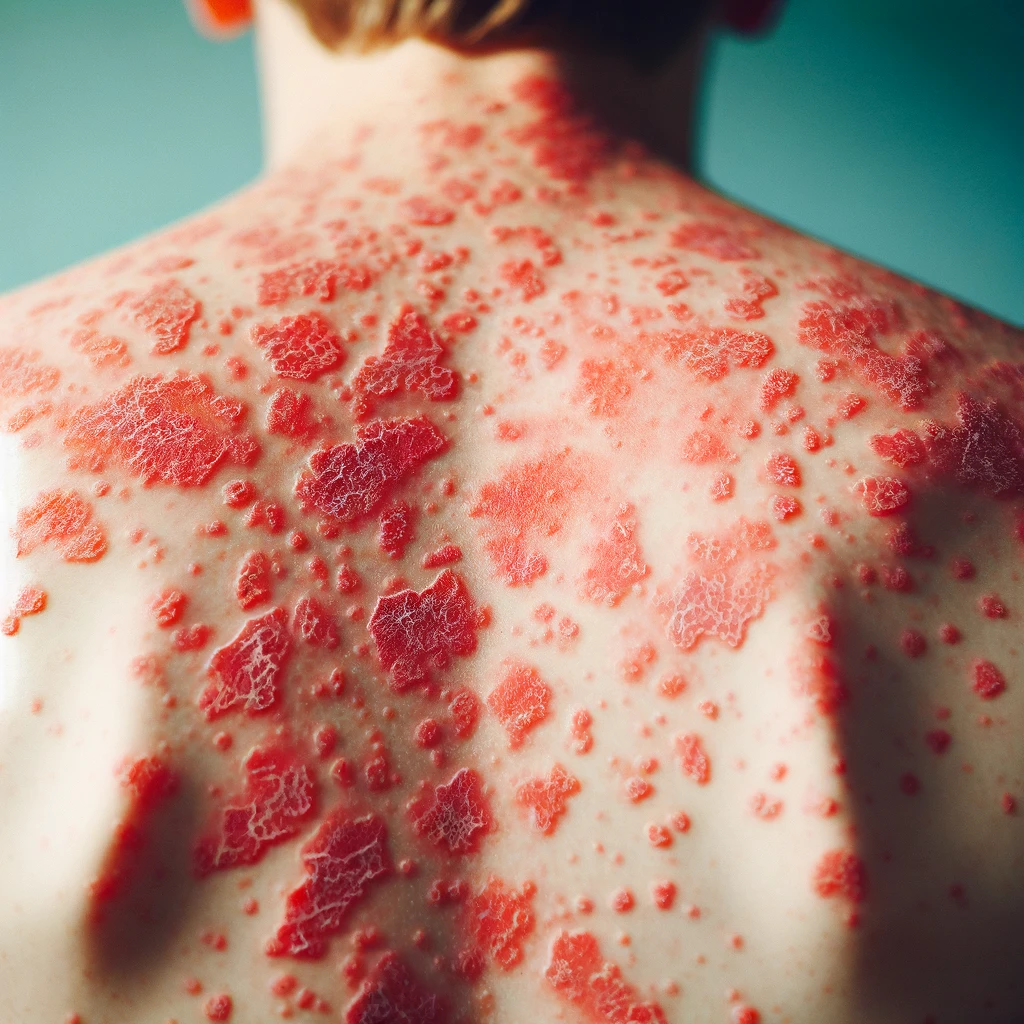

Supplement: Multimedia Appendix 1 [file ai_v3i1e58275_app1.zip › 37 custom GPT.png]

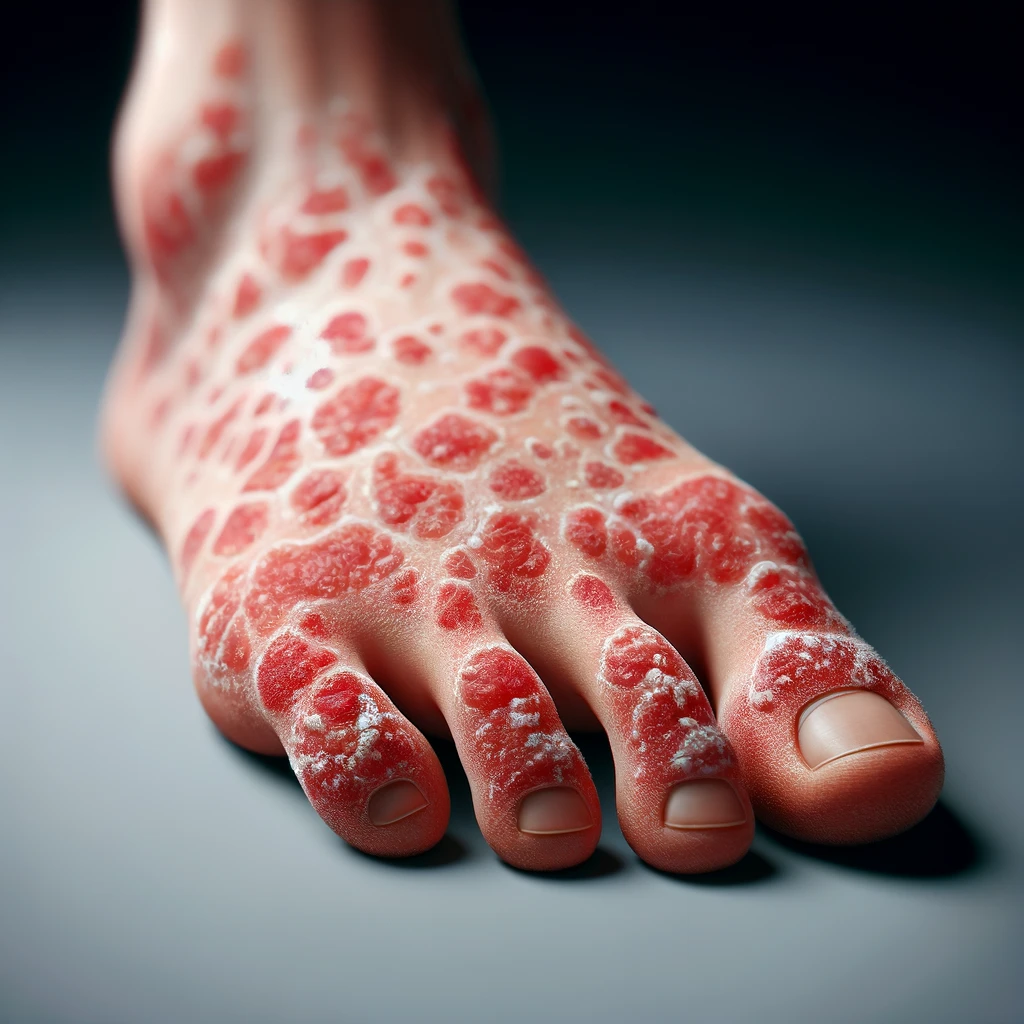

Supplement: Multimedia Appendix 1 [file ai_v3i1e58275_app1.zip › 99 custom GPT.png]

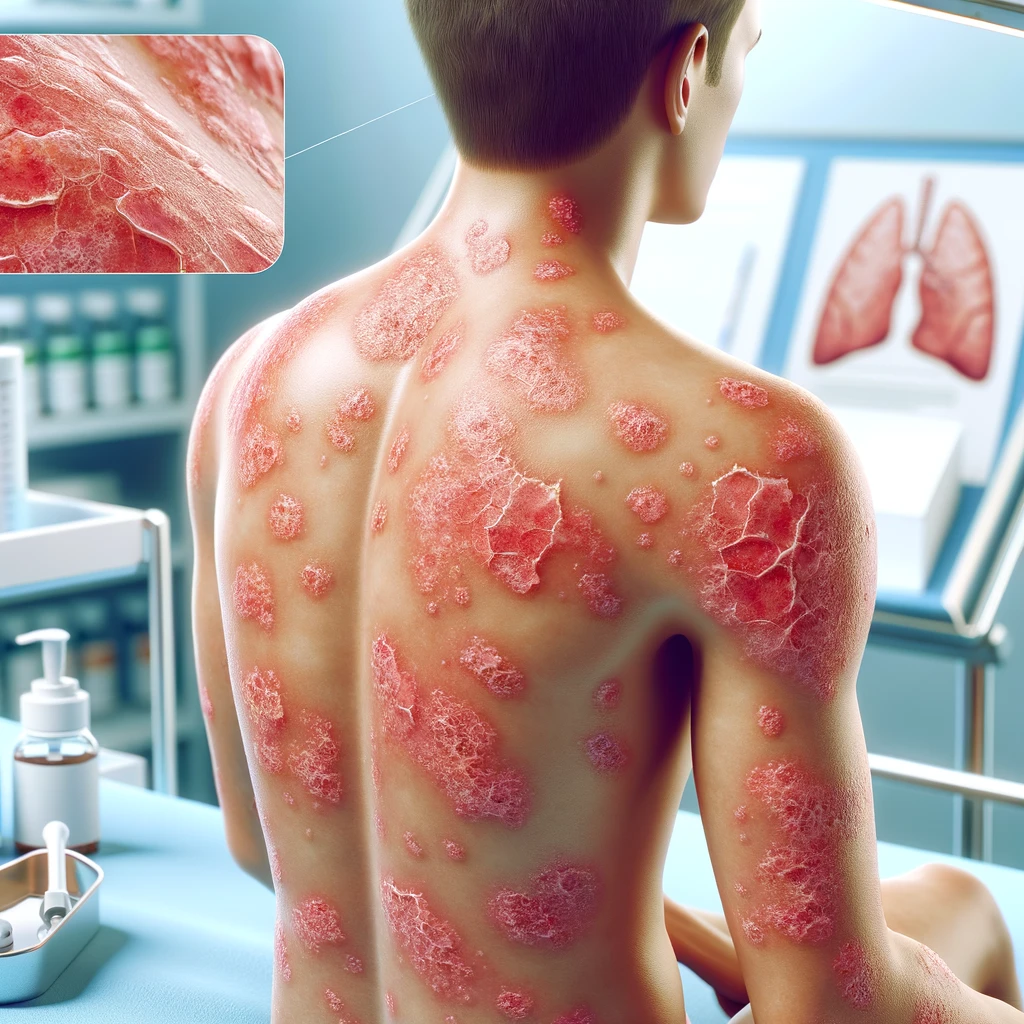

Supplement: Multimedia Appendix 1 [file ai_v3i1e58275_app1.zip › 15 custom GPT.png]

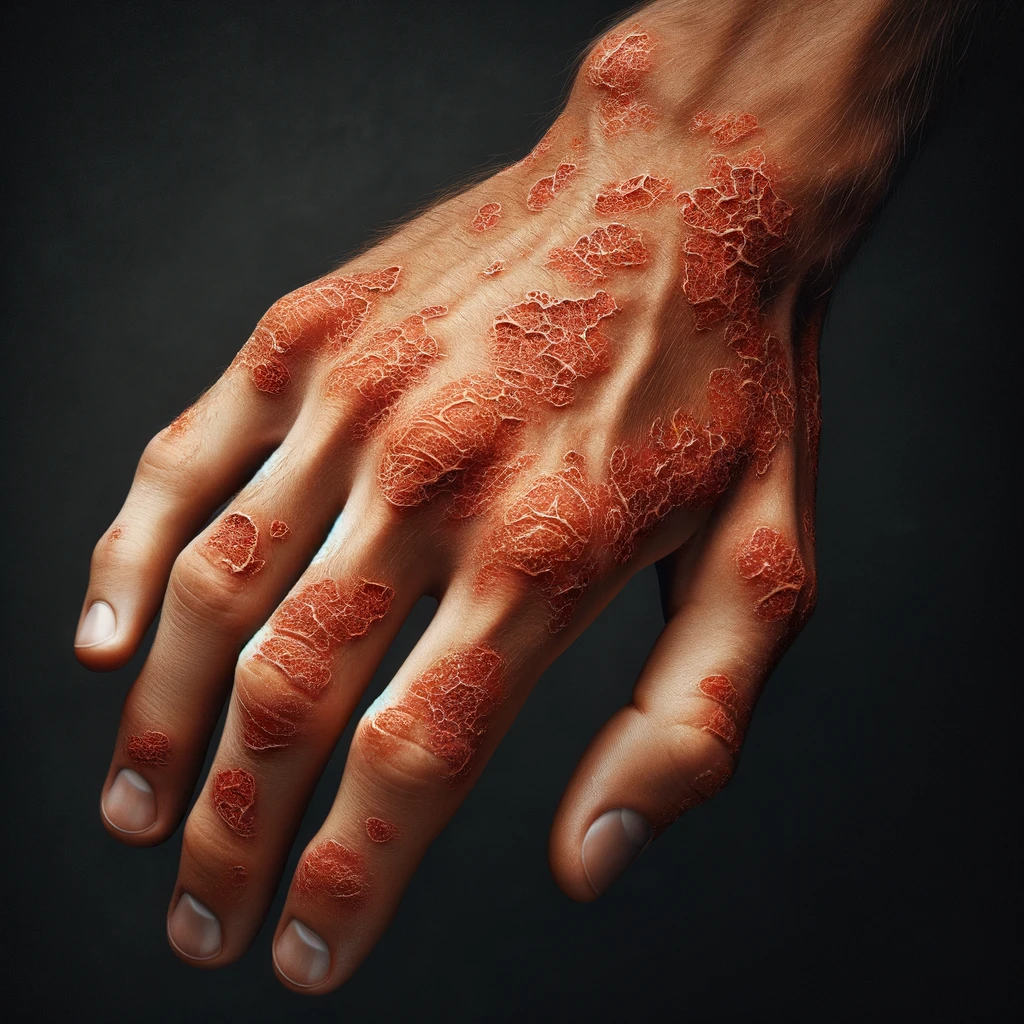

Supplement: Multimedia Appendix 1 [file ai_v3i1e58275_app1.zip › 60 custom GPT.png]

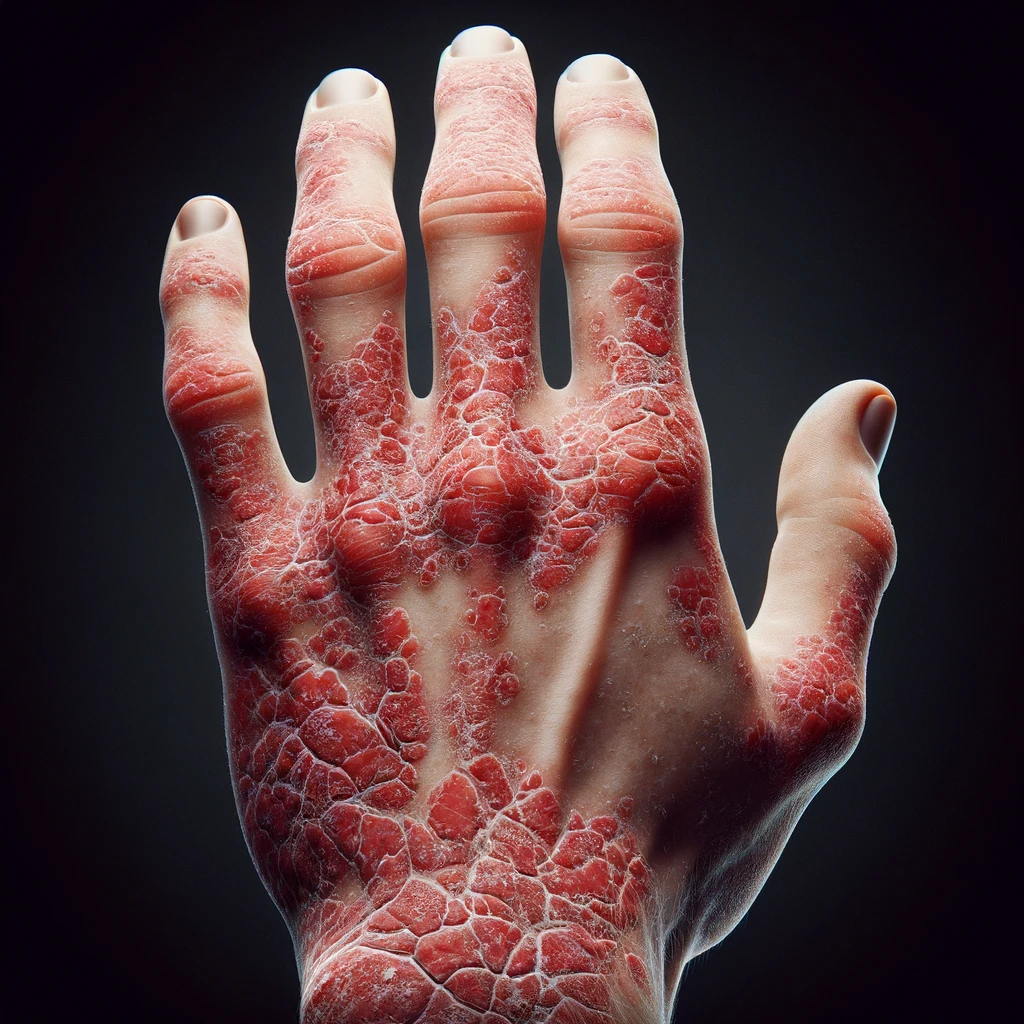

Supplement: Multimedia Appendix 1 [file ai_v3i1e58275_app1.zip › 66 custom GPT.png]

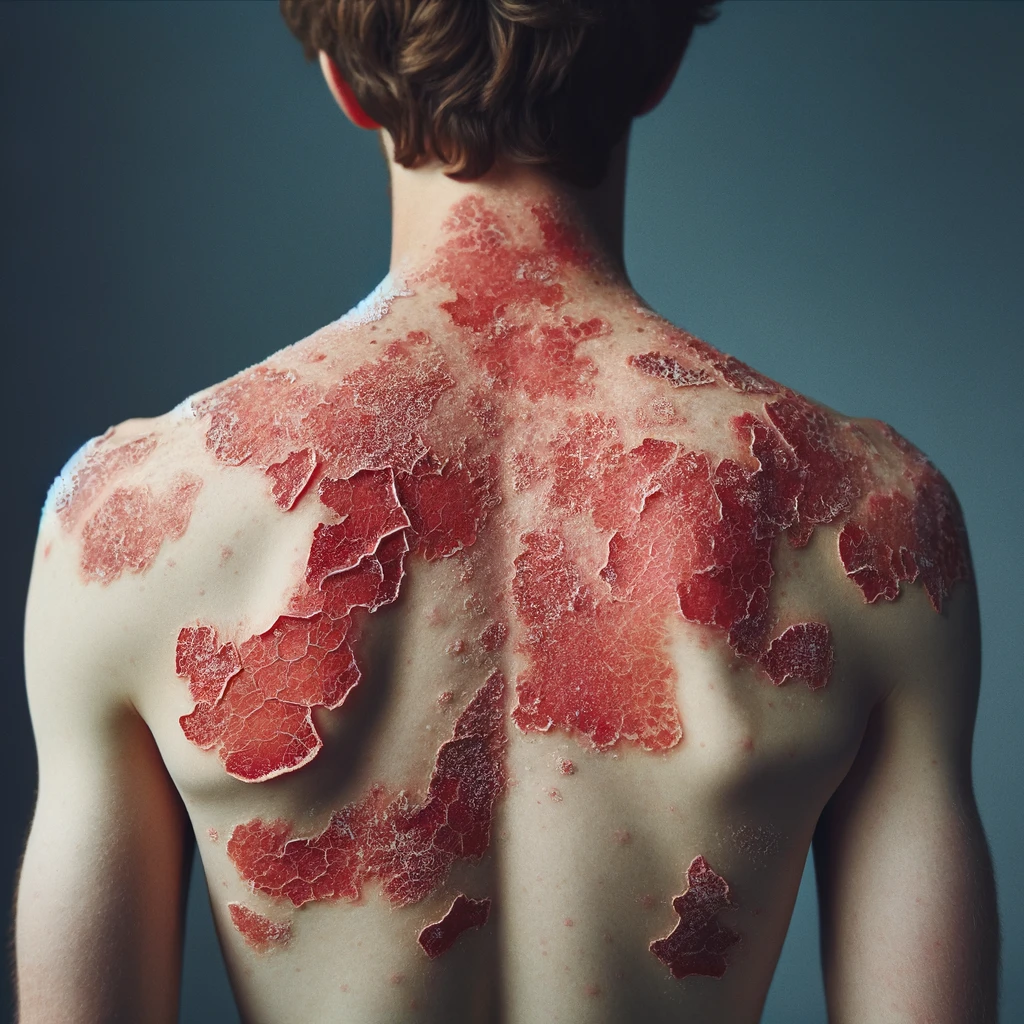

Supplement: Multimedia Appendix 1 [file ai_v3i1e58275_app1.zip › 46 custom GPT.png]

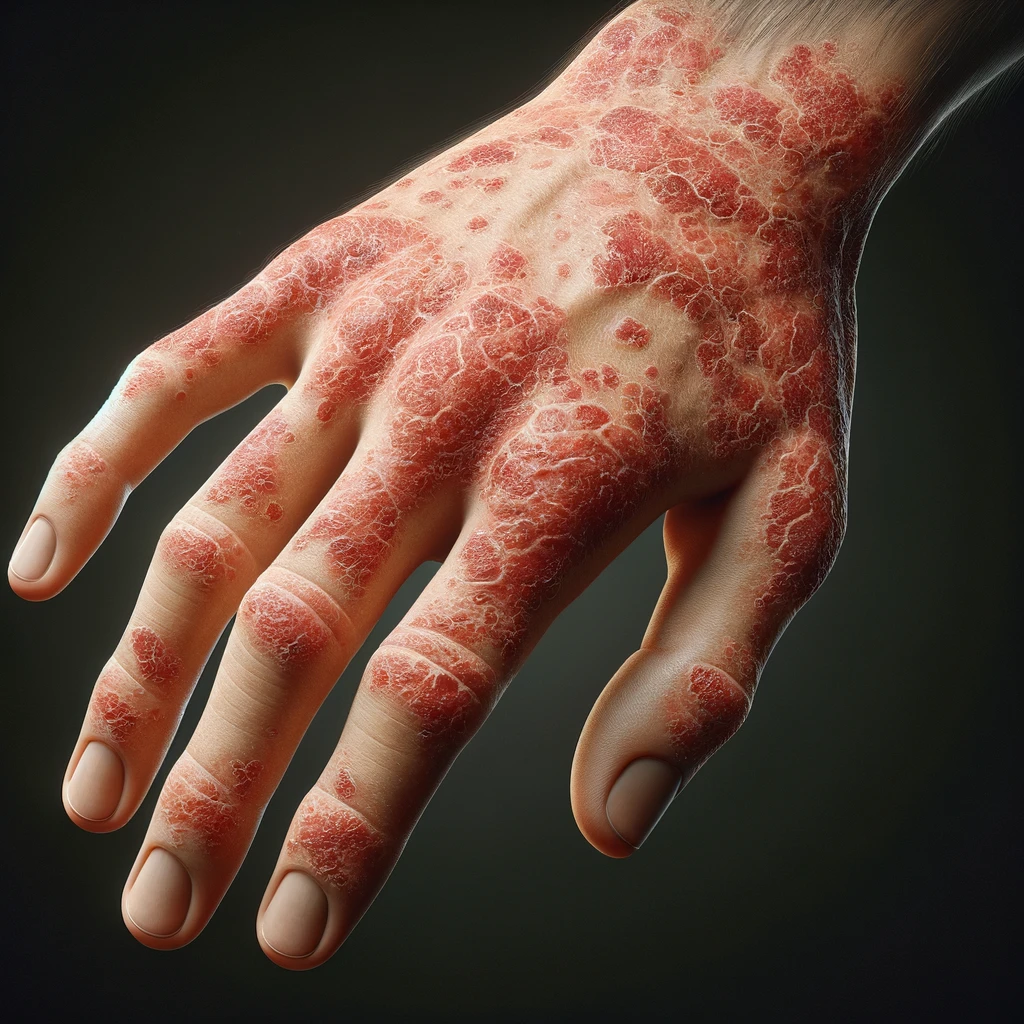

Supplement: Multimedia Appendix 1 [file ai_v3i1e58275_app1.zip › 44 custom GPT.png]

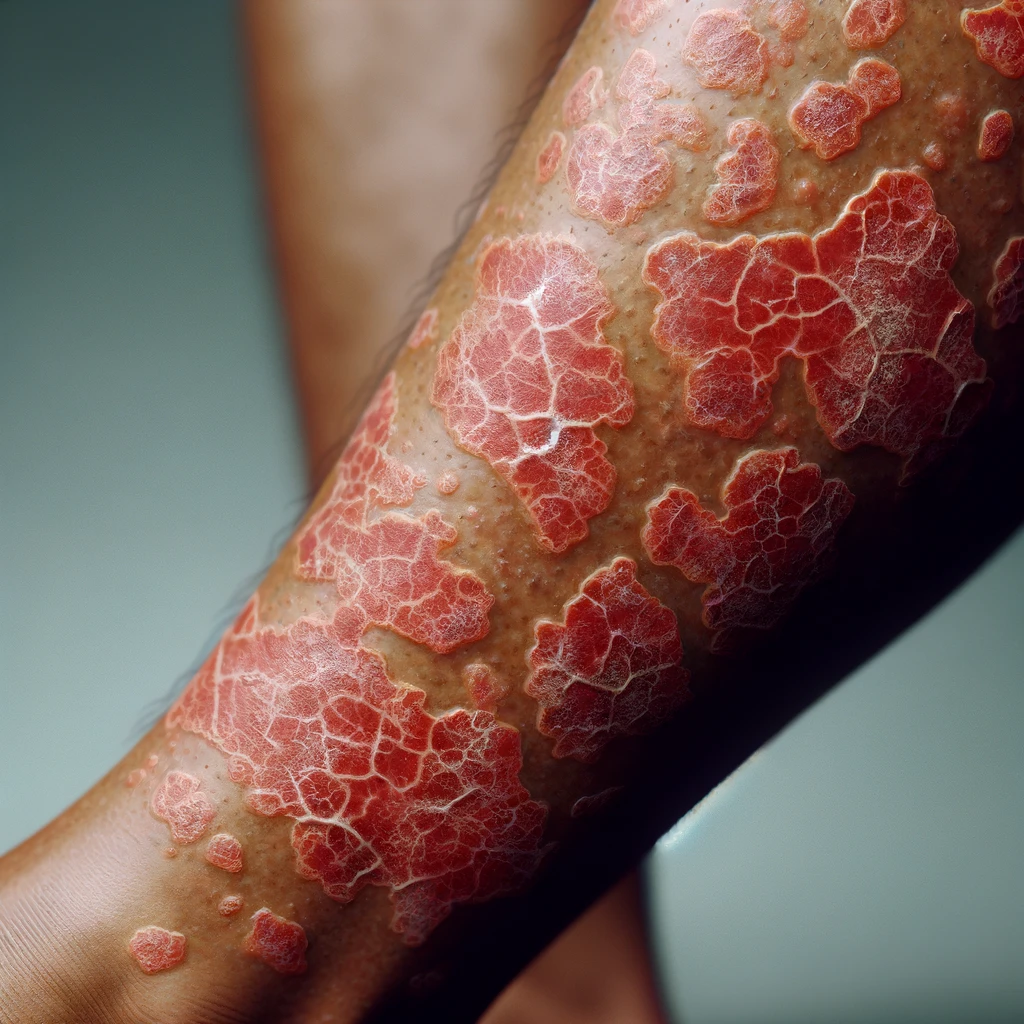

Supplement: Multimedia Appendix 1 [file ai_v3i1e58275_app1.zip › 43 custom GPT.png]

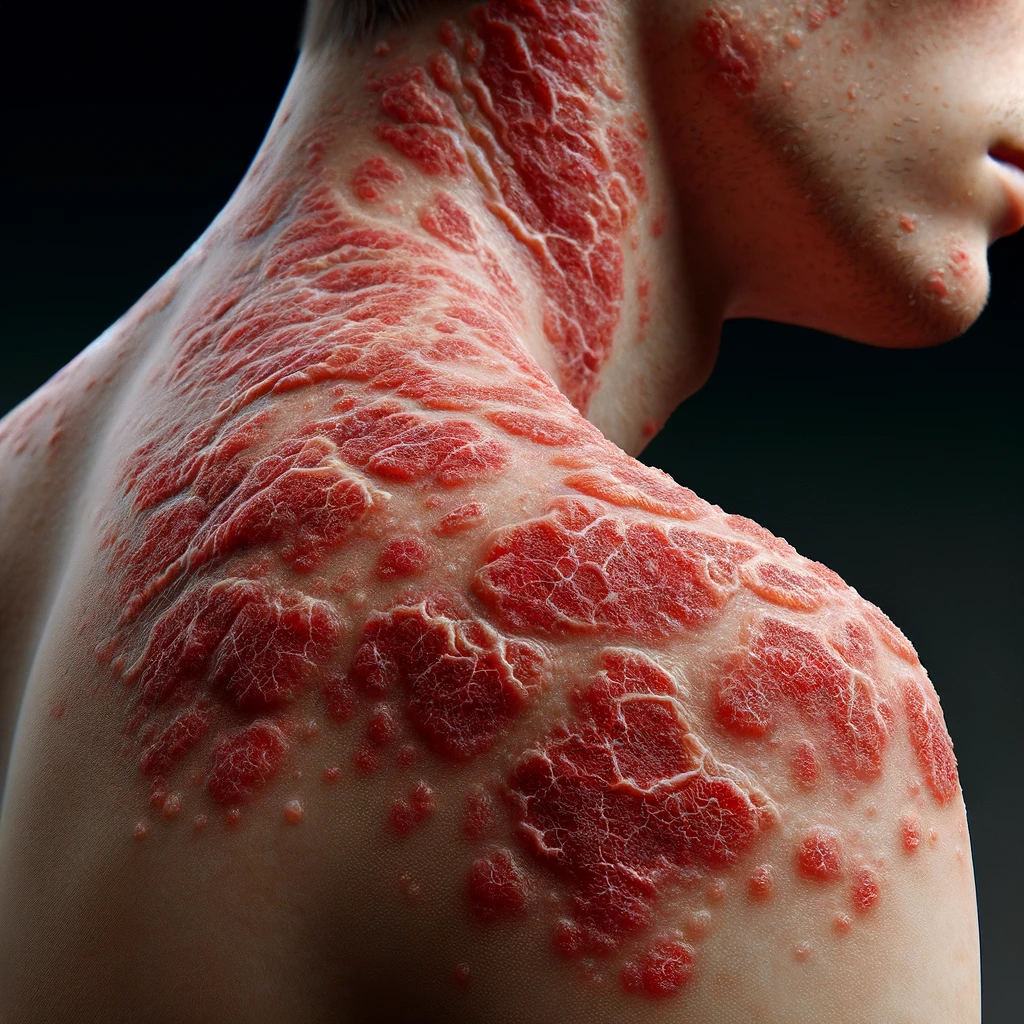

Supplement: Multimedia Appendix 1 [file ai_v3i1e58275_app1.zip › 10 custom GPT.png]

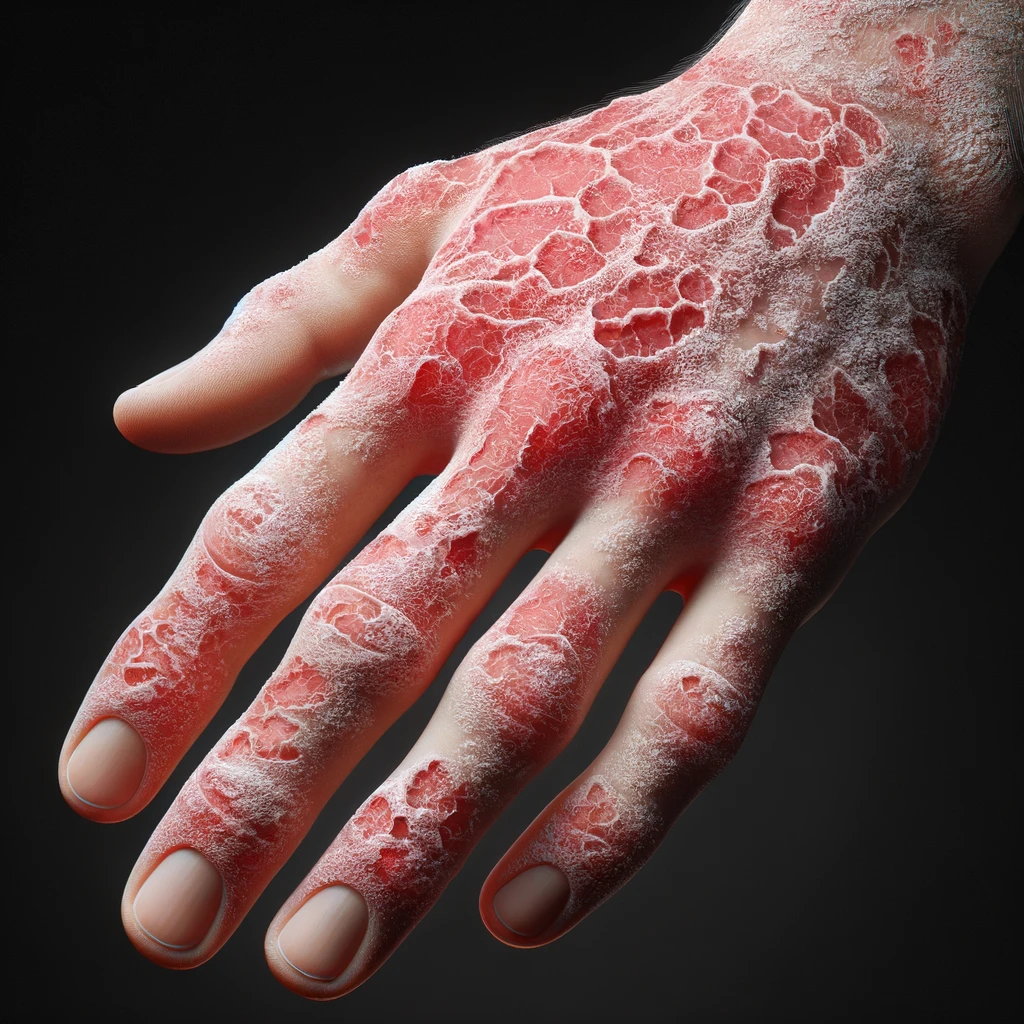

Supplement: Multimedia Appendix 1 [file ai_v3i1e58275_app1.zip › 24 custom GPT.png]

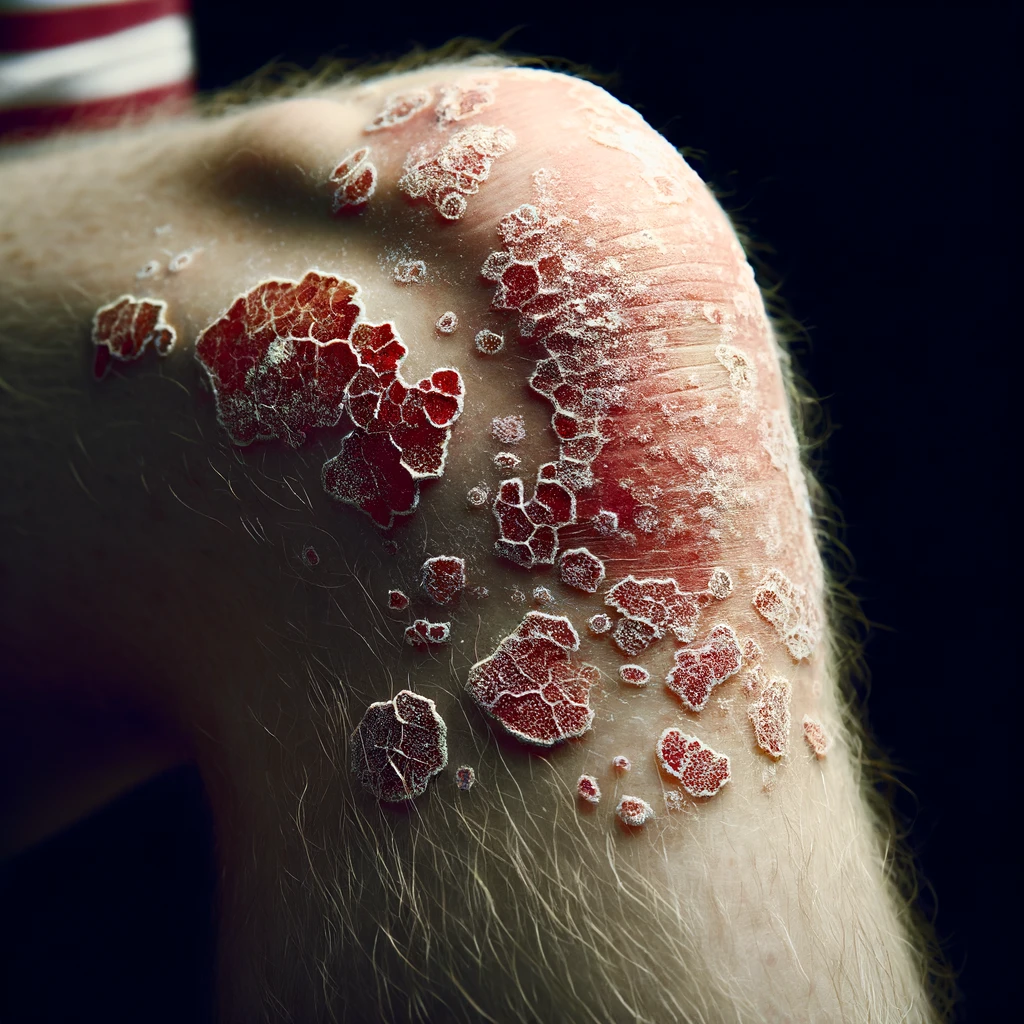

Supplement: Multimedia Appendix 1 [file ai_v3i1e58275_app1.zip › 19 custom GPT.png]

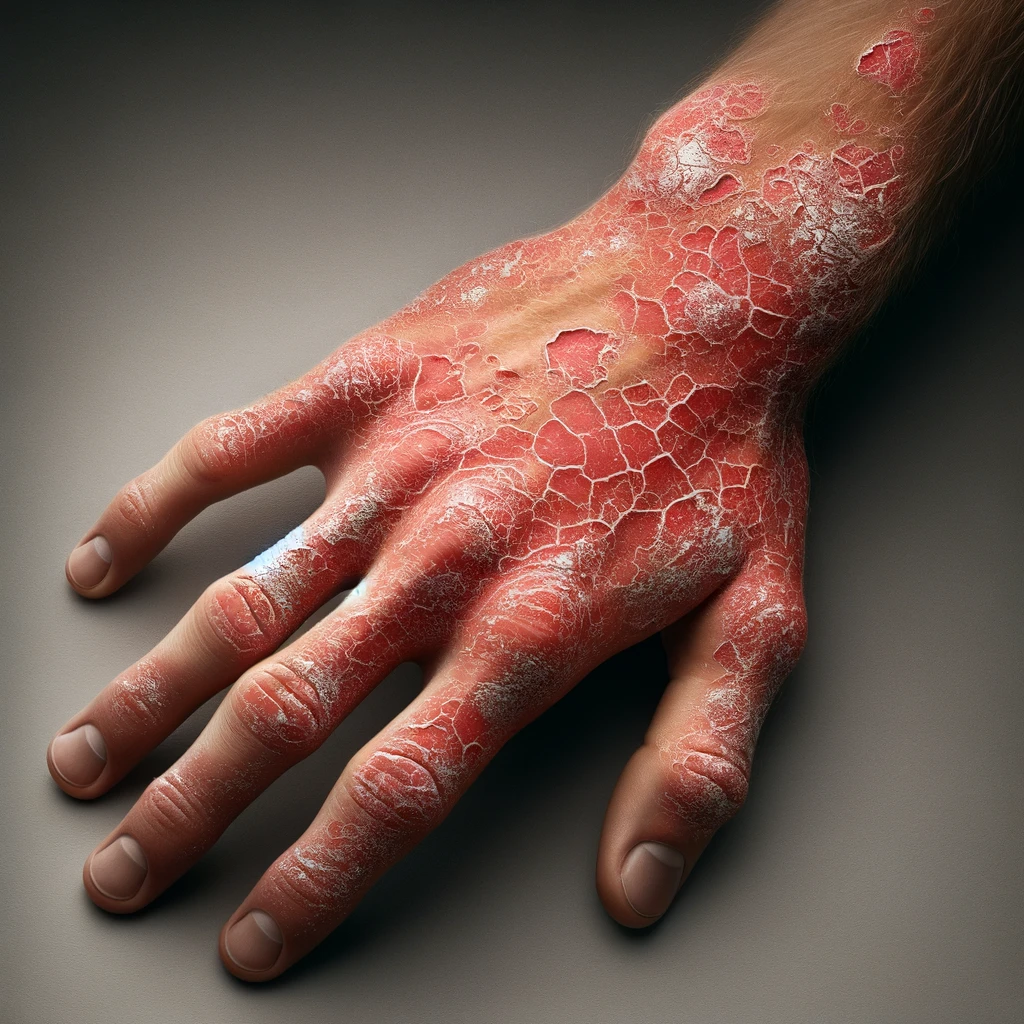

Supplement: Multimedia Appendix 1 [file ai_v3i1e58275_app1.zip › 06 custom GPT.png]

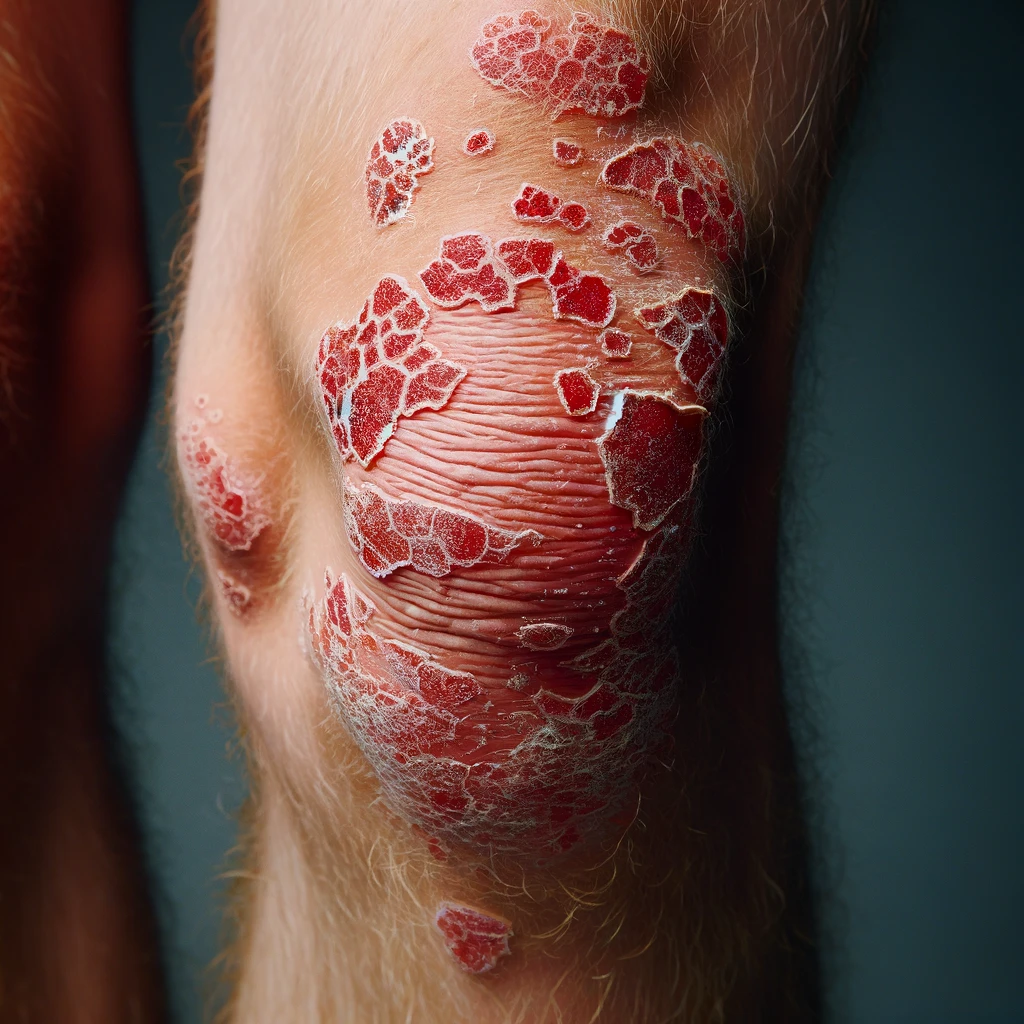

Supplement: Multimedia Appendix 1 [file ai_v3i1e58275_app1.zip › 41 custom GPT.png]

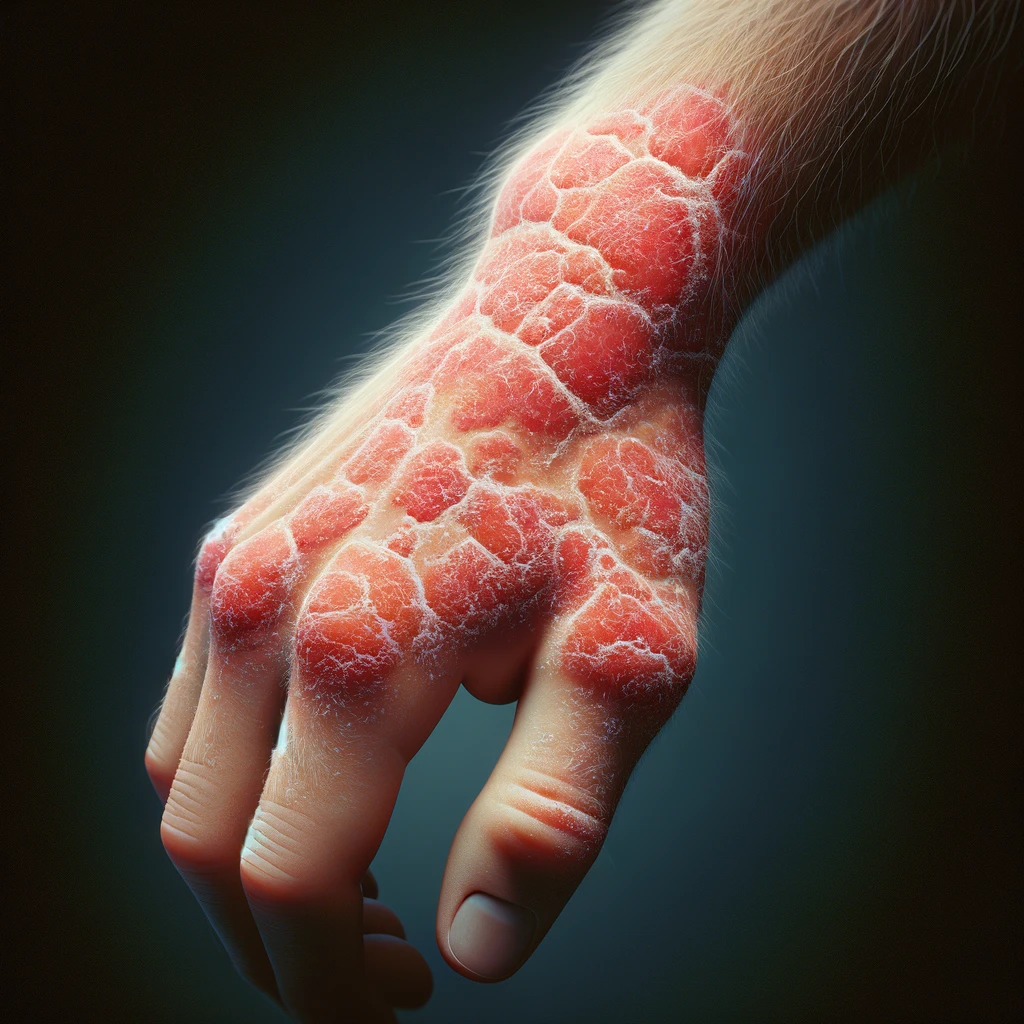

Supplement: Multimedia Appendix 1 [file ai_v3i1e58275_app1.zip › 86 custom GPT.png]

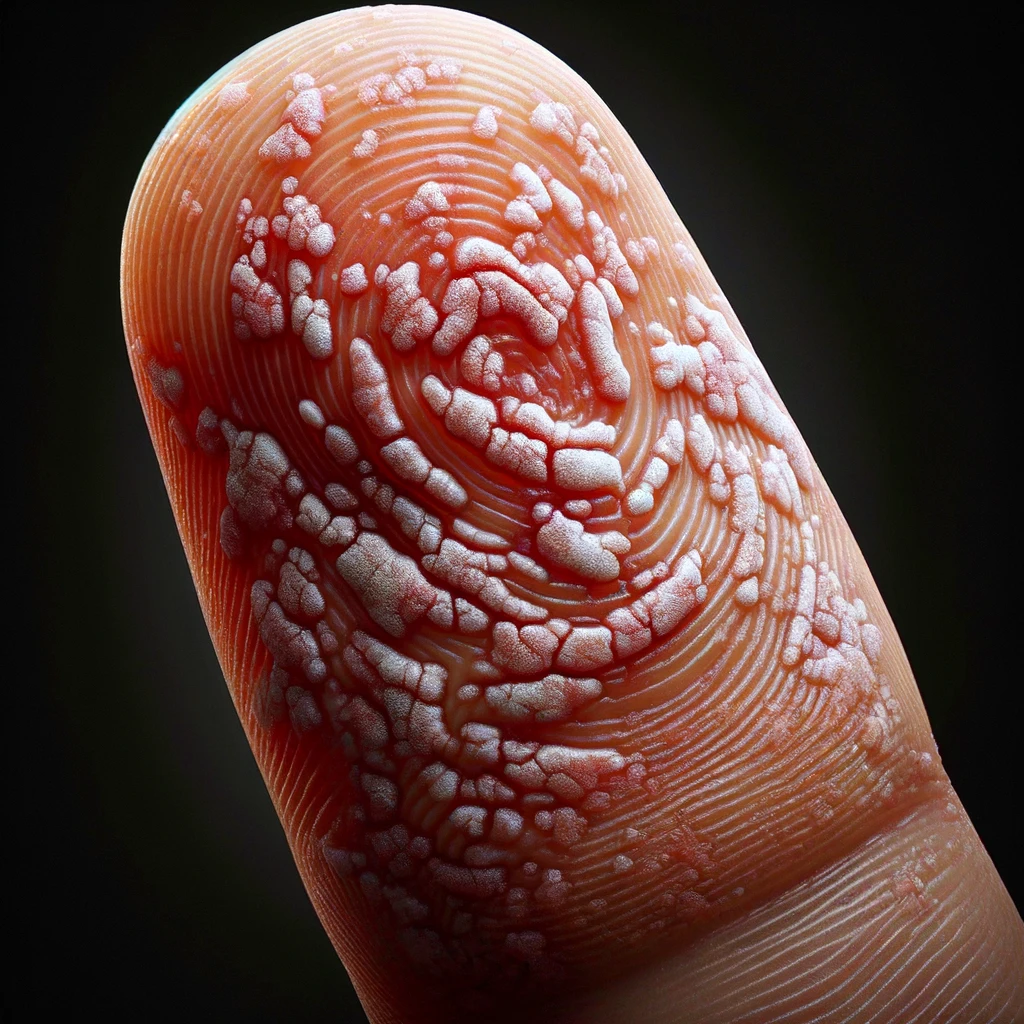

Supplement: Multimedia Appendix 1 [file ai_v3i1e58275_app1.zip › 100 custom GPT.png]

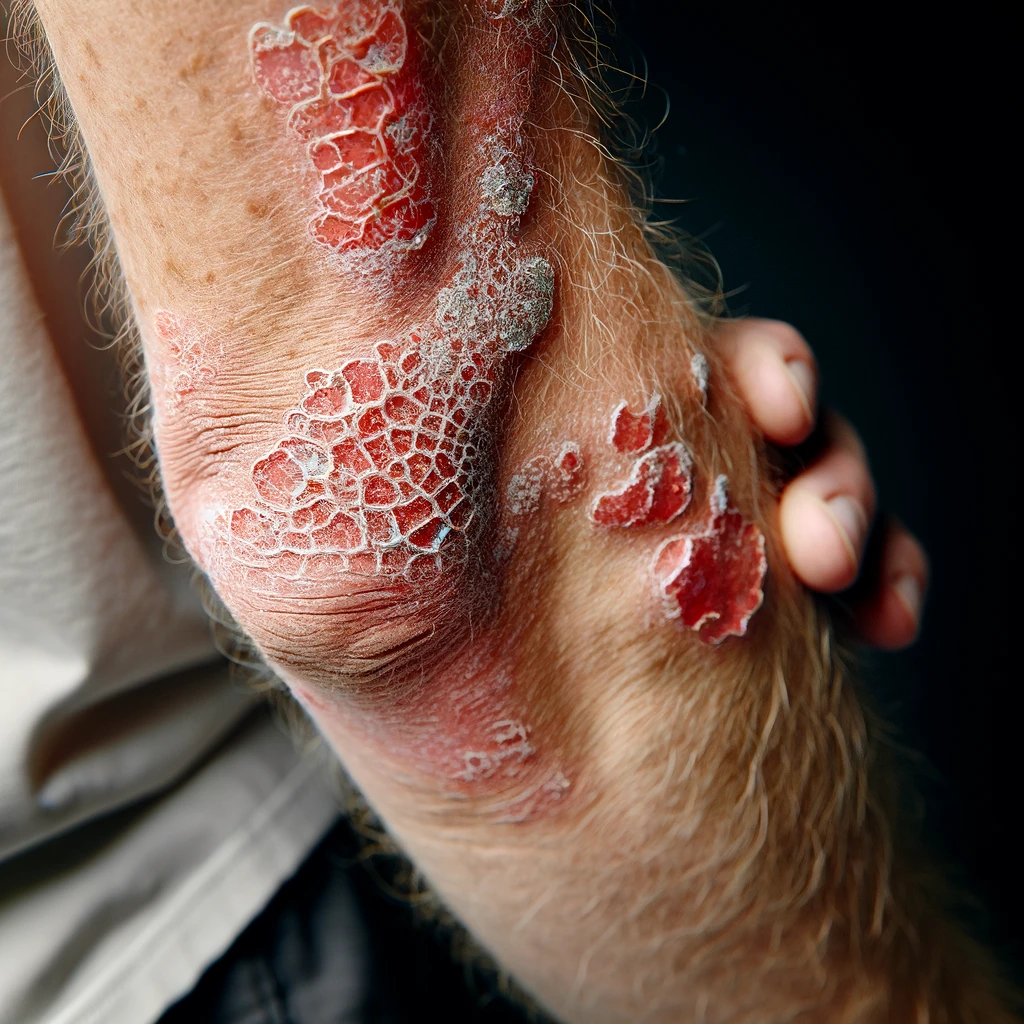

Supplement: Multimedia Appendix 1 [file ai_v3i1e58275_app1.zip › 56 custom GPT.png]

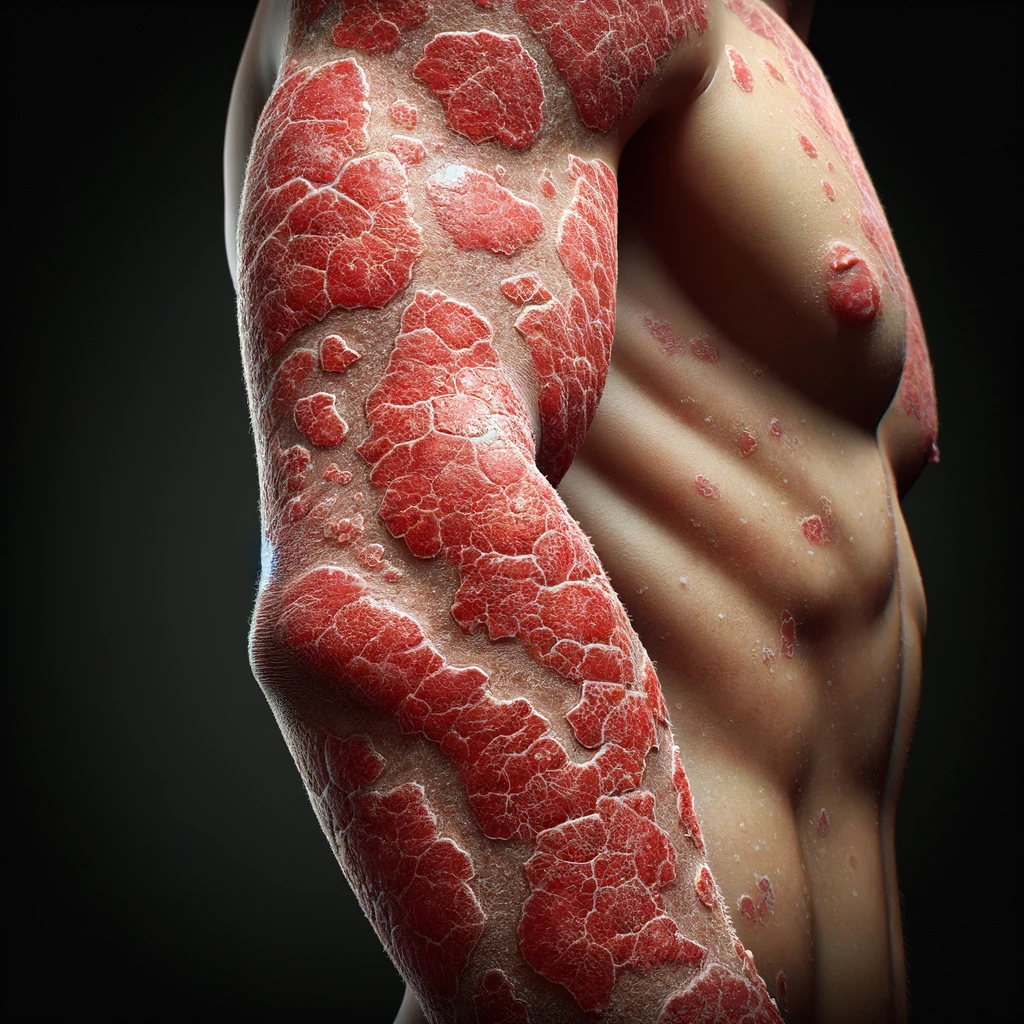

Supplement: Multimedia Appendix 1 [file ai_v3i1e58275_app1.zip › 20 custom GPT.png]

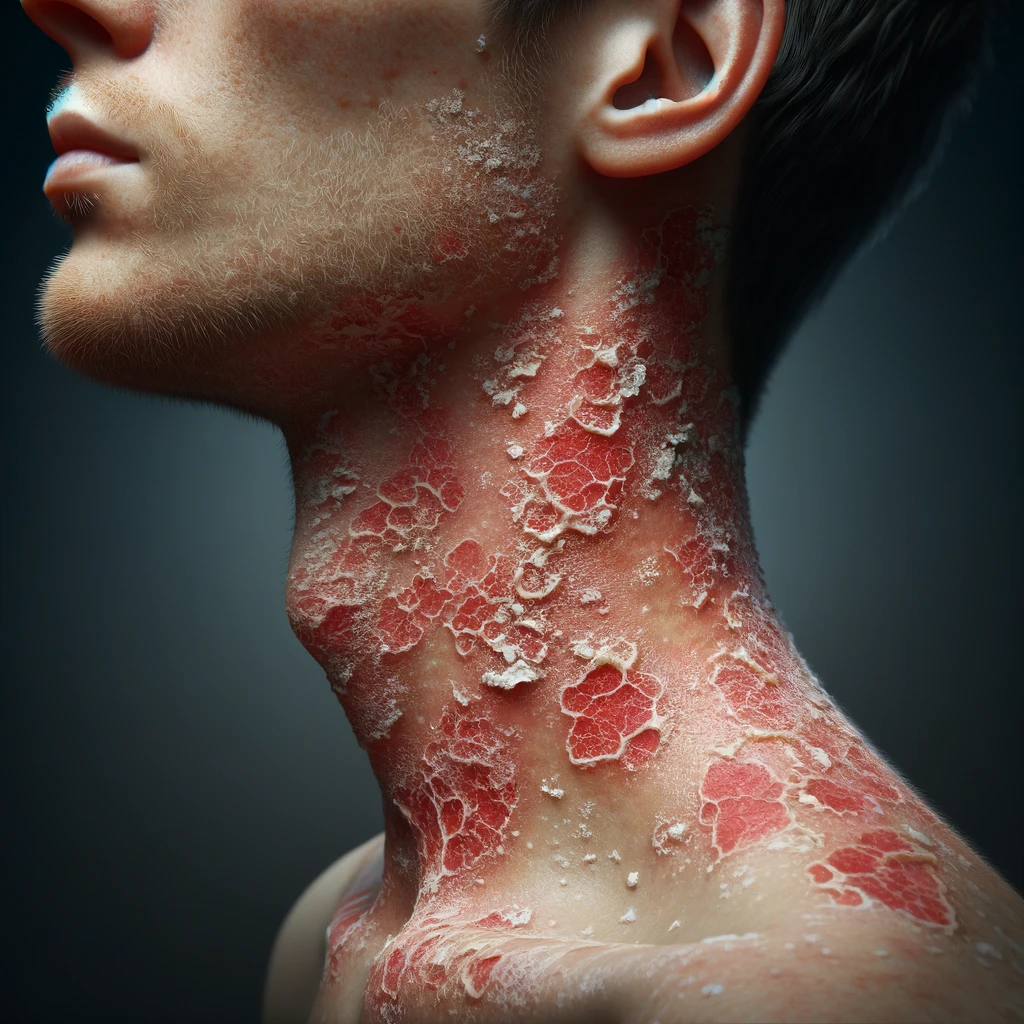

Supplement: Multimedia Appendix 1 [file ai_v3i1e58275_app1.zip › 83 custom GPT.png]

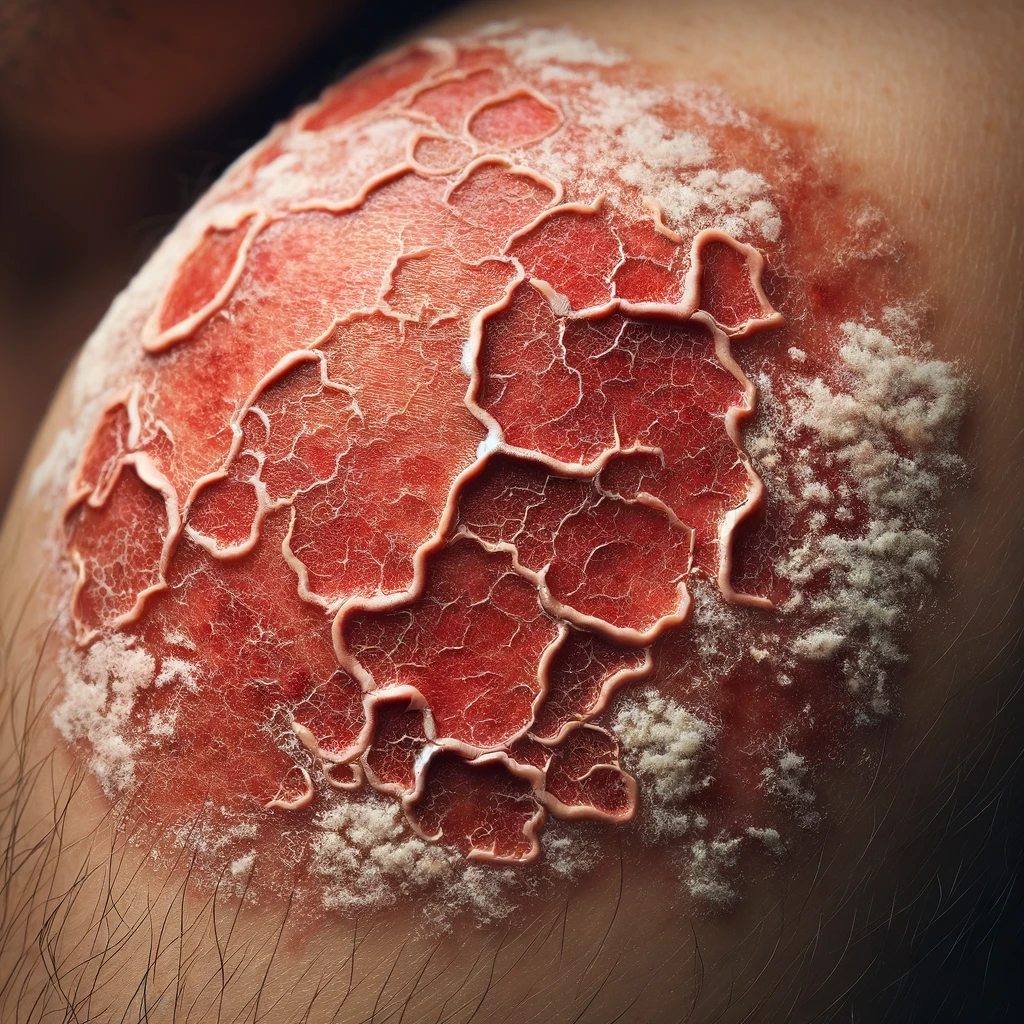

Supplement: Multimedia Appendix 1 [file ai_v3i1e58275_app1.zip › 50 custom GPT.png]

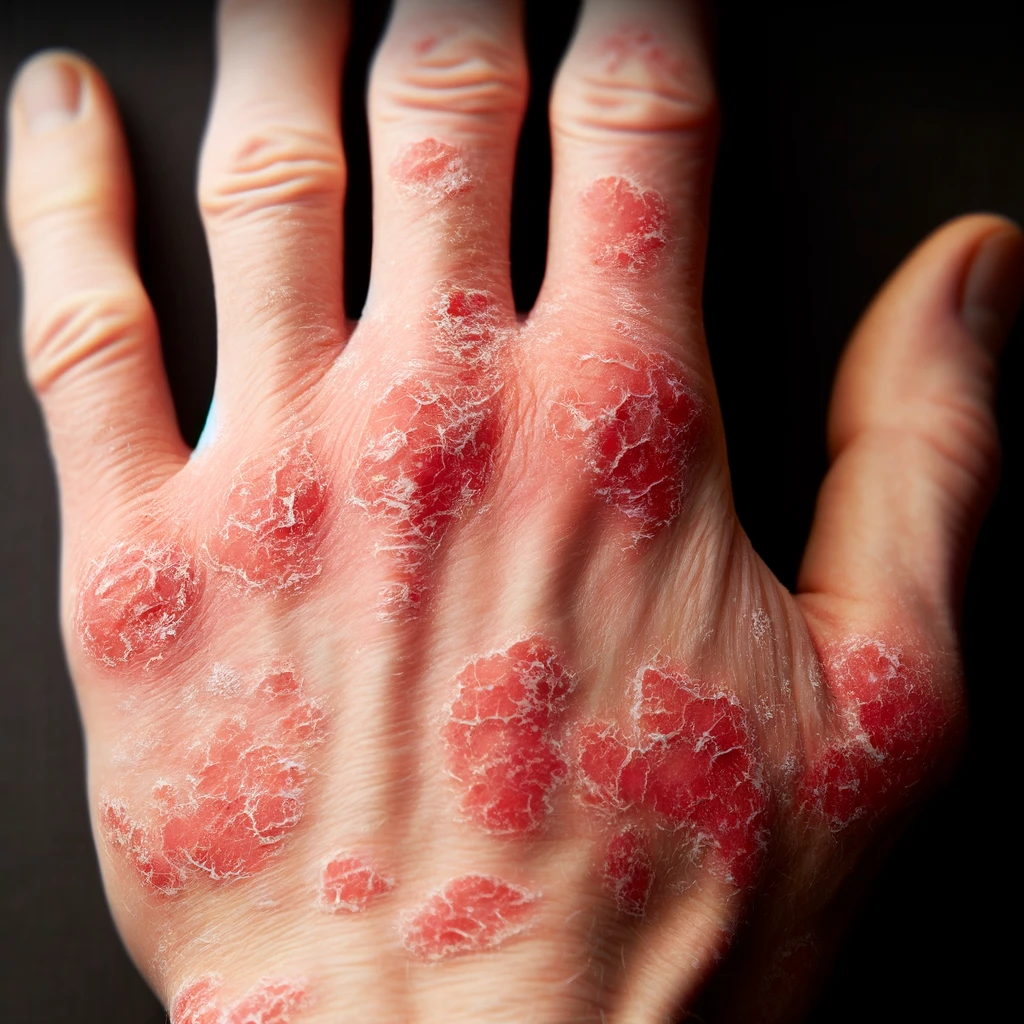

Supplement: Multimedia Appendix 1 [file ai_v3i1e58275_app1.zip › 25 custom GPT.png]

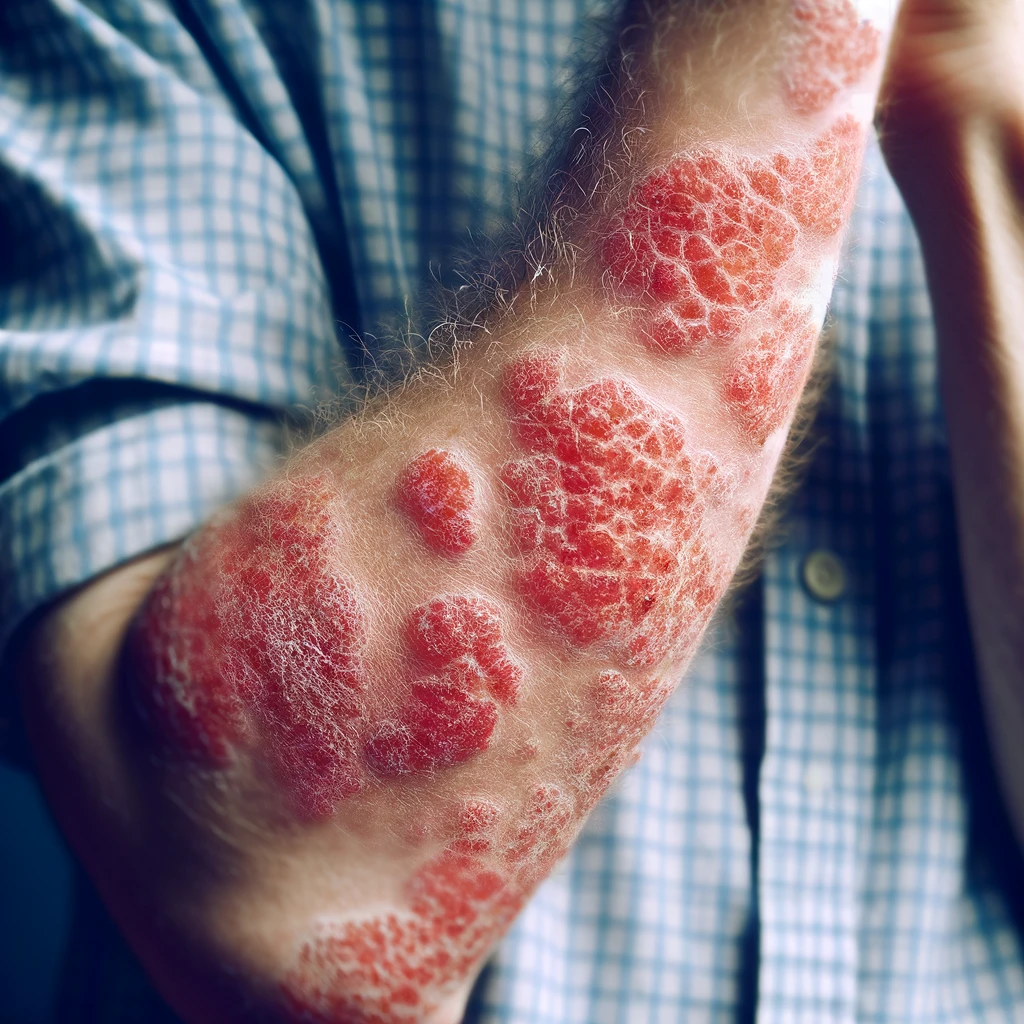

Supplement: Multimedia Appendix 1 [file ai_v3i1e58275_app1.zip › 04 custom GPT.png]

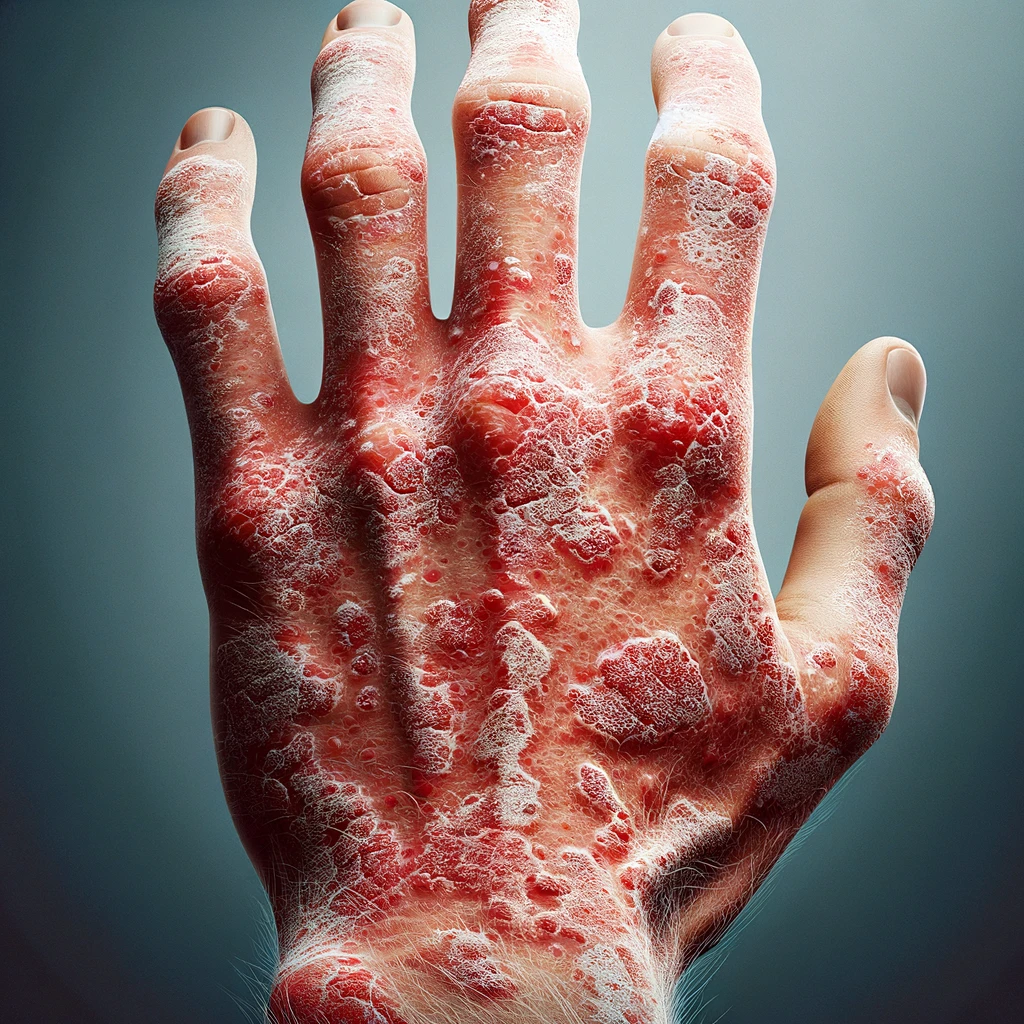

Supplement: Multimedia Appendix 1 [file ai_v3i1e58275_app1.zip › 97 custom GPT.png]

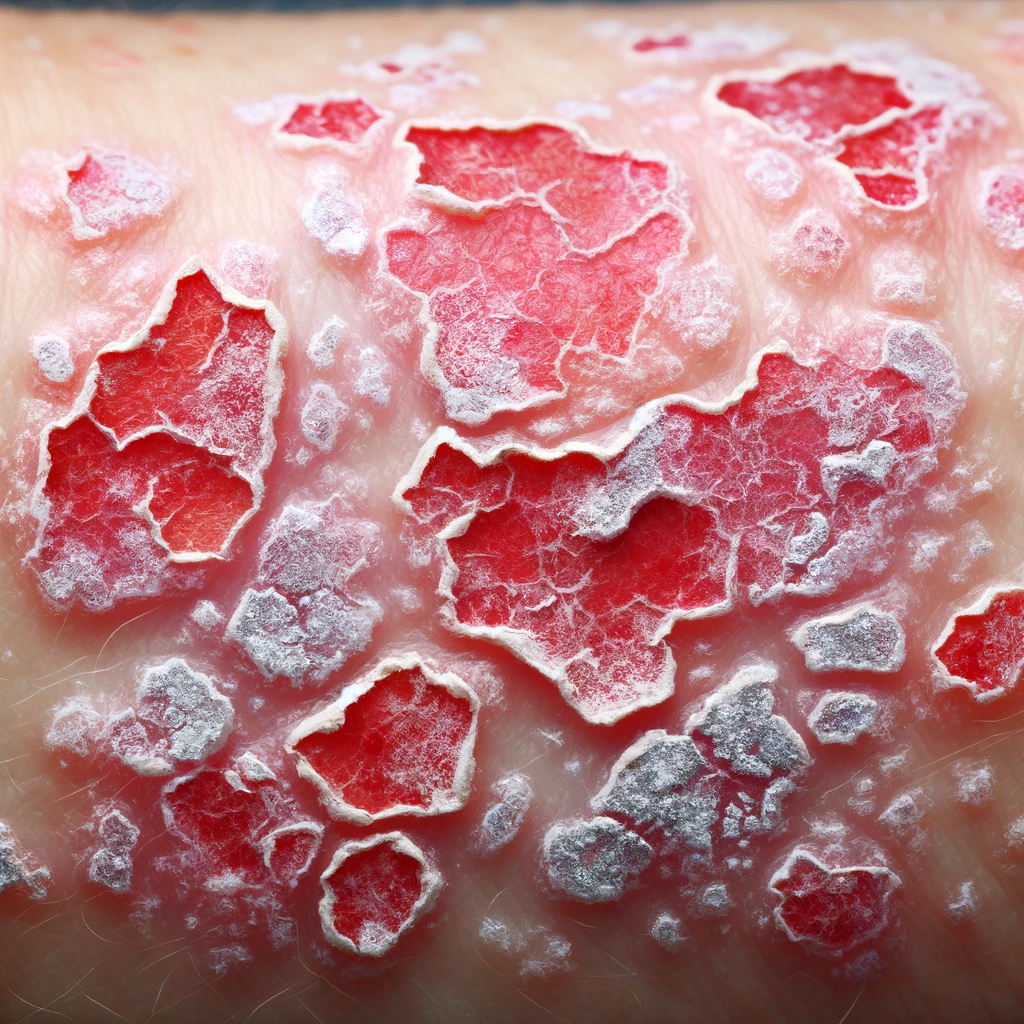

Supplement: Multimedia Appendix 1 [file ai_v3i1e58275_app1.zip › 71 custom GPT.png]

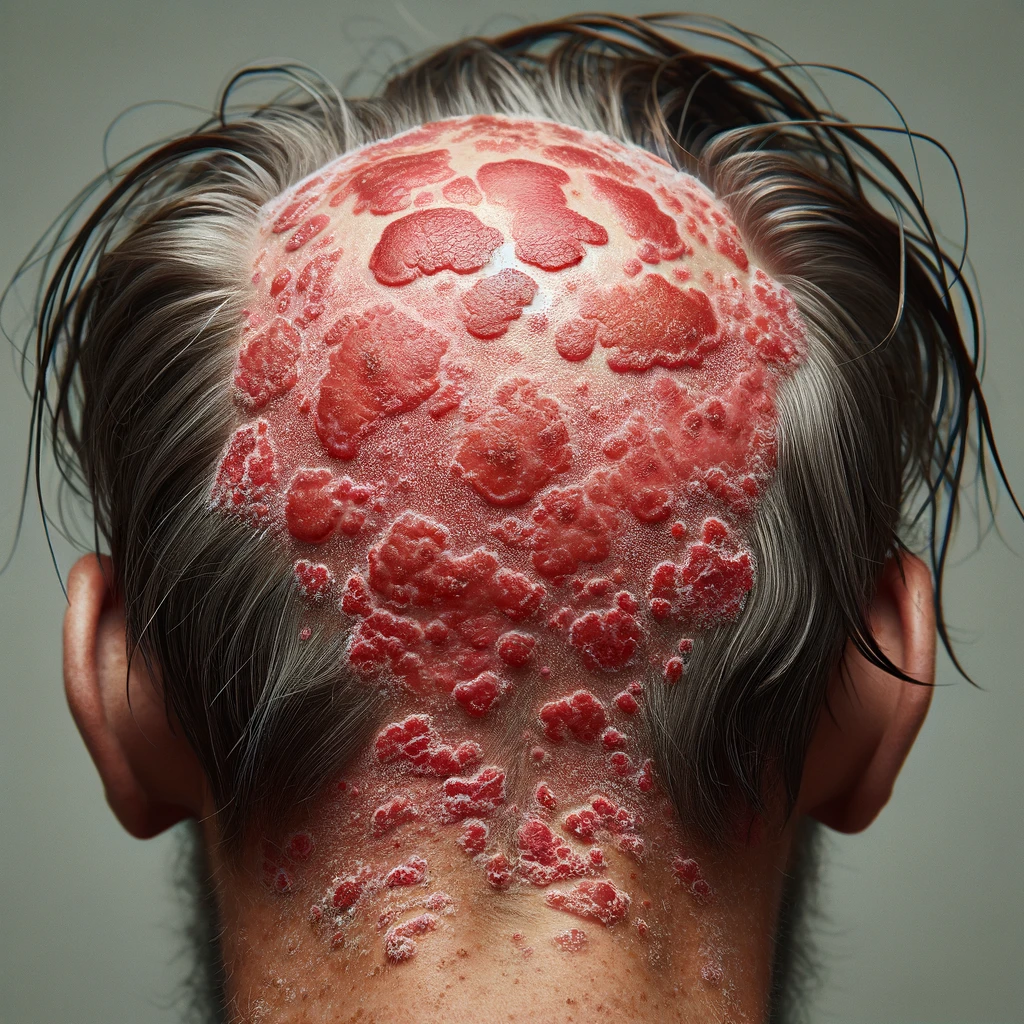

Supplement: Multimedia Appendix 1 [file ai_v3i1e58275_app1.zip › 75 custom GPT.png]
