# Supplementary figures and images for: Ensuring Appropriate Representation in Artificial Intelligence–Generated Medical Imagery: Protocol for a Methodological Approach to Address Skin Tone Bias (part 2 of 3)
Source: JMIR AI. 2024 Nov 27;3:e58275. doi: 10.2196/58275 (PMC11635324; doi:10.2196/58275)

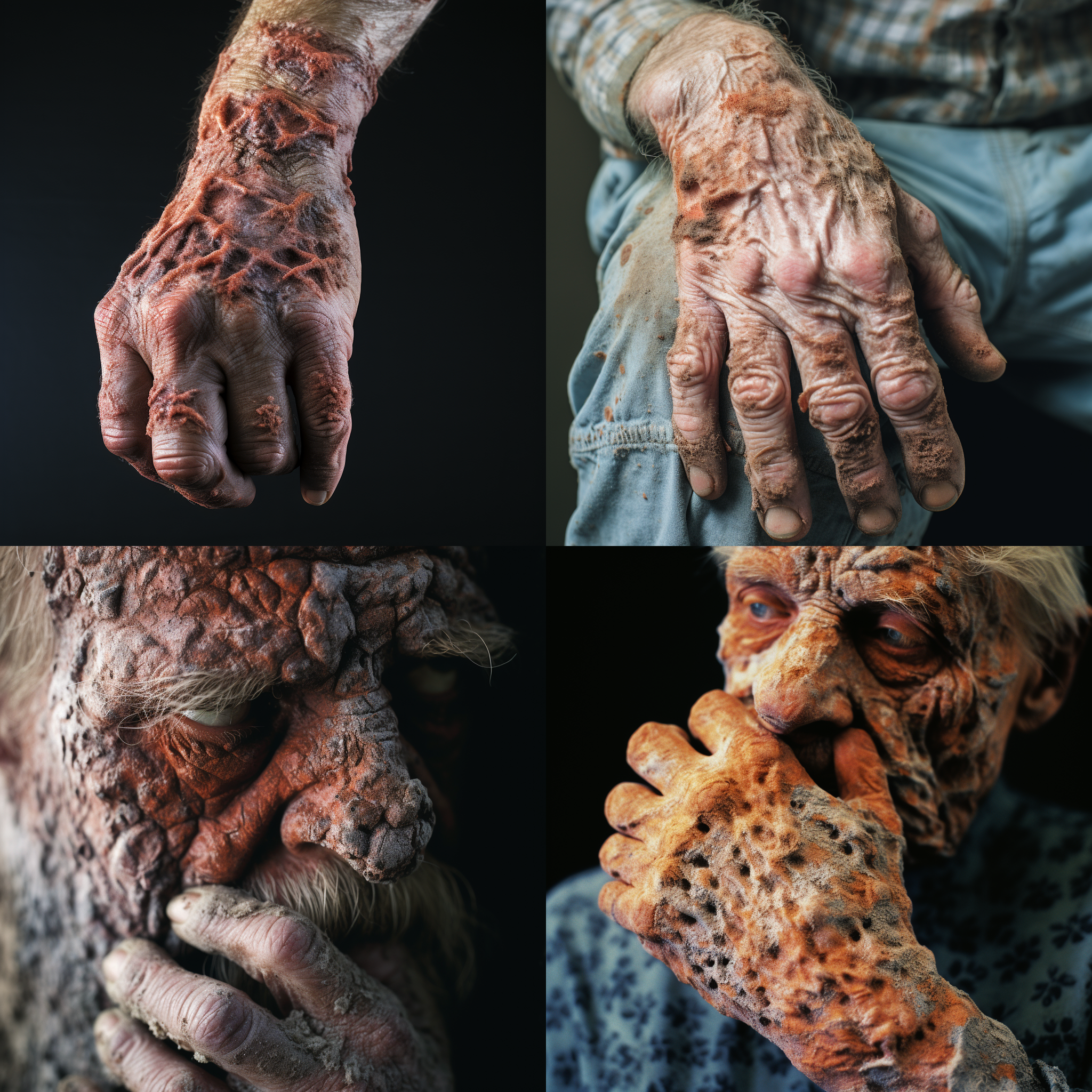

Supplement: Multimedia Appendix 2 [file ai_v3i1e58275_app2.zip › 25.andrewo999_a_photograph_showing_an_example_of_psoriasis_c94162cd-30da-4eac-b834-3443cf8b5410.PNG]

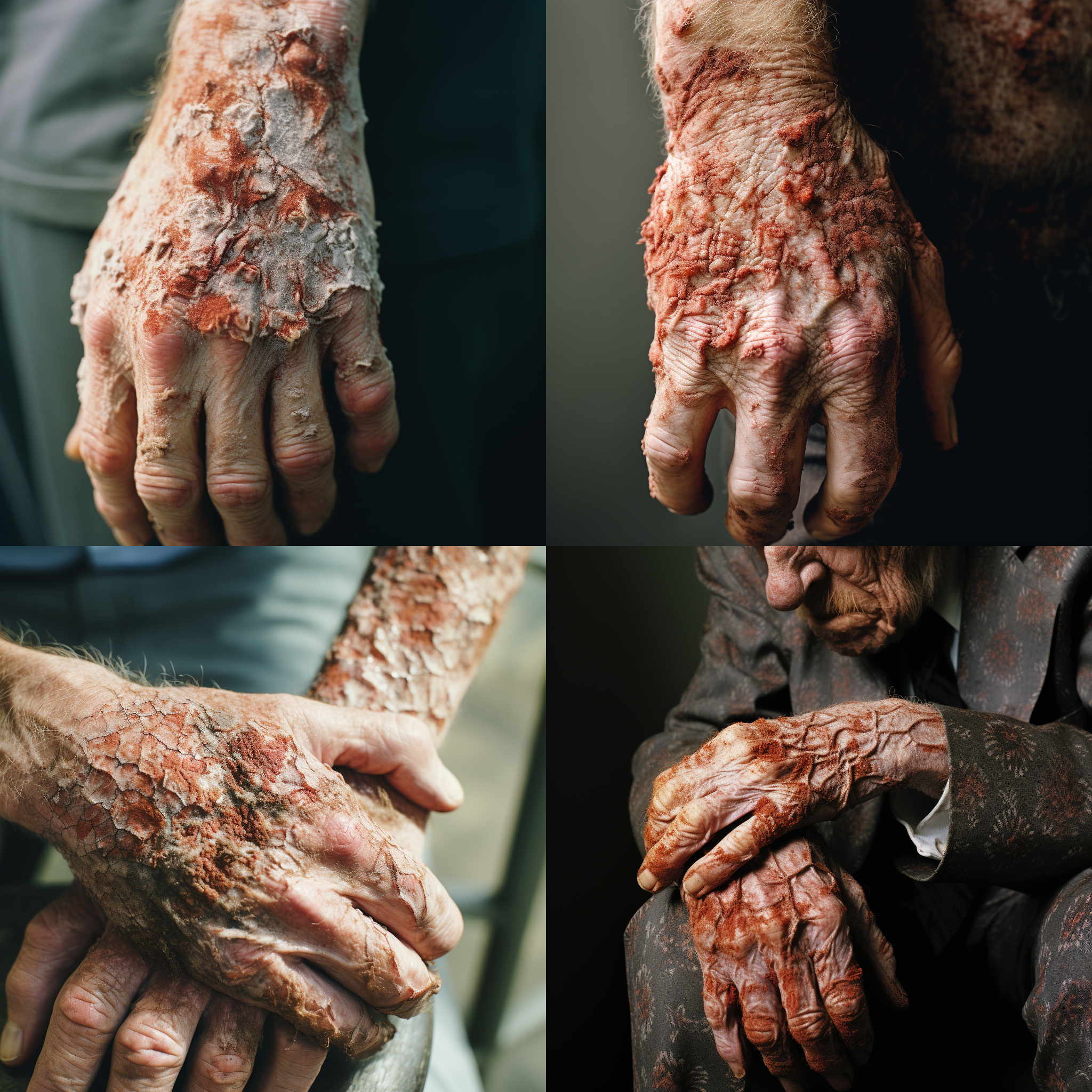

Supplement: Multimedia Appendix 2 [file ai_v3i1e58275_app2.zip › 05.andrewo999_a_photograph_showing_an_example_of_psoriasis_35836280-eb45-40d6-98e2-fcfb0b27a3e8.PNG]

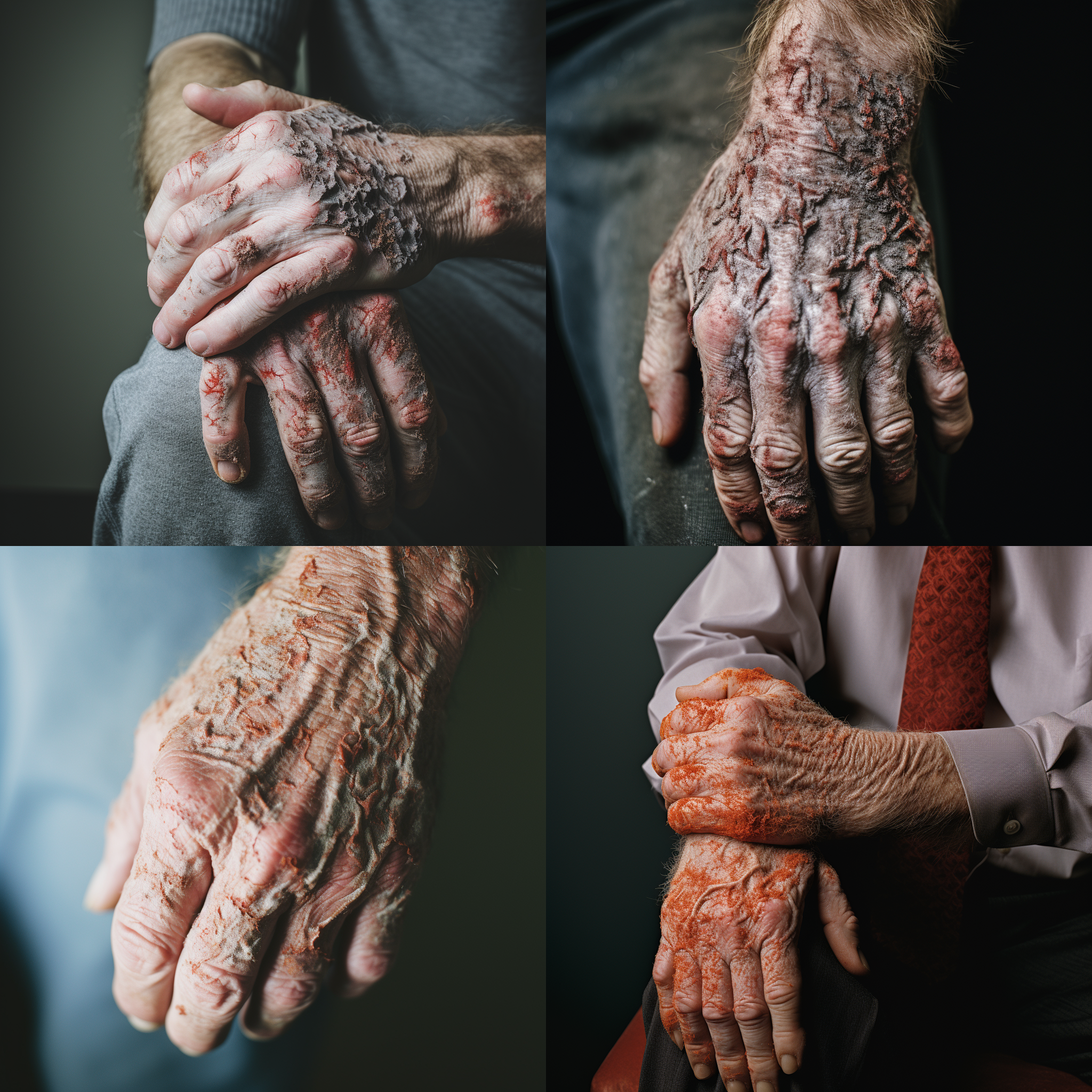

Supplement: Multimedia Appendix 2 [file ai_v3i1e58275_app2.zip › 10.andrewo999_a_photograph_showing_an_example_of_psoriasis_5ac7ceca-20bf-455c-b4a8-107473ffe4e3.PNG]

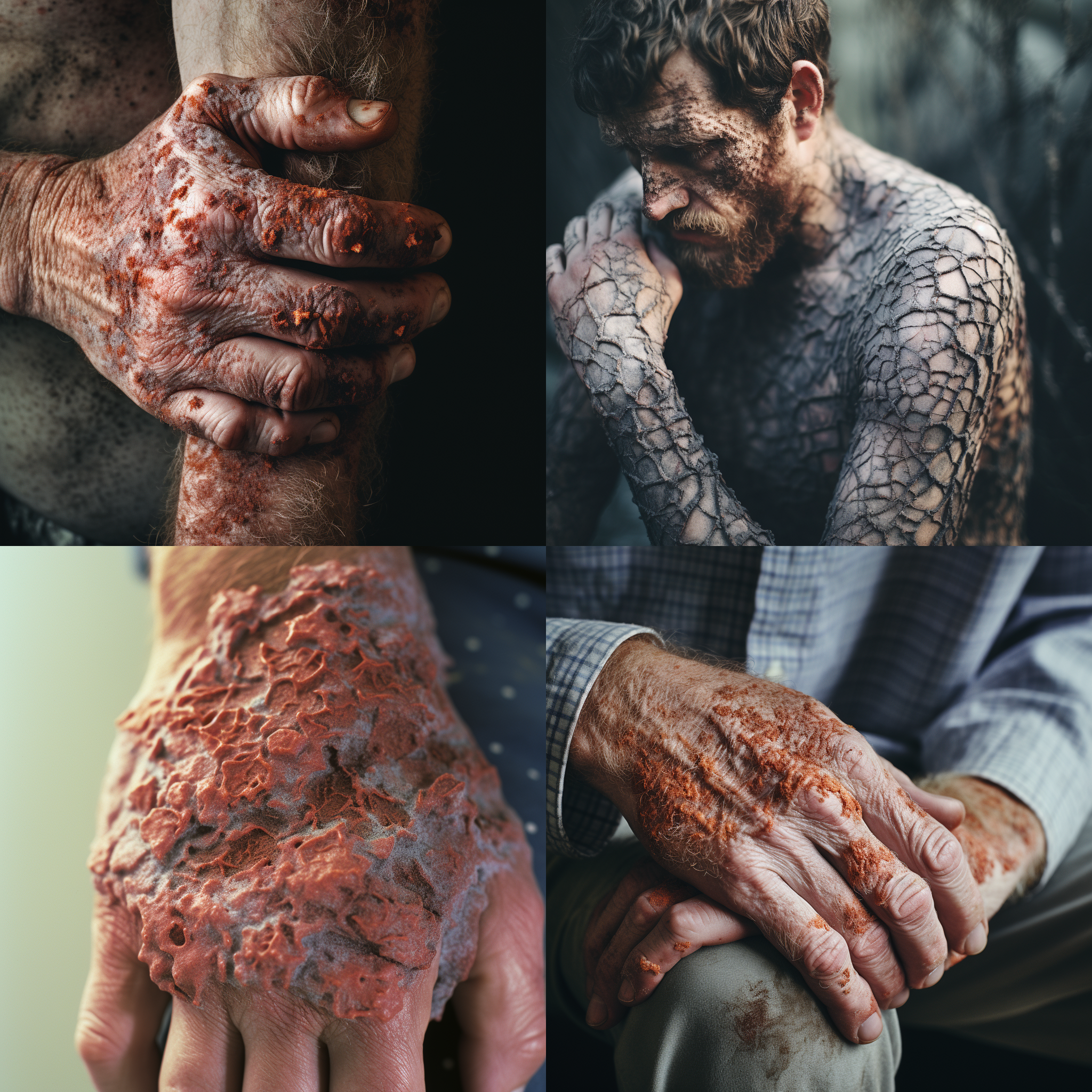

Supplement: Multimedia Appendix 2 [file ai_v3i1e58275_app2.zip › 24.andrewo999_a_photograph_showing_an_example_of_psoriasis_c8654a22-6ece-472e-afd7-a6c83960d4a3.PNG]

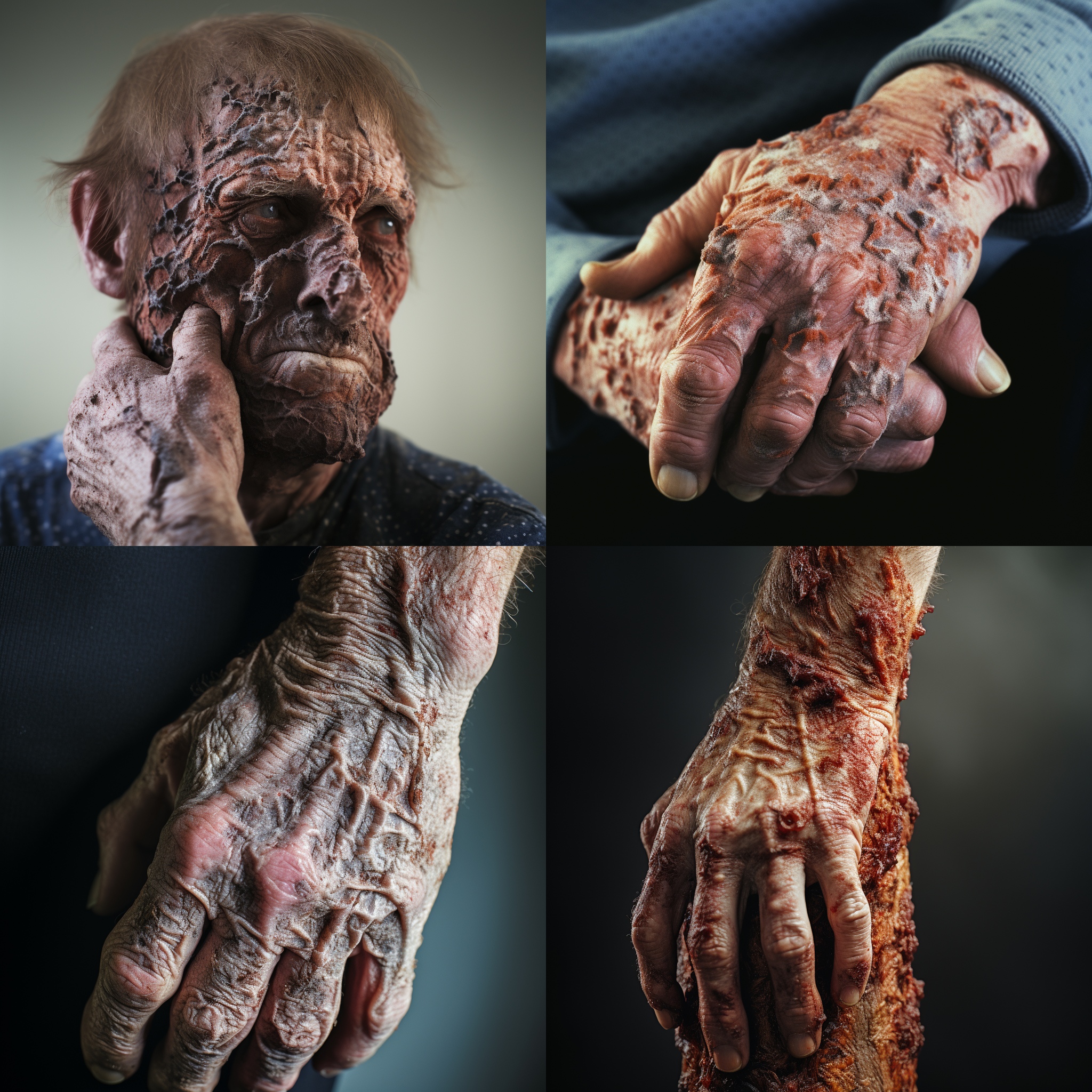

Supplement: Multimedia Appendix 2 [file ai_v3i1e58275_app2.zip › 21.andrewo999_a_photograph_showing_an_example_of_psoriasis_ab7db12b-77fd-44e4-bb90-8d644afa5c33.PNG]

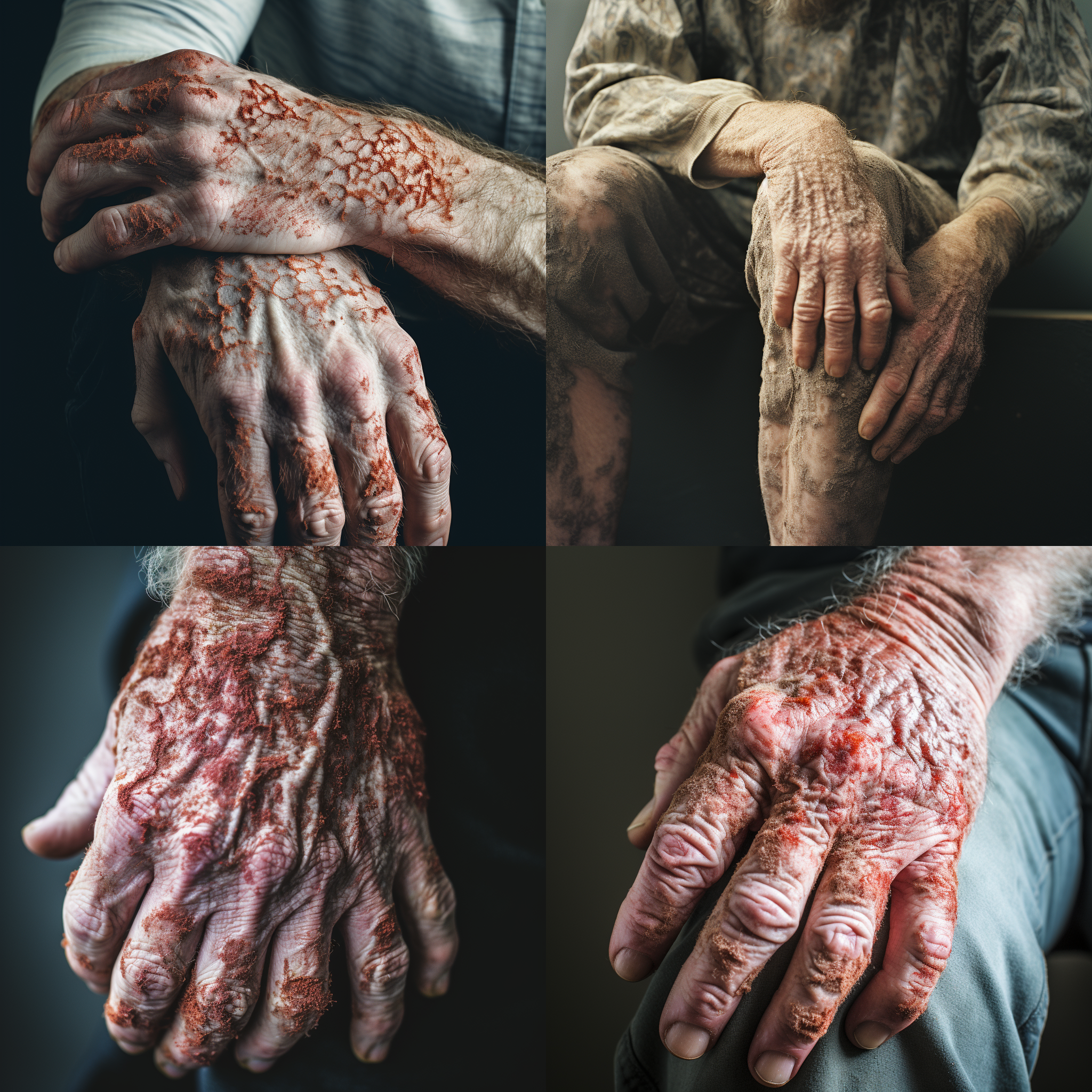

Supplement: Multimedia Appendix 2 [file ai_v3i1e58275_app2.zip › 22.andrewo999_a_photograph_showing_an_example_of_psoriasis_b2ceeb86-013c-46a5-a51c-27c68ccc0a88.PNG]

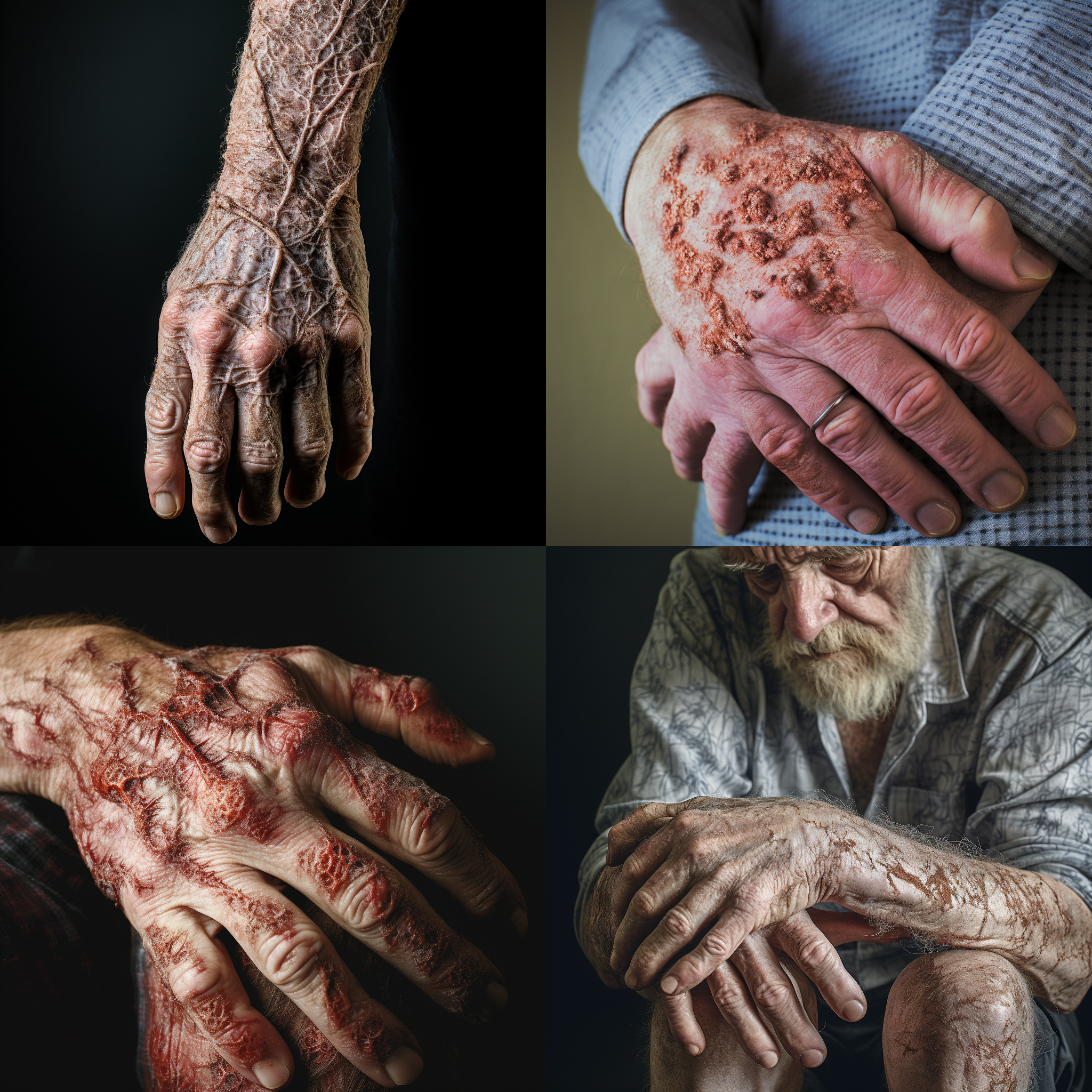

Supplement: Multimedia Appendix 2 [file ai_v3i1e58275_app2.zip › 01.andrewo999_a_photograph_showing_an_example_of_psoriasis_0361d45a-815a-47cb-96b2-28fa38d1a15b.PNG]

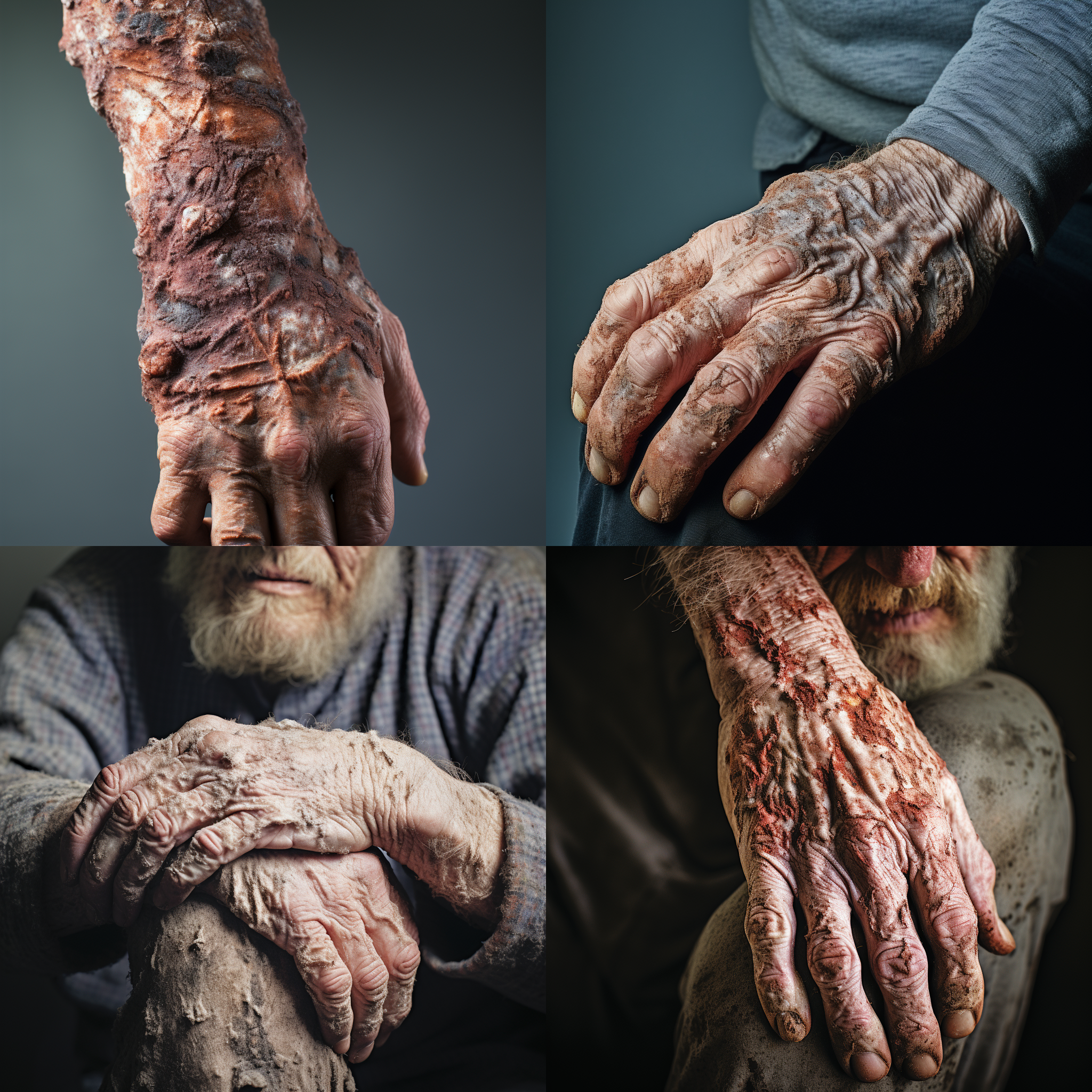

Supplement: Multimedia Appendix 2 [file ai_v3i1e58275_app2.zip › 13.andrewo999_a_photograph_showing_an_example_of_psoriasis_65546094-4824-4628-a722-dcb8ea52d277.PNG]

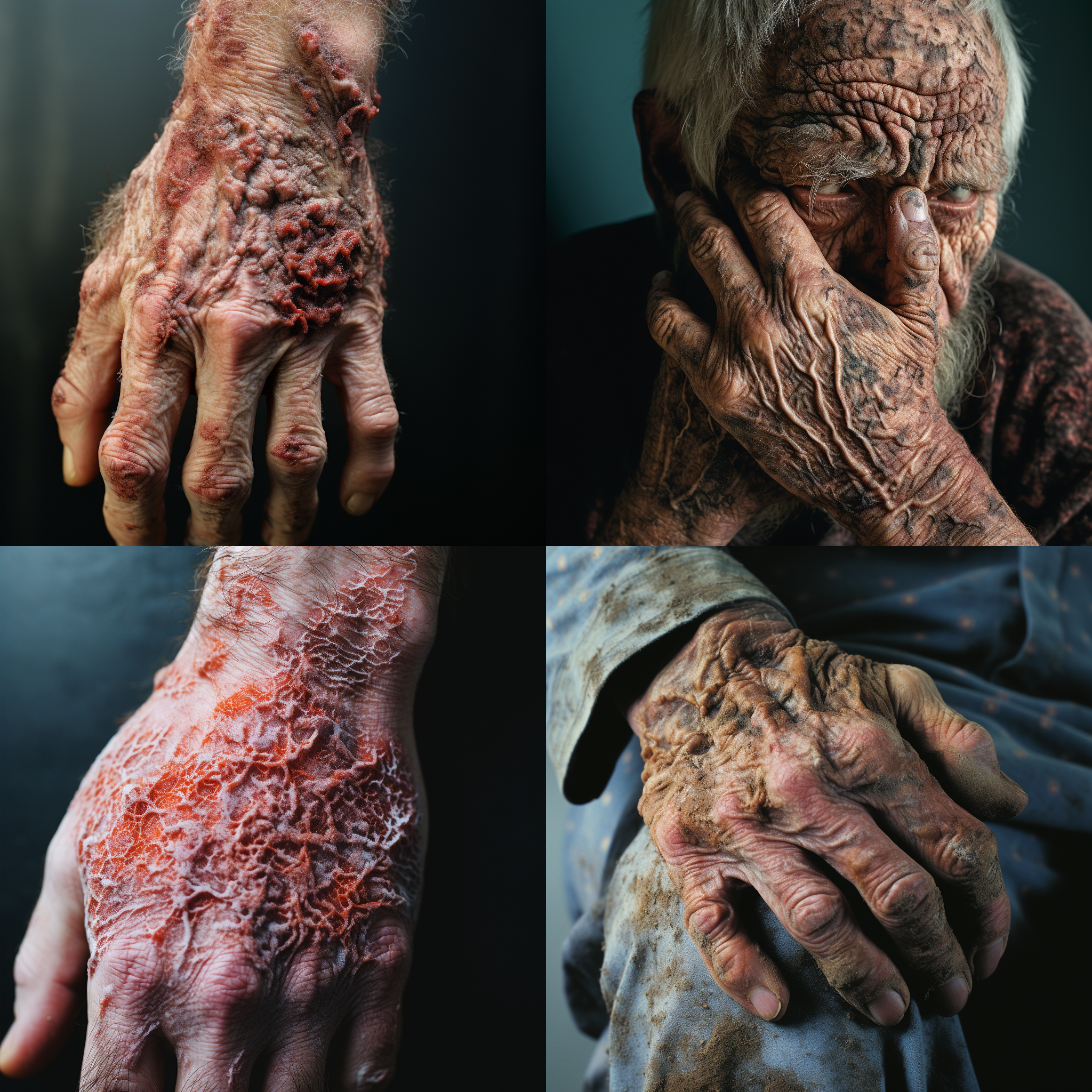

Supplement: Multimedia Appendix 2 [file ai_v3i1e58275_app2.zip › 11.andrewo999_a_photograph_showing_an_example_of_psoriasis_5c374a6f-f4ff-4bec-9af9-c0d467006bce.PNG]

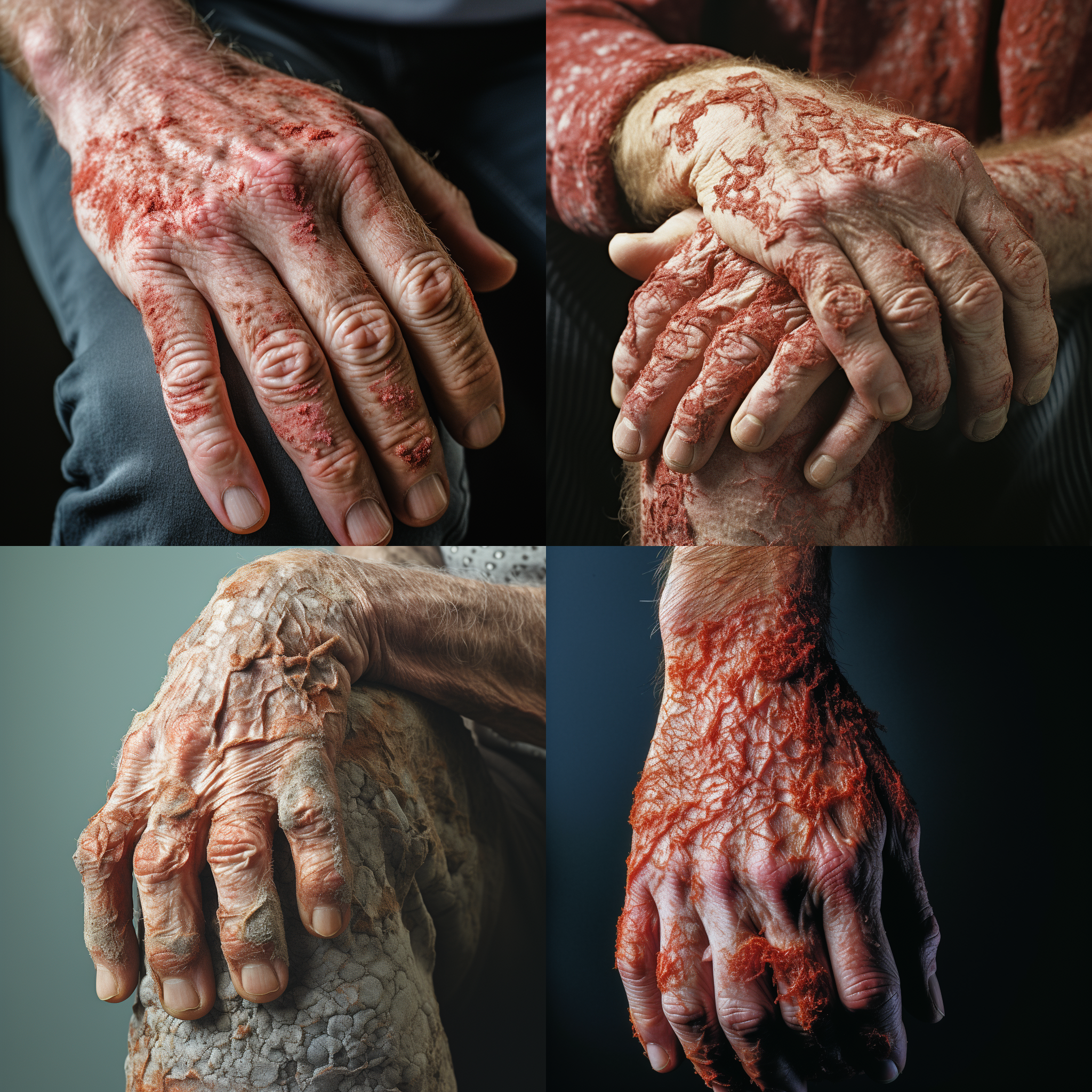

Supplement: Multimedia Appendix 2 [file ai_v3i1e58275_app2.zip › 16.andrewo999_a_photograph_showing_an_example_of_psoriasis_76b426a6-6b5b-461f-b61c-eb945b458837.PNG]

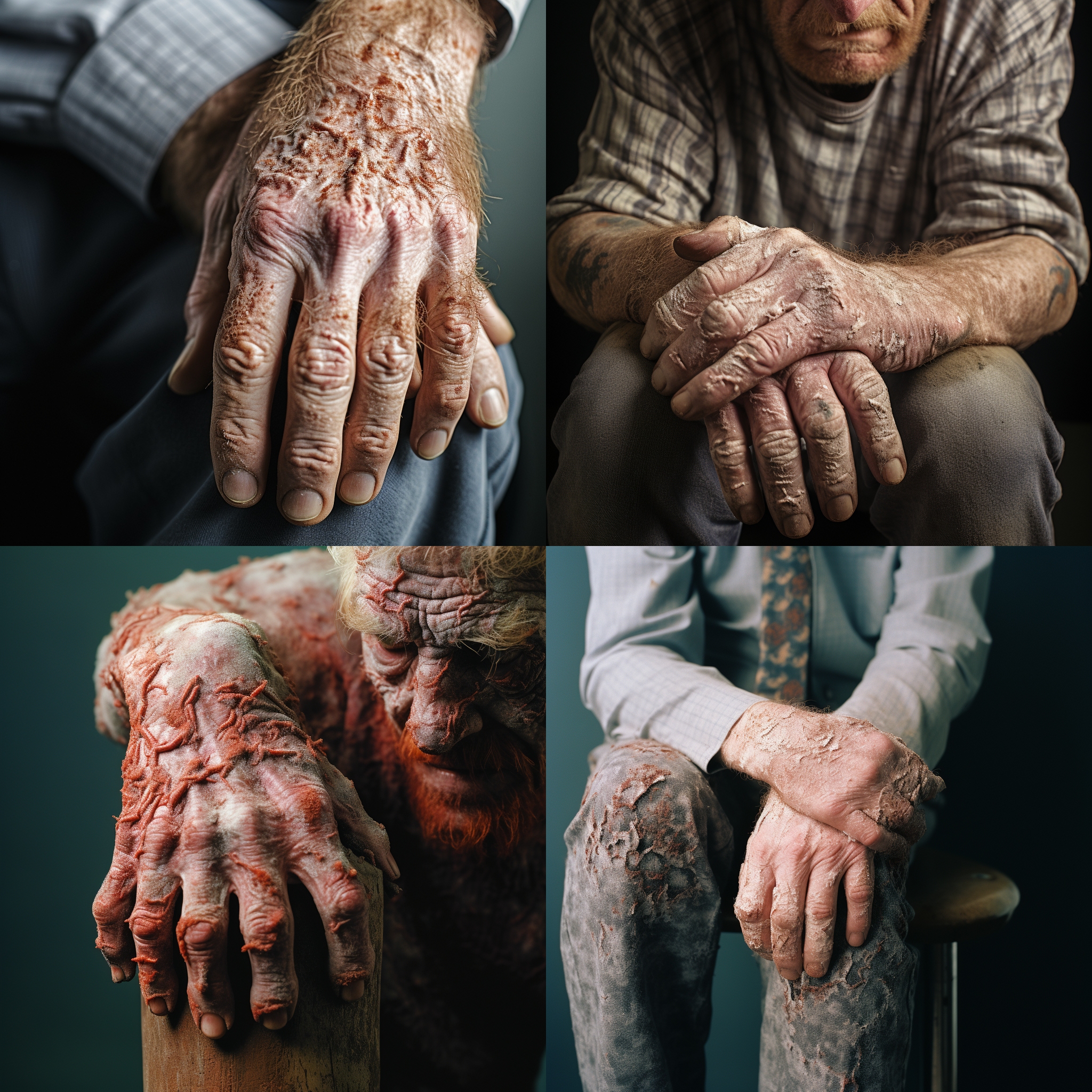

Supplement: Multimedia Appendix 2 [file ai_v3i1e58275_app2.zip › 09.andrewo999_a_photograph_showing_an_example_of_psoriasis_594073b5-0f0a-4065-9479-119d84b78453.PNG]

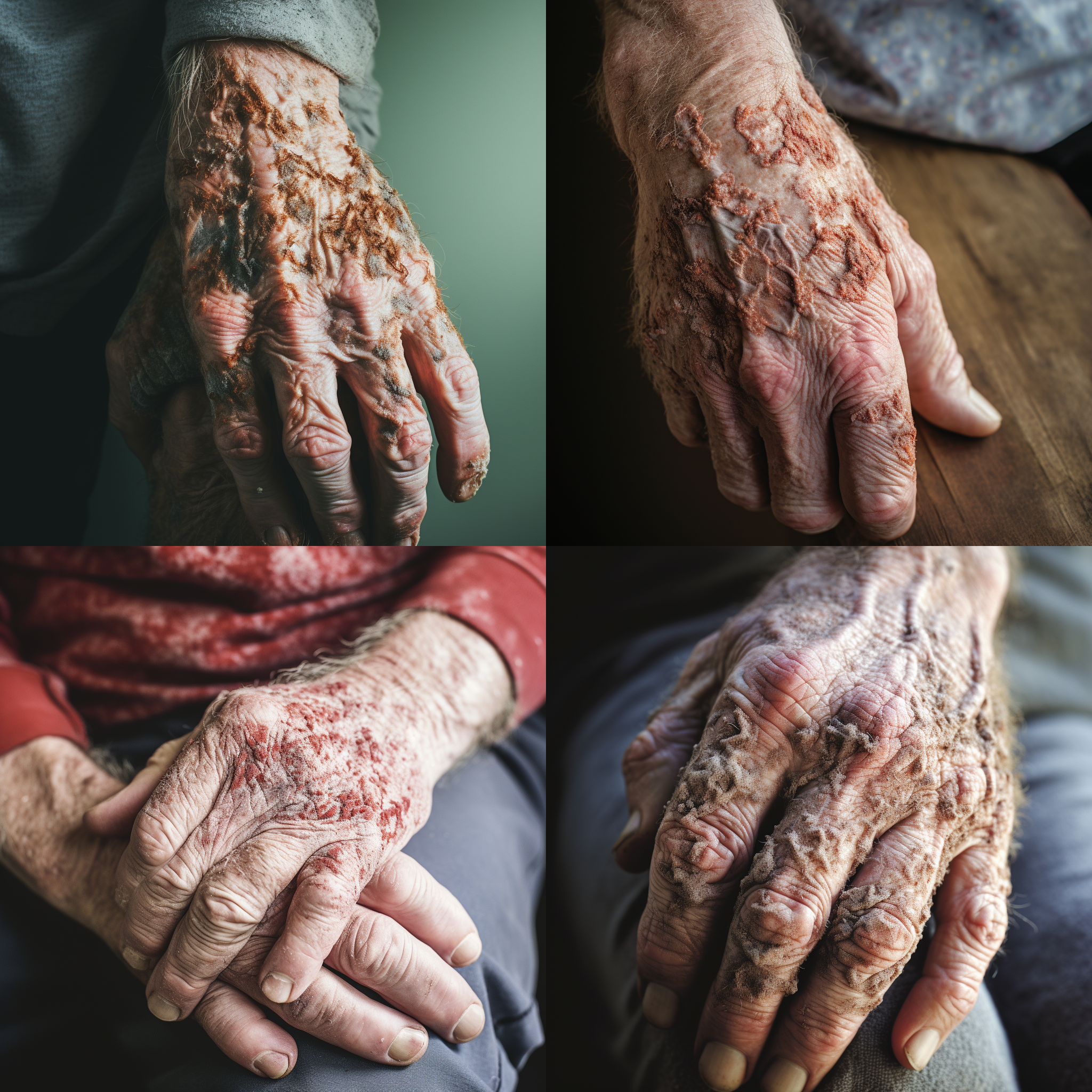

Supplement: Multimedia Appendix 2 [file ai_v3i1e58275_app2.zip › 20.andrewo999_a_photograph_showing_an_example_of_psoriasis_aaaad0b1-09c9-427d-829c-d737cf6f7f6d.PNG]

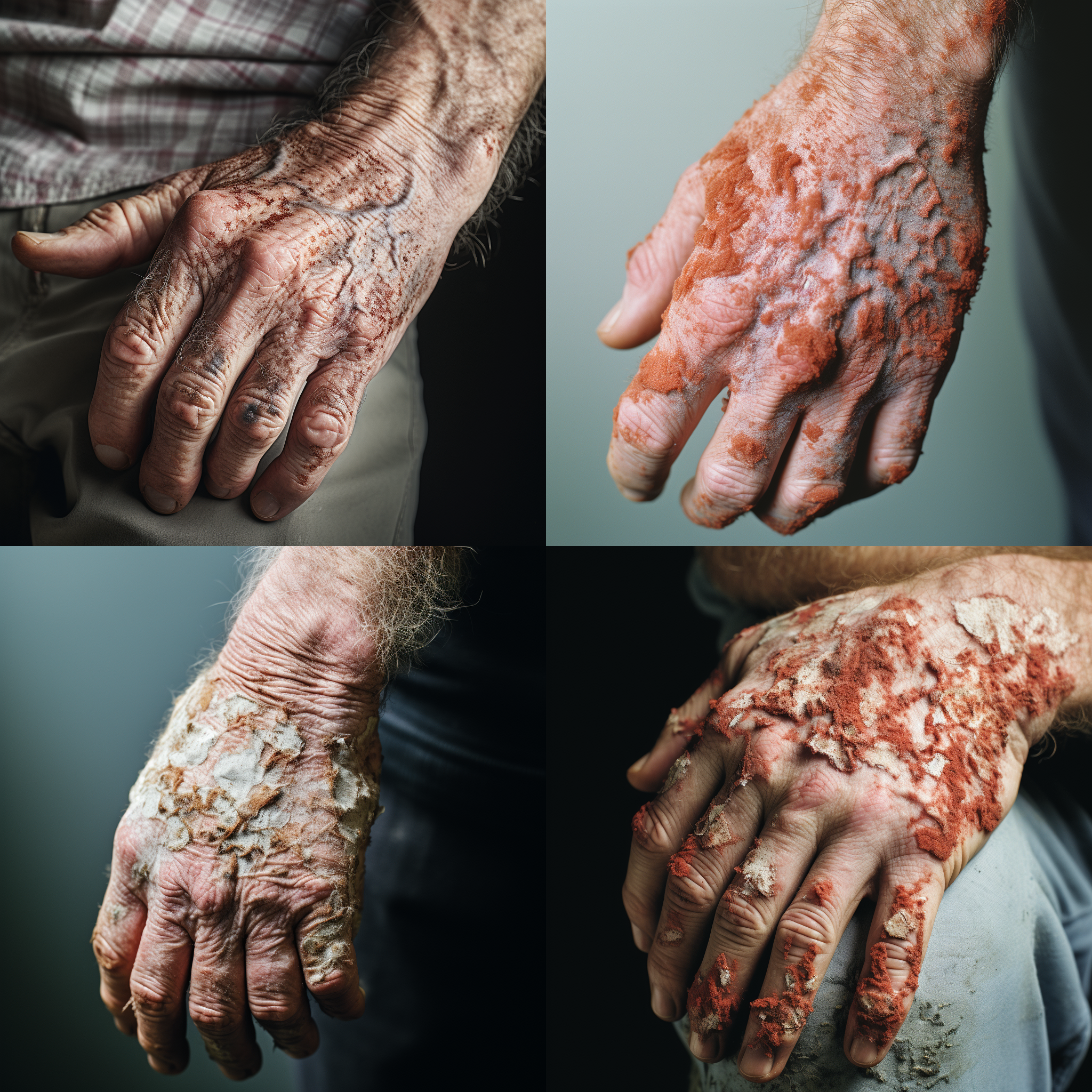

Supplement: Multimedia Appendix 2 [file ai_v3i1e58275_app2.zip › 03.andrewo999_a_photograph_showing_an_example_of_psoriasis_230c606d-e0f1-47ca-970d-fc8f317c4e2c.PNG]

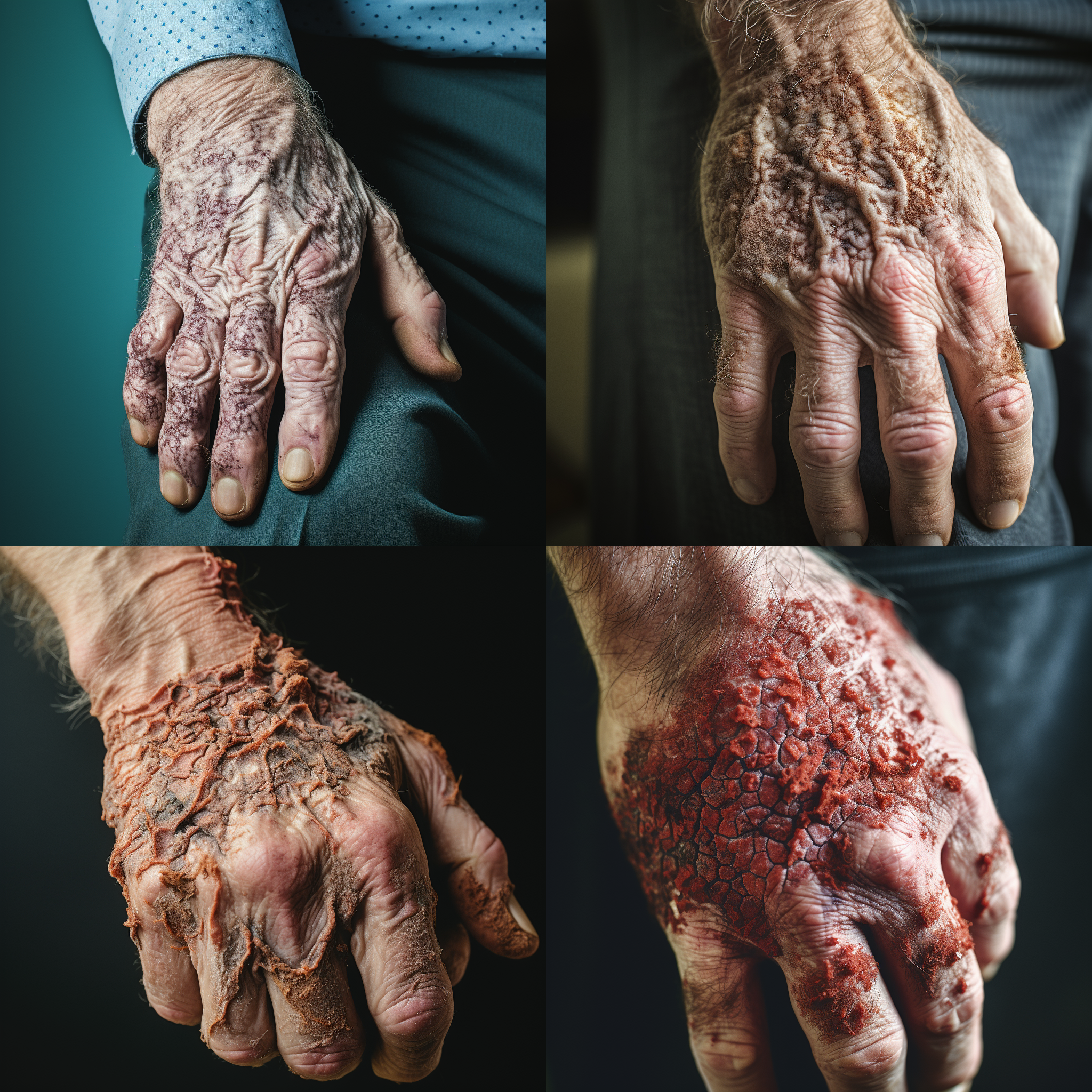

Supplement: Multimedia Appendix 2 [file ai_v3i1e58275_app2.zip › 15.andrewo999_a_photograph_showing_an_example_of_psoriasis_735dd934-0578-4dd9-9363-aecbb9b93009.PNG]

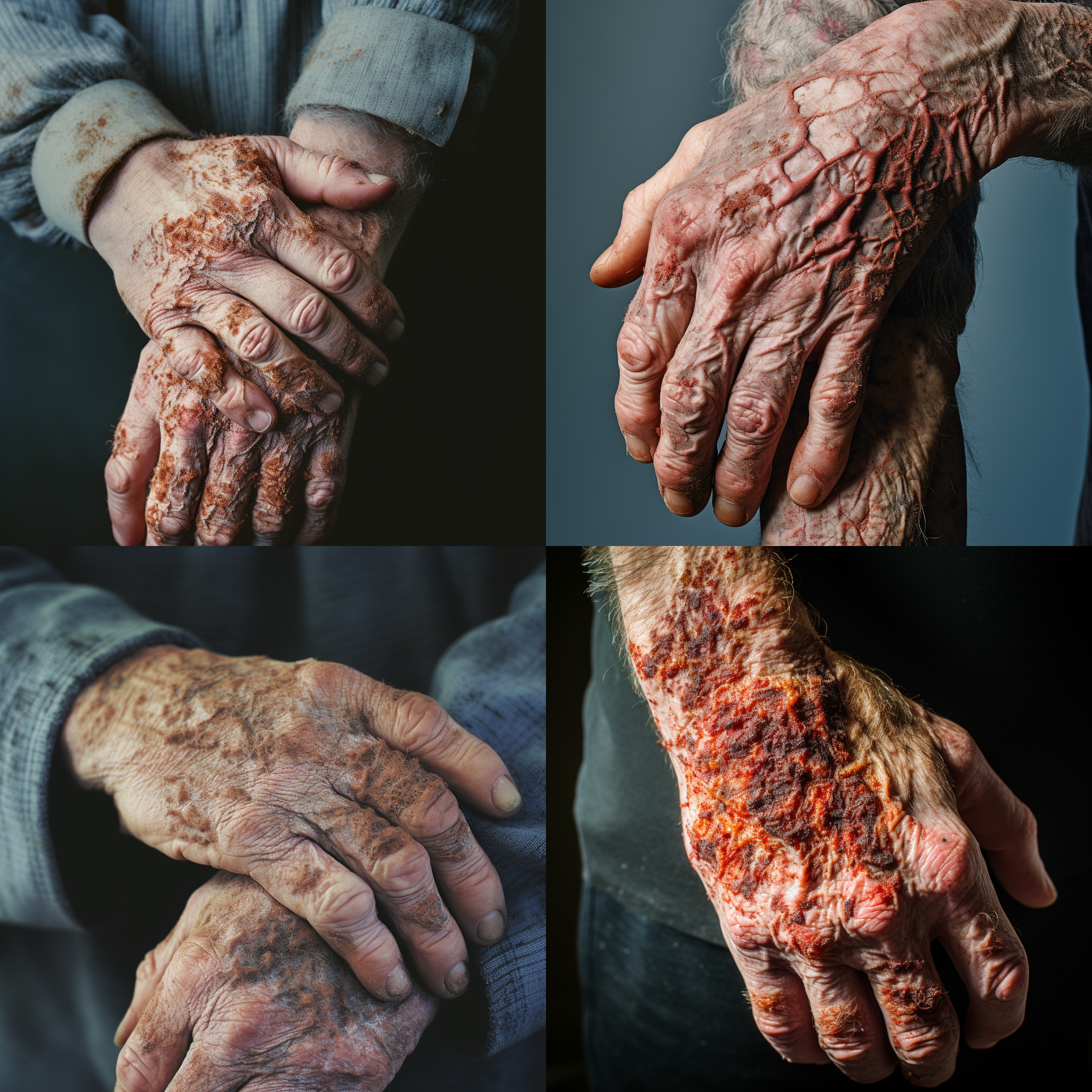

Supplement: Multimedia Appendix 2 [file ai_v3i1e58275_app2.zip › 06.andrewo999_a_photograph_showing_an_example_of_psoriasis_42de7ef9-d1d8-4e62-895f-20737923d228.PNG]

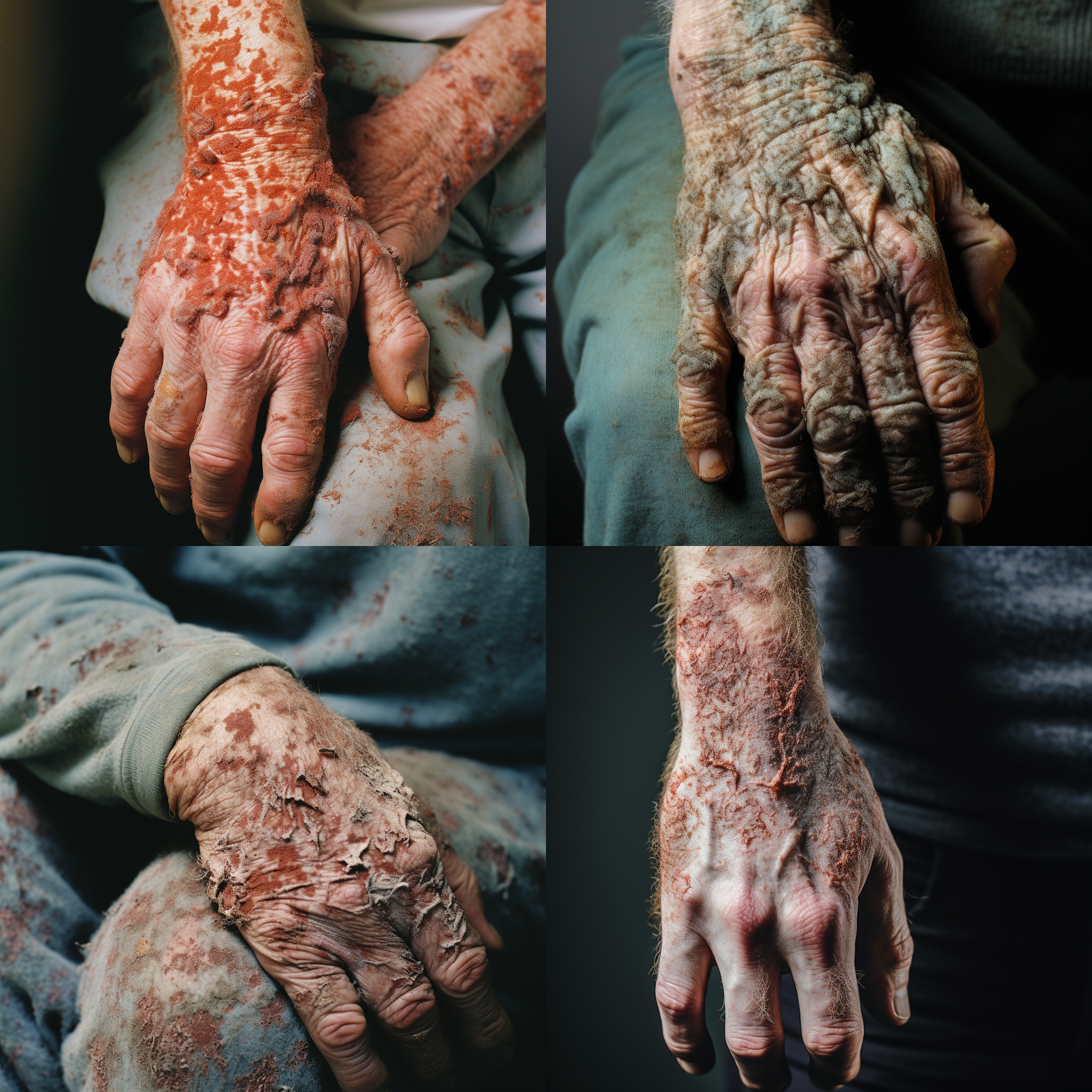

Supplement: Multimedia Appendix 2 [file ai_v3i1e58275_app2.zip › 08.andrewo999_a_photograph_showing_an_example_of_psoriasis_59262e03-744f-4153-ae4d-29ec70bfb57f.PNG]

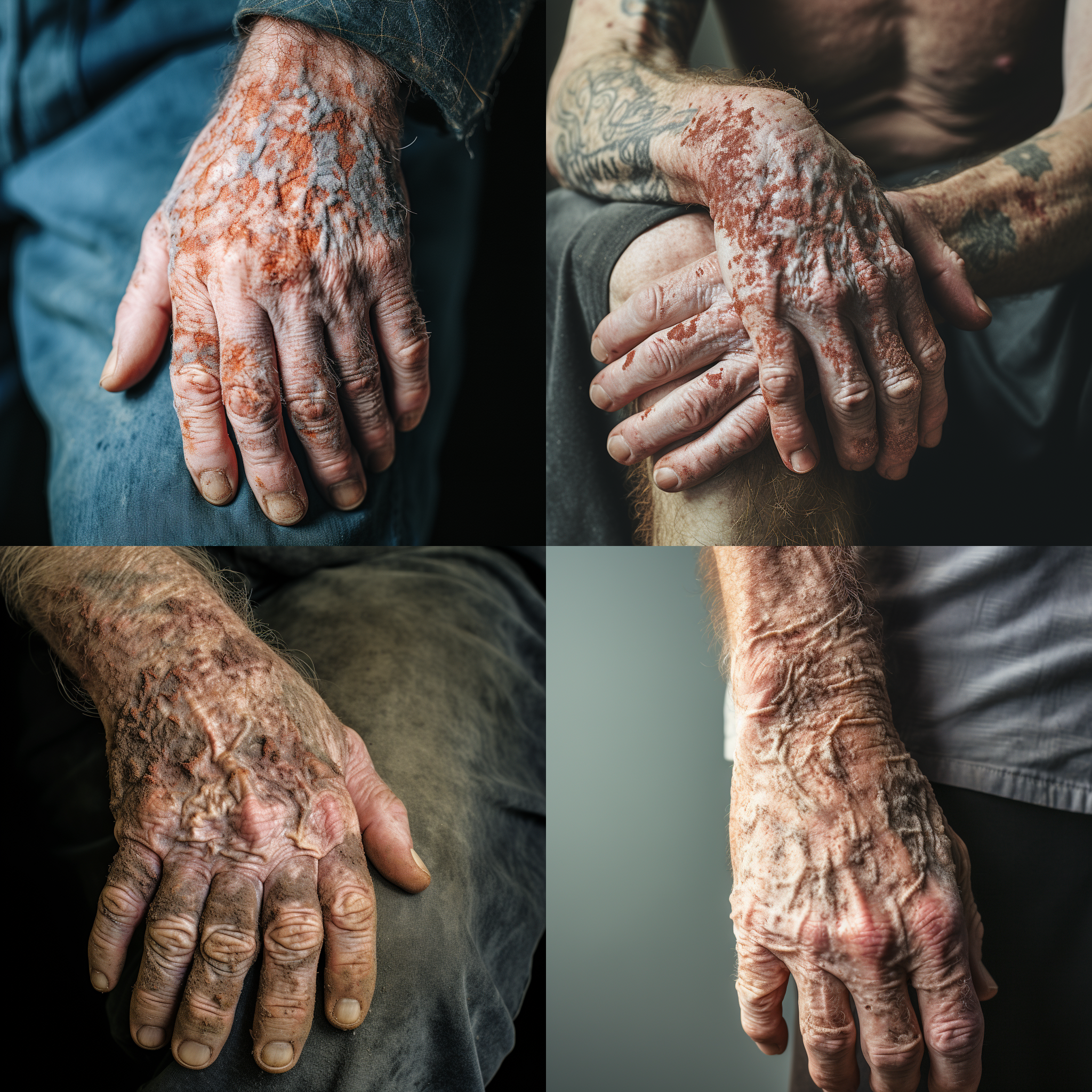

Supplement: Multimedia Appendix 2 [file ai_v3i1e58275_app2.zip › 14.andrewo999_a_photograph_showing_an_example_of_psoriasis_7185c6d2-58b0-4a4e-86cb-b3cfc40694a4.PNG]

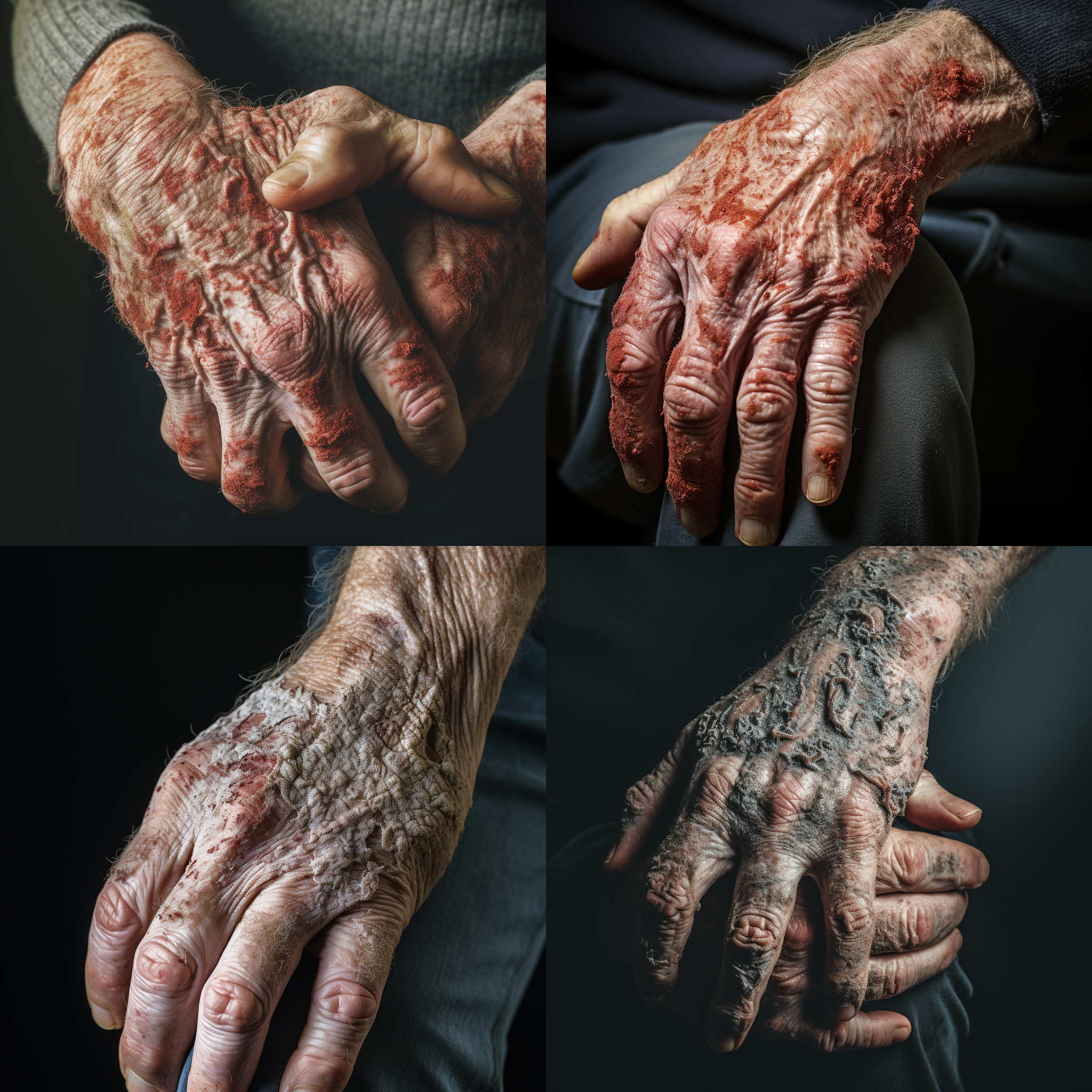

Supplement: Multimedia Appendix 2 [file ai_v3i1e58275_app2.zip › 12.andrewo999_a_photograph_showing_an_example_of_psoriasis_613592eb-6e7f-4144-98bd-f182ee5622d8.PNG]

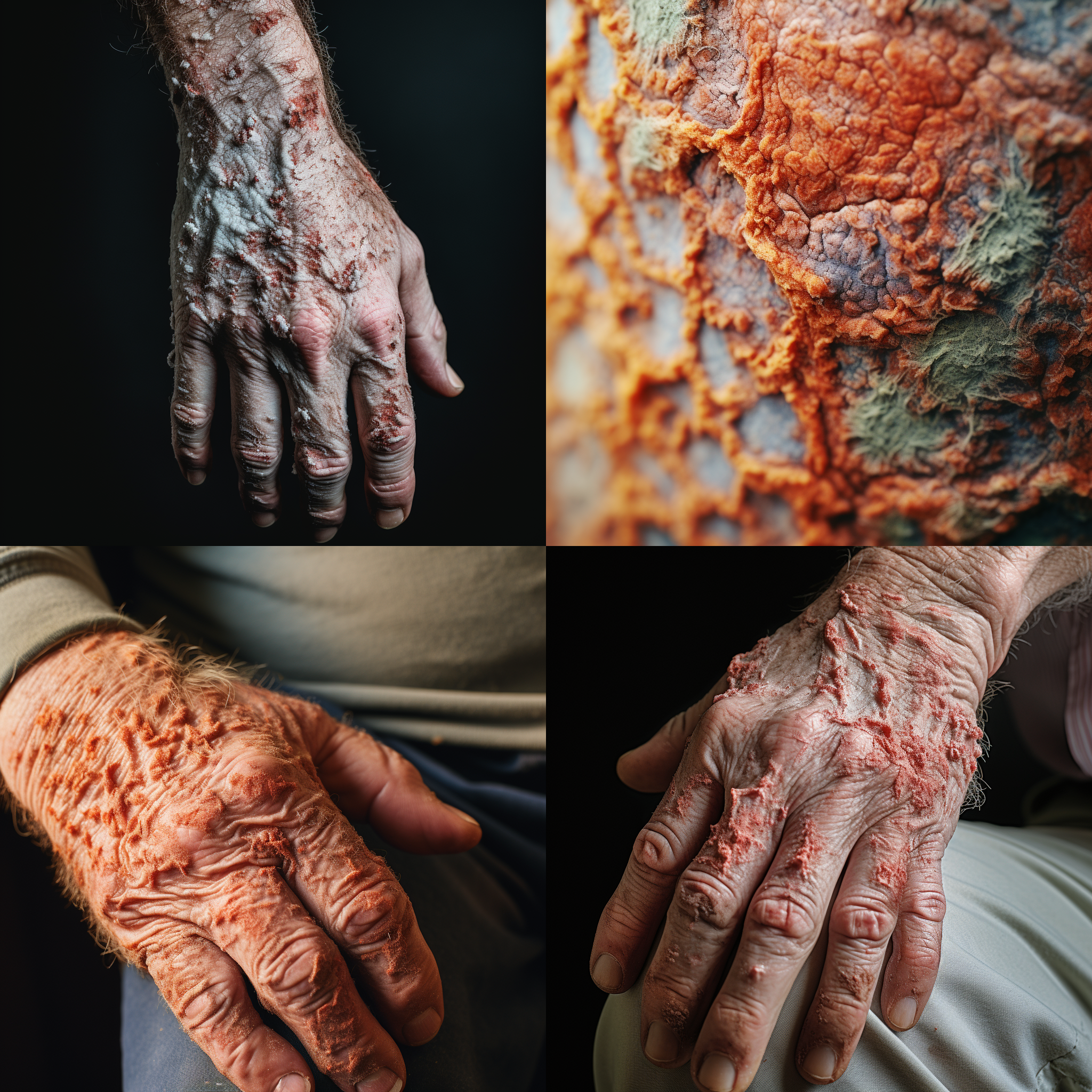

Supplement: Multimedia Appendix 2 [file ai_v3i1e58275_app2.zip › 07.andrewo999_a_photograph_showing_an_example_of_psoriasis_4ccfbc4f-1cb4-410c-a636-48632260222b.PNG]

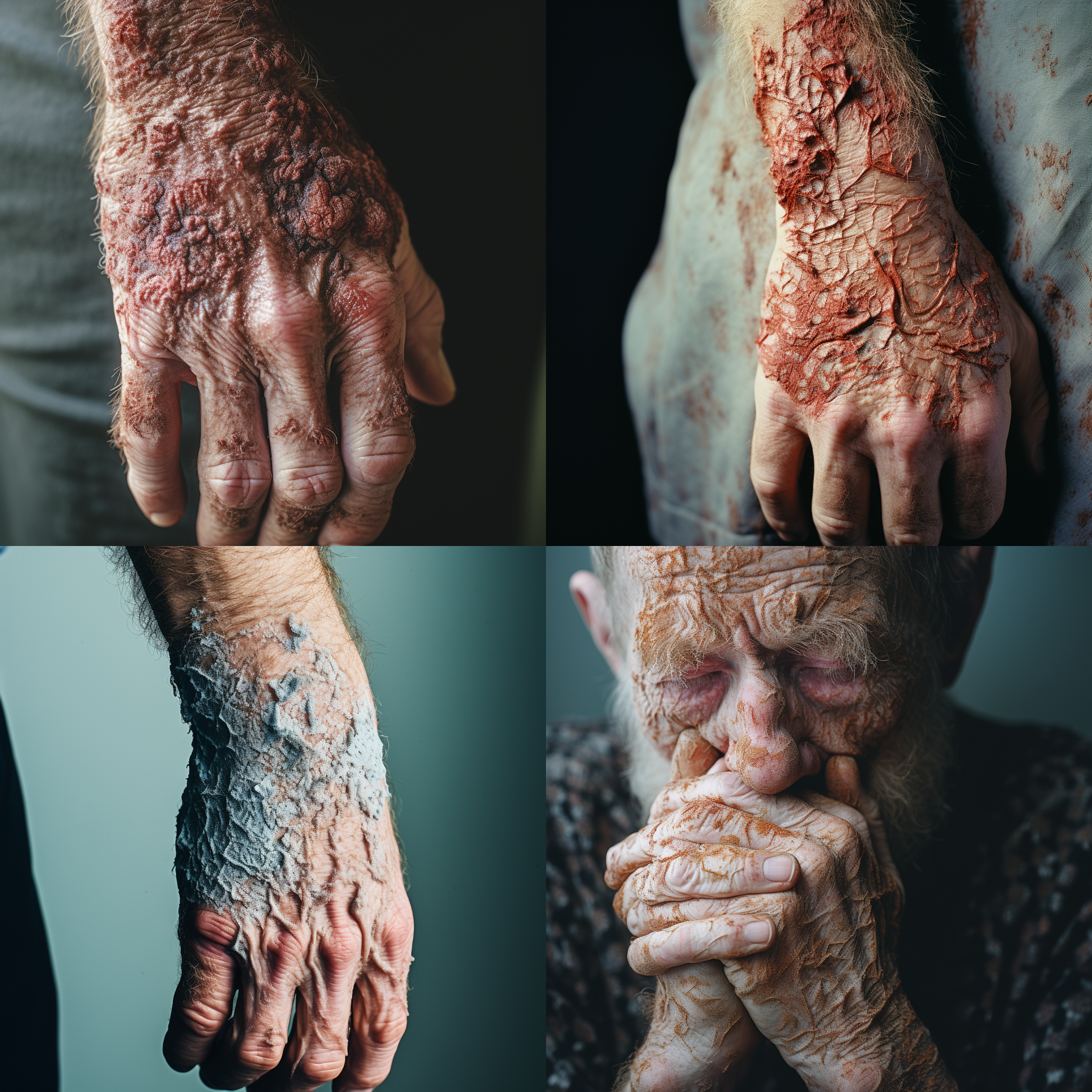

Supplement: Multimedia Appendix 2 [file ai_v3i1e58275_app2.zip › 19.andrewo999_a_photograph_showing_an_example_of_psoriasis_98f05d76-8a9f-474e-ab2b-6b65e040e0d4.PNG]

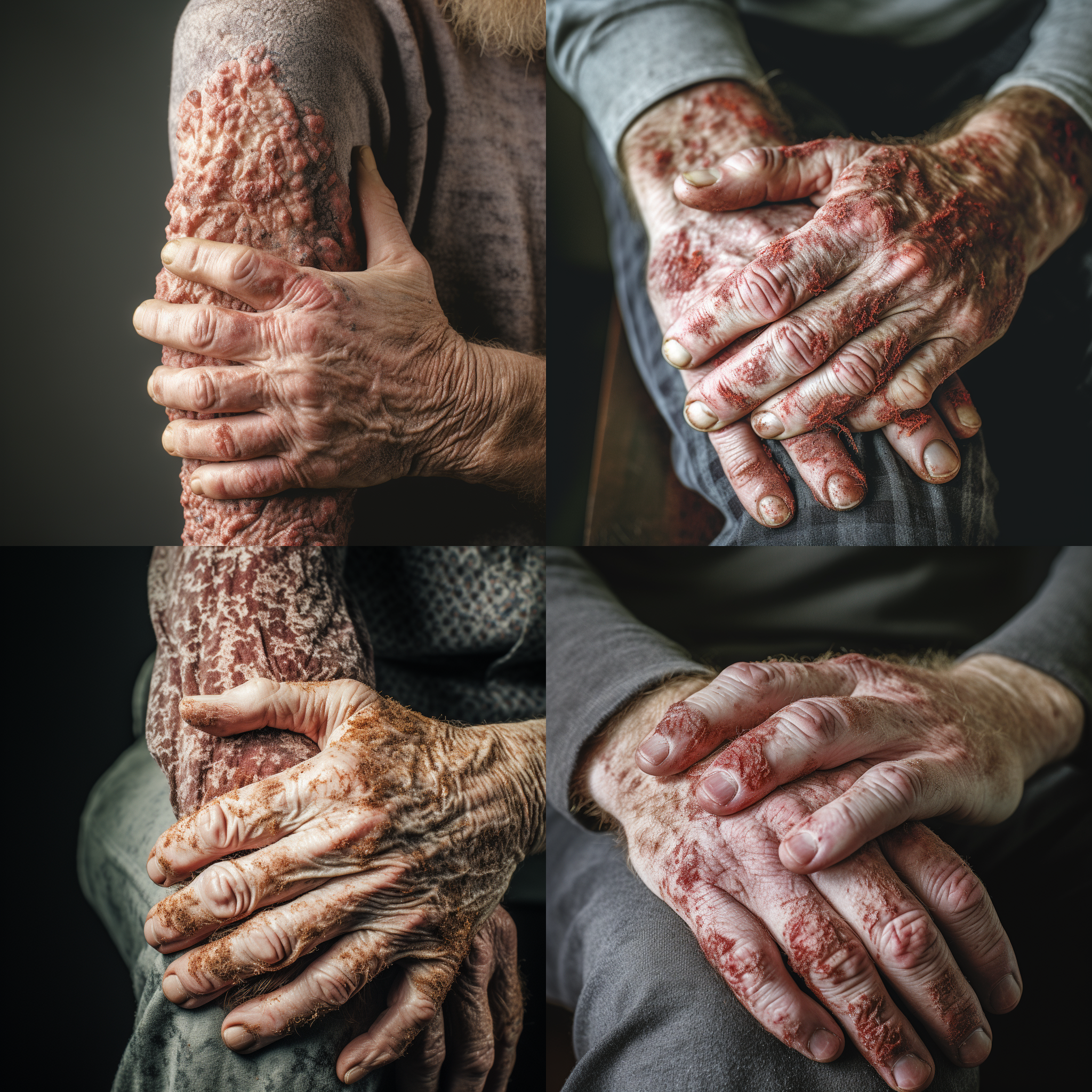

Supplement: Multimedia Appendix 2 [file ai_v3i1e58275_app2.zip › 23.andrewo999_a_photograph_showing_an_example_of_psoriasis_c5ac60d9-4084-4467-bf1f-d8b9cbfb6098.PNG]

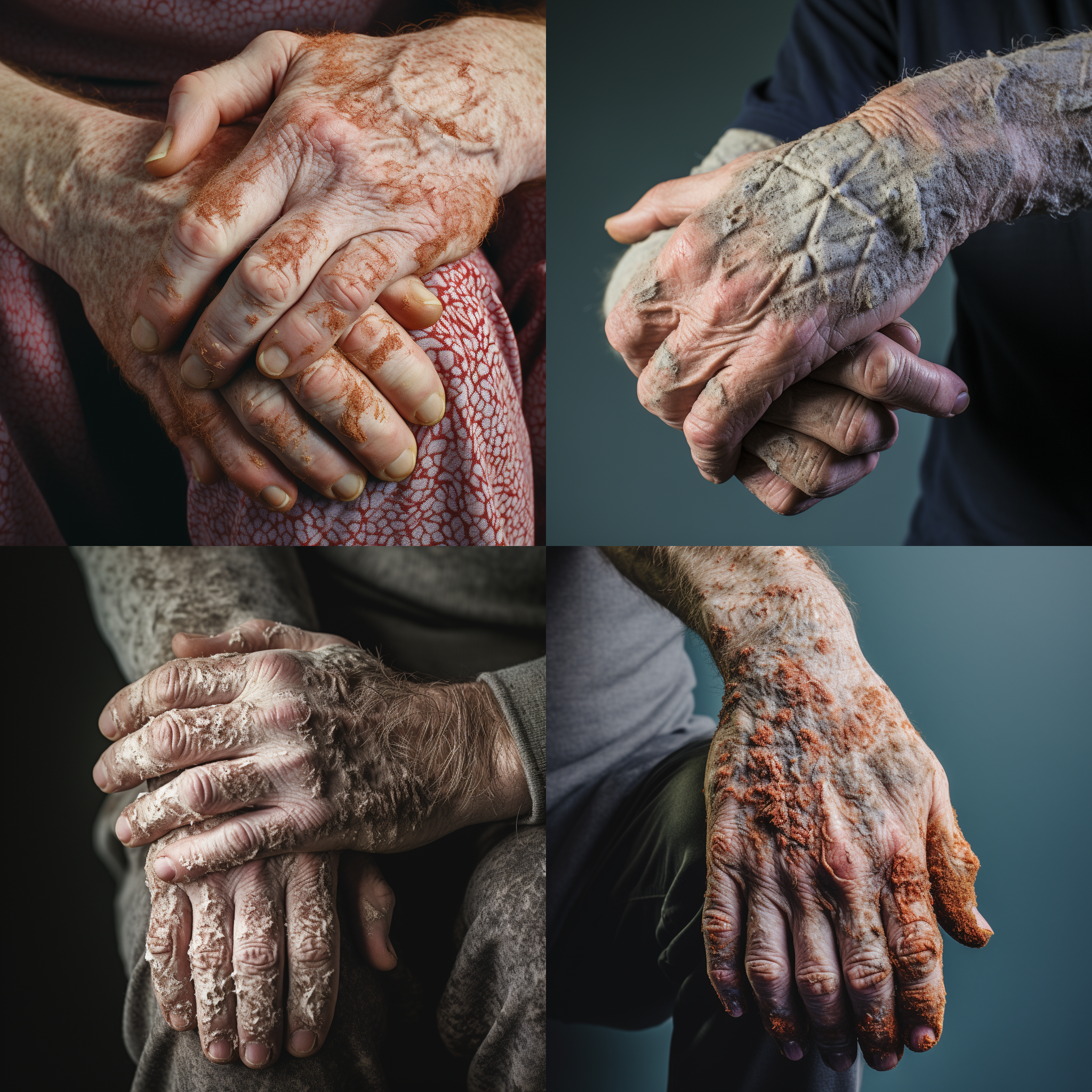

Supplement: Multimedia Appendix 2 [file ai_v3i1e58275_app2.zip › 17.andrewo999_a_photograph_showing_an_example_of_psoriasis_8dc5ce74-95ec-448d-9cb0-2beeb780943a.PNG]

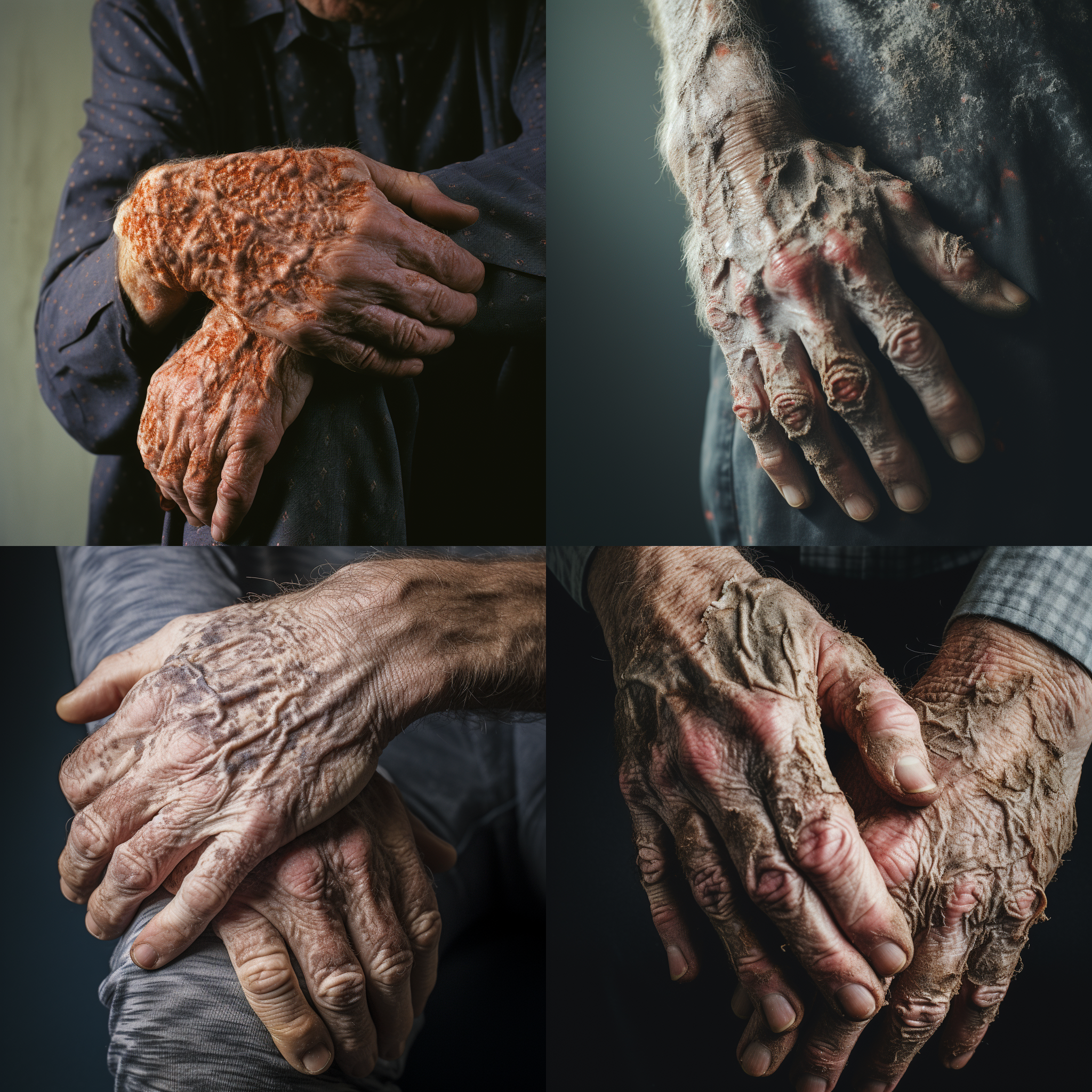

Supplement: Multimedia Appendix 2 [file ai_v3i1e58275_app2.zip › 02.andrewo999_a_photograph_showing_an_example_of_psoriasis_0d1963fc-5ce7-4200-a06d-53a0b0e5ec5d.PNG]

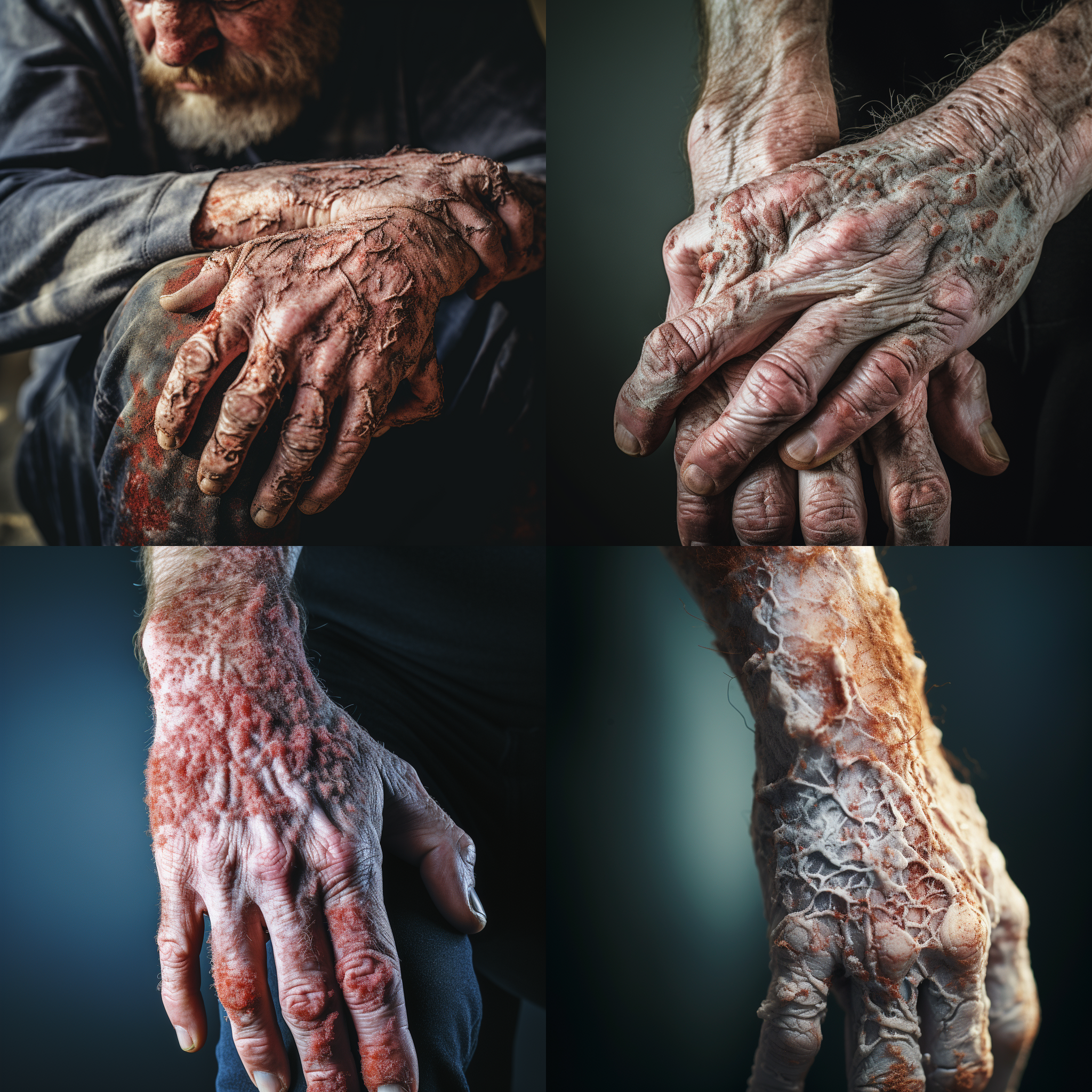

Supplement: Multimedia Appendix 2 [file ai_v3i1e58275_app2.zip › 18.andrewo999_a_photograph_showing_an_example_of_psoriasis_8fbff582-38ab-4fa8-b06d-0ecf9a800843.PNG]

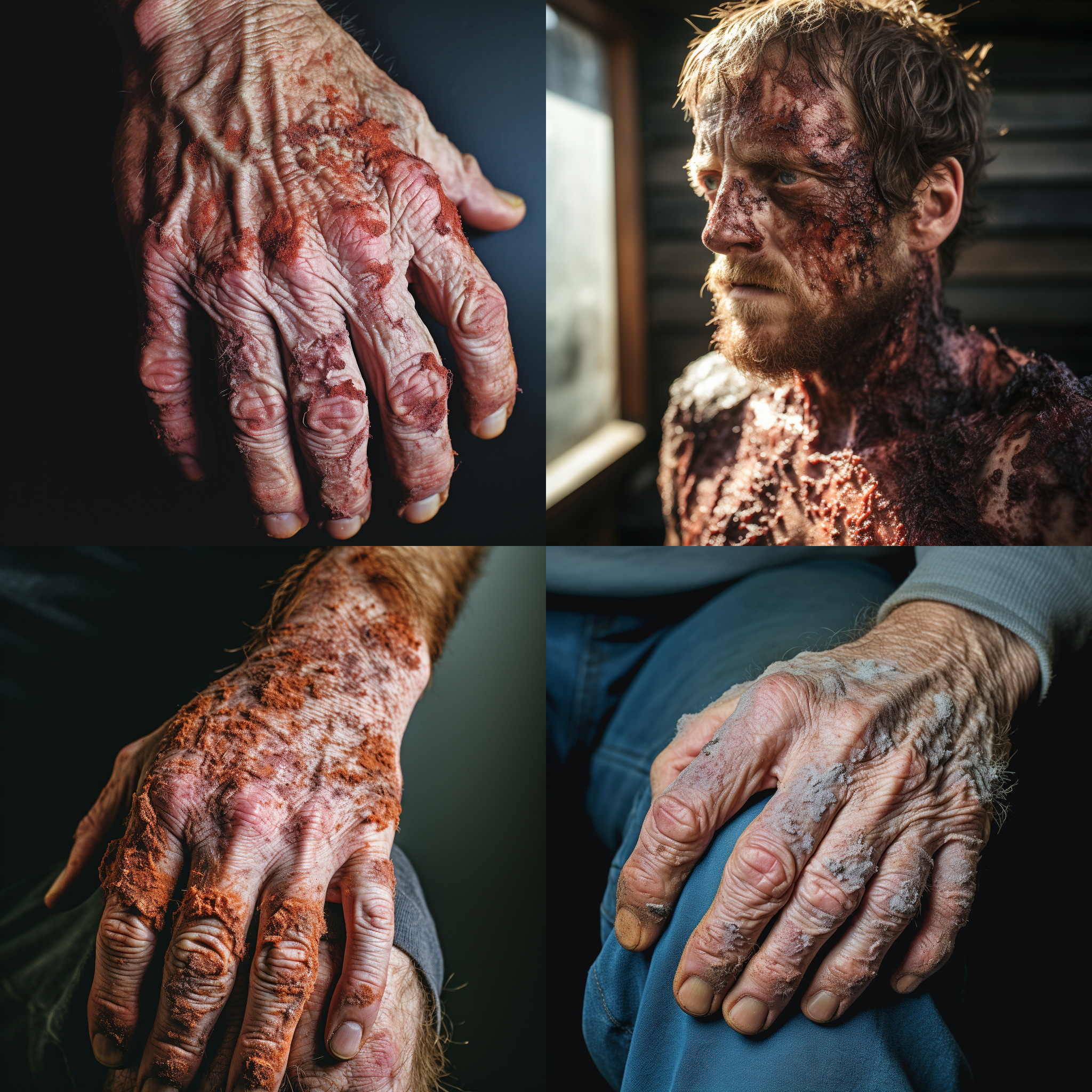

Supplement: Multimedia Appendix 2 [file ai_v3i1e58275_app2.zip › 04.andrewo999_a_photograph_showing_an_example_of_psoriasis_34a02b12-0333-43d0-b53b-3cf6597deaf9.PNG]

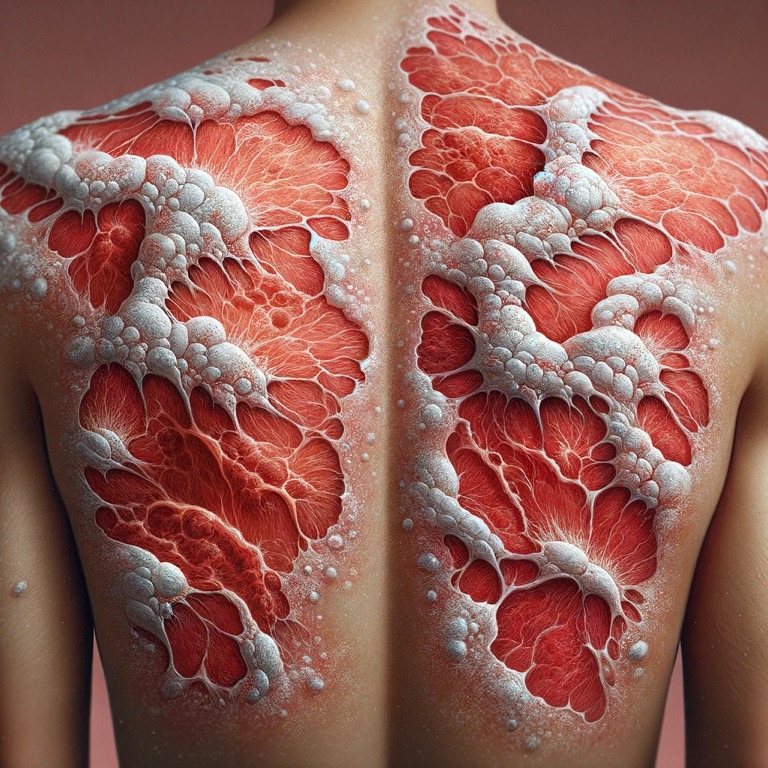

Supplement: Multimedia Appendix 3 [file ai_v3i1e58275_app3.zip › 50.jpeg]

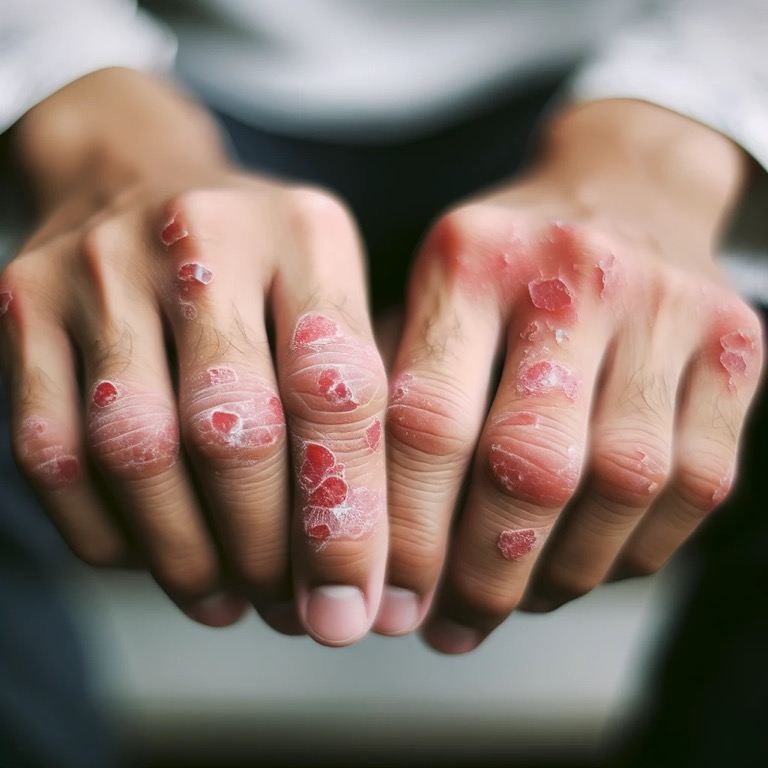

Supplement: Multimedia Appendix 3 [file ai_v3i1e58275_app3.zip › 27.JPG]

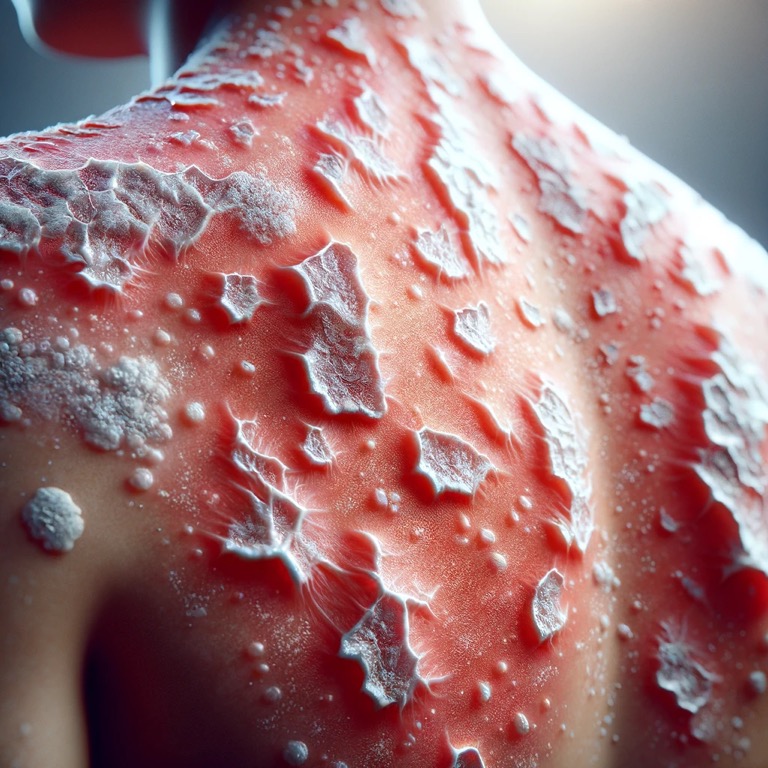

Supplement: Multimedia Appendix 3 [file ai_v3i1e58275_app3.zip › 53.jpeg]

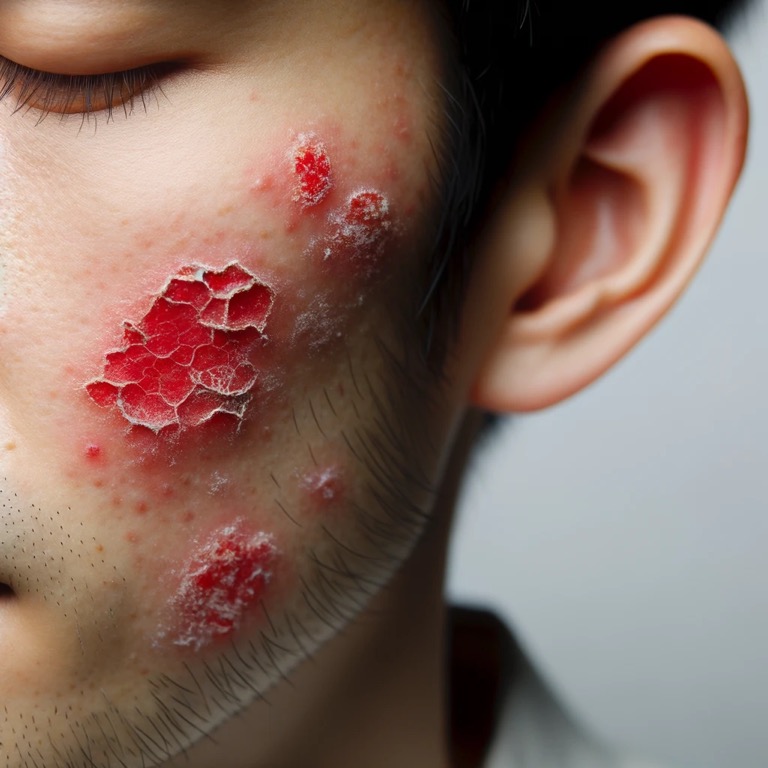

Supplement: Multimedia Appendix 3 [file ai_v3i1e58275_app3.zip › 25.JPG]

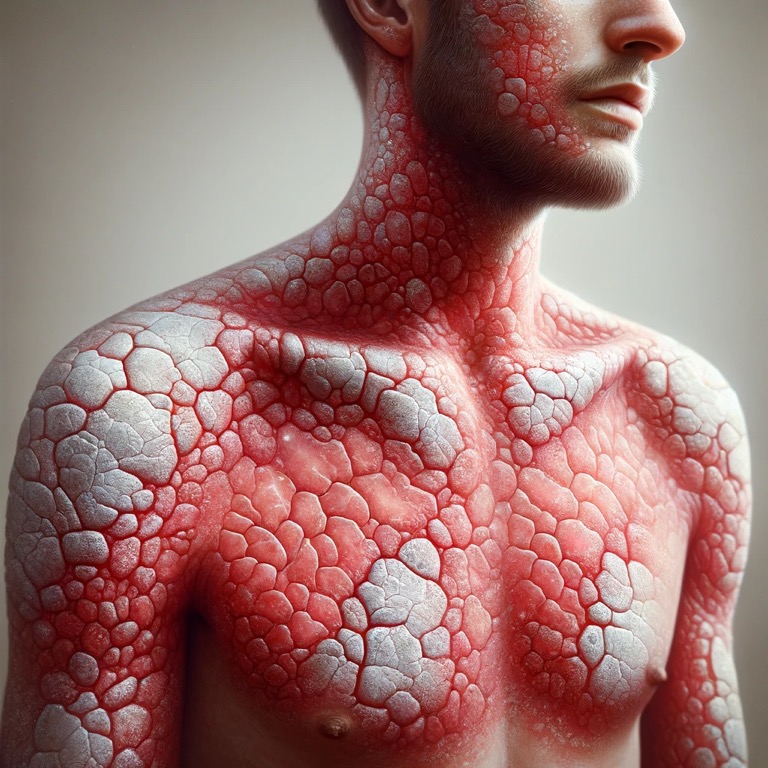

Supplement: Multimedia Appendix 3 [file ai_v3i1e58275_app3.zip › 55.jpeg]

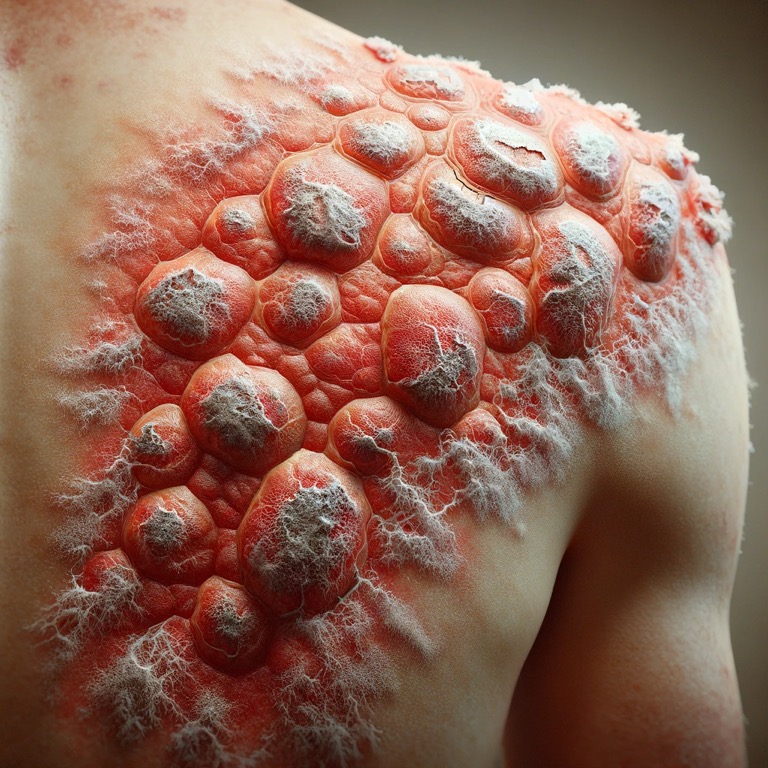

Supplement: Multimedia Appendix 3 [file ai_v3i1e58275_app3.zip › 34.jpeg]

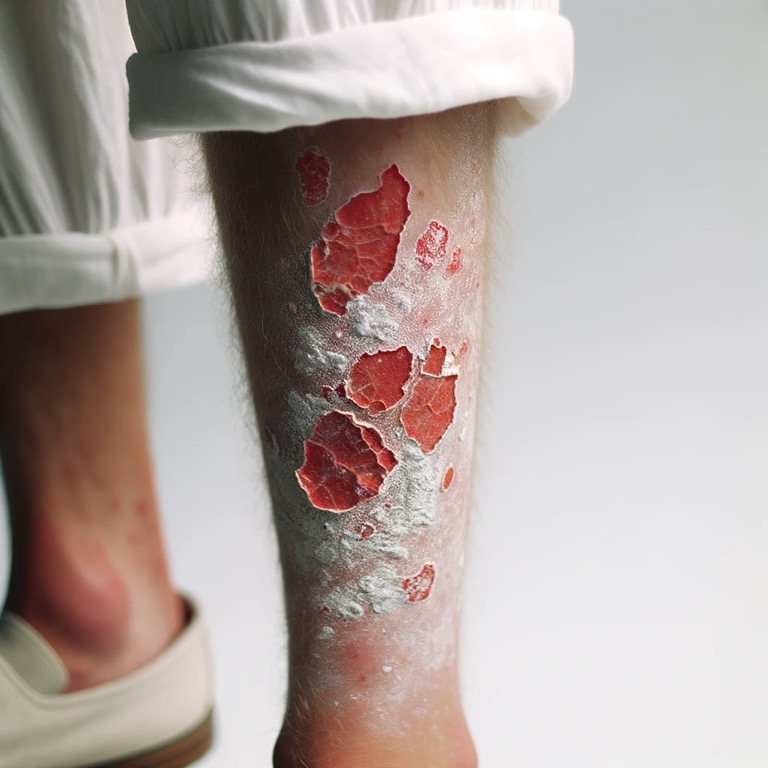

Supplement: Multimedia Appendix 3 [file ai_v3i1e58275_app3.zip › 28.JPG]

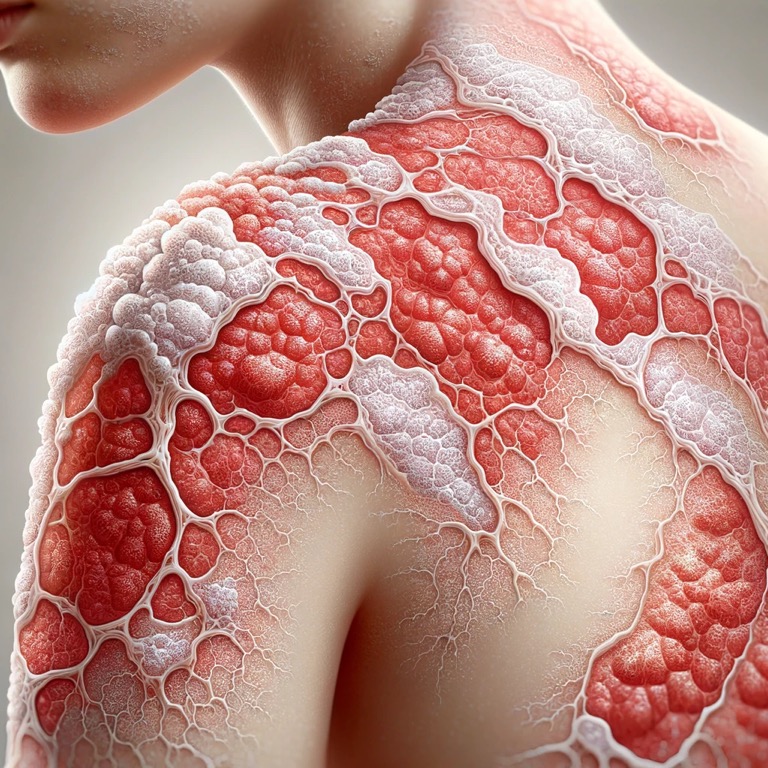

Supplement: Multimedia Appendix 3 [file ai_v3i1e58275_app3.zip › 49.jpeg]

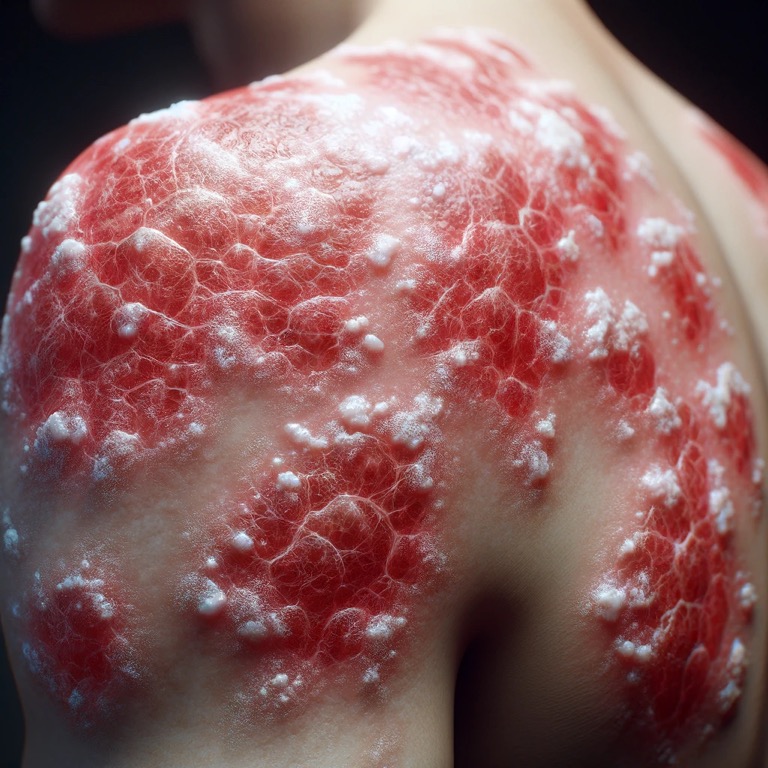

Supplement: Multimedia Appendix 3 [file ai_v3i1e58275_app3.zip › 45.jpeg]

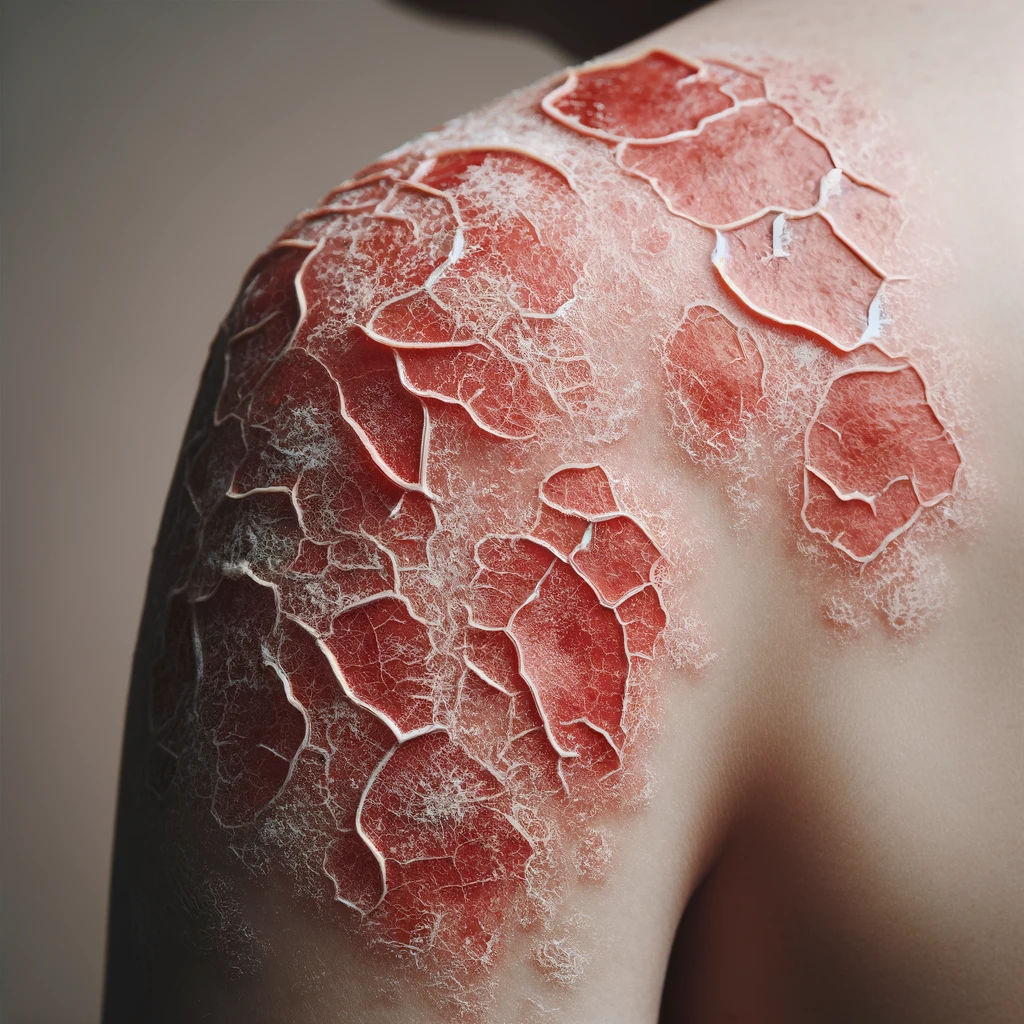

Supplement: Multimedia Appendix 3 [file ai_v3i1e58275_app3.zip › 57.WEBP]

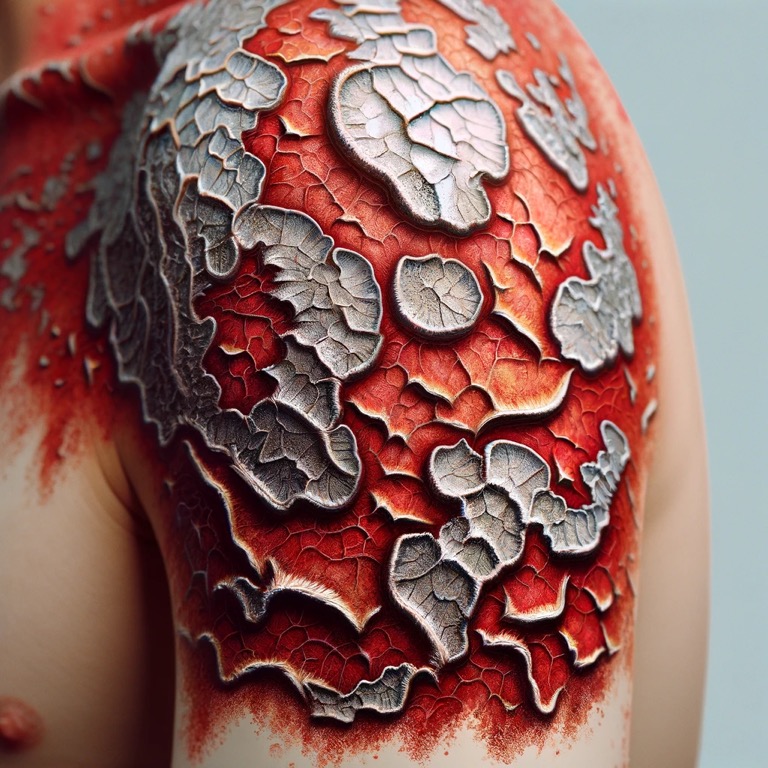

Supplement: Multimedia Appendix 3 [file ai_v3i1e58275_app3.zip › 54.jpeg]

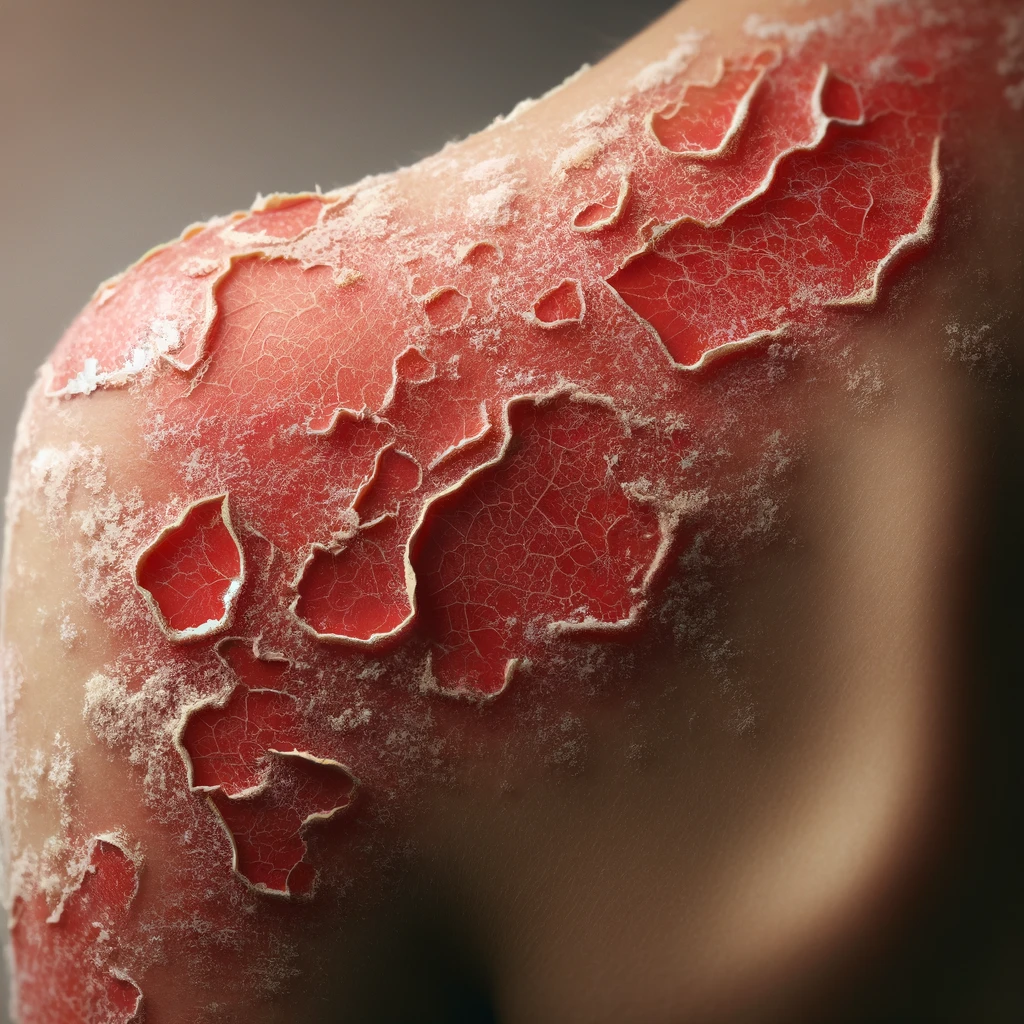

Supplement: Multimedia Appendix 3 [file ai_v3i1e58275_app3.zip › 77.WEBP]

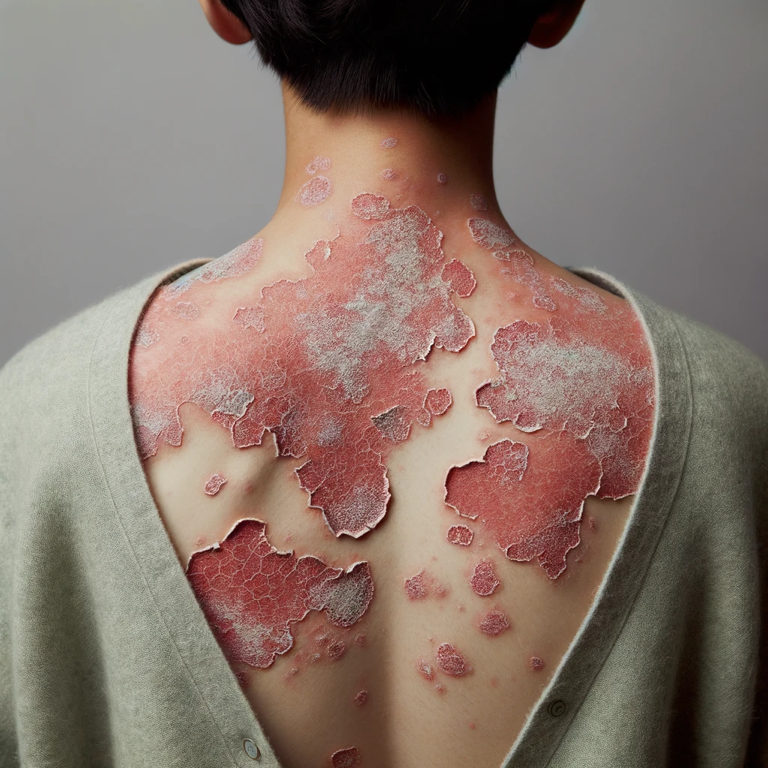

Supplement: Multimedia Appendix 3 [file ai_v3i1e58275_app3.zip › 09.PNG]

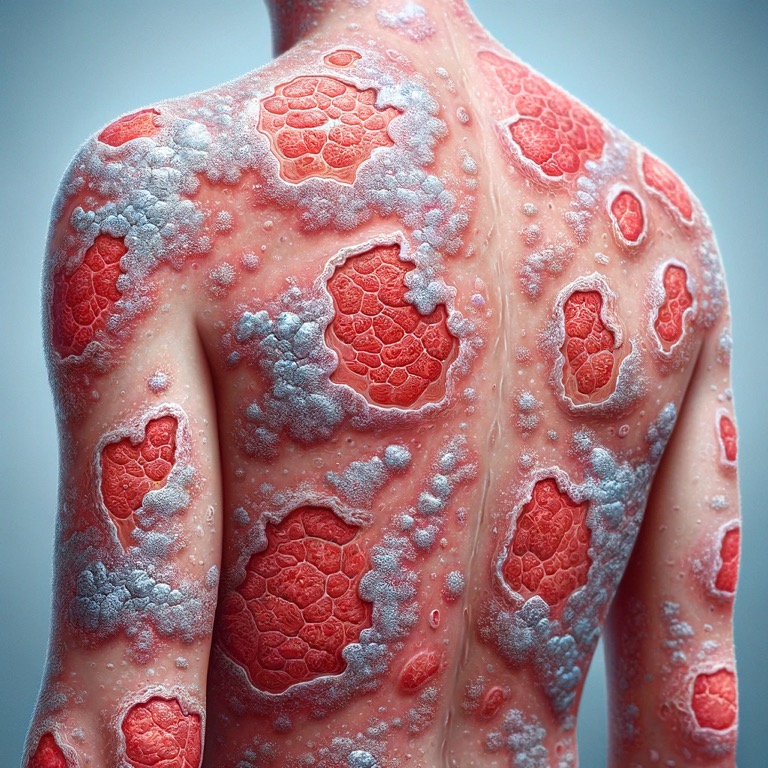

Supplement: Multimedia Appendix 3 [file ai_v3i1e58275_app3.zip › 47.jpeg]

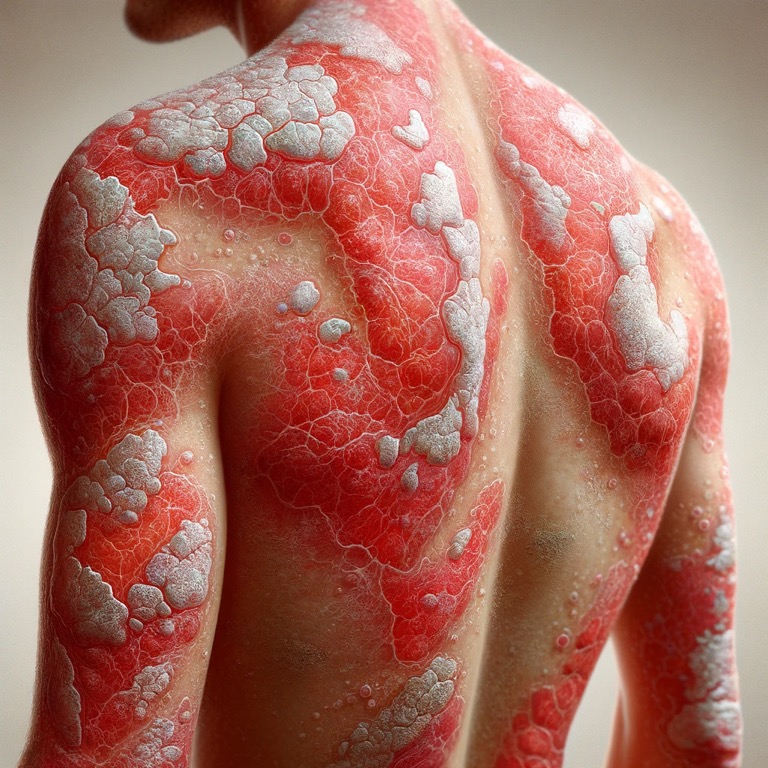

Supplement: Multimedia Appendix 3 [file ai_v3i1e58275_app3.zip › 62.jpeg]

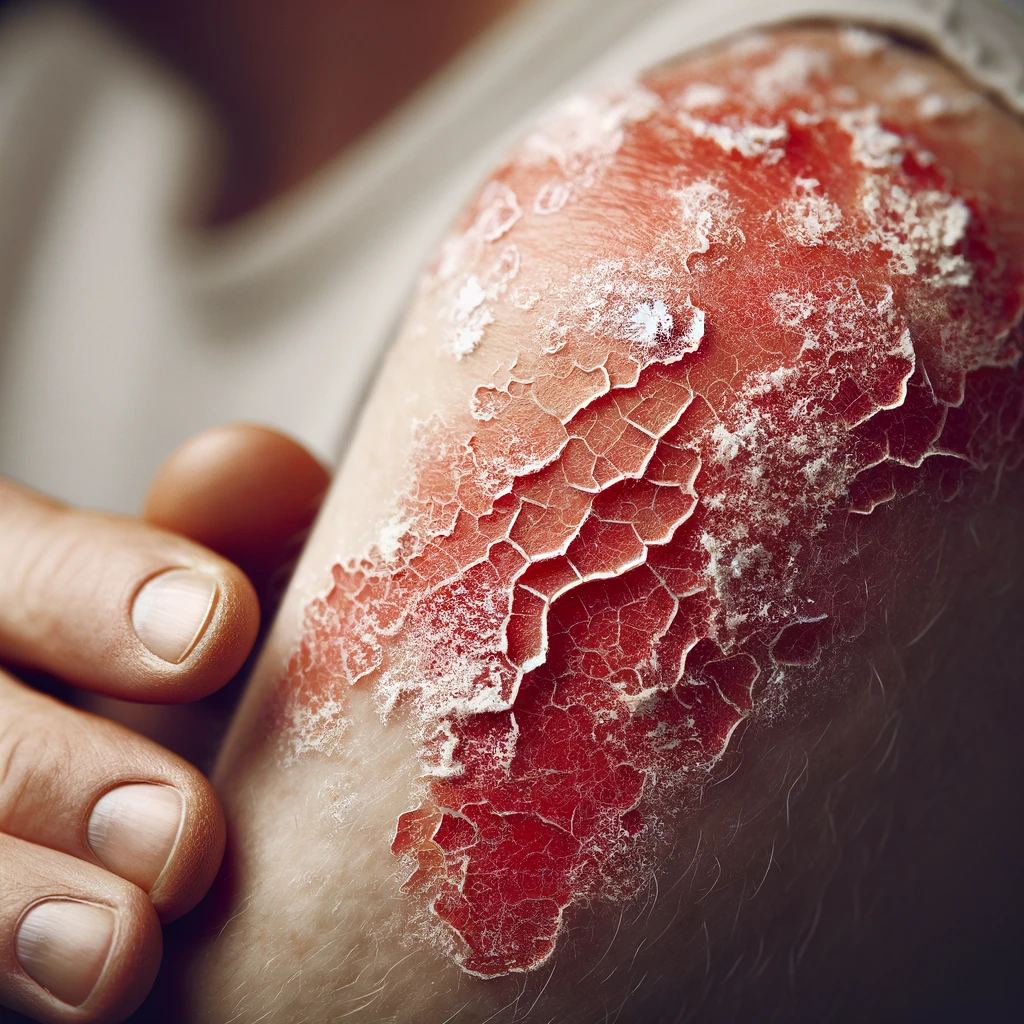

Supplement: Multimedia Appendix 3 [file ai_v3i1e58275_app3.zip › 83.WEBP]

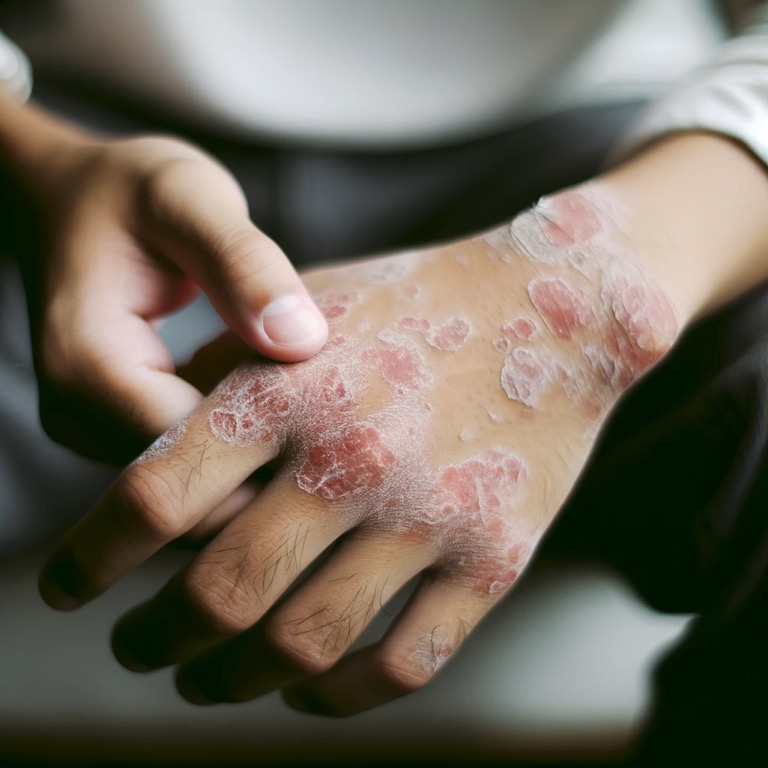

Supplement: Multimedia Appendix 3 [file ai_v3i1e58275_app3.zip › 26.JPG]

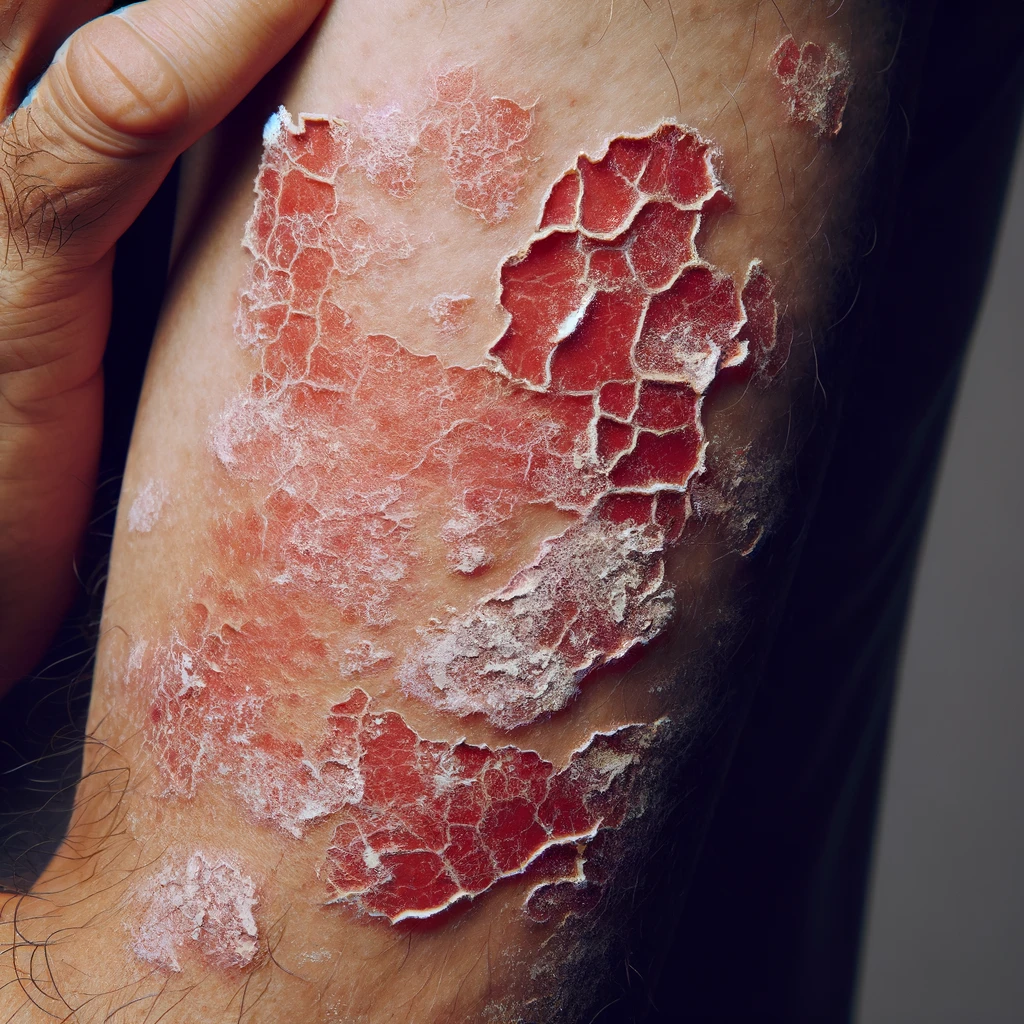

Supplement: Multimedia Appendix 3 [file ai_v3i1e58275_app3.zip › 84.WEBP]

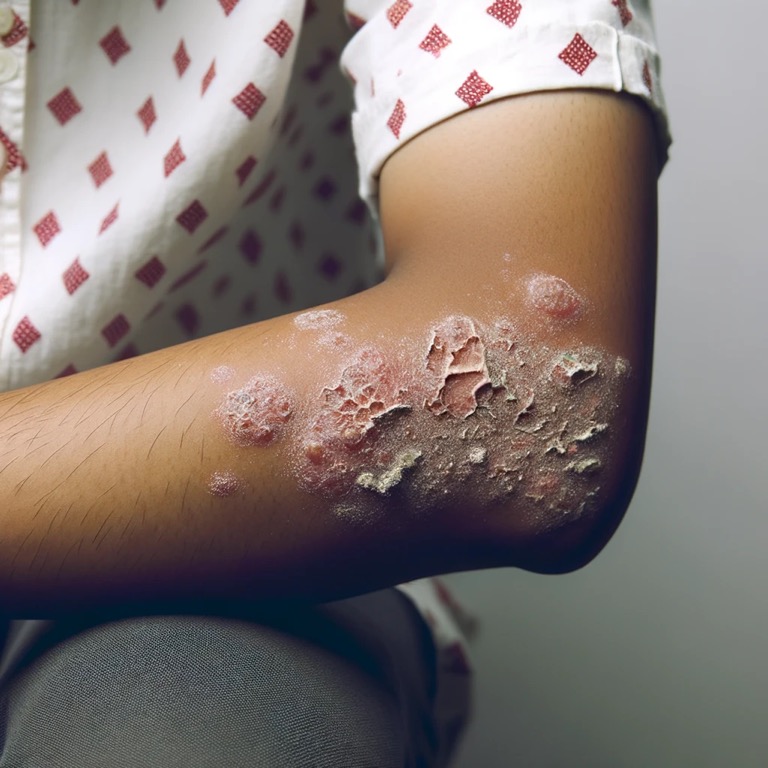

Supplement: Multimedia Appendix 3 [file ai_v3i1e58275_app3.zip › 29.JPG]

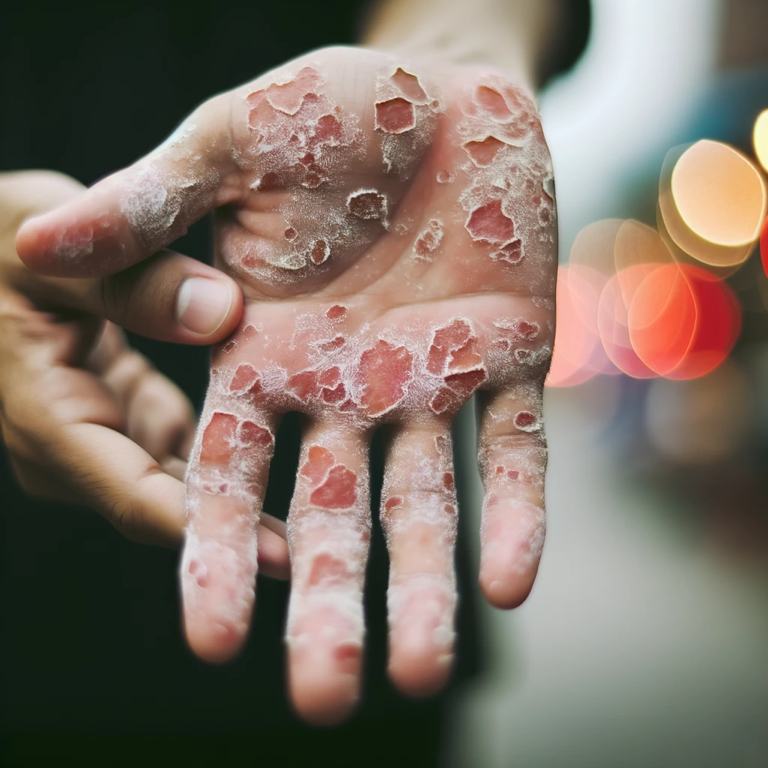

Supplement: Multimedia Appendix 3 [file ai_v3i1e58275_app3.zip › 02.PNG]

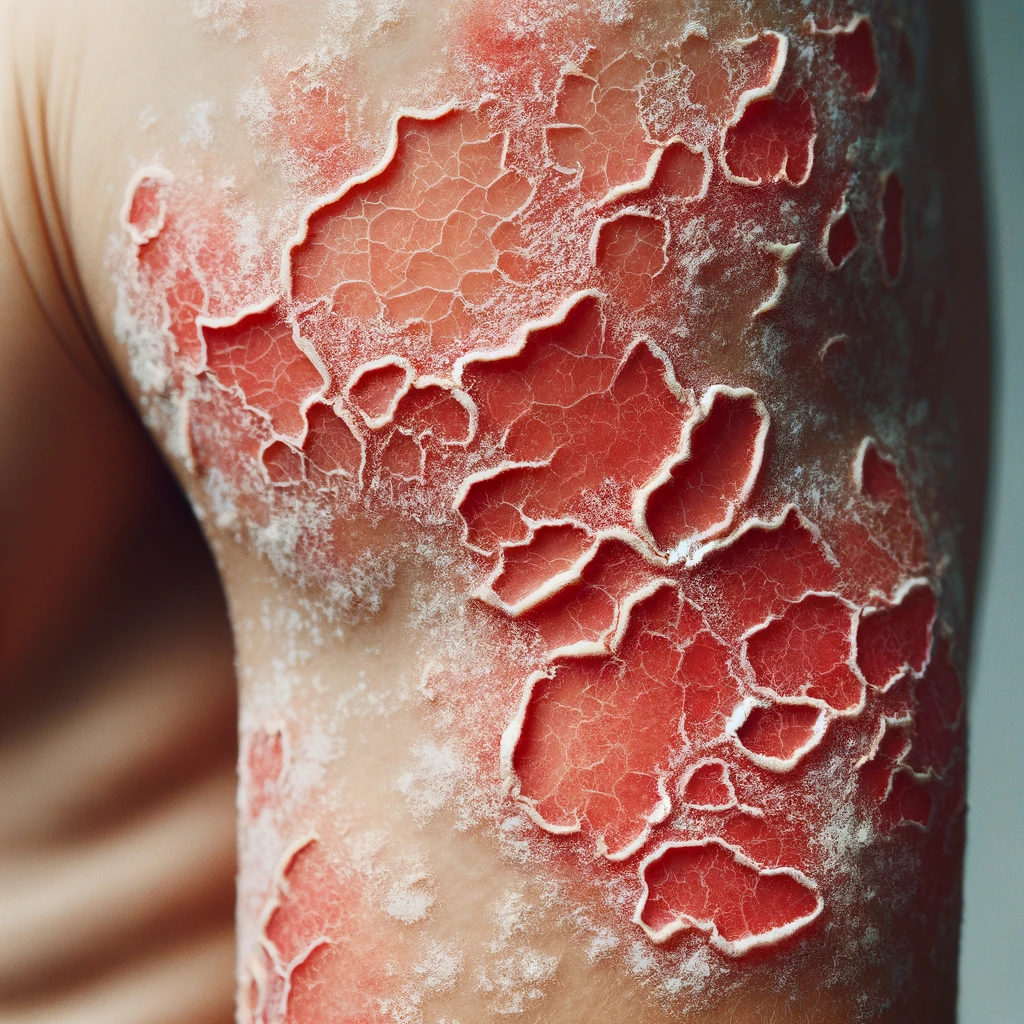

Supplement: Multimedia Appendix 3 [file ai_v3i1e58275_app3.zip › 91.WEBP]

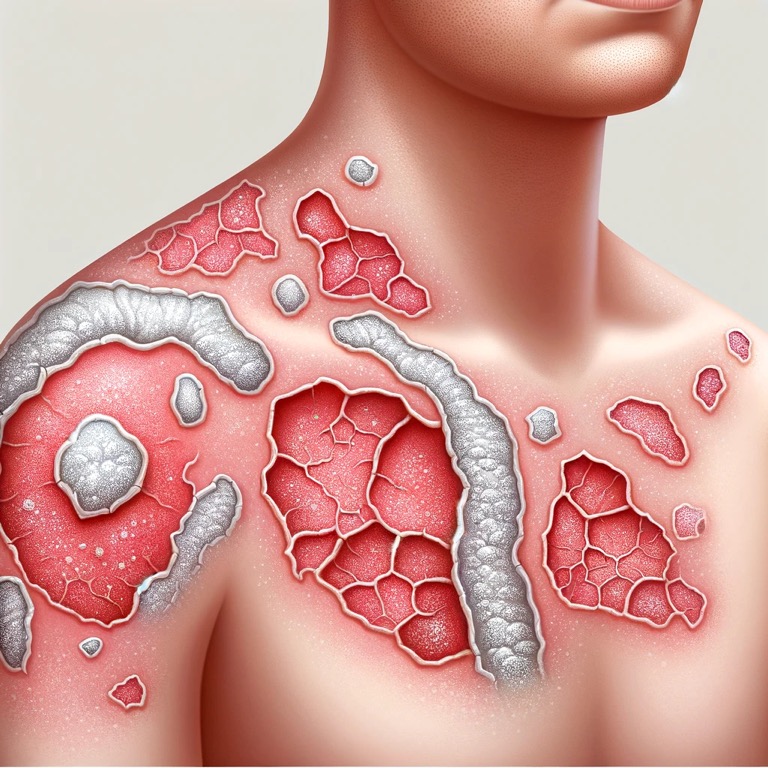

Supplement: Multimedia Appendix 3 [file ai_v3i1e58275_app3.zip › 51.jpeg]

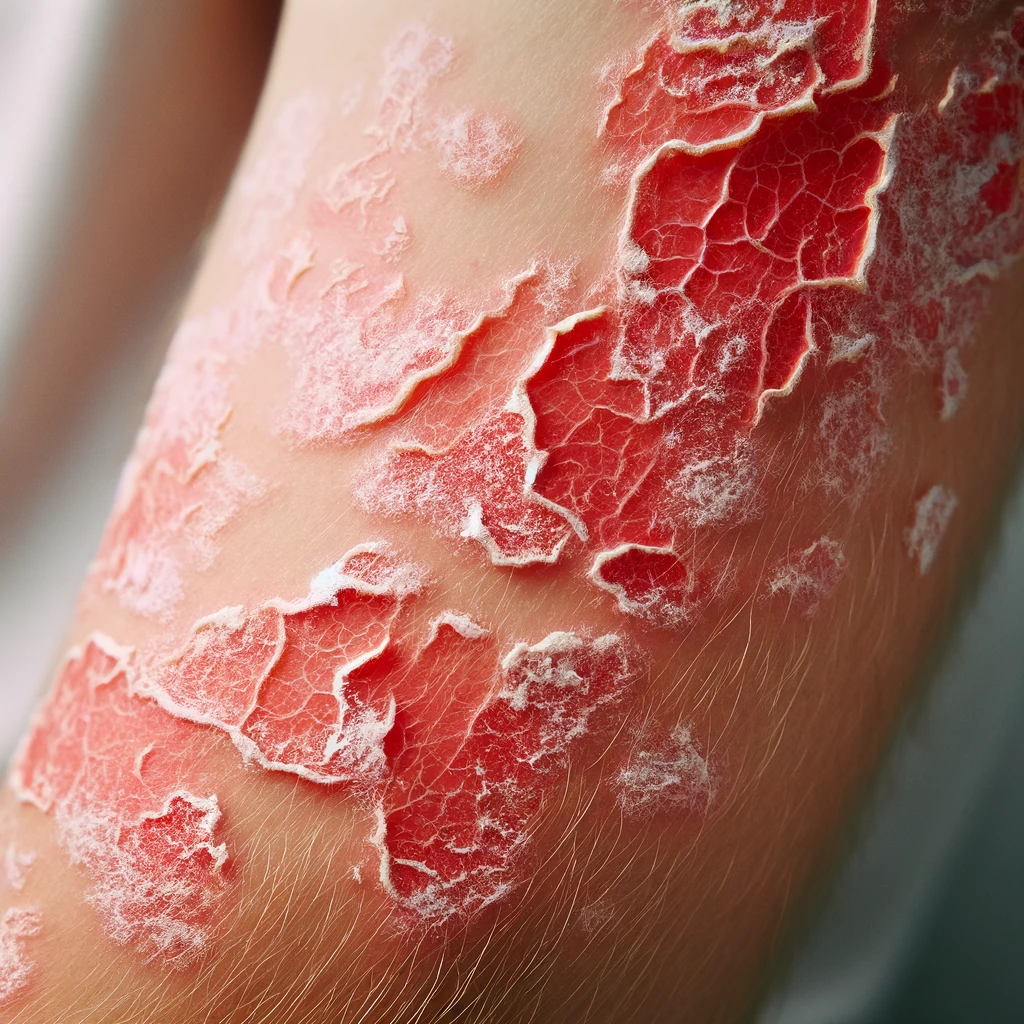

Supplement: Multimedia Appendix 3 [file ai_v3i1e58275_app3.zip › 65.WEBP]

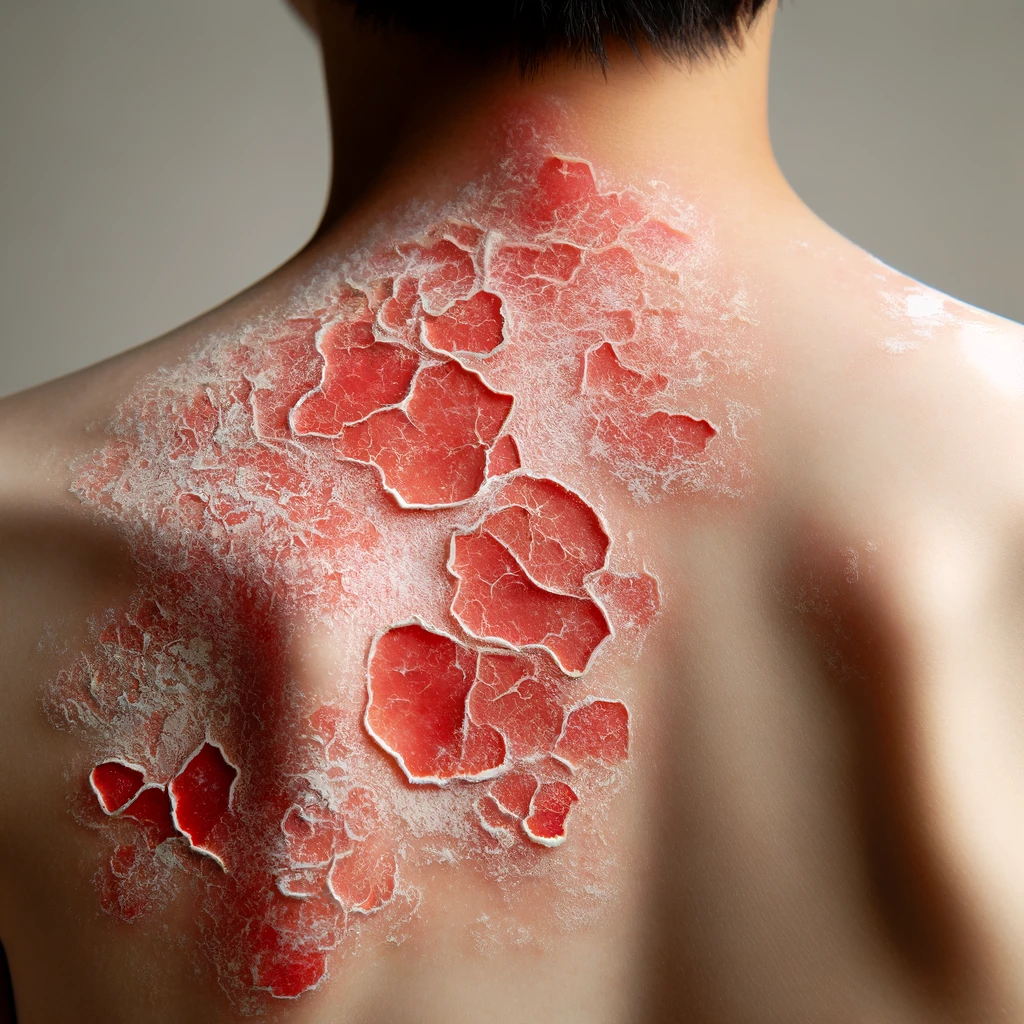

Supplement: Multimedia Appendix 3 [file ai_v3i1e58275_app3.zip › 79.WEBP]

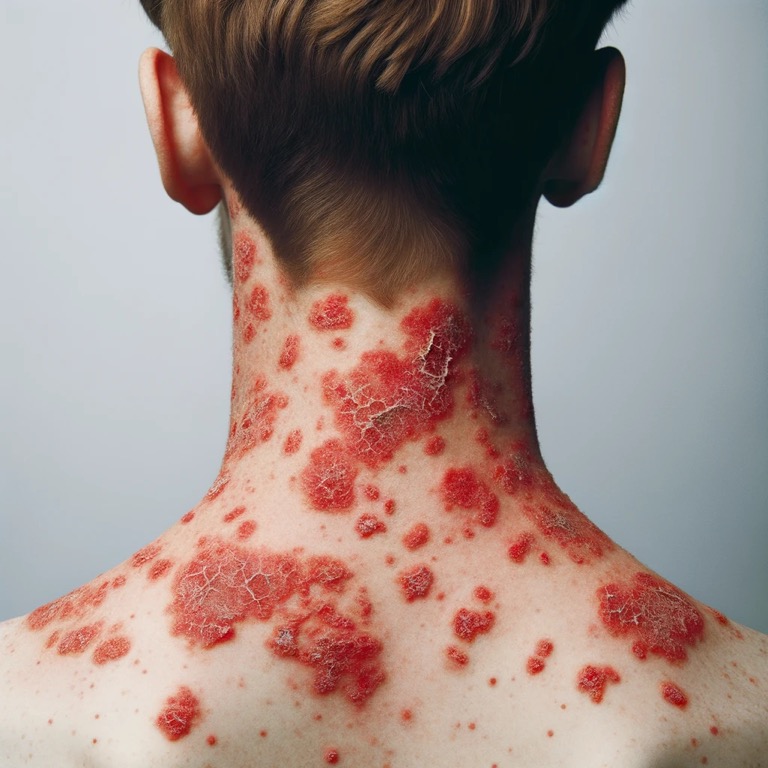

Supplement: Multimedia Appendix 3 [file ai_v3i1e58275_app3.zip › 22.JPG]

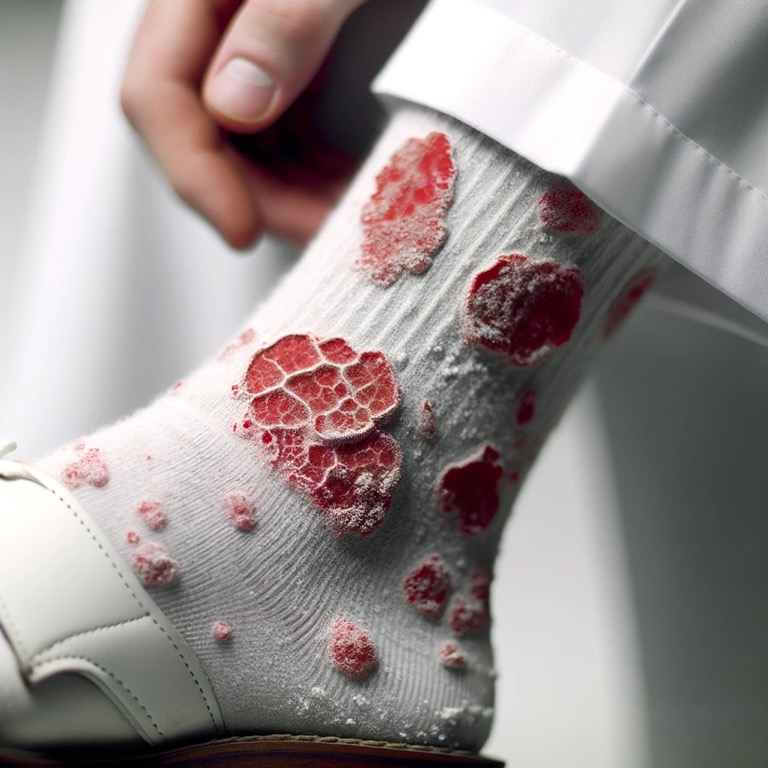

Supplement: Multimedia Appendix 3 [file ai_v3i1e58275_app3.zip › 19.JPG]

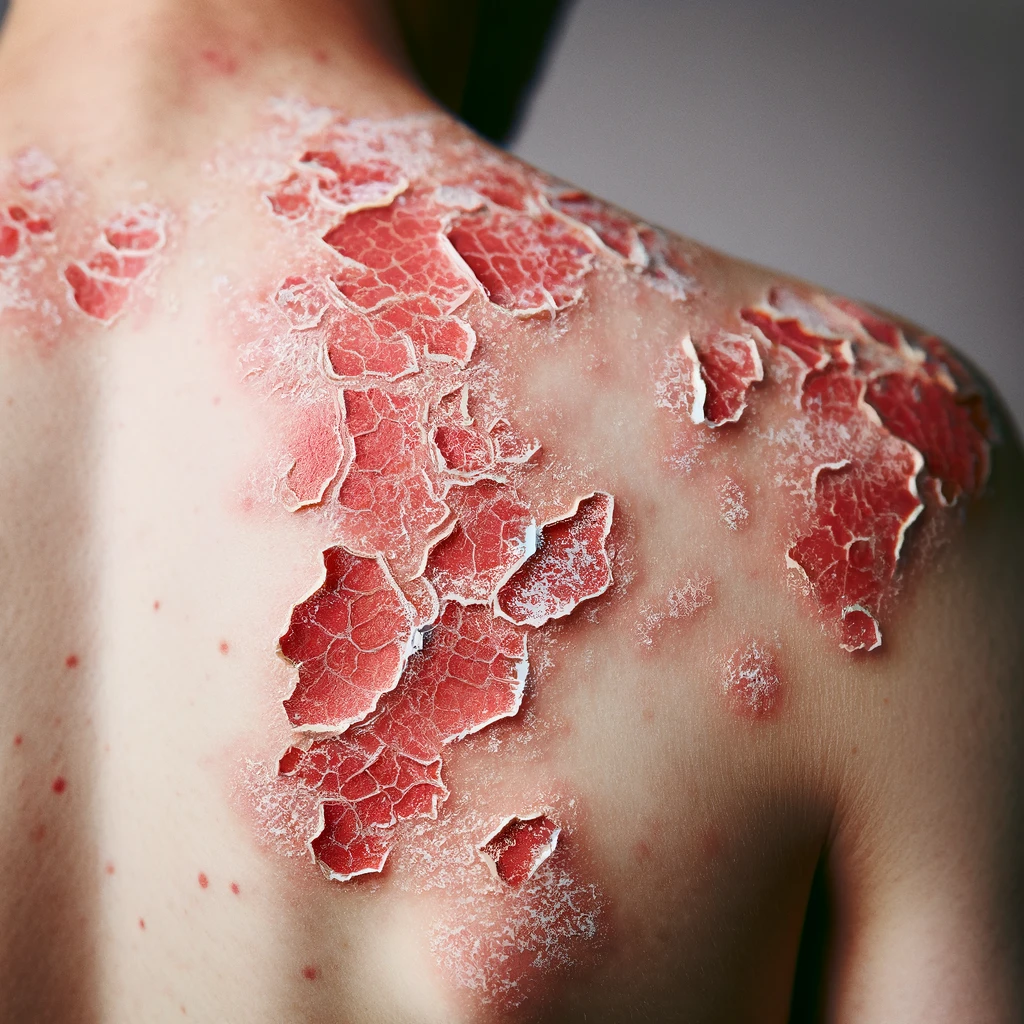

Supplement: Multimedia Appendix 3 [file ai_v3i1e58275_app3.zip › 78.WEBP]

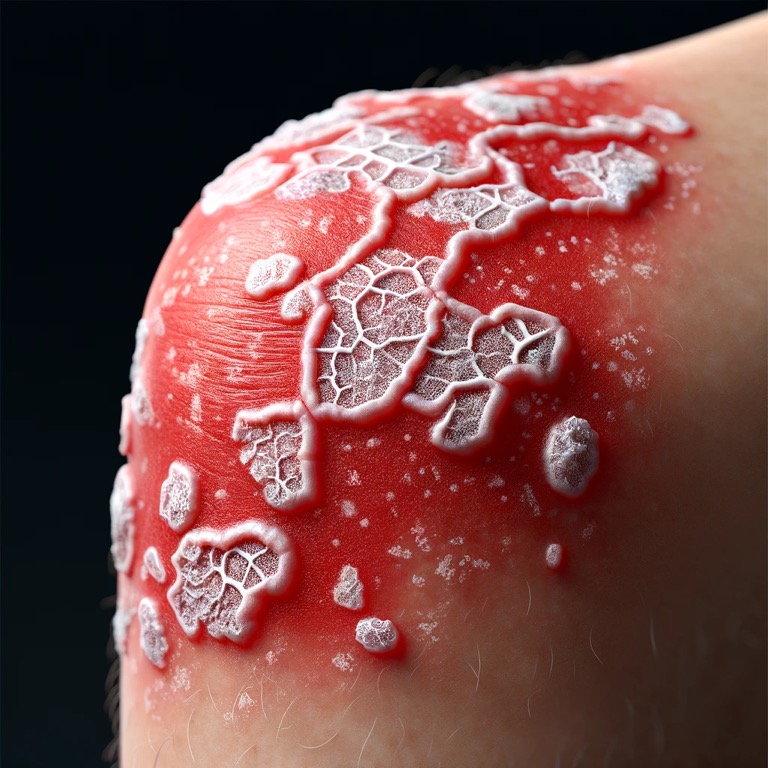

Supplement: Multimedia Appendix 3 [file ai_v3i1e58275_app3.zip › 52.jpeg]

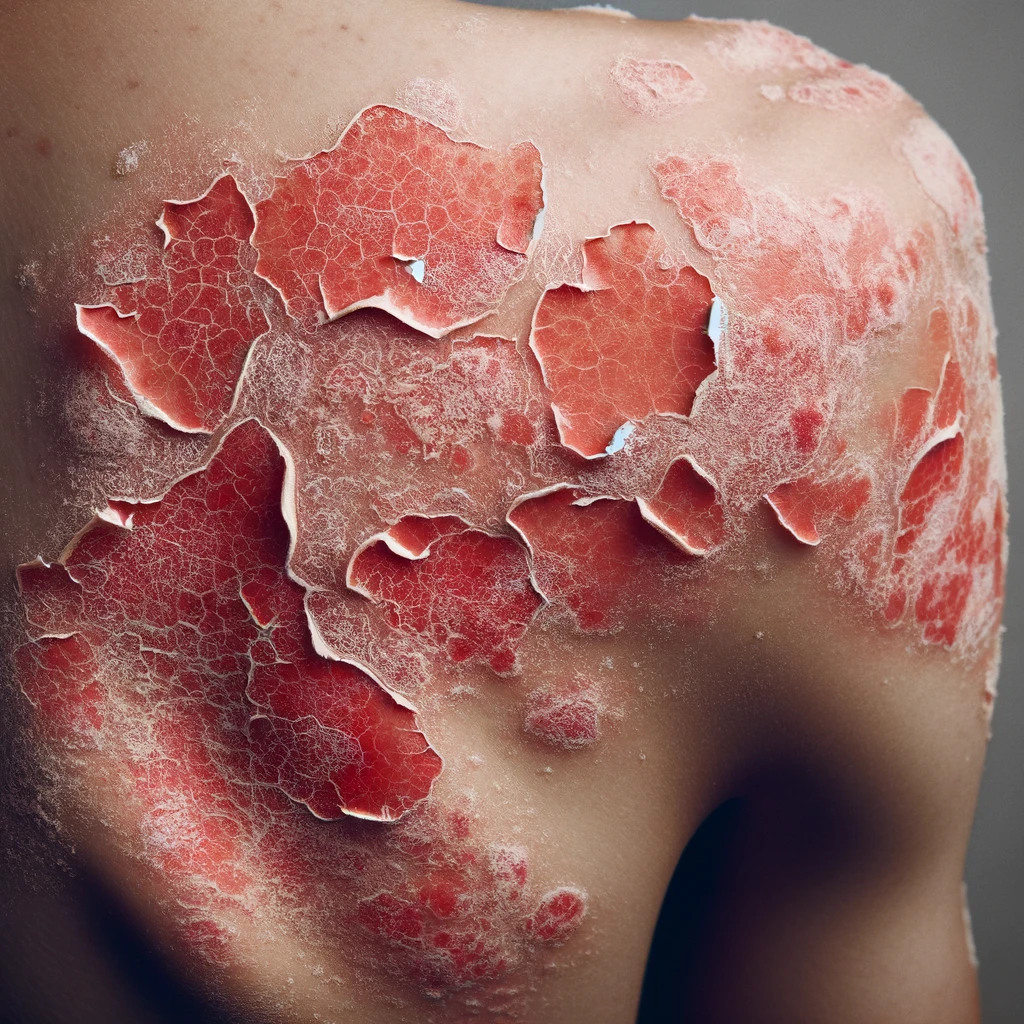

Supplement: Multimedia Appendix 3 [file ai_v3i1e58275_app3.zip › 85.WEBP]

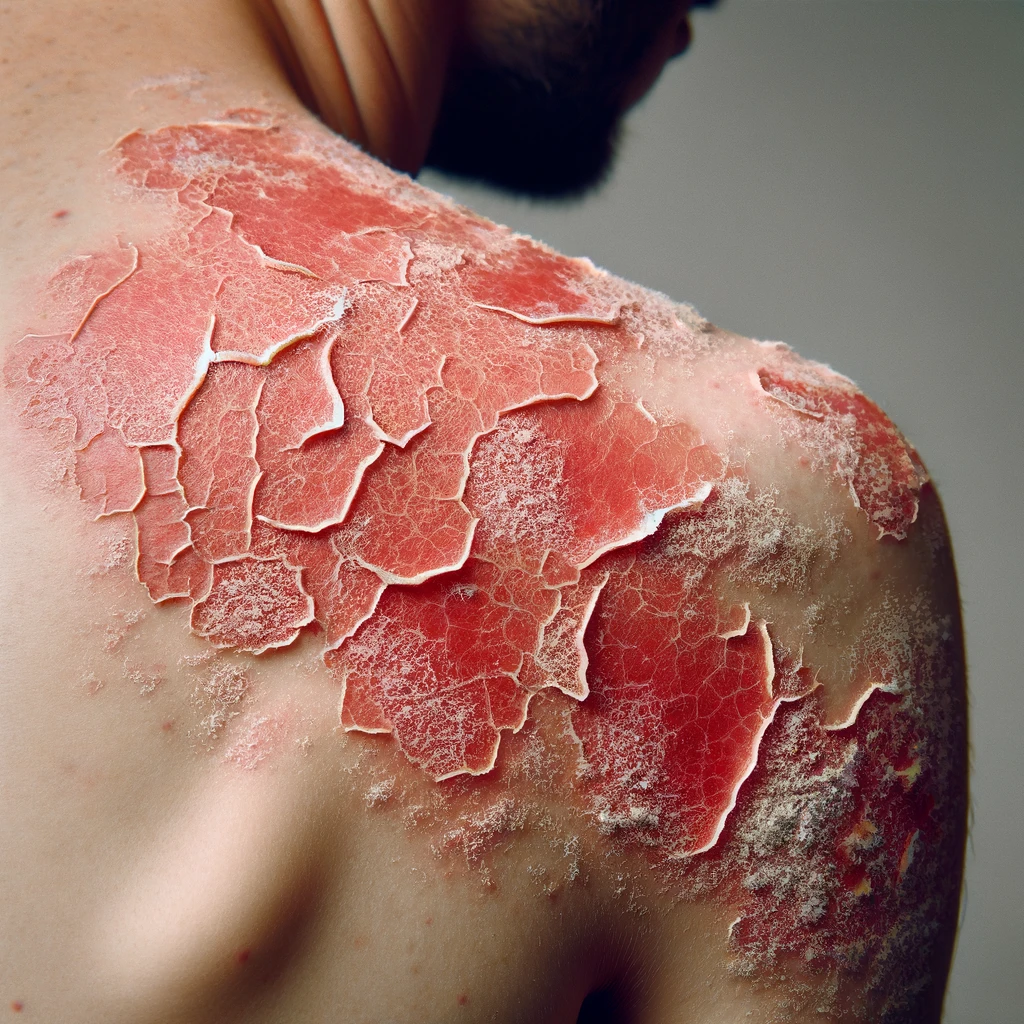

Supplement: Multimedia Appendix 3 [file ai_v3i1e58275_app3.zip › 73.WEBP]

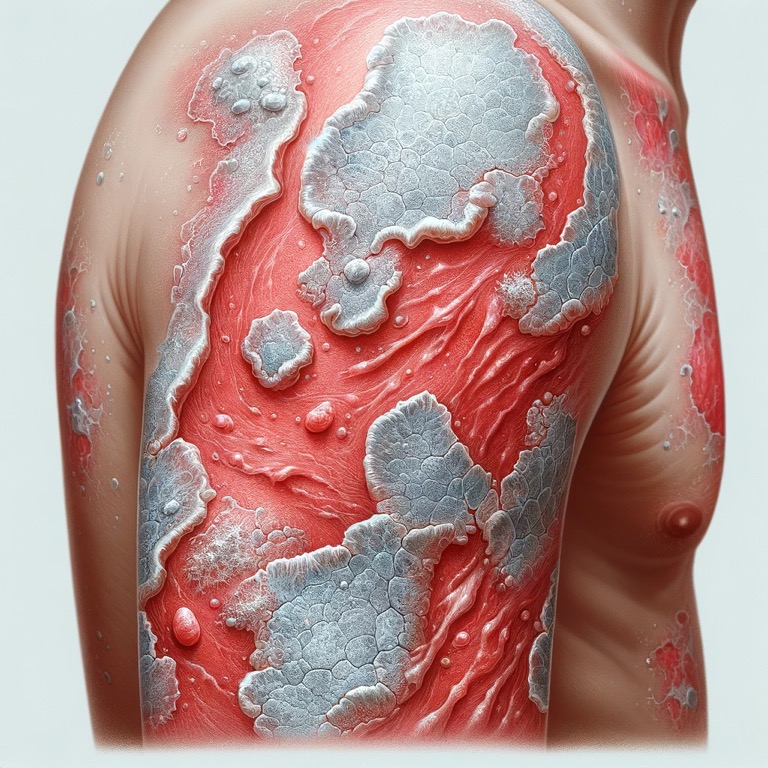

Supplement: Multimedia Appendix 3 [file ai_v3i1e58275_app3.zip › 46.jpeg]

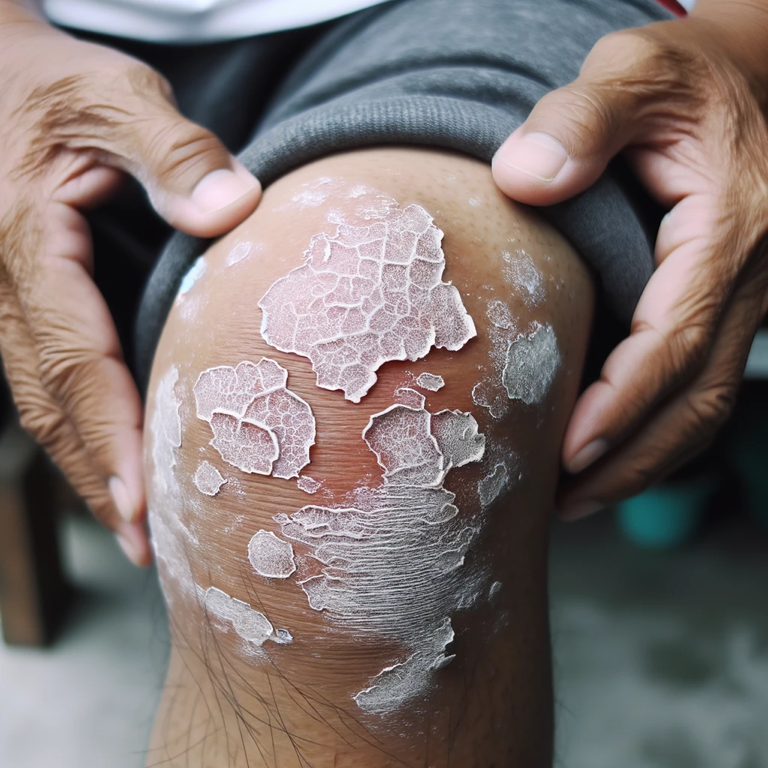

Supplement: Multimedia Appendix 3 [file ai_v3i1e58275_app3.zip › 13.PNG]

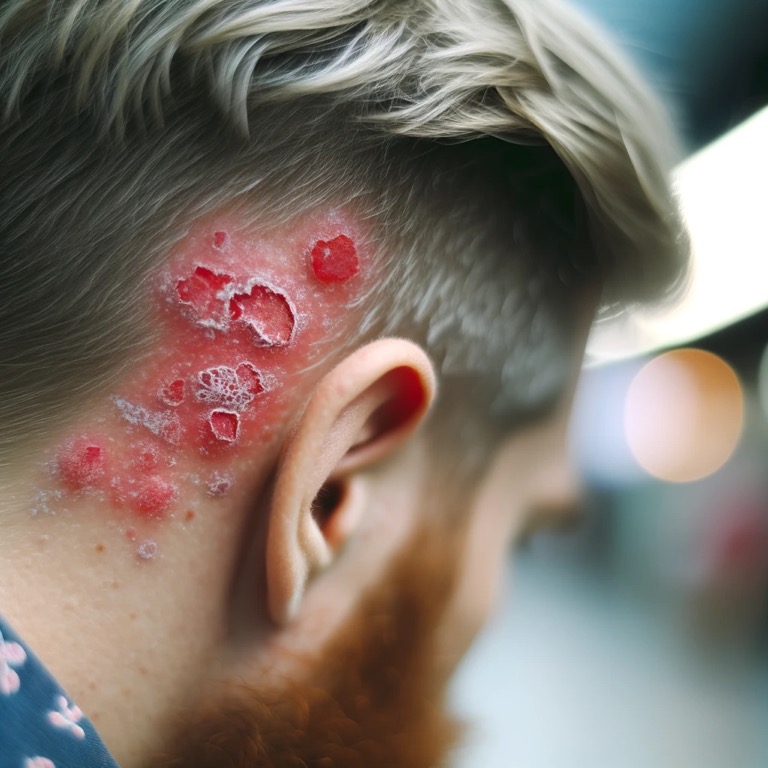

Supplement: Multimedia Appendix 3 [file ai_v3i1e58275_app3.zip › 24.JPG]

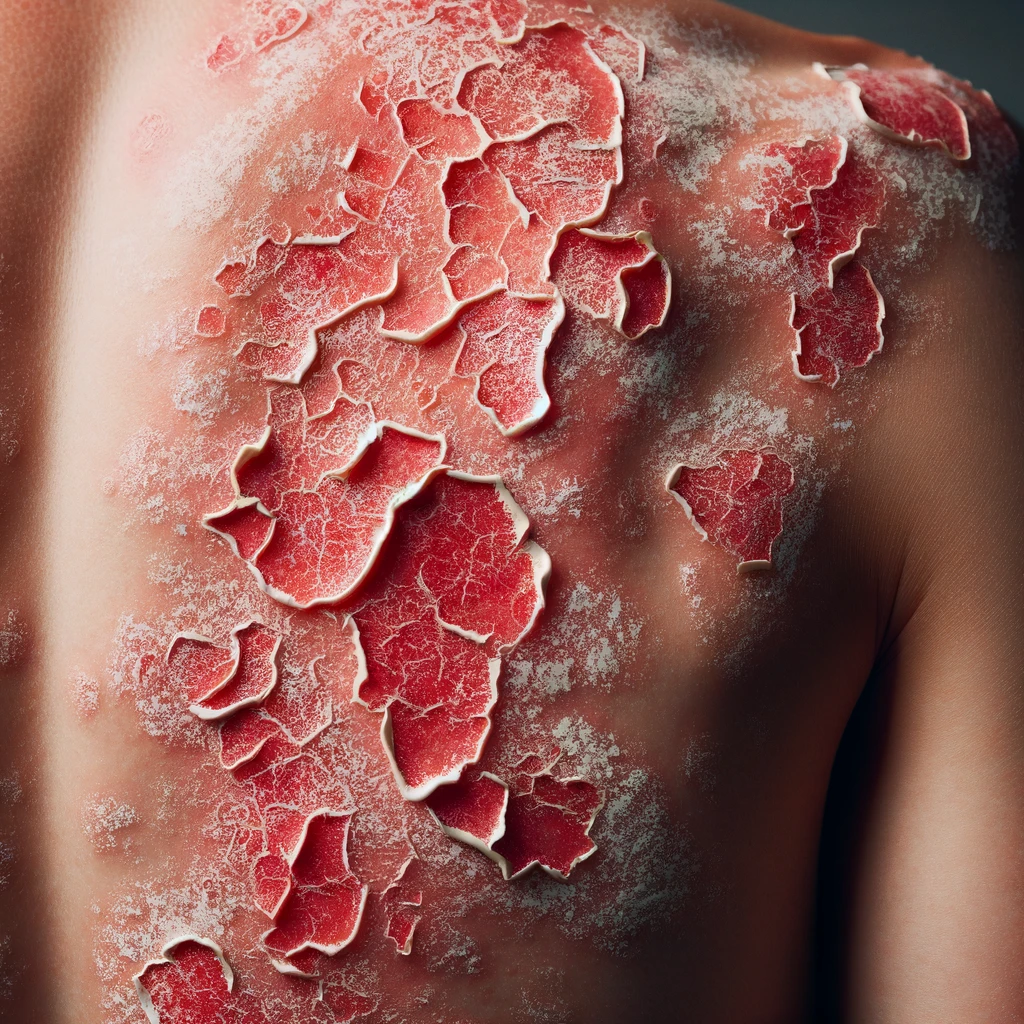

Supplement: Multimedia Appendix 3 [file ai_v3i1e58275_app3.zip › 88.WEBP]

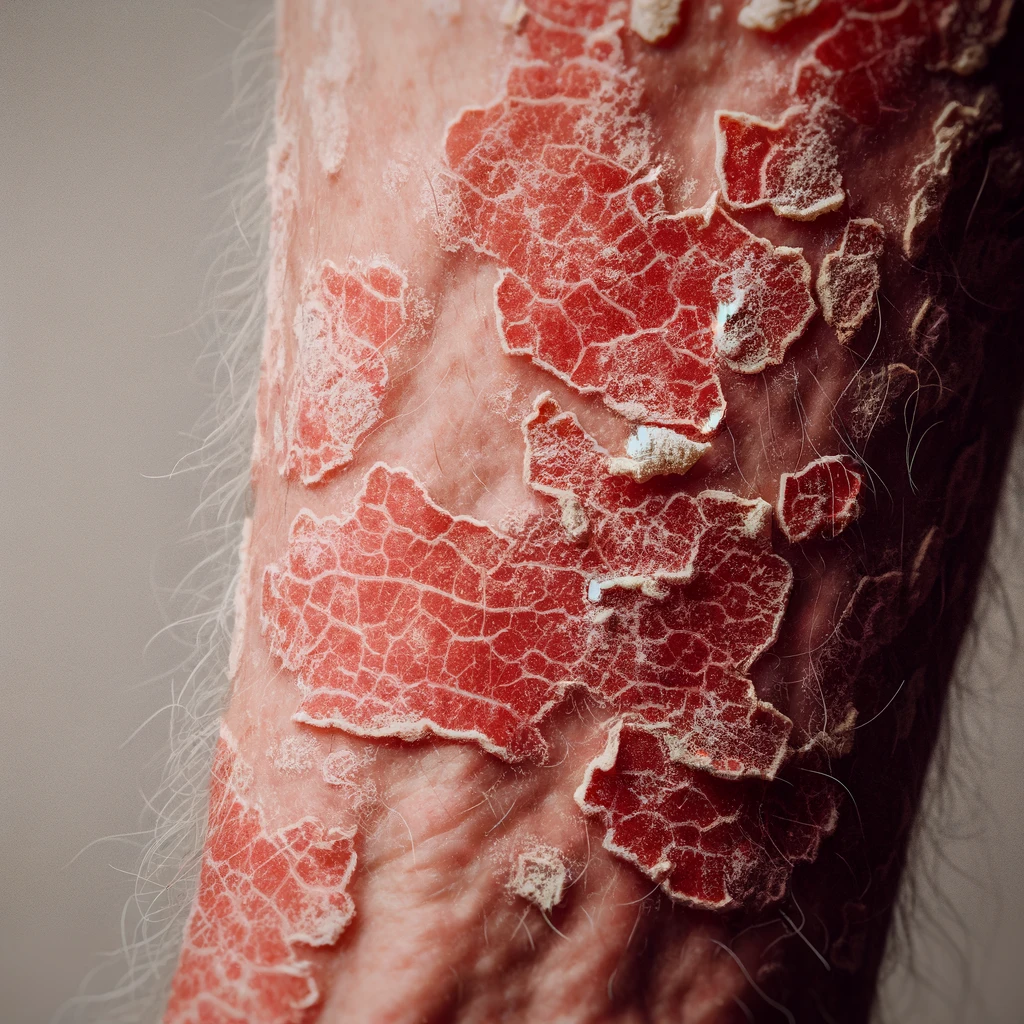

Supplement: Multimedia Appendix 3 [file ai_v3i1e58275_app3.zip › 94.WEBP]

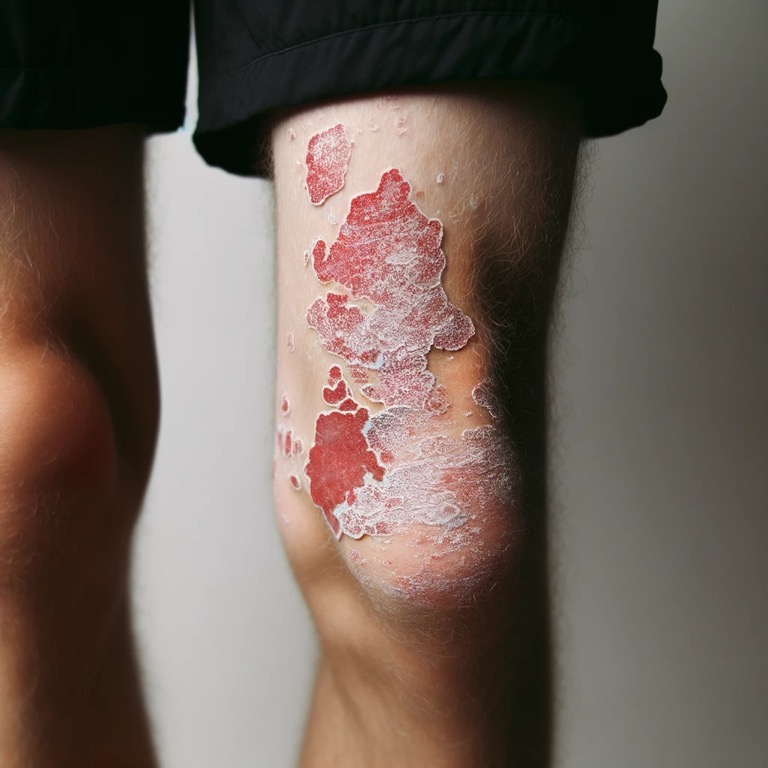

Supplement: Multimedia Appendix 3 [file ai_v3i1e58275_app3.zip › 20.JPG]

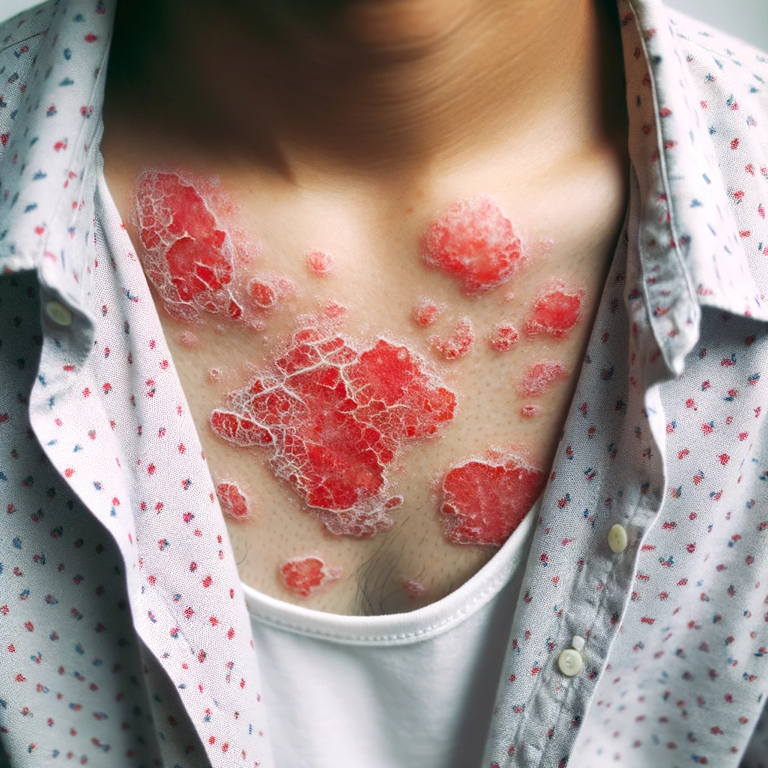

Supplement: Multimedia Appendix 3 [file ai_v3i1e58275_app3.zip › 14.PNG]

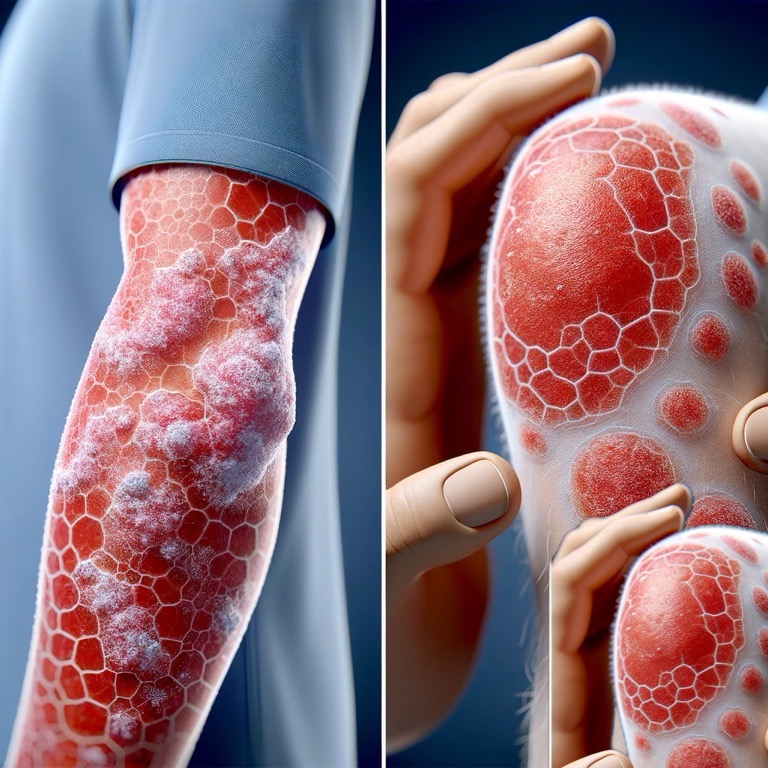

Supplement: Multimedia Appendix 3 [file ai_v3i1e58275_app3.zip › 37+38.jpeg]

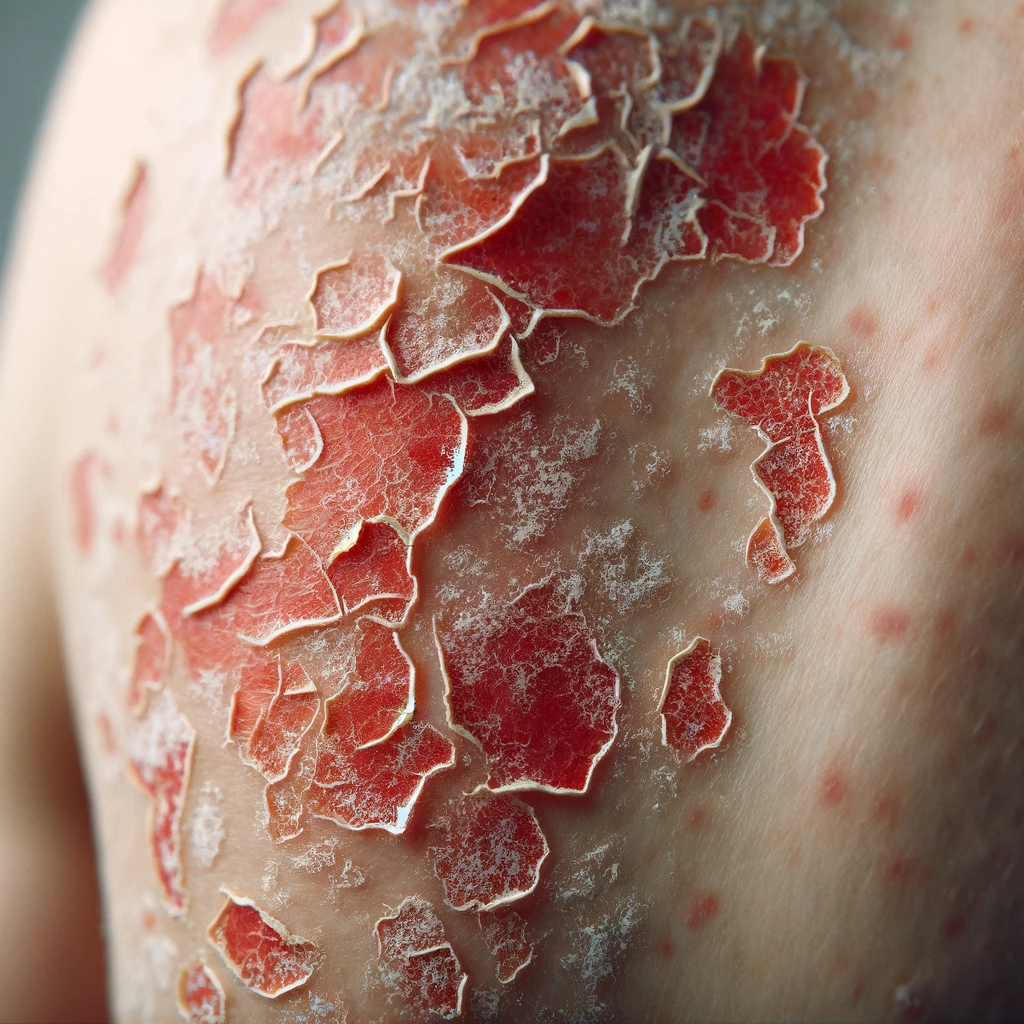

Supplement: Multimedia Appendix 3 [file ai_v3i1e58275_app3.zip › 69.WEBP]

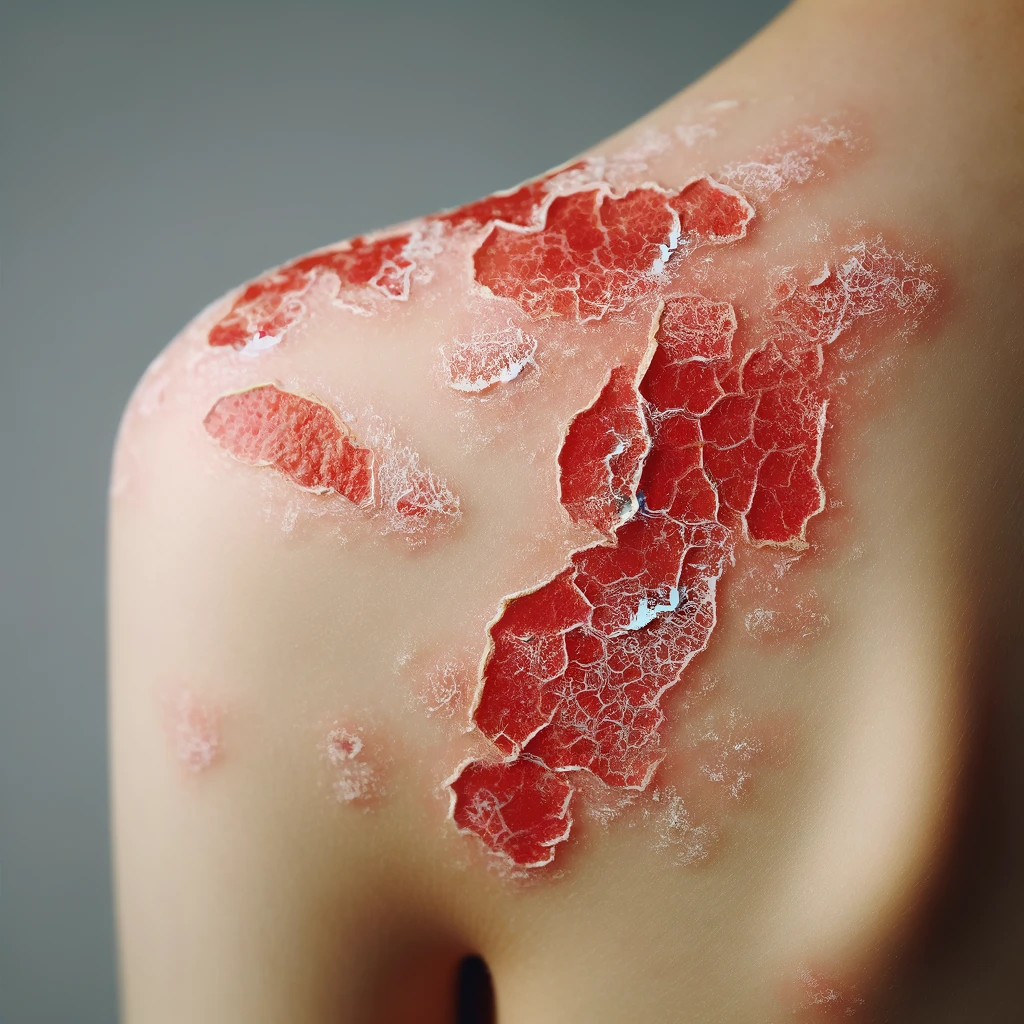

Supplement: Multimedia Appendix 3 [file ai_v3i1e58275_app3.zip › 74.WEBP]

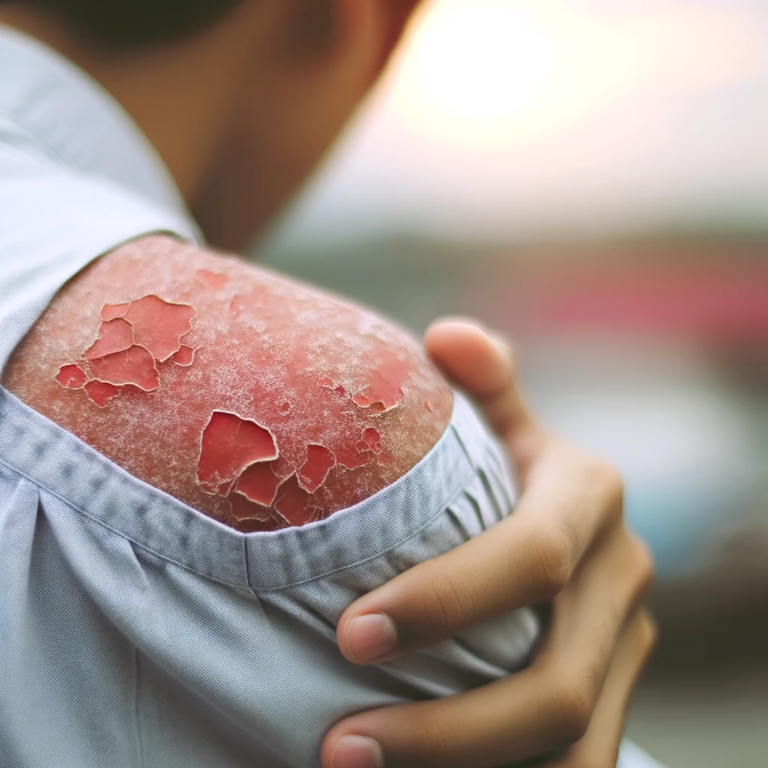

Supplement: Multimedia Appendix 3 [file ai_v3i1e58275_app3.zip › 10.PNG]

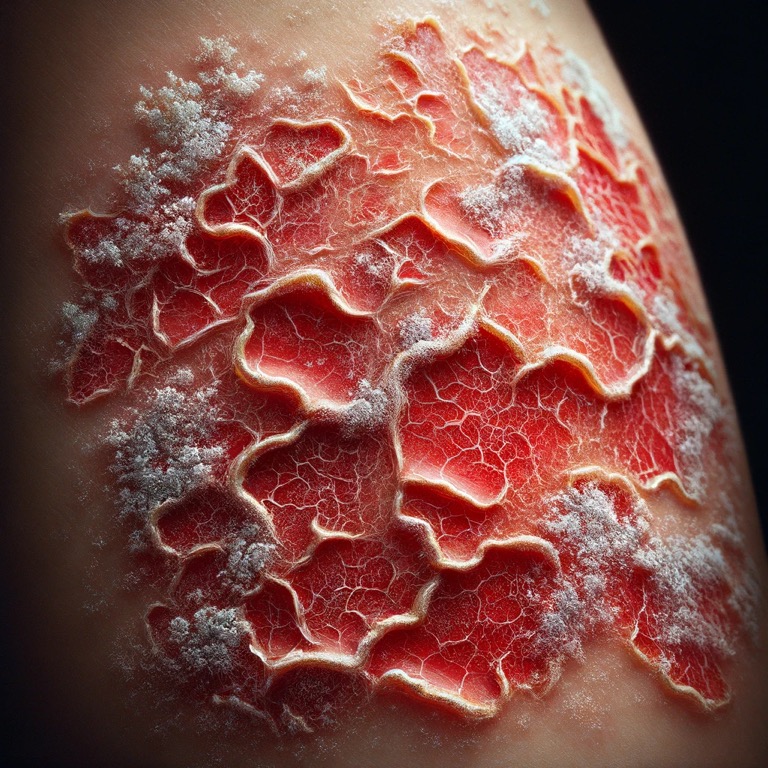

Supplement: Multimedia Appendix 3 [file ai_v3i1e58275_app3.zip › 96.JPG]

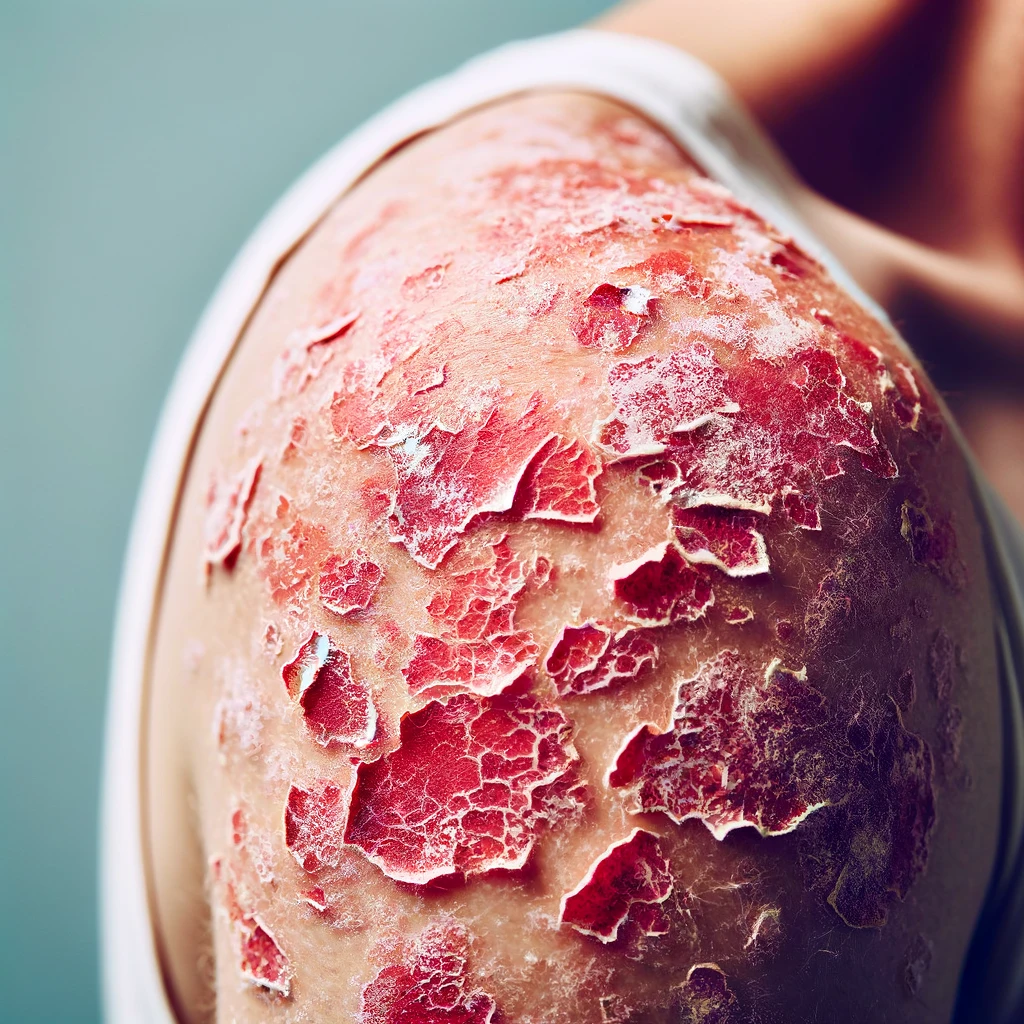

Supplement: Multimedia Appendix 3 [file ai_v3i1e58275_app3.zip › 68.WEBP]

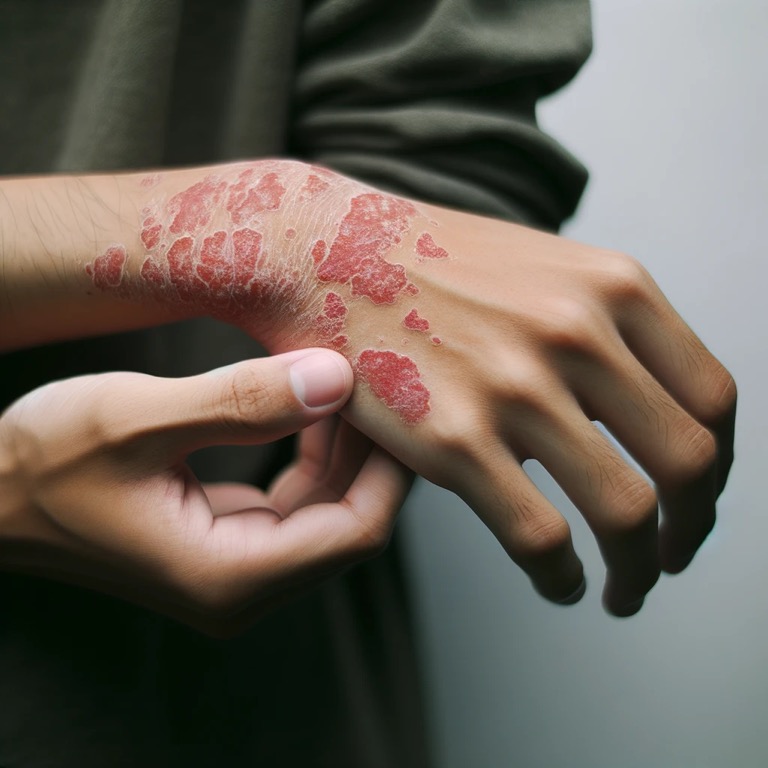

Supplement: Multimedia Appendix 3 [file ai_v3i1e58275_app3.zip › 21.JPG]

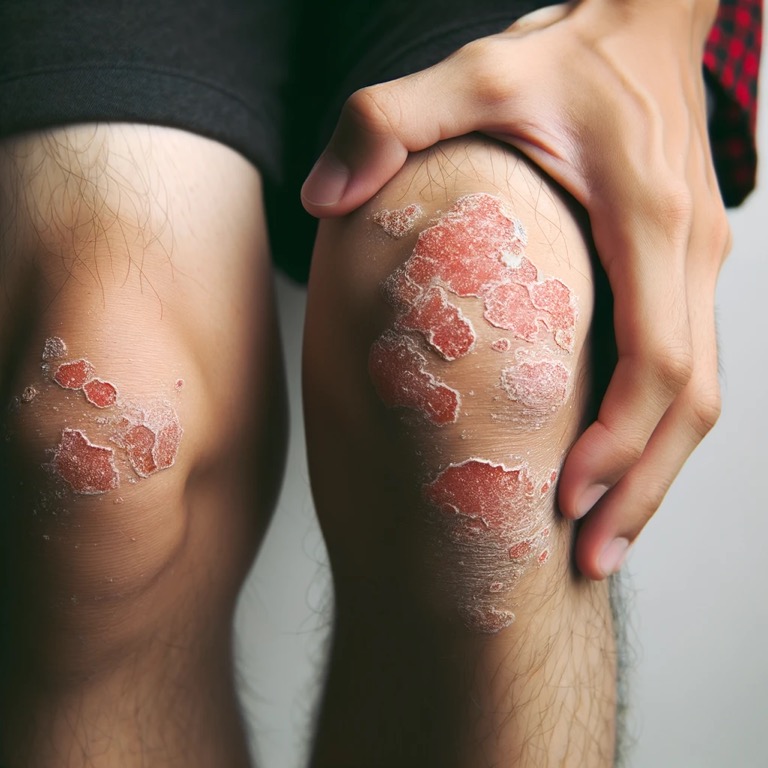

Supplement: Multimedia Appendix 3 [file ai_v3i1e58275_app3.zip › 17.JPG]

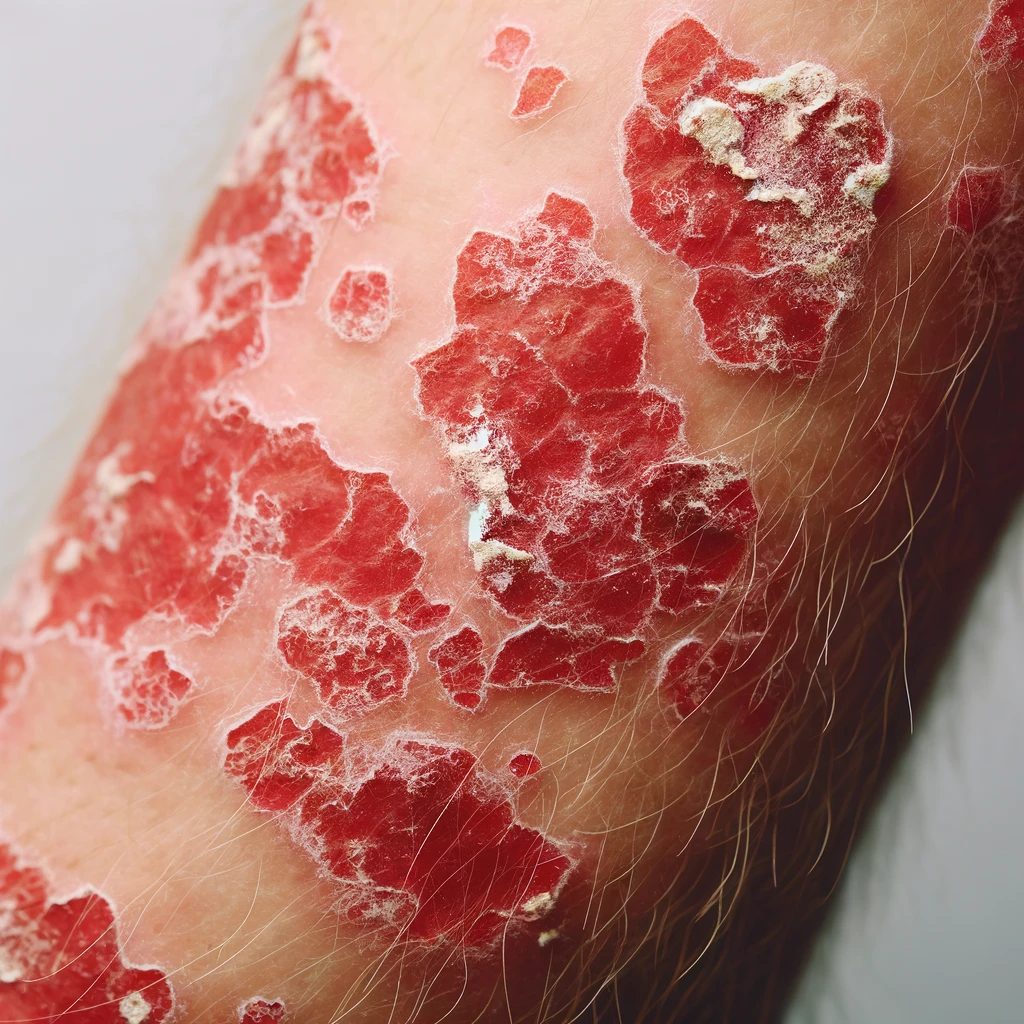

Supplement: Multimedia Appendix 3 [file ai_v3i1e58275_app3.zip › 93.WEBP]

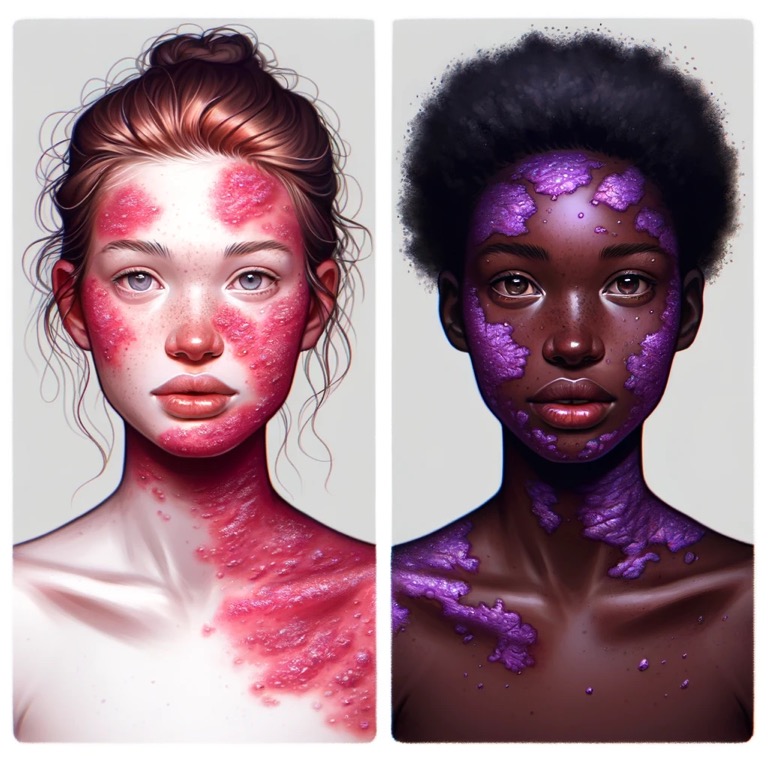

Supplement: Multimedia Appendix 3 [file ai_v3i1e58275_app3.zip › 35+36.jpeg]

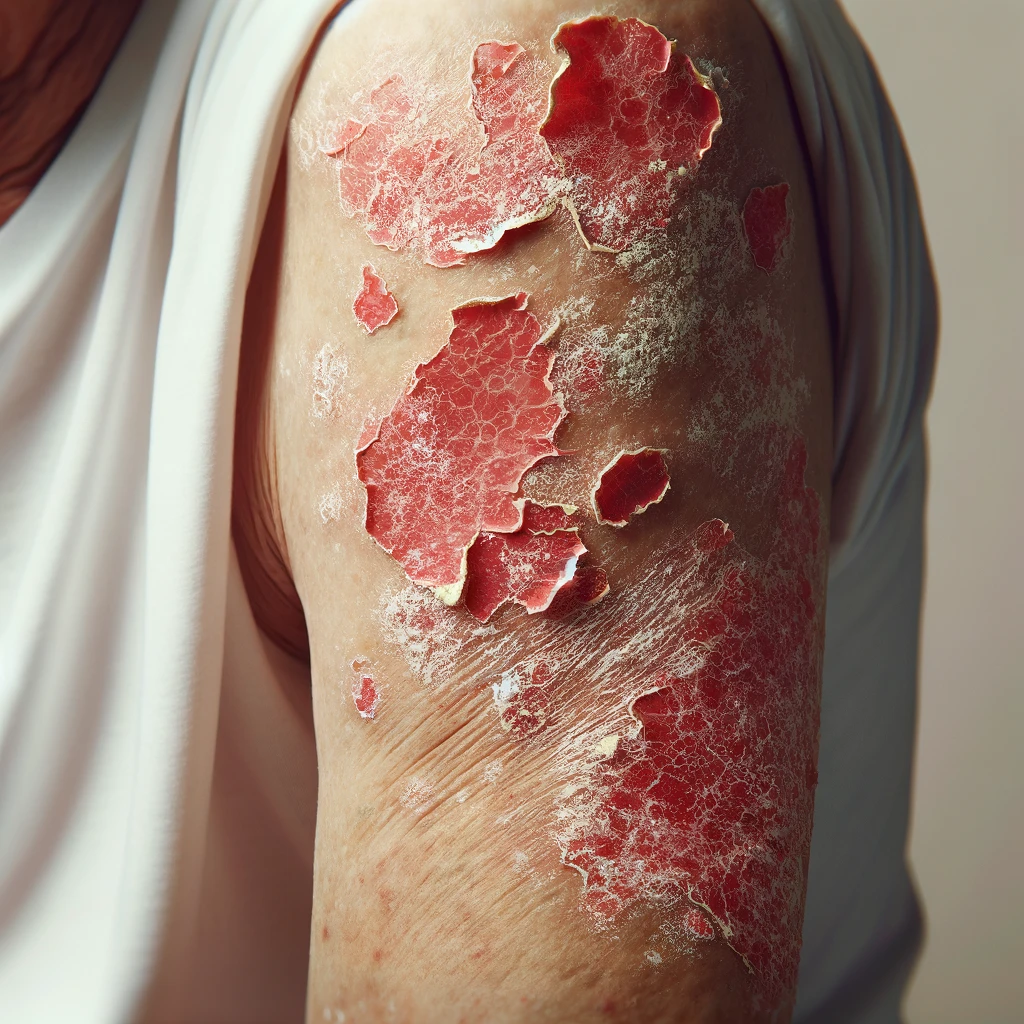

Supplement: Multimedia Appendix 3 [file ai_v3i1e58275_app3.zip › 81.WEBP]

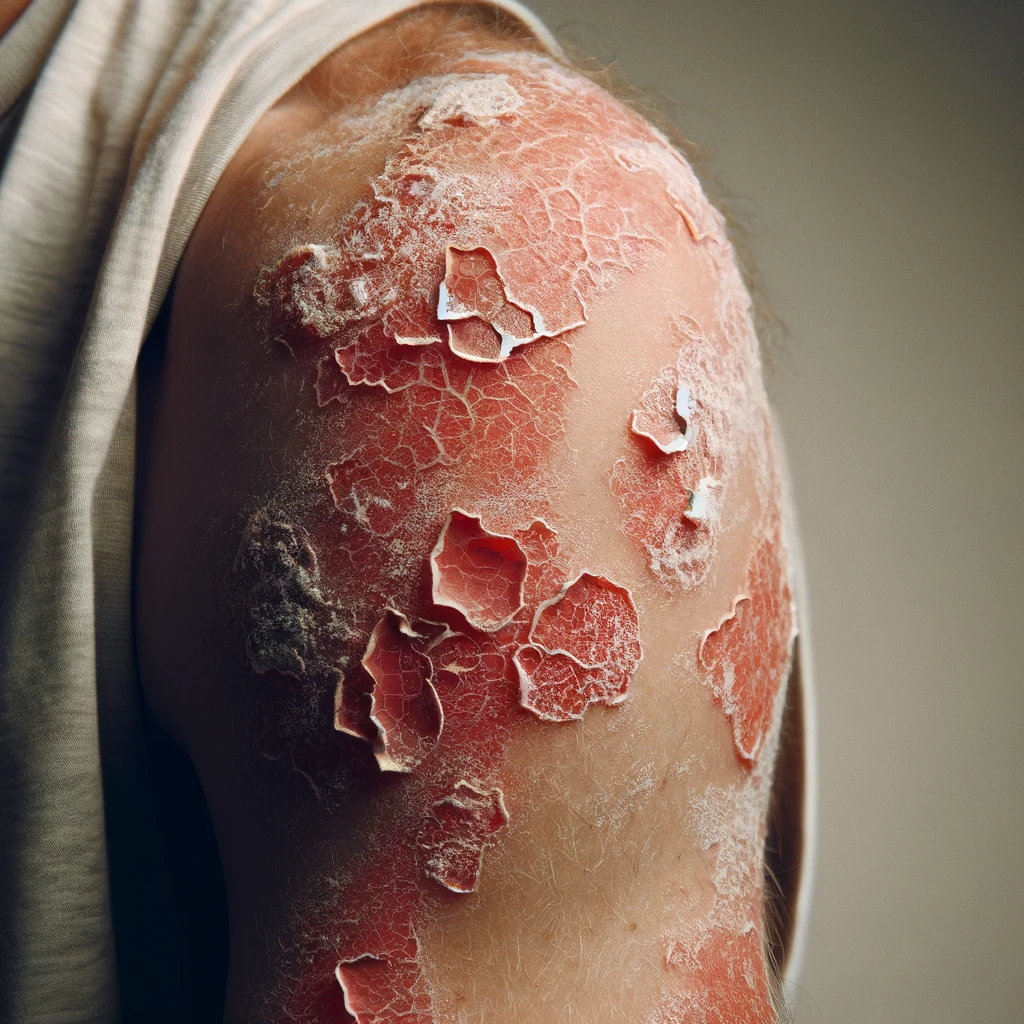

Supplement: Multimedia Appendix 3 [file ai_v3i1e58275_app3.zip › 66.WEBP]

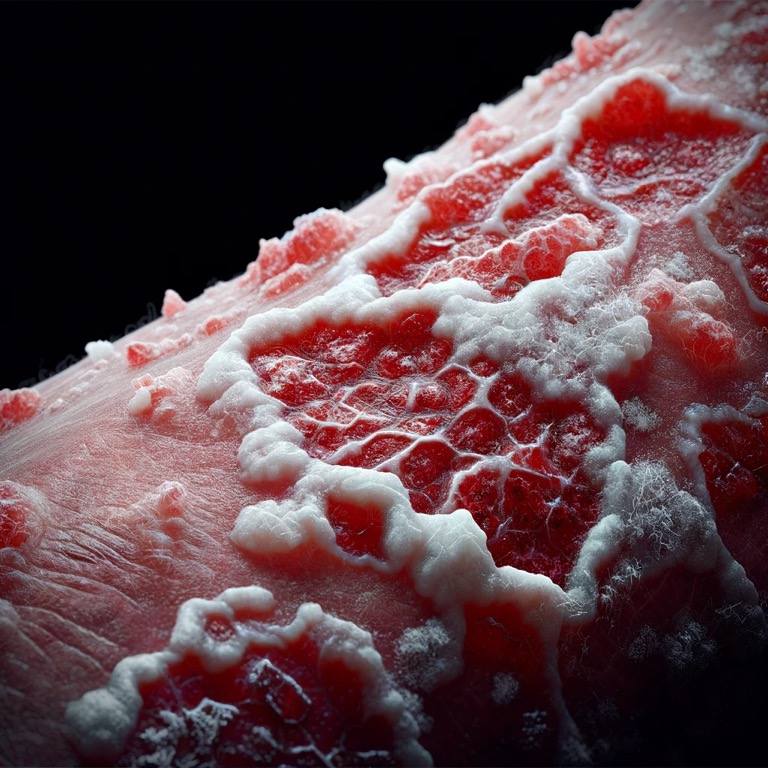

Supplement: Multimedia Appendix 3 [file ai_v3i1e58275_app3.zip › 42.jpeg]

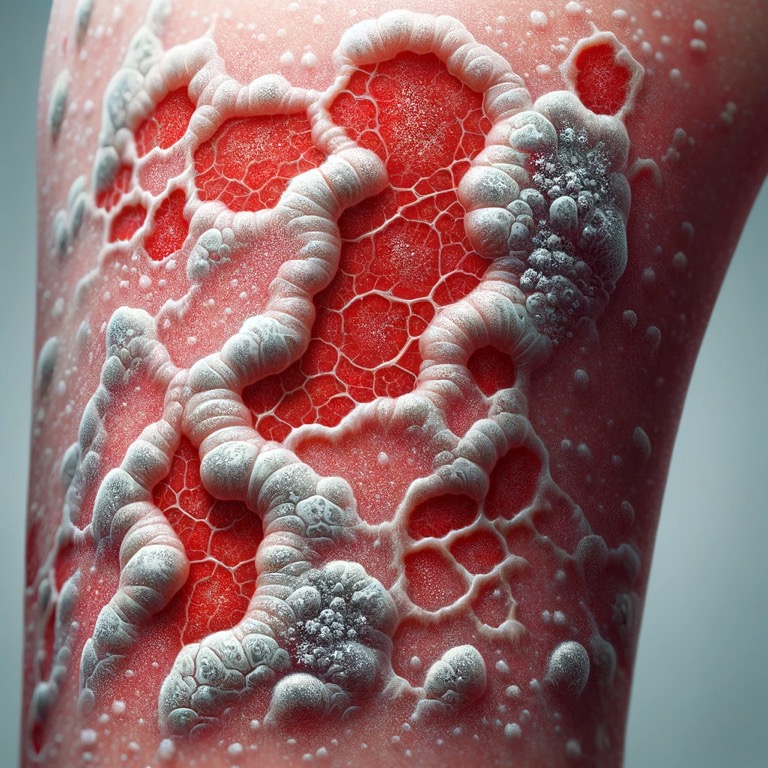

Supplement: Multimedia Appendix 3 [file ai_v3i1e58275_app3.zip › 48.jpeg]

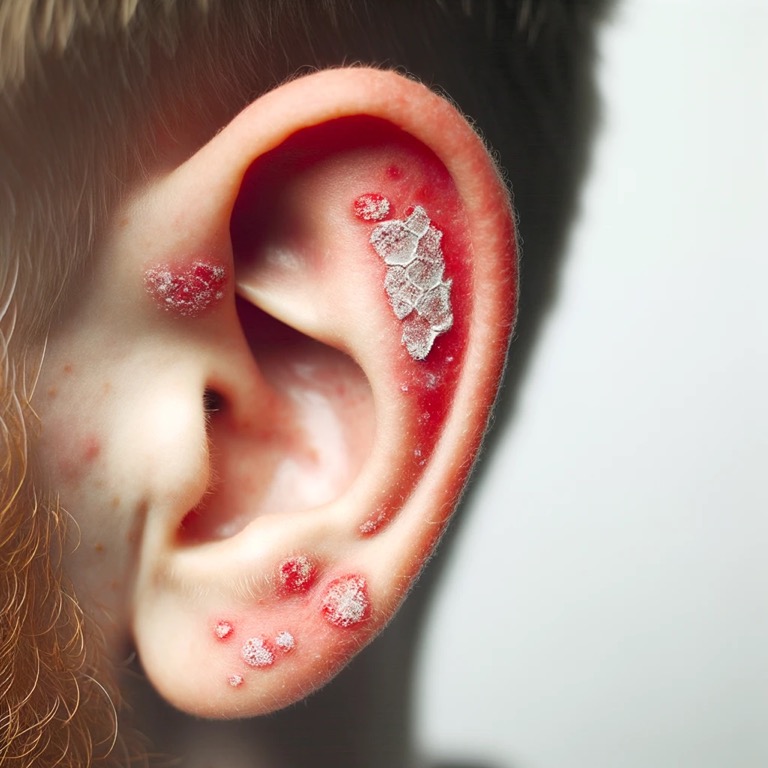

Supplement: Multimedia Appendix 3 [file ai_v3i1e58275_app3.zip › 16.JPG]

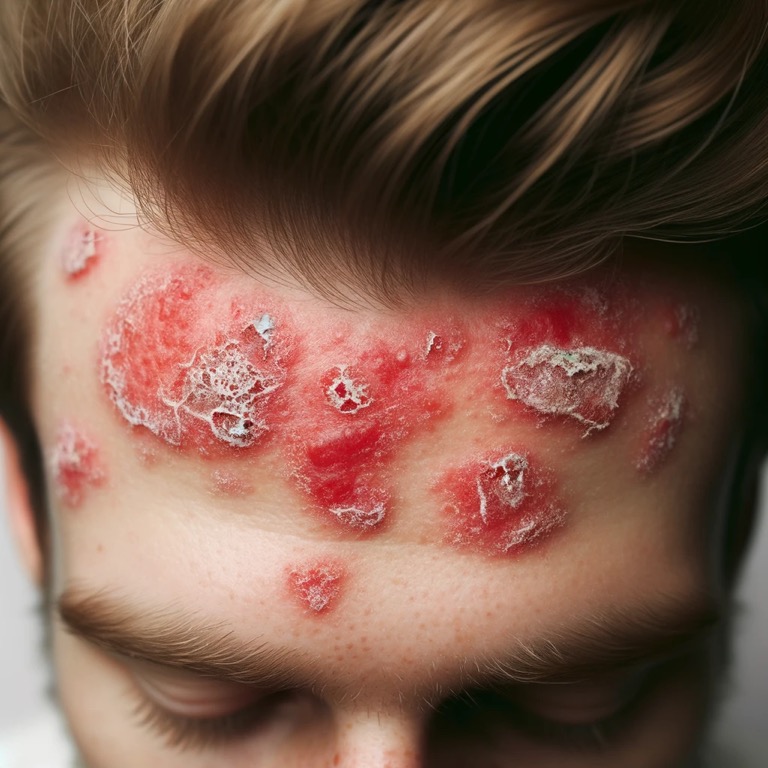

Supplement: Multimedia Appendix 3 [file ai_v3i1e58275_app3.zip › 08.JPG]

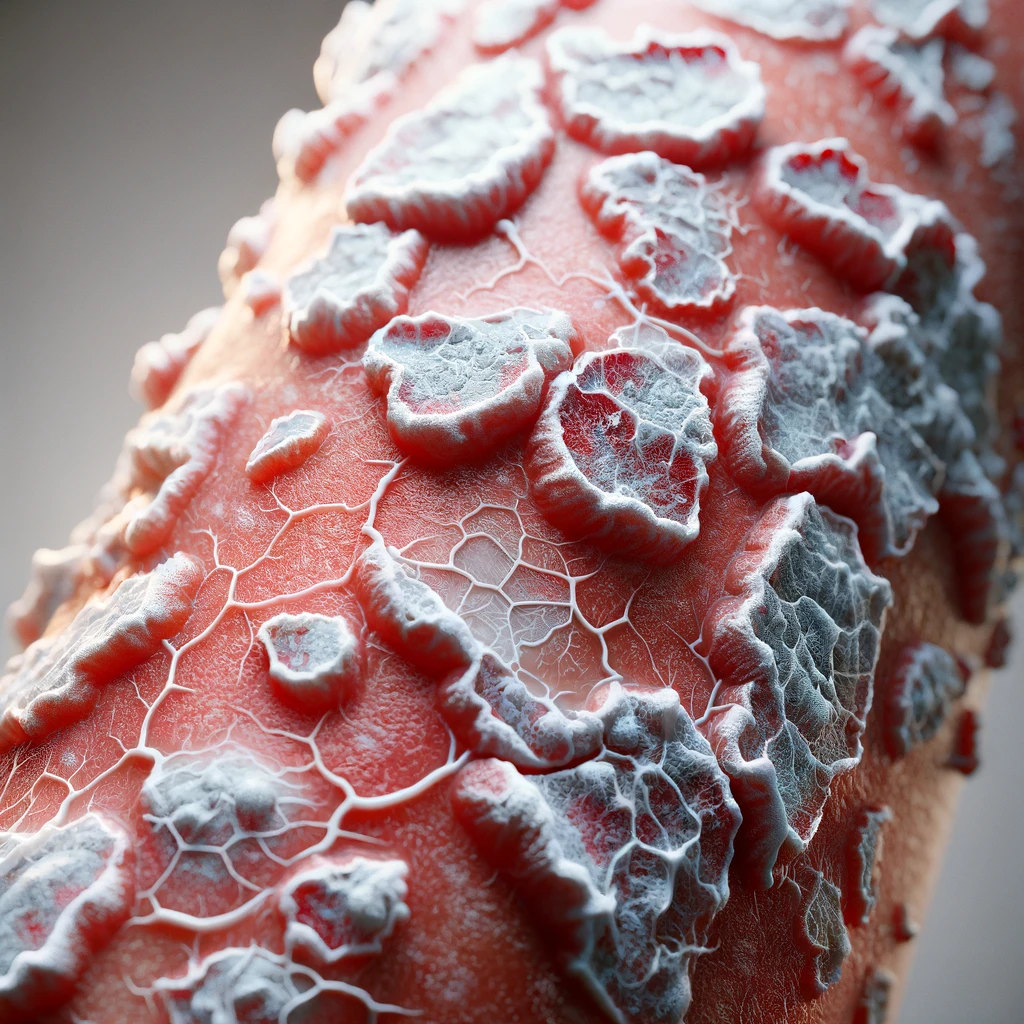

Supplement: Multimedia Appendix 3 [file ai_v3i1e58275_app3.zip › 59.WEBP]

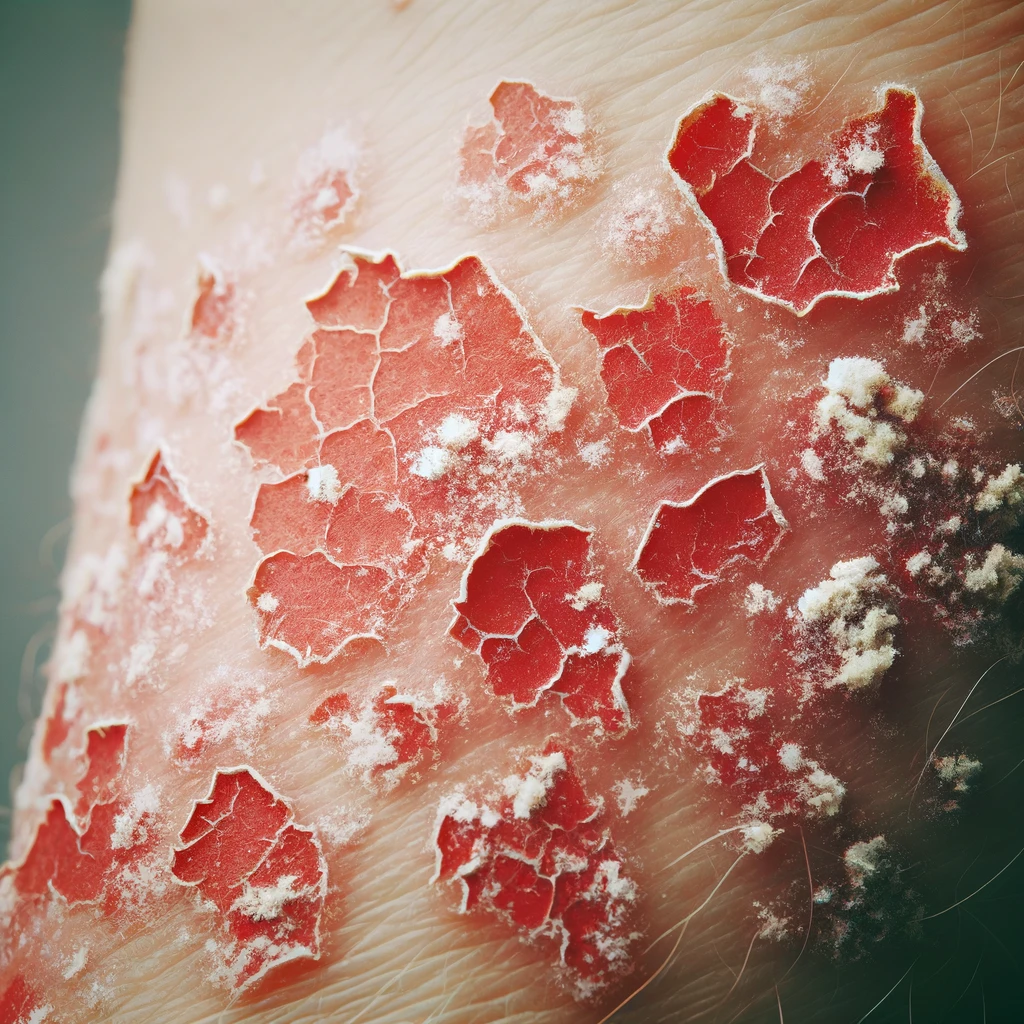

Supplement: Multimedia Appendix 3 [file ai_v3i1e58275_app3.zip › 70.WEBP]

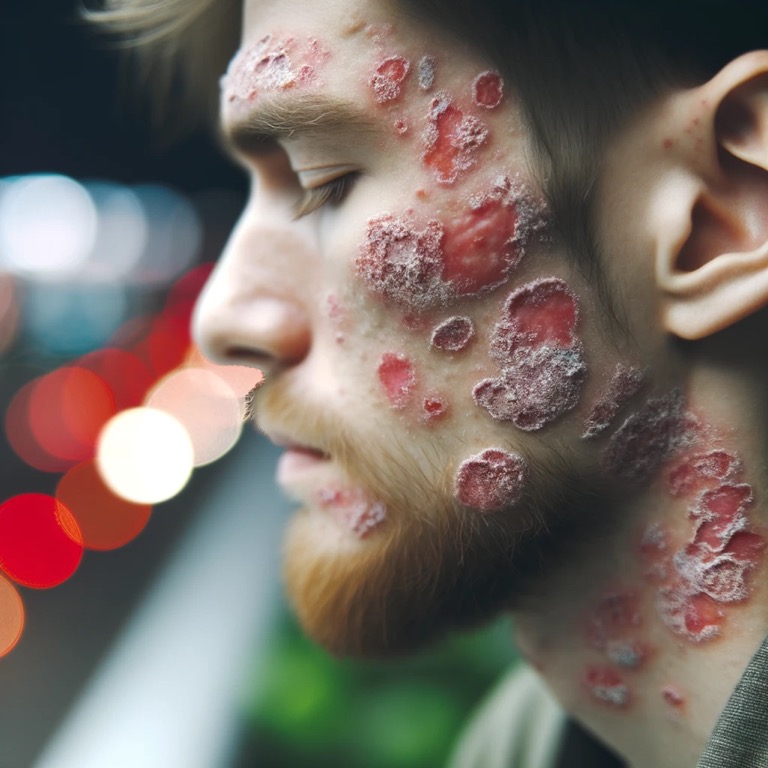

Supplement: Multimedia Appendix 3 [file ai_v3i1e58275_app3.zip › 15.JPG]

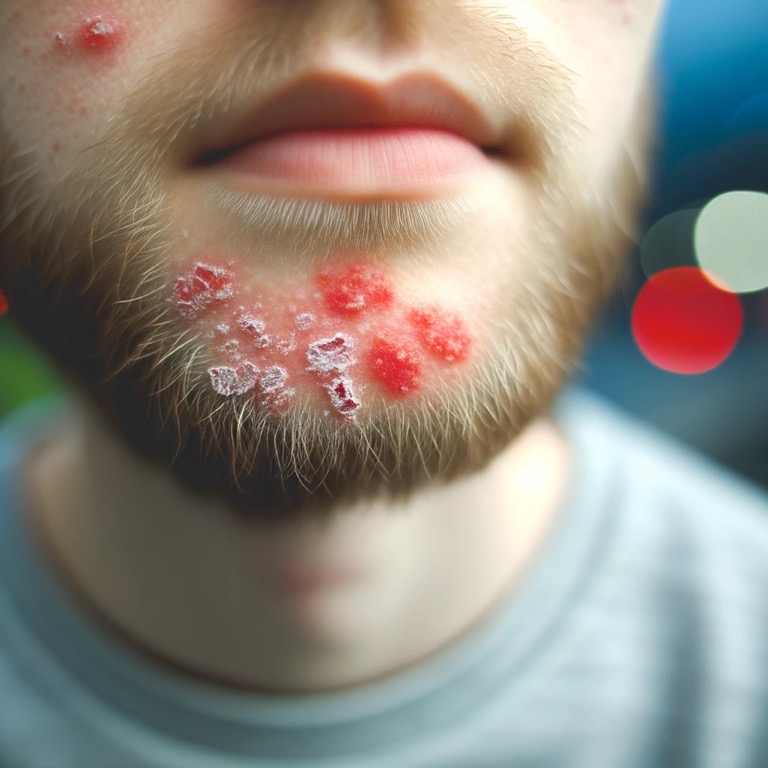

Supplement: Multimedia Appendix 3 [file ai_v3i1e58275_app3.zip › 30.JPG]

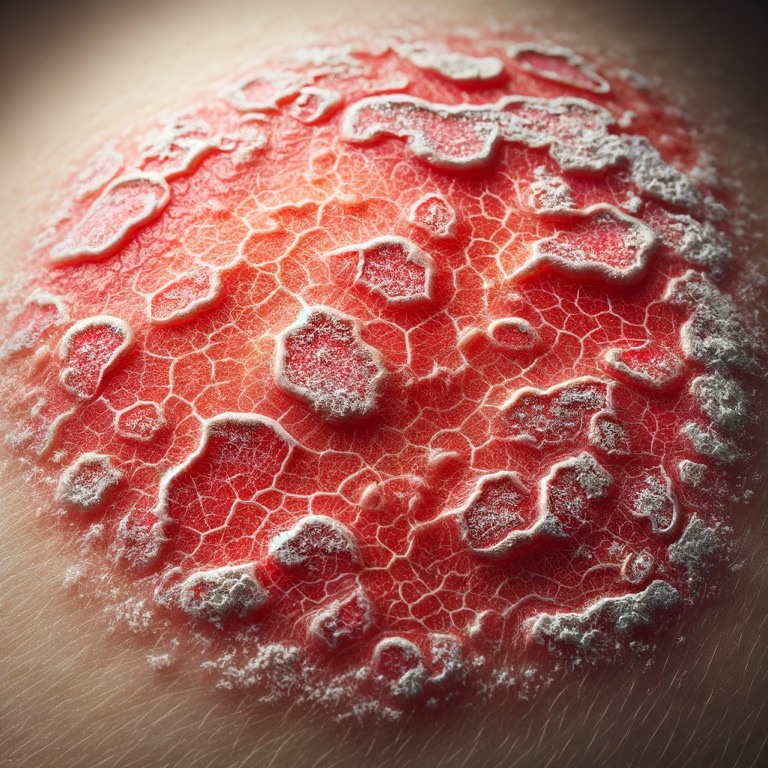

Supplement: Multimedia Appendix 3 [file ai_v3i1e58275_app3.zip › 99.JPG]

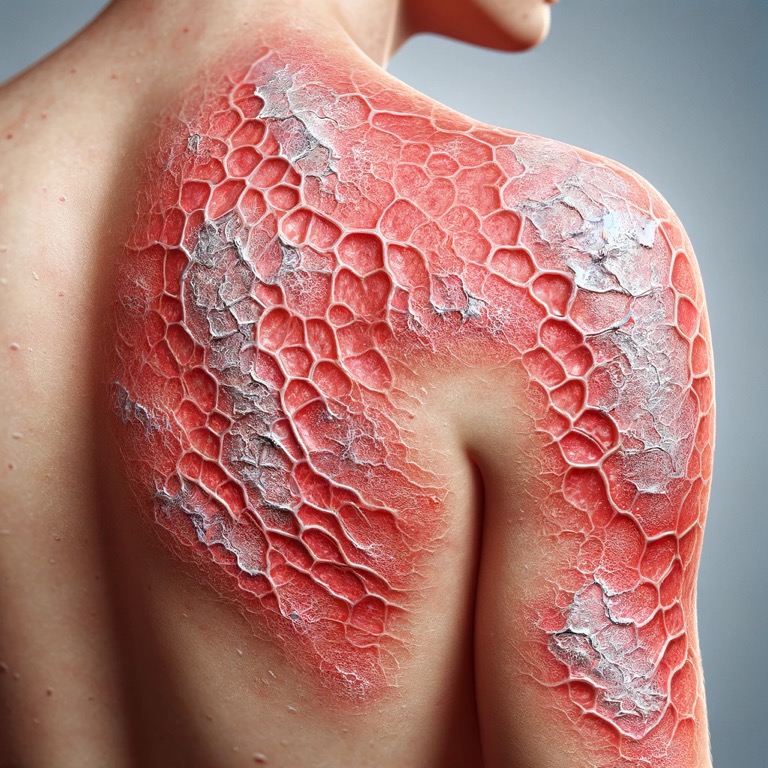

Supplement: Multimedia Appendix 3 [file ai_v3i1e58275_app3.zip › 63.jpeg]

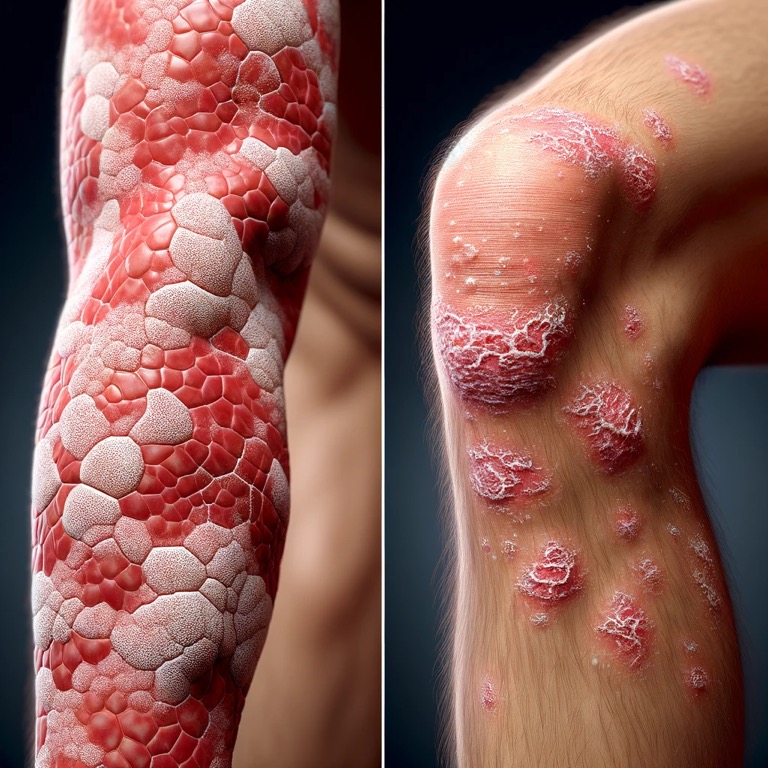

Supplement: Multimedia Appendix 3 [file ai_v3i1e58275_app3.zip › 39+40.jpeg]

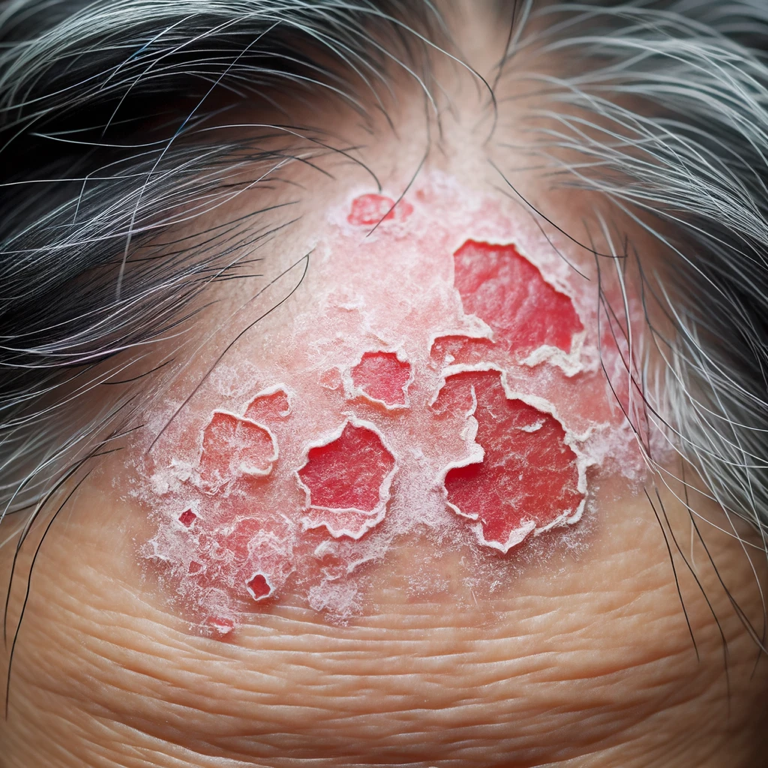

Supplement: Multimedia Appendix 3 [file ai_v3i1e58275_app3.zip › 01.PNG]

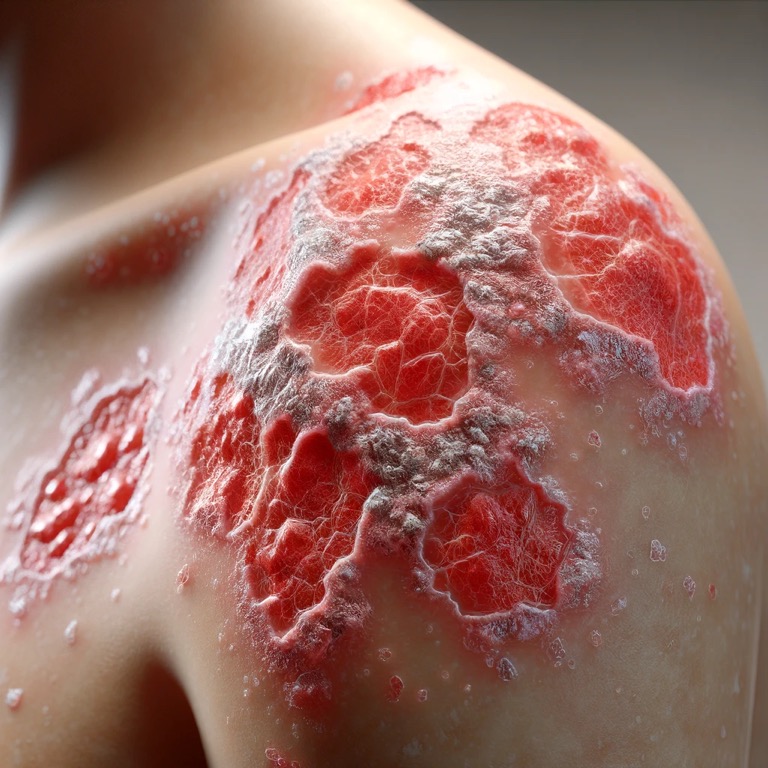

Supplement: Multimedia Appendix 3 [file ai_v3i1e58275_app3.zip › 33.jpeg]

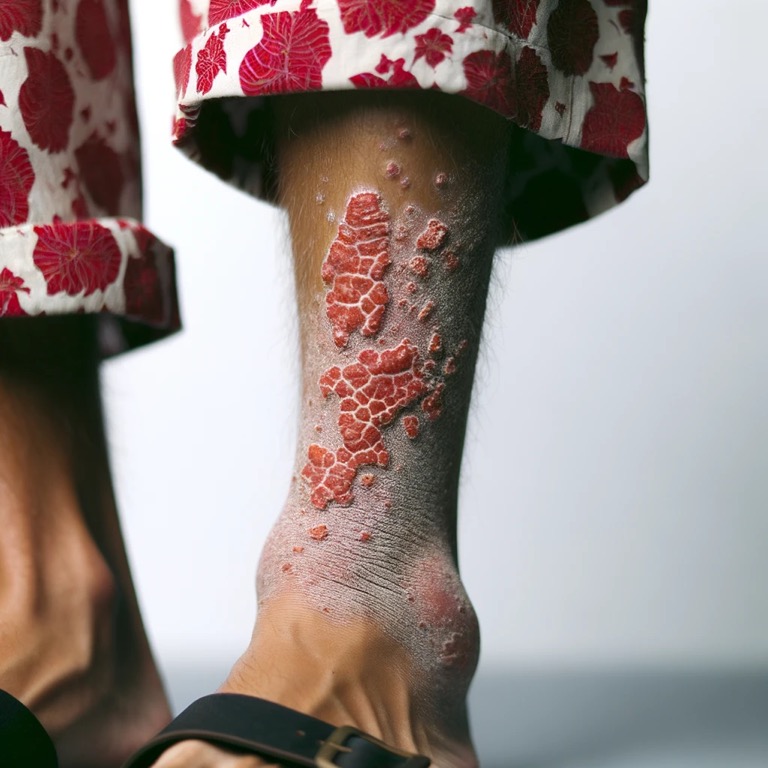

Supplement: Multimedia Appendix 3 [file ai_v3i1e58275_app3.zip › 07.JPG]

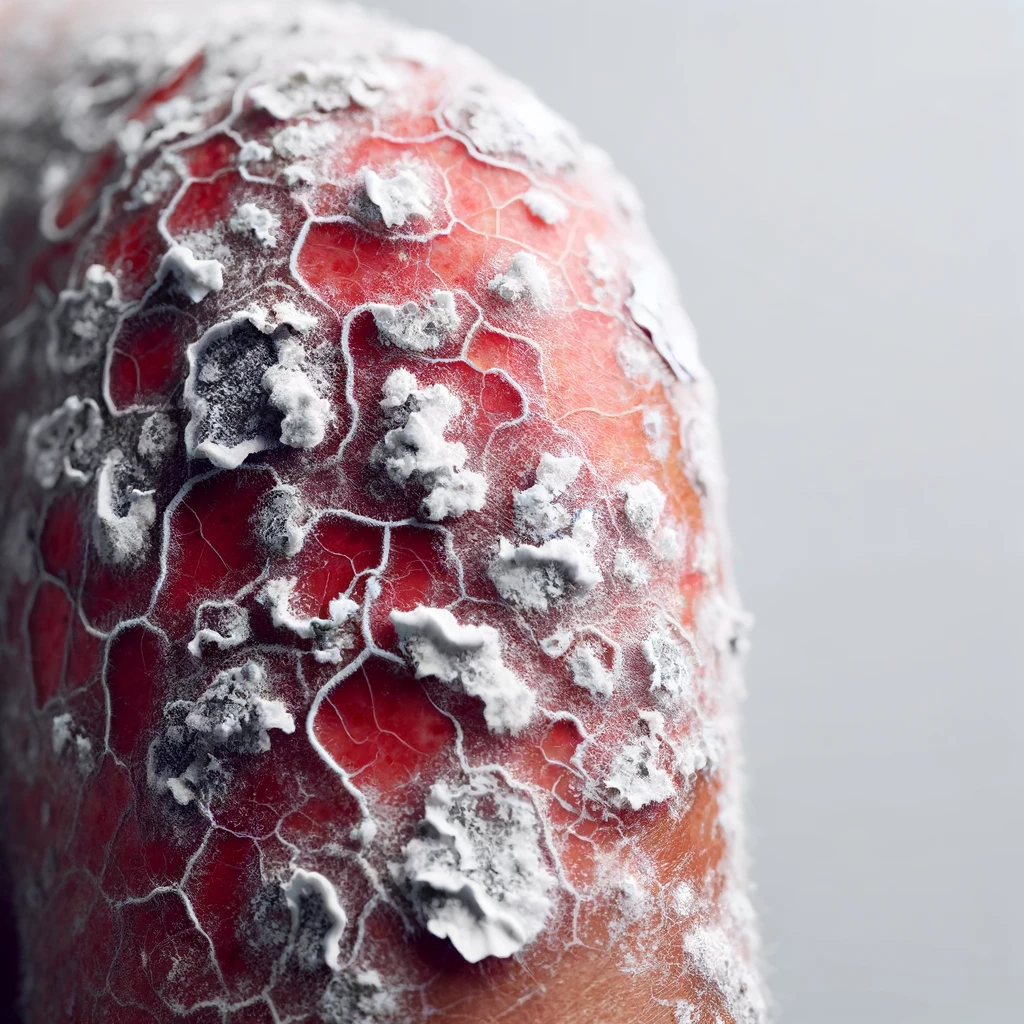

Supplement: Multimedia Appendix 3 [file ai_v3i1e58275_app3.zip › 86.WEBP]

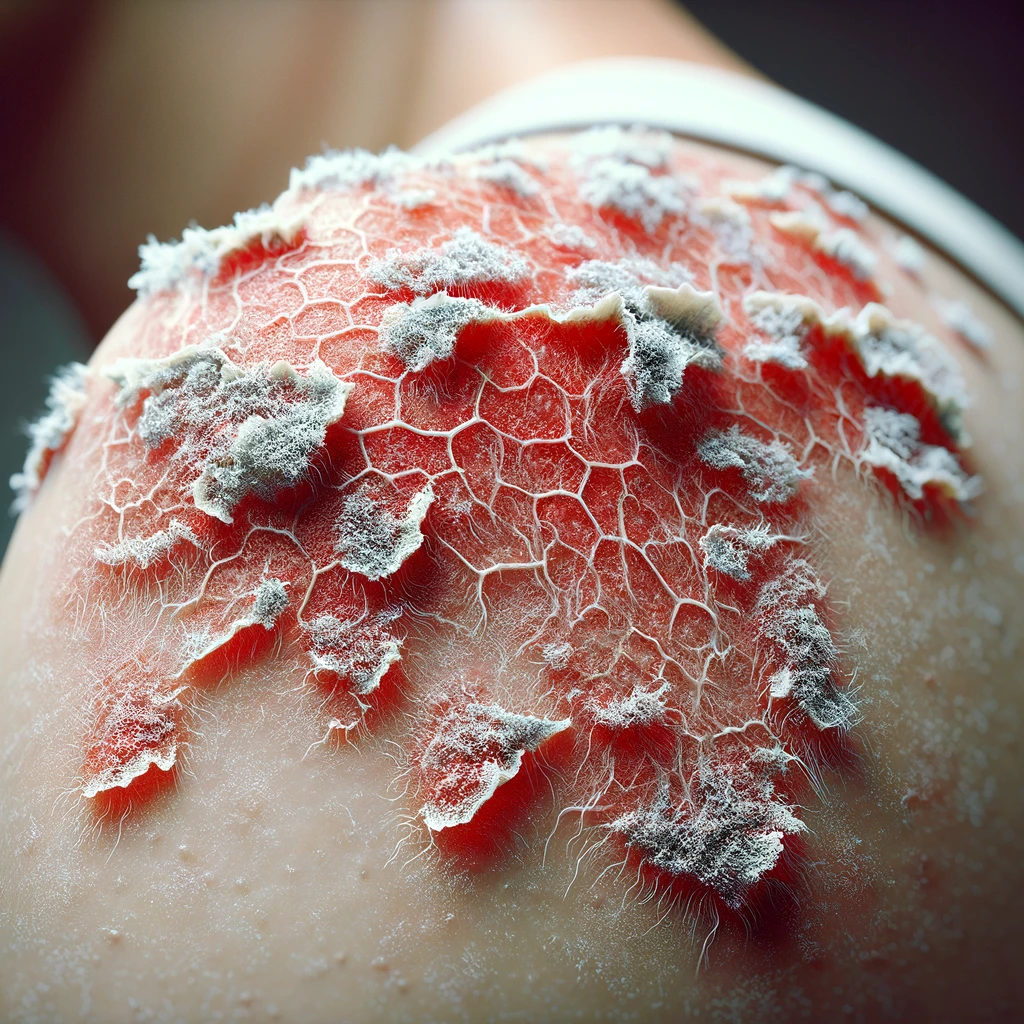

Supplement: Multimedia Appendix 3 [file ai_v3i1e58275_app3.zip › 60.WEBP]

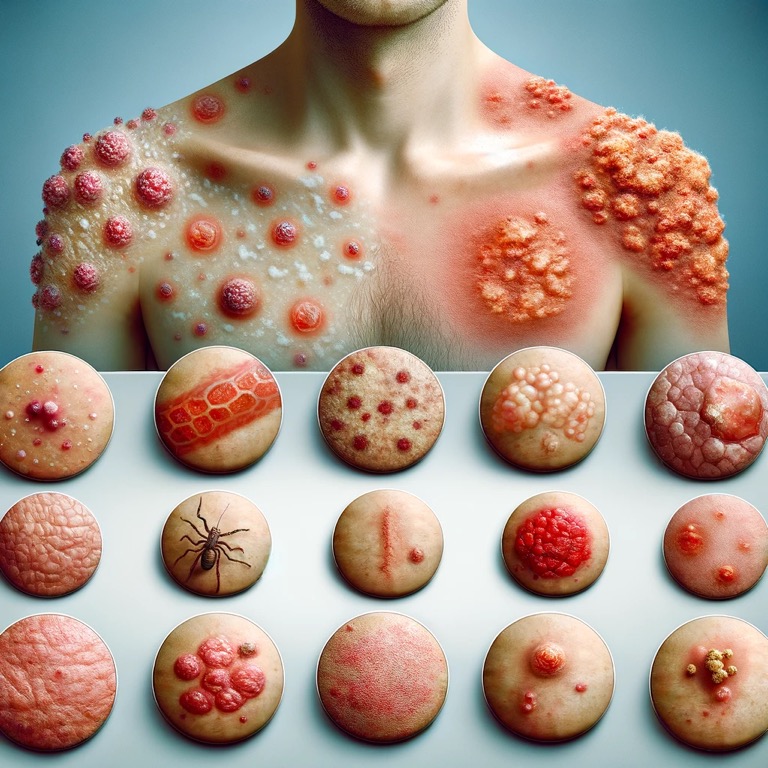

Supplement: Multimedia Appendix 3 [file ai_v3i1e58275_app3.zip › 32 (main image).jpeg]

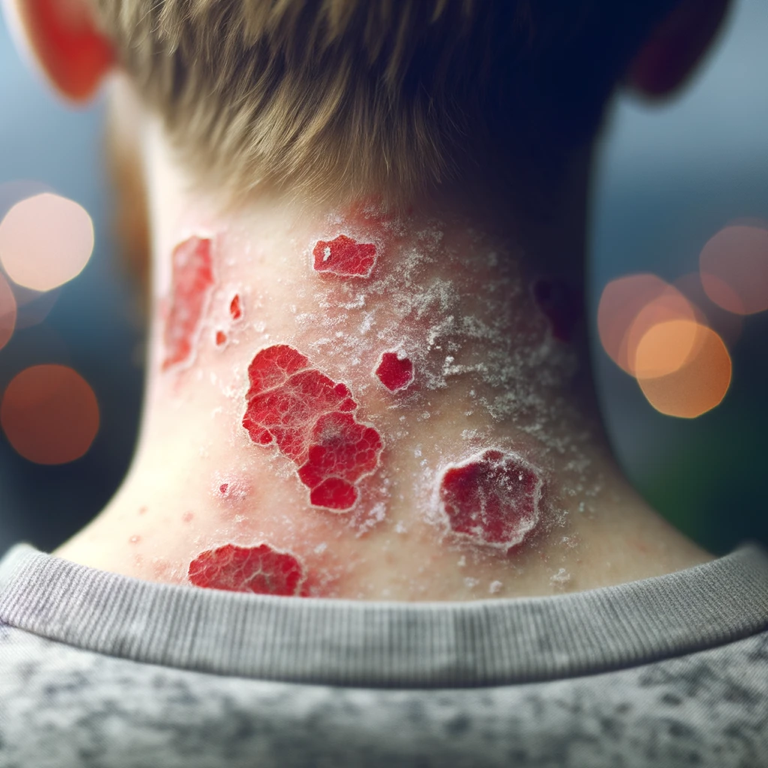

Supplement: Multimedia Appendix 3 [file ai_v3i1e58275_app3.zip › 11.PNG]

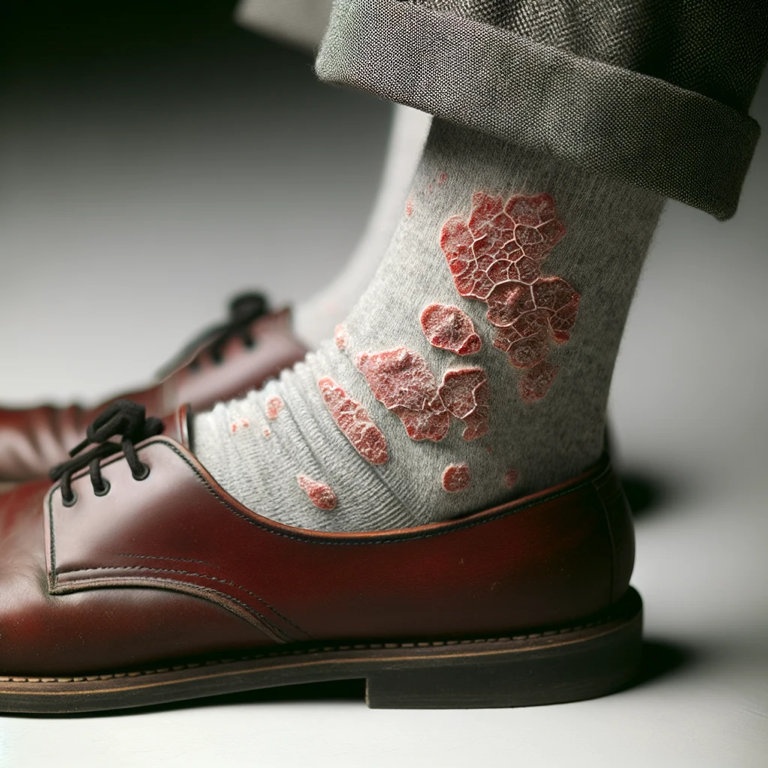

Supplement: Multimedia Appendix 3 [file ai_v3i1e58275_app3.zip › 12.PNG]

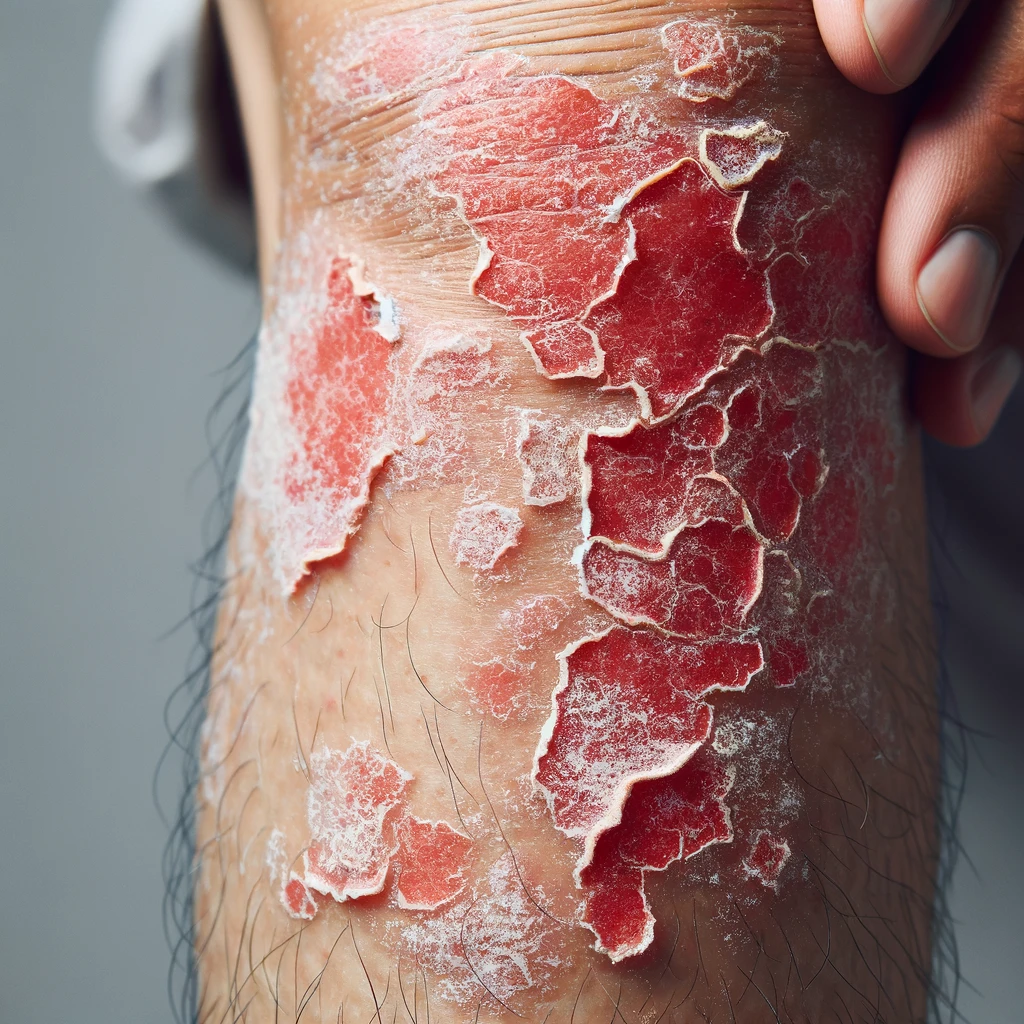

Supplement: Multimedia Appendix 3 [file ai_v3i1e58275_app3.zip › 87.WEBP]

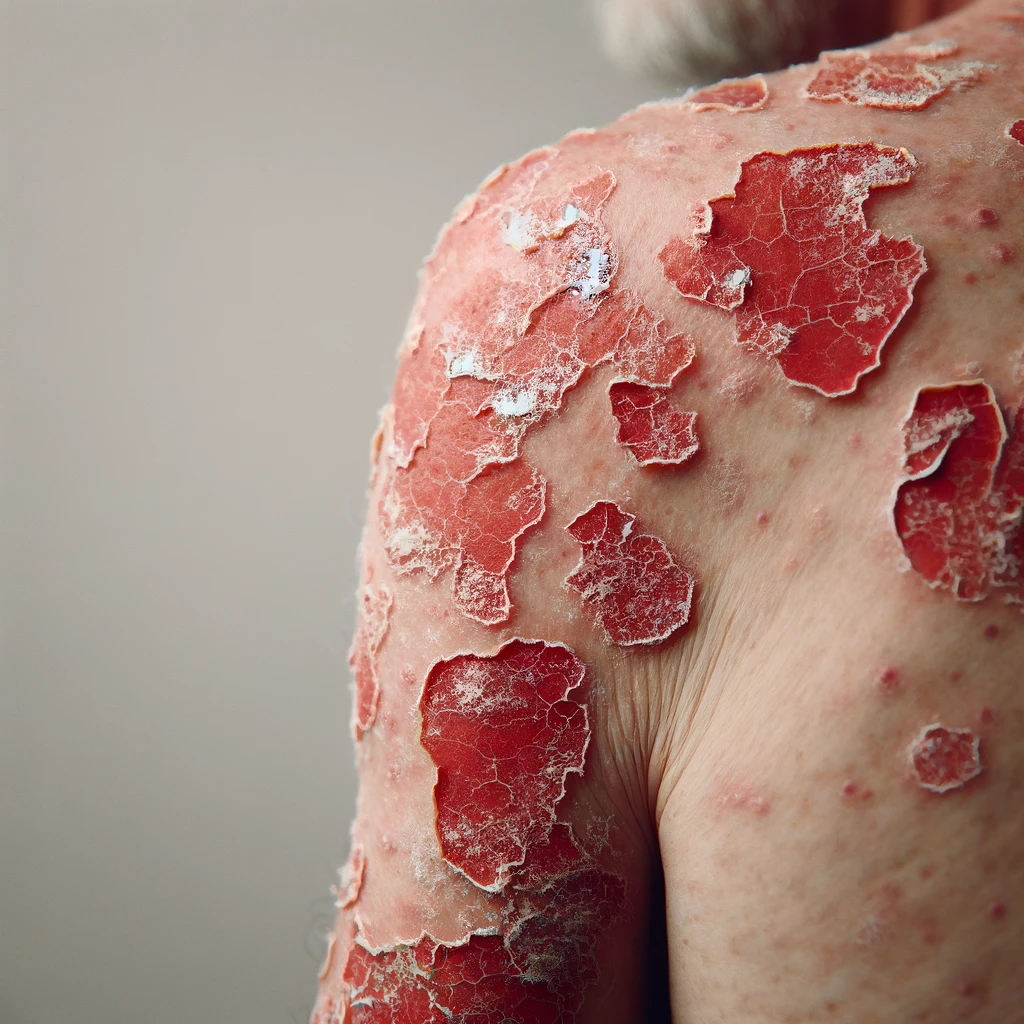

Supplement: Multimedia Appendix 3 [file ai_v3i1e58275_app3.zip › 71.WEBP]

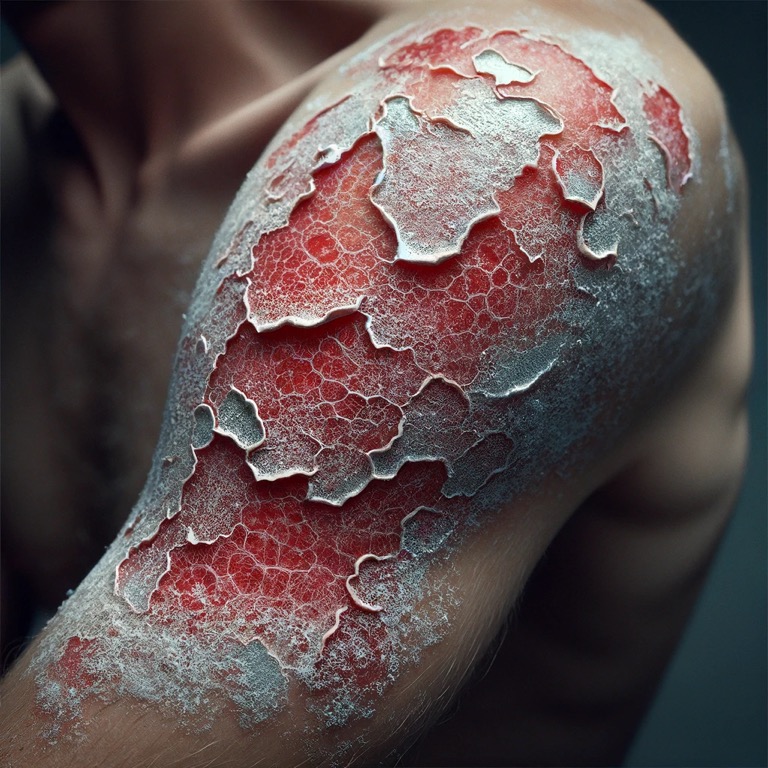

Supplement: Multimedia Appendix 3 [file ai_v3i1e58275_app3.zip › 98.JPG]

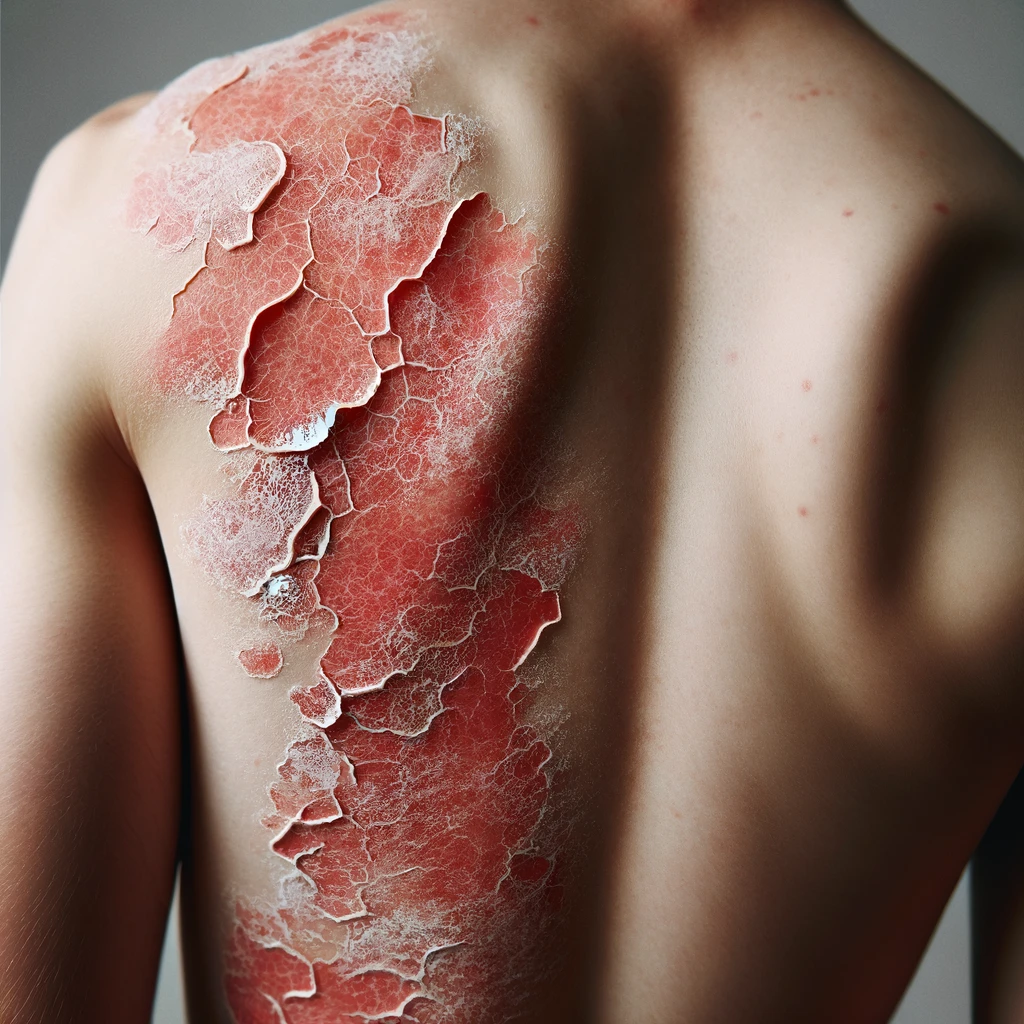

Supplement: Multimedia Appendix 3 [file ai_v3i1e58275_app3.zip › 82.WEBP]

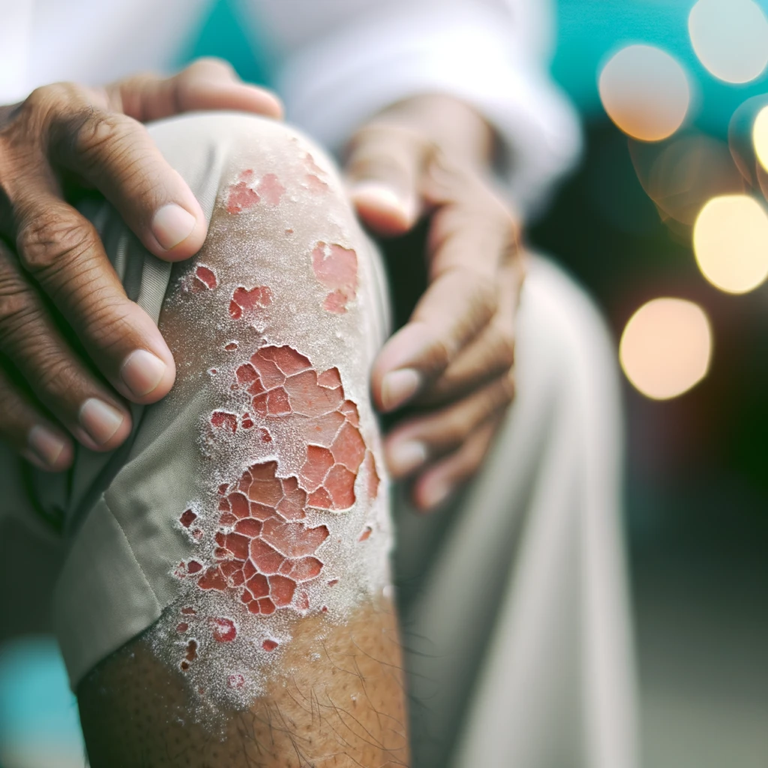

Supplement: Multimedia Appendix 3 [file ai_v3i1e58275_app3.zip › 04.PNG]

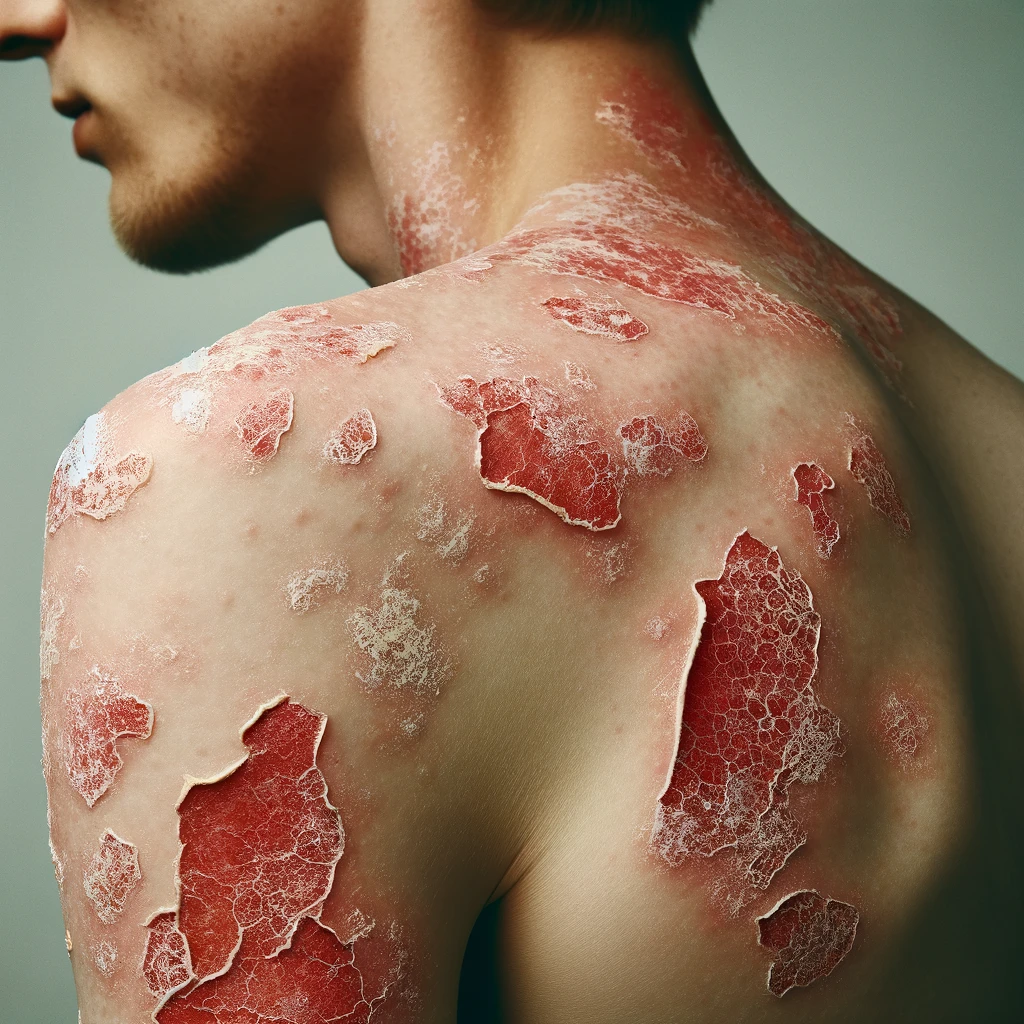

Supplement: Multimedia Appendix 3 [file ai_v3i1e58275_app3.zip › 67.WEBP]

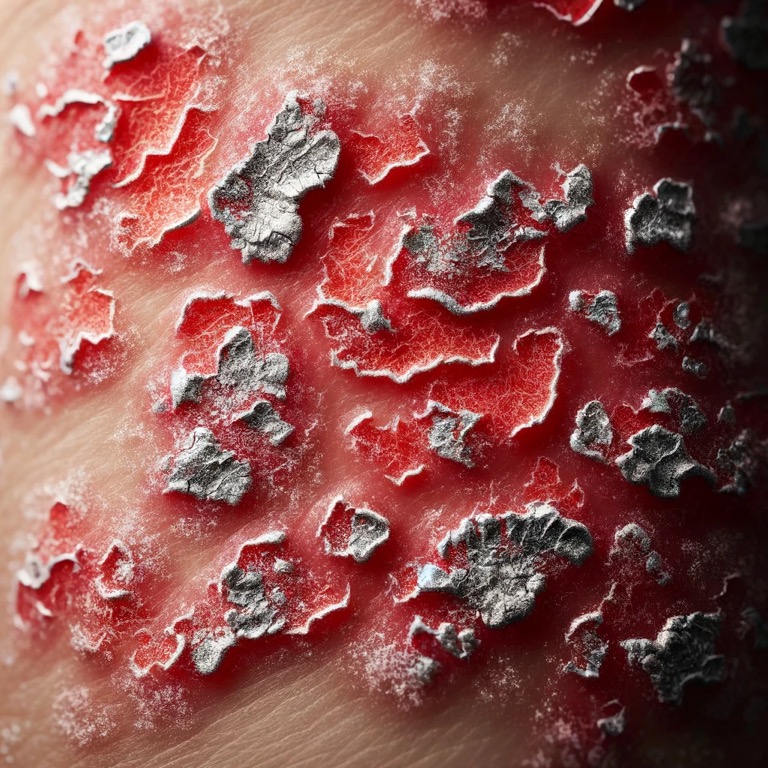

Supplement: Multimedia Appendix 3 [file ai_v3i1e58275_app3.zip › 100.JPG]
