# Supplementary figures and images for: Ensuring Appropriate Representation in Artificial Intelligence–Generated Medical Imagery: Protocol for a Methodological Approach to Address Skin Tone Bias (part 3 of 3)
Source: JMIR AI. 2024 Nov 27;3:e58275. doi: 10.2196/58275 (PMC11635324; doi:10.2196/58275)

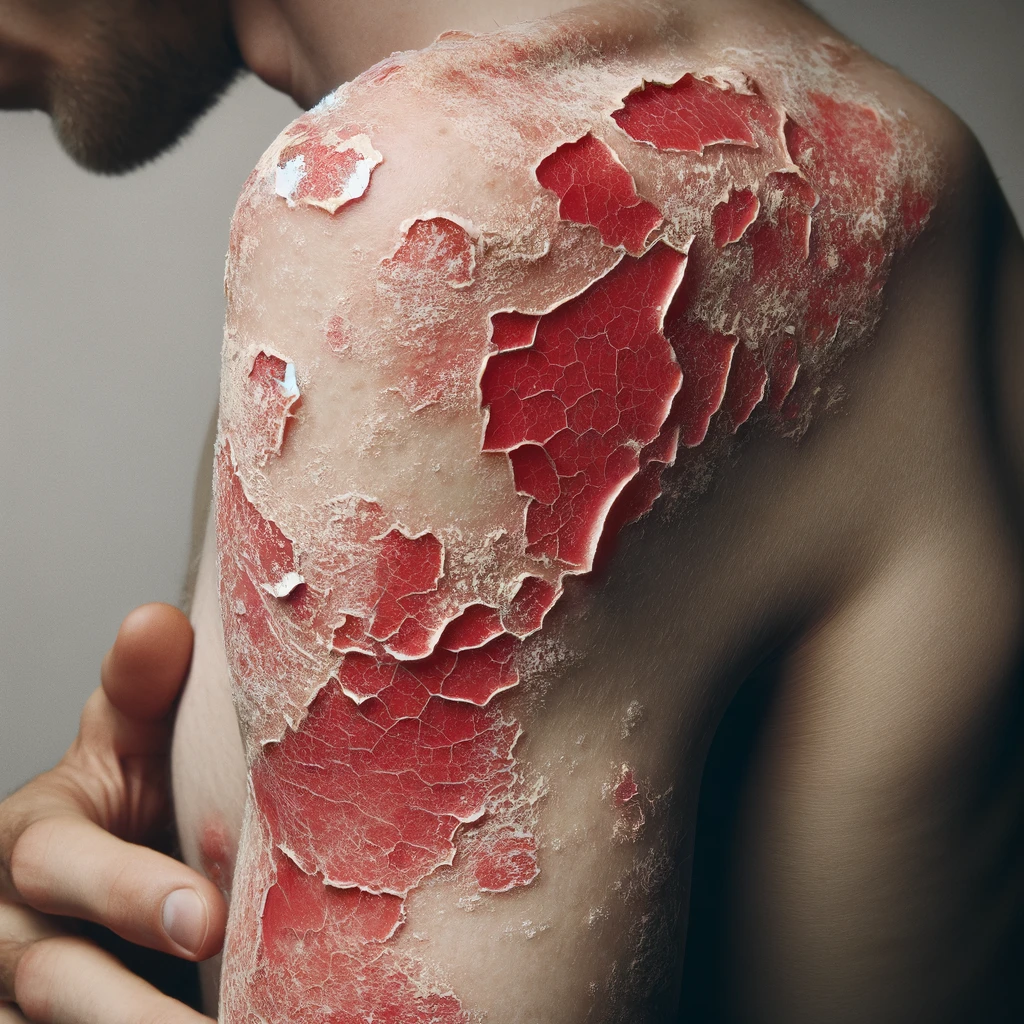

Supplement: Multimedia Appendix 3 [file ai_v3i1e58275_app3.zip › 76.WEBP]

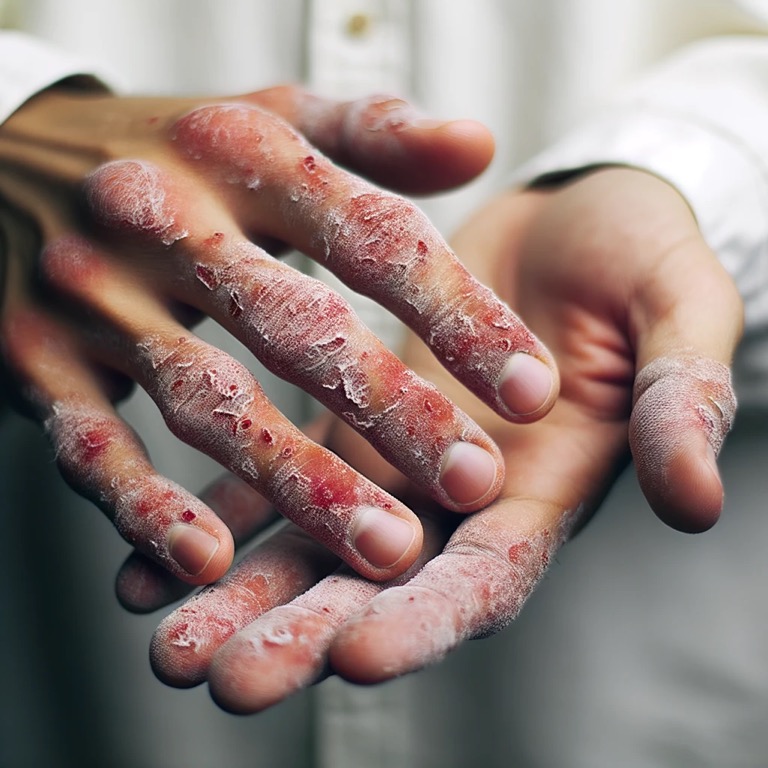

Supplement: Multimedia Appendix 3 [file ai_v3i1e58275_app3.zip › 23.JPG]

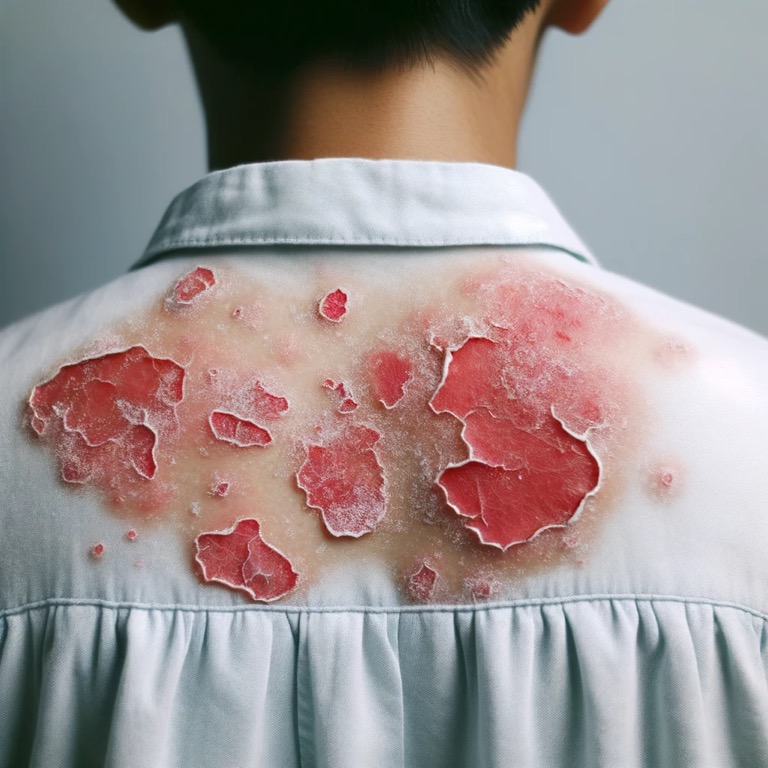

Supplement: Multimedia Appendix 3 [file ai_v3i1e58275_app3.zip › 18.JPG]

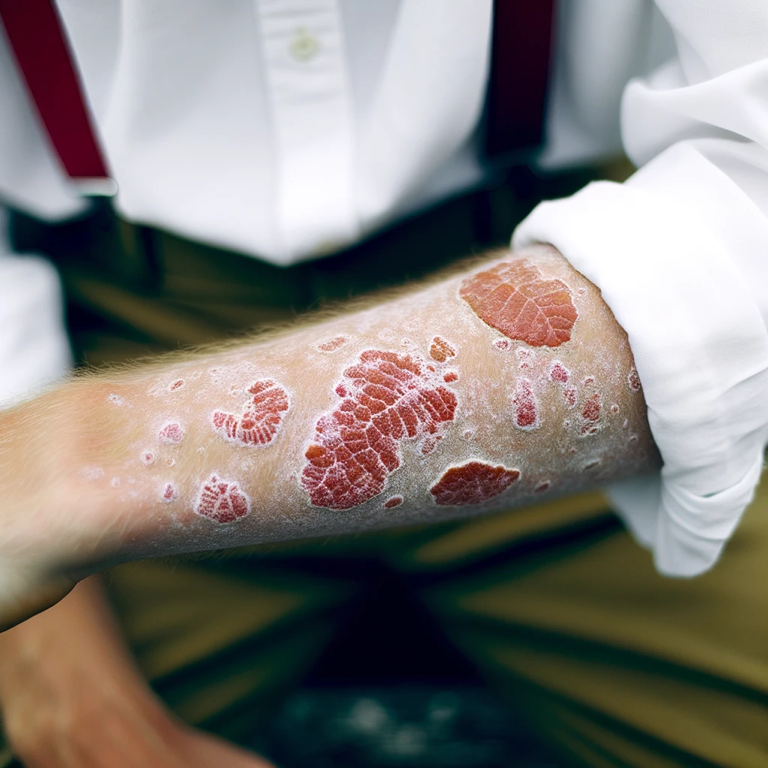

Supplement: Multimedia Appendix 3 [file ai_v3i1e58275_app3.zip › 05.PNG]

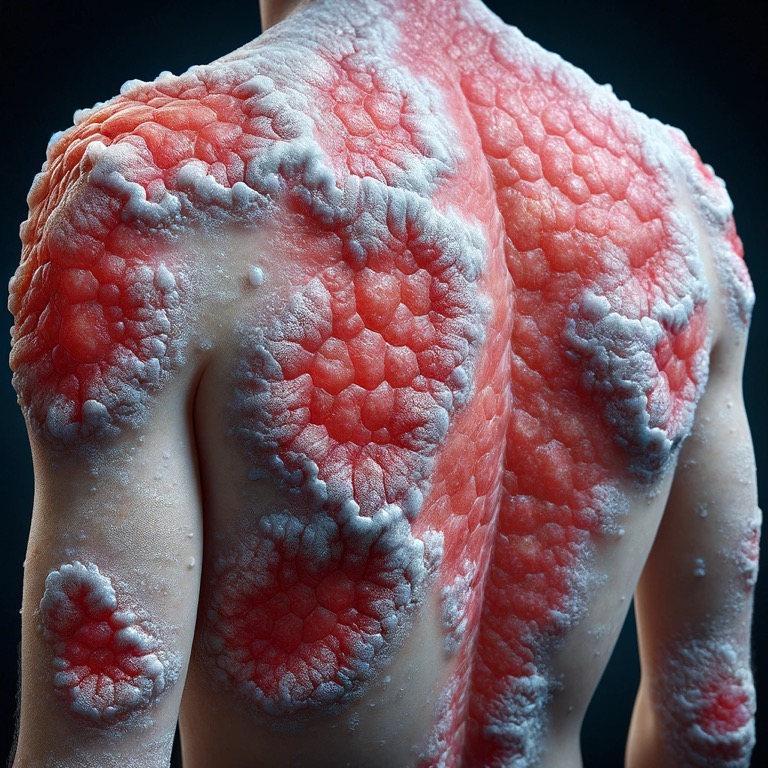

Supplement: Multimedia Appendix 3 [file ai_v3i1e58275_app3.zip › 41.jpeg]

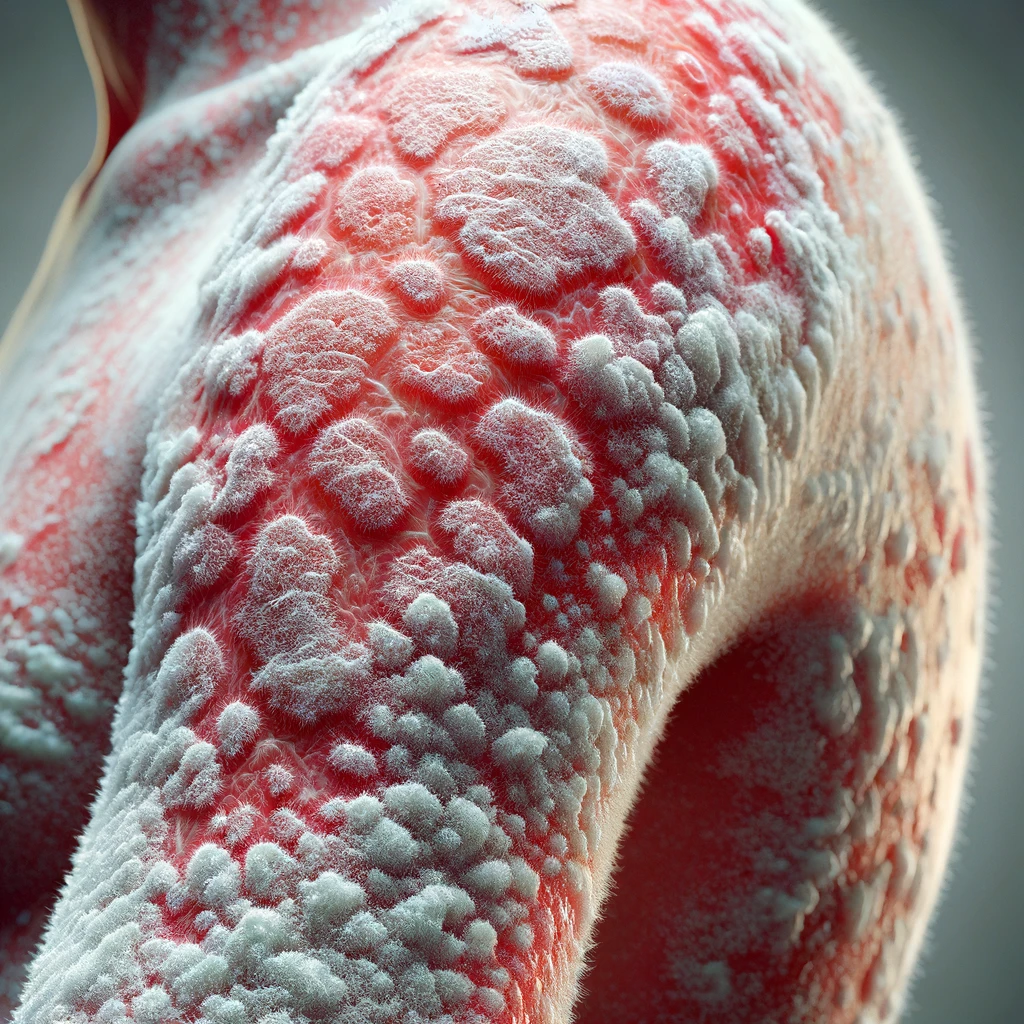

Supplement: Multimedia Appendix 3 [file ai_v3i1e58275_app3.zip › 61.WEBP]

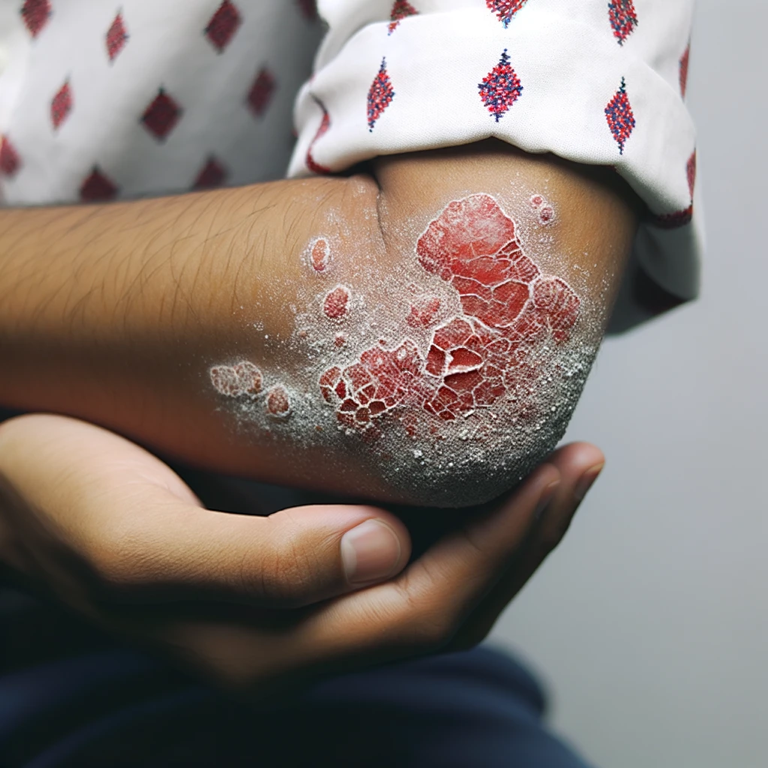

Supplement: Multimedia Appendix 3 [file ai_v3i1e58275_app3.zip › 03.PNG]

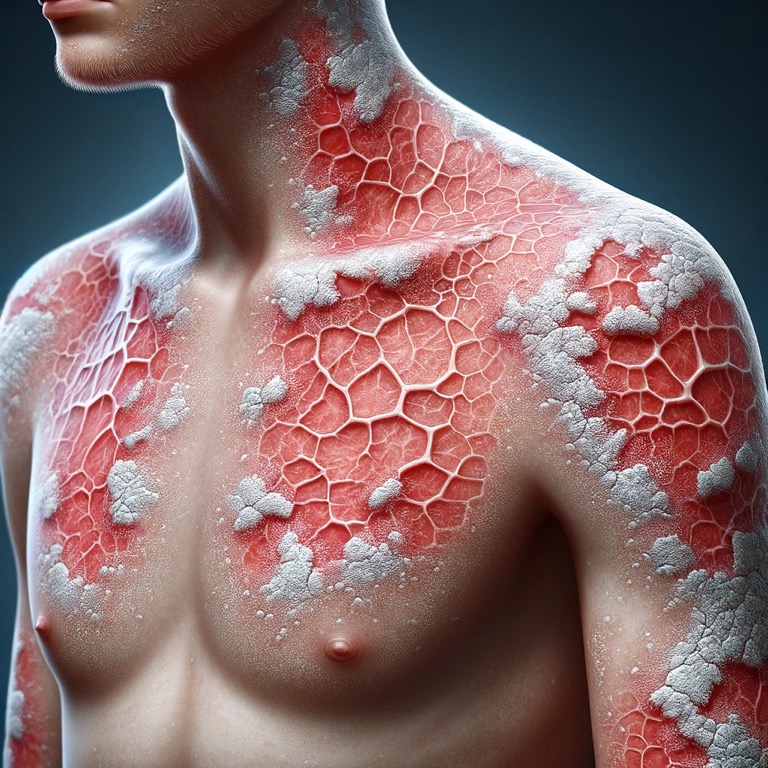

Supplement: Multimedia Appendix 3 [file ai_v3i1e58275_app3.zip › 43.jpeg]

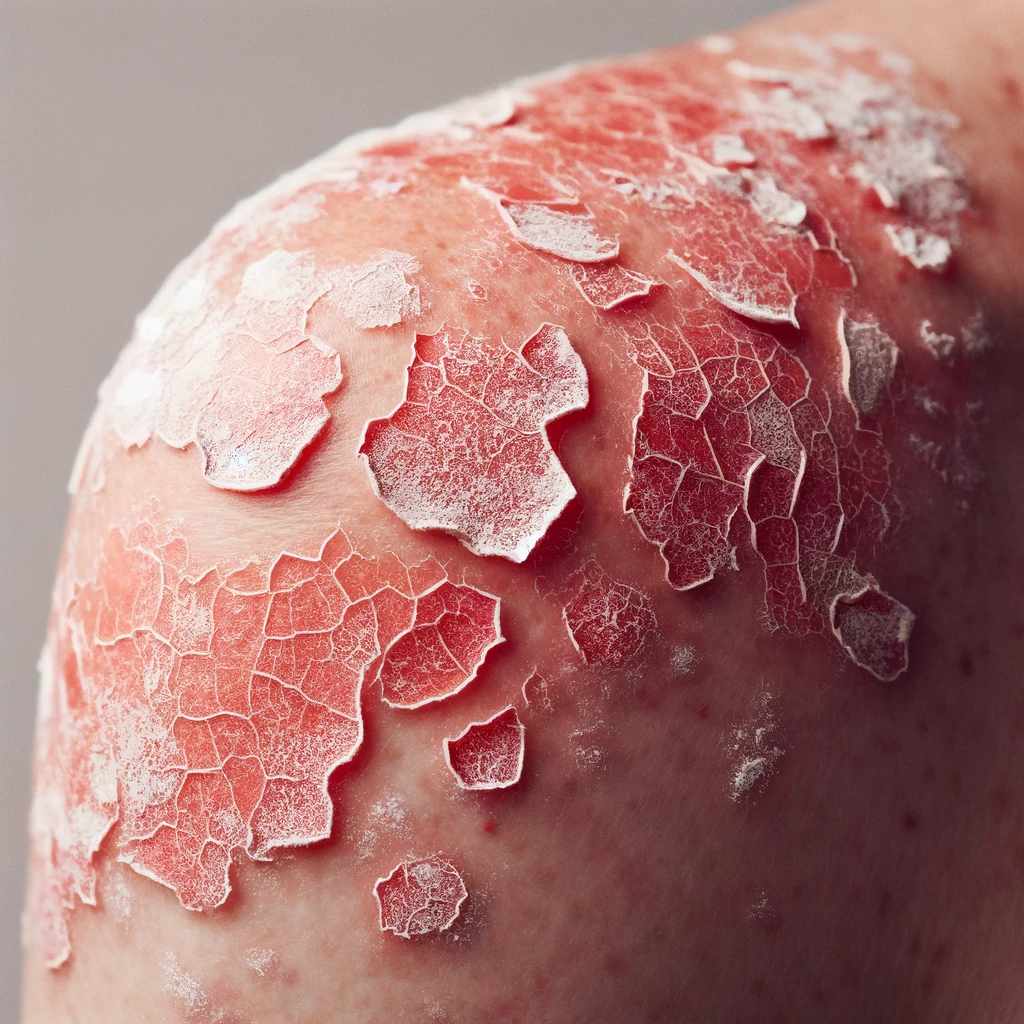

Supplement: Multimedia Appendix 3 [file ai_v3i1e58275_app3.zip › 92.WEBP]

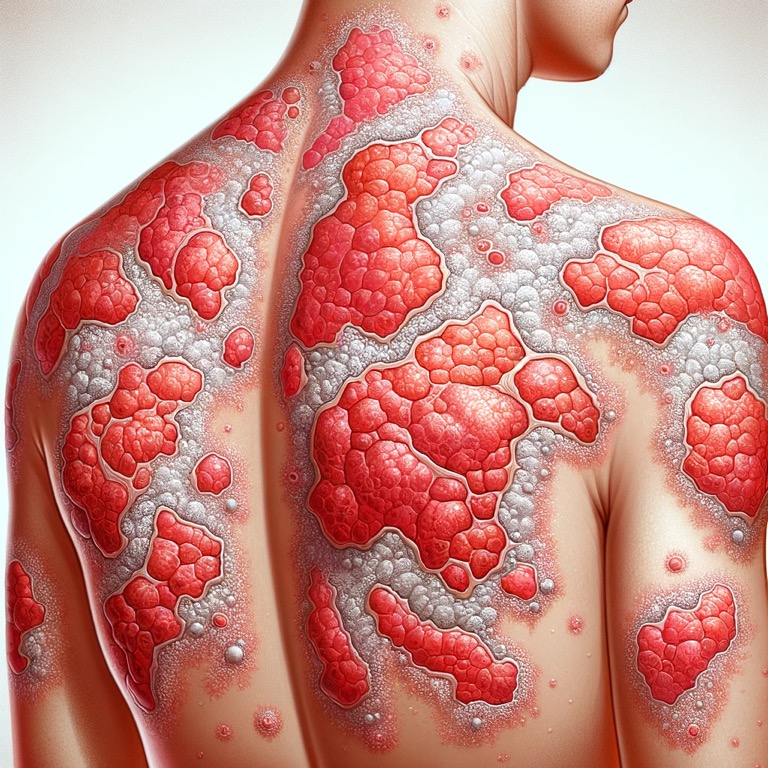

Supplement: Multimedia Appendix 3 [file ai_v3i1e58275_app3.zip › 56.jpeg]

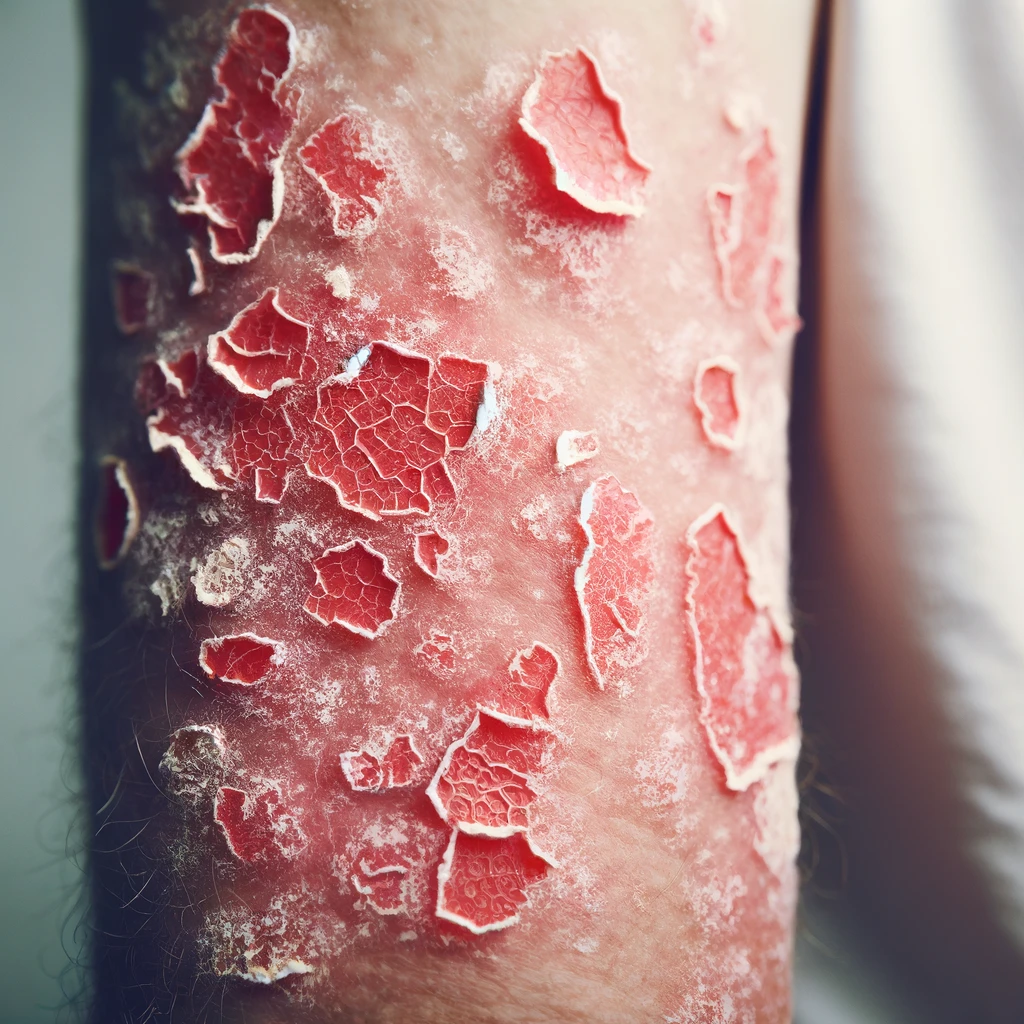

Supplement: Multimedia Appendix 3 [file ai_v3i1e58275_app3.zip › 89.WEBP]

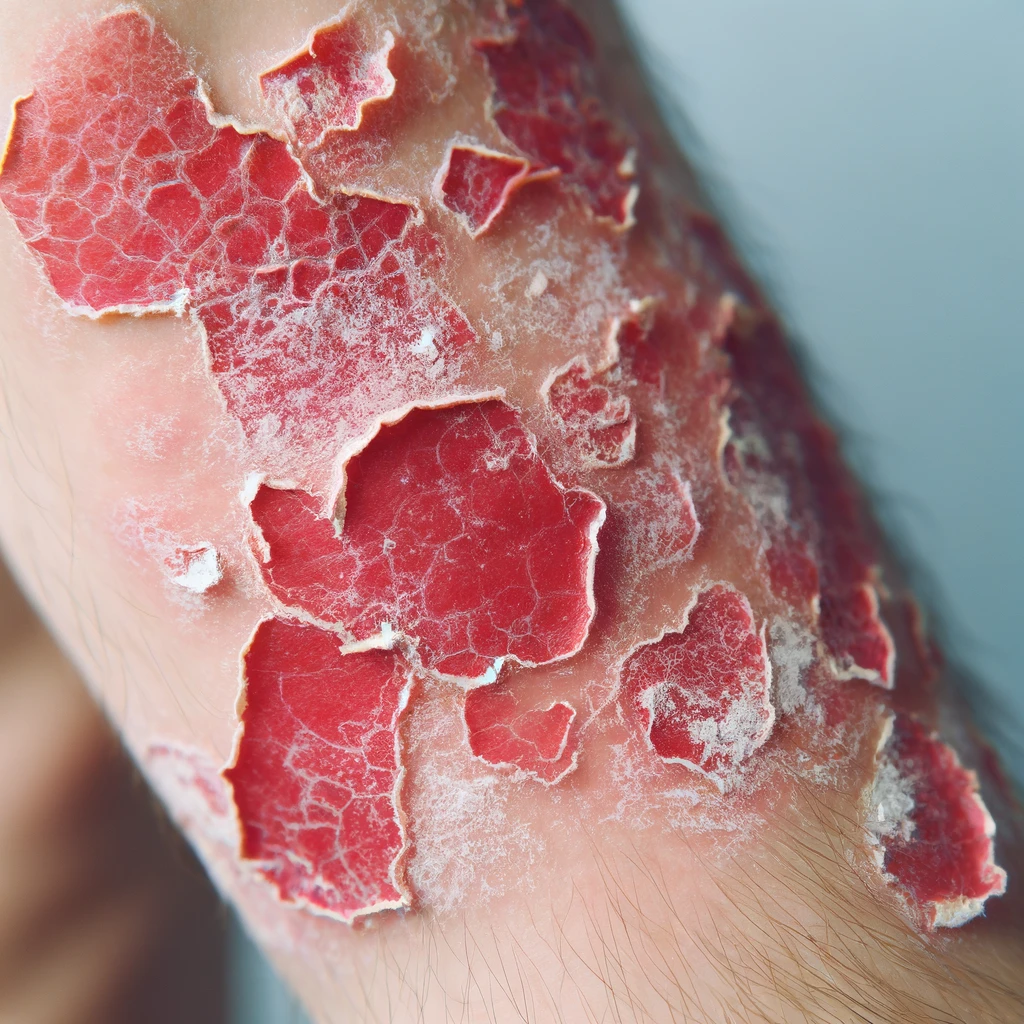

Supplement: Multimedia Appendix 3 [file ai_v3i1e58275_app3.zip › 80.WEBP]

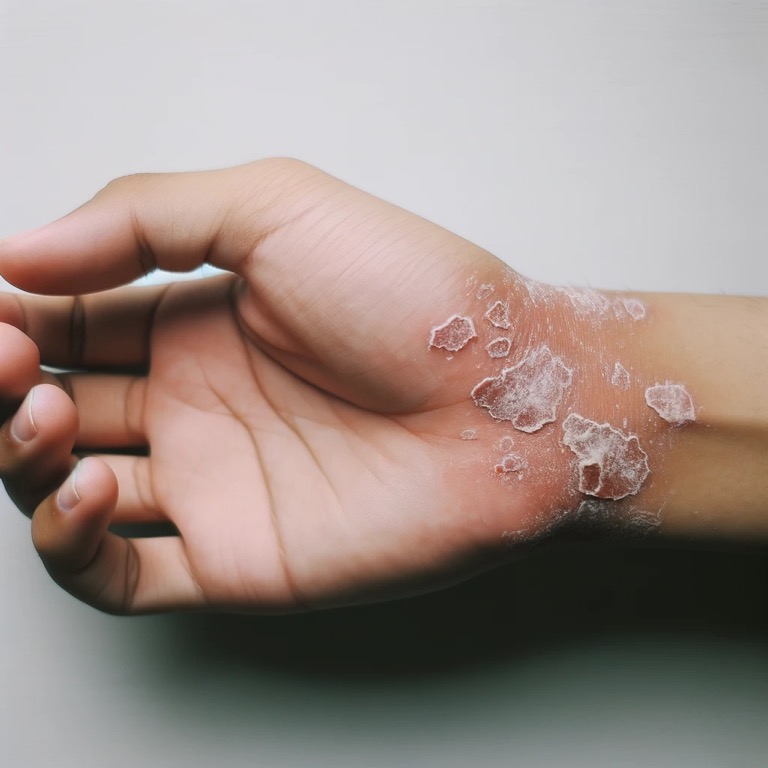

Supplement: Multimedia Appendix 3 [file ai_v3i1e58275_app3.zip › 31.JPG]

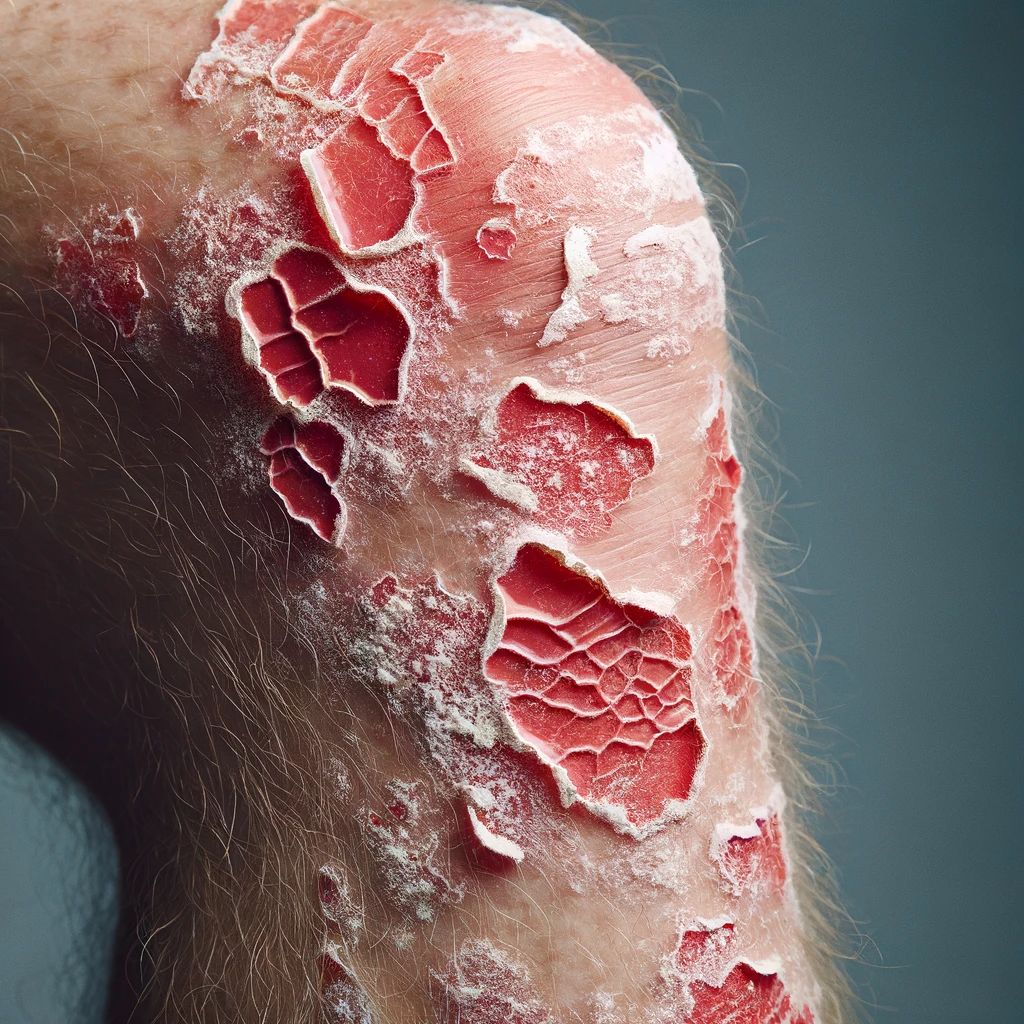

Supplement: Multimedia Appendix 3 [file ai_v3i1e58275_app3.zip › 75.WEBP]

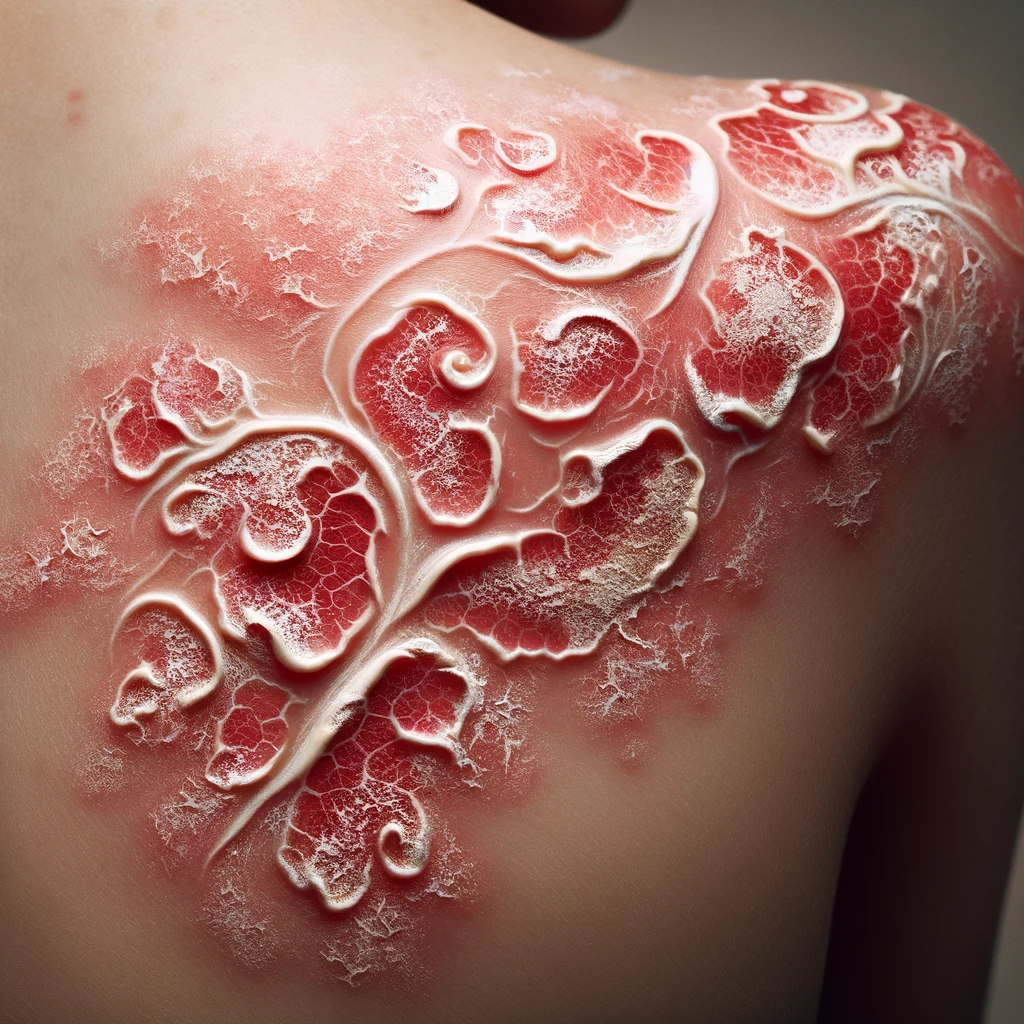

Supplement: Multimedia Appendix 3 [file ai_v3i1e58275_app3.zip › 90.WEBP]

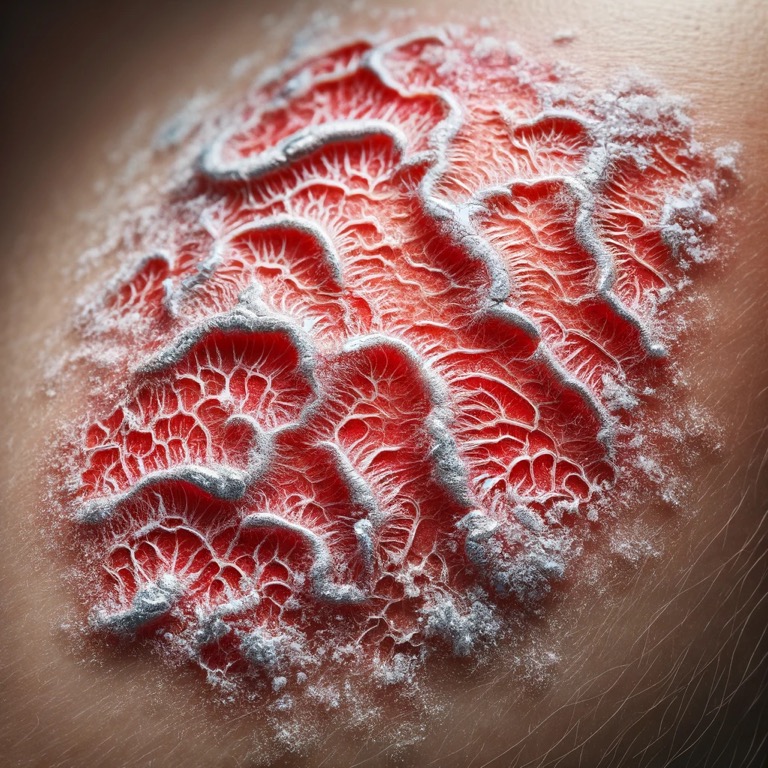

Supplement: Multimedia Appendix 3 [file ai_v3i1e58275_app3.zip › 97.JPG]

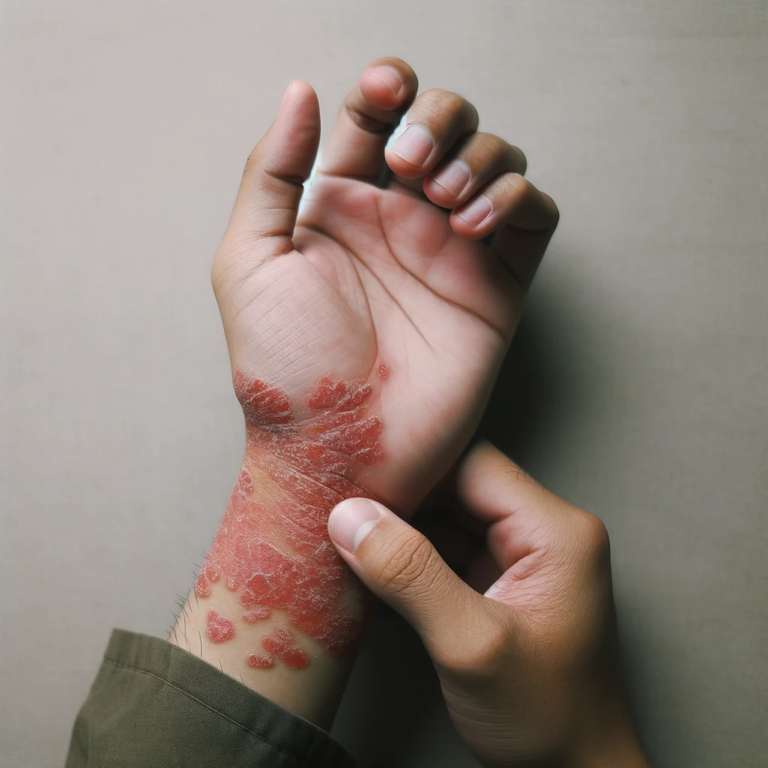

Supplement: Multimedia Appendix 3 [file ai_v3i1e58275_app3.zip › 06.PNG]

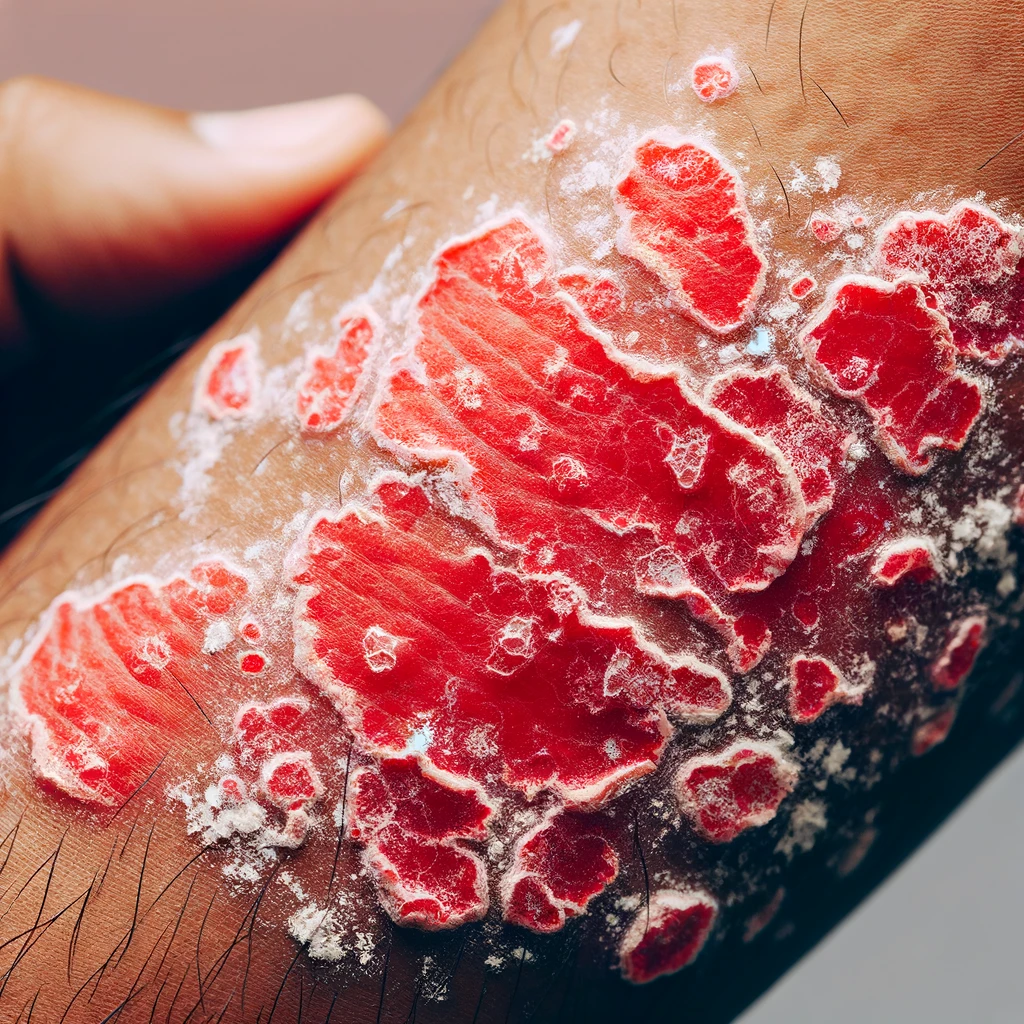

Supplement: Multimedia Appendix 3 [file ai_v3i1e58275_app3.zip › 72.WEBP]

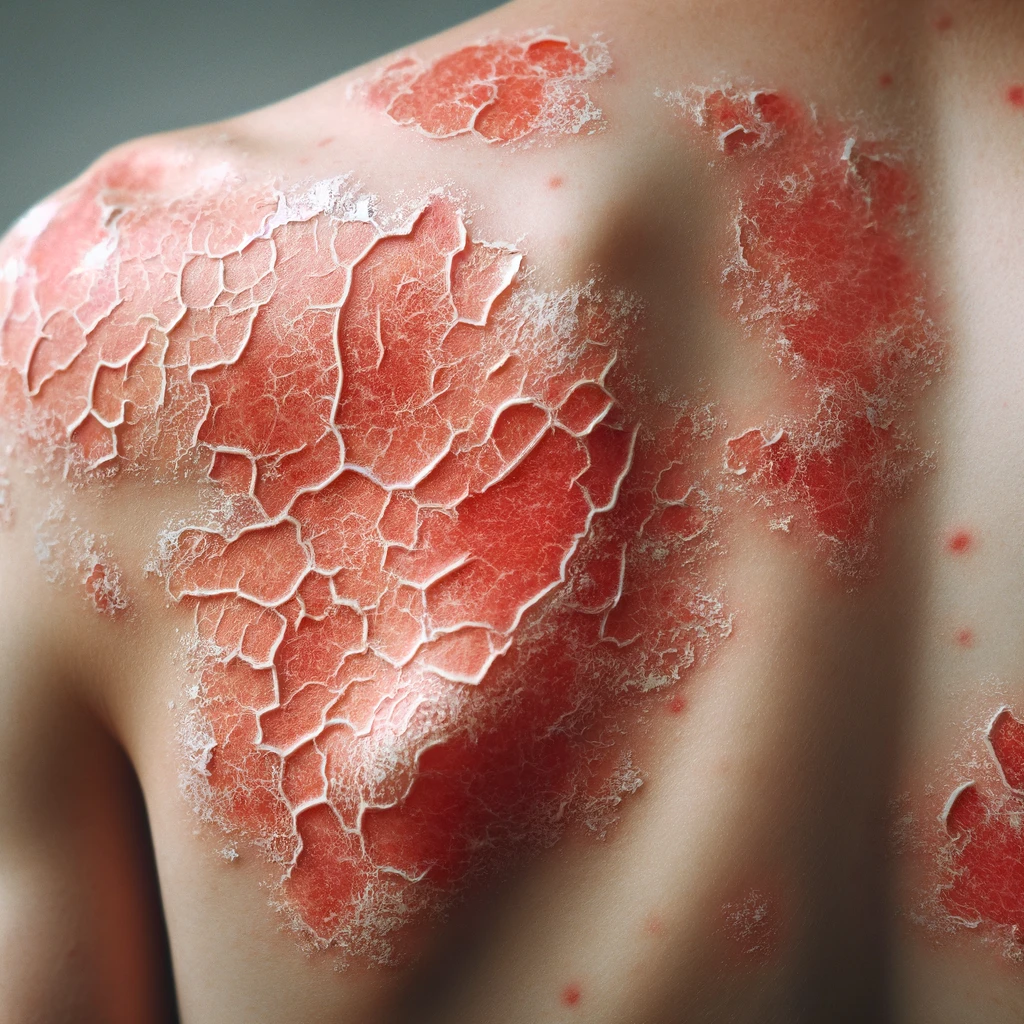

Supplement: Multimedia Appendix 3 [file ai_v3i1e58275_app3.zip › 64.WEBP]

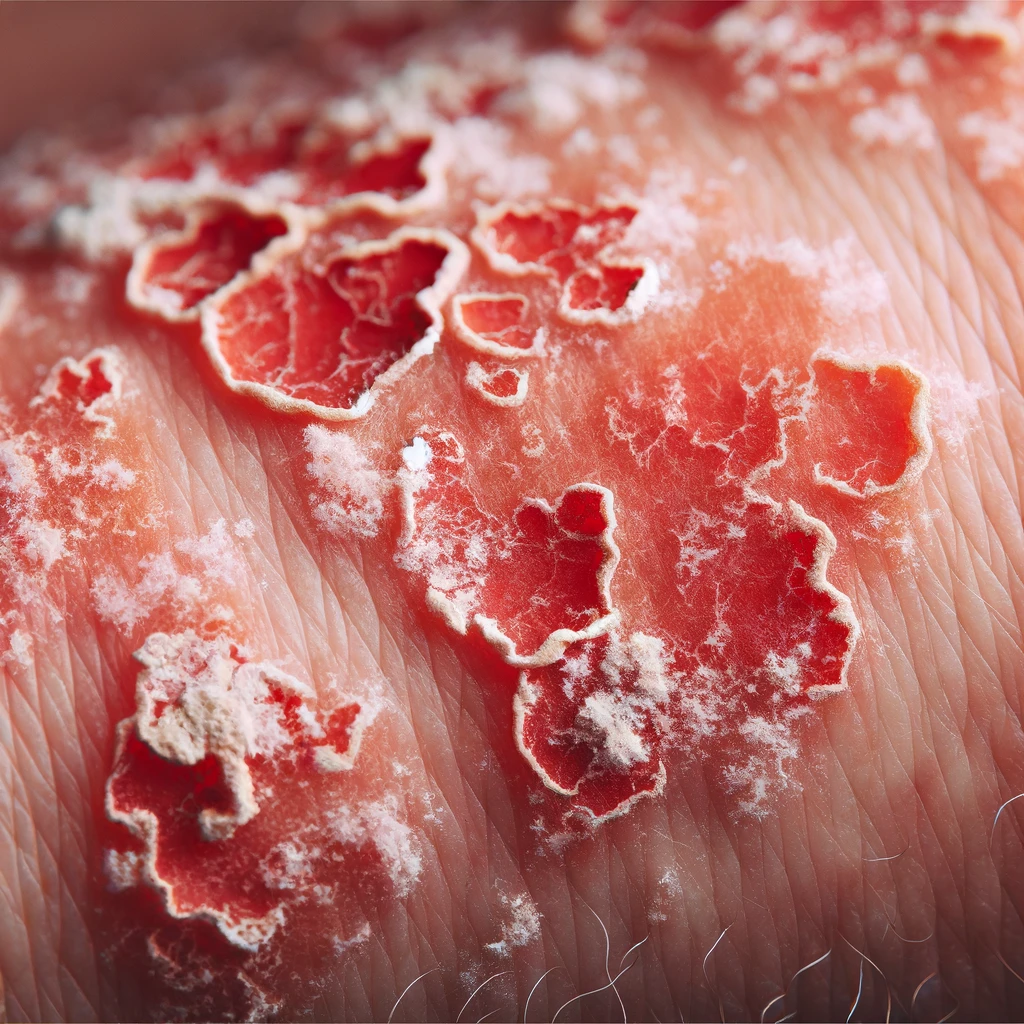

Supplement: Multimedia Appendix 3 [file ai_v3i1e58275_app3.zip › 95.WEBP]

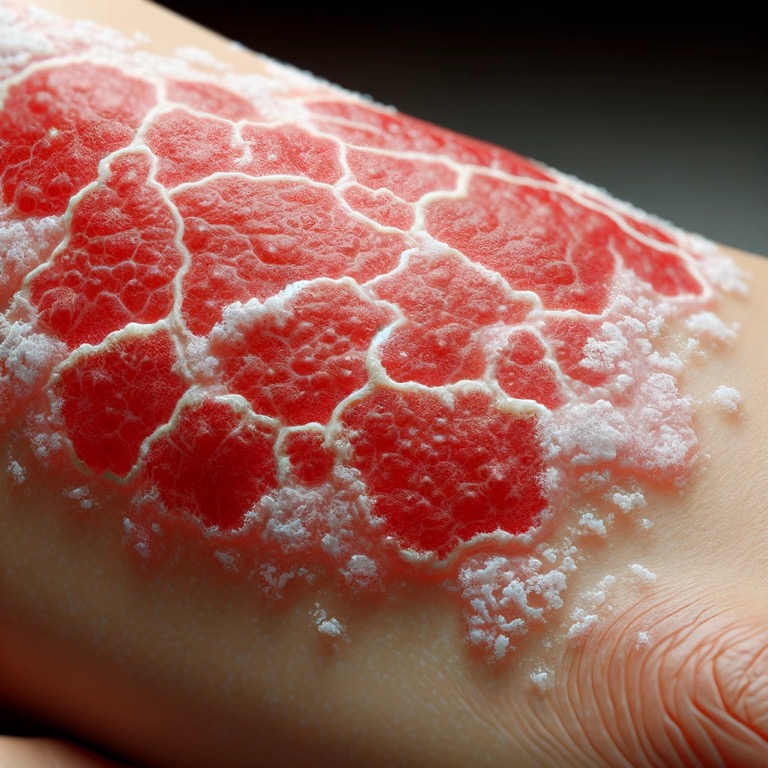

Supplement: Multimedia Appendix 3 [file ai_v3i1e58275_app3.zip › 44.jpeg]

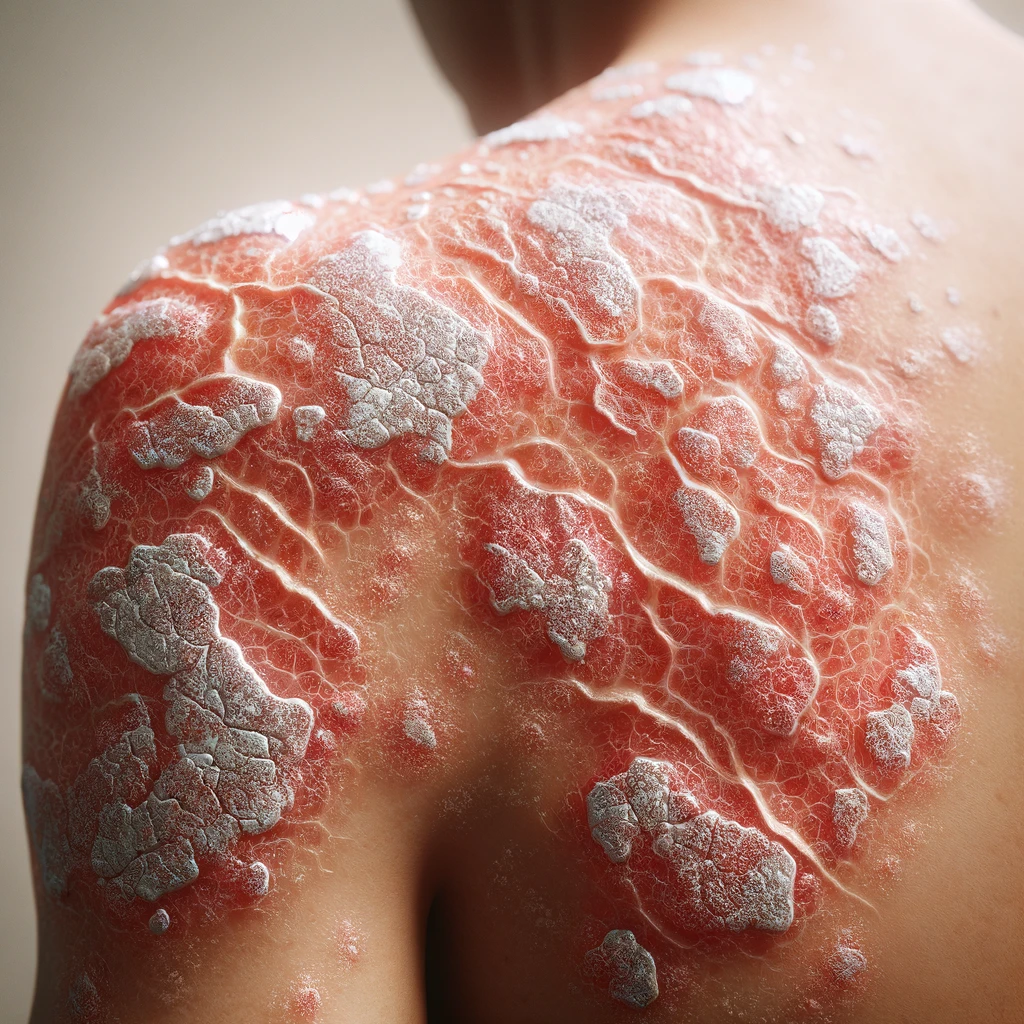

Supplement: Multimedia Appendix 3 [file ai_v3i1e58275_app3.zip › 58.WEBP]
